# ^31^P Nuclear Magnetic Resonance Spectroscopy as a Probe of Thorium–Phosphorus Bond Covalency: Correlating Phosphorus Chemical Shift to Metal–Phosphorus Bond Order

**DOI:** 10.1021/jacs.3c02775

**Published:** 2023-09-28

**Authors:** Jingzhen Du, Joseph Hurd, John A. Seed, Gábor Balázs, Manfred Scheer, Ralph W. Adams, Daniel Lee, Stephen T. Liddle

**Affiliations:** † Department of Chemistry, The University of Manchester, Oxford Road, Manchester, M13 9PL, U.K.; ‡ Department of Chemical Engineering, The University of Manchester, Oxford Road, Manchester, M13 9PL, U.K.; § Institute of Inorganic Chemistry, University of Regensburg, Universitätsstr. 31, 93053 Regensburg, Germany

## Abstract

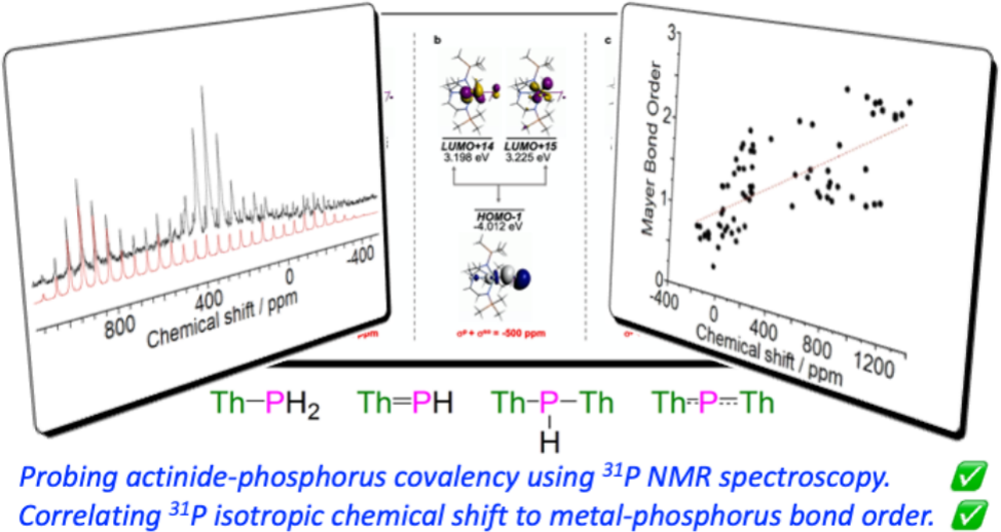

We report the use of solution and solid-state ^31^P Nuclear Magnetic Resonance (NMR) spectroscopy combined with Density Functional Theory calculations to benchmark the covalency of actinide-phosphorus bonds, thus introducing ^31^P NMR spectroscopy to the investigation of molecular f-element chemical bond covalency. The ^31^P NMR data for [Th(PH_2_)(Tren^TIPS^)] (**1**, Tren^TIPS^ = {N(CH_2_CH_2_NSiPr^i^_3_)_3_}^3–^), [Th(PH)(Tren^TIPS^)][Na(12C4)_2_] (**2**, 12C4 = 12-crown-4 ether), [{Th(Tren^TIPS^)}_2_(μ-PH)] (**3**), and [{Th(Tren^TIPS^)}_2_(μ-P)][Na(12C4)_2_] (**4**) demonstrate a chemical shift anisotropy (CSA) ordering of (μ-P)^3–^ > (=PH)^2–^ > (μ-PH)^2–^ > (−PH_2_)^1–^ and for **4** the largest CSA for any bridging phosphido unit. The B3LYP functional with 50% Hartree–Fock mixing produced spin–orbit δ_iso_ values that closely match the experimental data, providing experimentally benchmarked quantification of the nature and extent of covalency in the Th–P linkages in **1**–**4** via Natural Bond Orbital and Natural Localized Molecular Orbital analyses. Shielding analysis revealed that the ^31^P δ_iso_ values are essentially only due to the nature of the Th–P bonds in **1**–**4**, with largely invariant diamagnetic but variable paramagnetic and spin–orbit shieldings that reflect the Th–P bond multiplicities and s-orbital mediated transmission of spin–orbit effects from Th to P. This study has permitted correlation of Th–P δ_iso_ values to Mayer bond orders, revealing qualitative correlations generally, but which should be examined with respect to specific ancillary ligand families rather than generally to be quantitative, reflecting that ^31^P δ_iso_ values are a very sensitive reporter due to phosphorus being a soft donor that responds to the rest of the ligand field much more than stronger, harder donors like nitrogen.

## Introduction

A longstanding central challenge in actinide (An) chemistry, and indeed across the periodic table, is reliably determining the extent and nature of covalency in An–ligand bonds.^1−3^ This has prompted extensive studies of An–ligand multiple bonds since, apart from realizing novel new structural linkages to compare with long-standing transition metal analogues, arguably An-ligand multiple bonds inherently exhibit the maximum potential for covalency that is amenable to practical study.^4−9^ However, while the fundamentals of Pauling’s model of chemical bonding are straightforward to grasp,^10^ the fine detail is difficult to probe experimentally. Nevertheless, in recent years with ever improving experimental, analytical, and computational methods becoming available, significant advances have been made experimentally probing An-covalency, when underpinned by quantum chemical calculations,^11^ including X-ray absorption spectroscopy,^12−20^ pulsed electron paramagnetic resonance spectroscopy,^21^ and nuclear magnetic resonance (NMR) spectroscopy.^22,23^

Where the use of NMR spectroscopy to probe An–ligand covalency is concerned, solution and solid-state (SS) studies include ^1^H,^24,25^^13^C,^25−34^^15^N,^35−37^^17^O,^38−41^^19^F,^42−45^^29^Si,^46^^35/37^Cl,^47^^77^Se,^48^ and ^125^Te^48^ nuclei, which when taken together has revealed an intimate relationship between chemical shift (δ) properties and the nature of the An–ligand bond. This in turn can afford in-depth information about shielding (σ) tensors and hence the ability to experimentally benchmark and probe An–ligand bonding and thus covalency in great detail. However, one nucleus conspicuous by its absence to date in molecular f-element NMR covalency studies is ^31^P, which is surprising given that ^31^P is a 100% abundant *I* = 1/2 nucleus and hence an extremely attractive and prevalent nucleus for NMR studies generally. However, the notable absence of ^31^P NMR covalency studies in the molecular An domain to date could be that in Hard–Soft Acid–Base (HSAB) theory Ans and P are hard and soft nuclei, respectively, and thus poorly matched, a situation exacerbated when considering that metal–ligand multiple bond systems usually by definition require metal centers in mid- to high-oxidation states (typically +4 to +6) which increases their hardness. It is also the case that many An complexes are paramagnetic, so the range of diamagnetic An–P derivatives is relatively limited, and although Th(IV) is diamagnetic, its bonding is usually more ionic than that of, for example, U, and hence synthesizing and isolating stable Th–ligand multiple bond linkages is often more challenging for the larger Th compared to U on HSAB and steric reasons. Furthermore, to realistically use ^31^P NMR spectroscopy for covalency studies a family of related molecules with varied An–P bond types is required to rigorously construct the necessary framework approach where a single molecular example would not suffice, and there are relatively few complexes with direct An–P bonds that can be described as meeting that criterion.^49−71^ It is also the case that An–P bonds are generally prone to decomposition in the presence of air or moisture, making studies more experimentally challenging to undertake. Lastly, another challenge of incorporating ^31^P NMR as a tool for studying An-covalency is that the ^31^P nucleus is exceedingly sensitive to its environment (bond lengths, hybridization, molecular dynamics, formal molecule charge, phase, solvent), which can render rigorous and systematic understanding challenging to acquire,^72^ again highlighting the need for structurally related An–P molecules from which to validate the use of ^31^P NMR spectroscopy in probing covalency.

**Figure 1:**
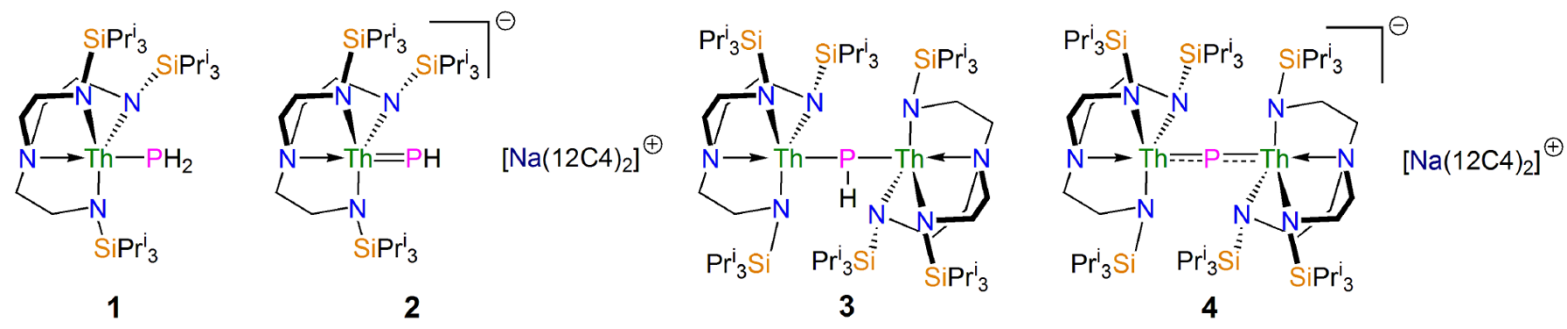
Complexes **1**–**4**.

Germane to the aforementioned arguments, we previously reported a family of triamidoamine–Th complexes with a range of Th–P linkages.^52a^ Specifically, we reported the phosphanide [Th(PH_2_)(Tren^TIPS^)] (**1**, Tren^TIPS^ = {N(CH_2_CH_2_NSiPr^i^_3_)_3_}^3–^), the phosphinidene [Th(PH)(Tren^TIPS^)][Na(12C4)_2_] (**2**, 12C4 = 12-crown-4 ether), the phosphinidiide [{Th(Tren^TIPS^)}_2_(μ-PH)] (**3**), and the bridging phosphido [{Th(Tren^TIPS^)}_2_(μ-P)][Na(12C4)_2_] (**4**), Figure 1. Given our prior work probing the covalency of a terminal uranium(VI)-nitride linkage,^37^ and introducing ^29^Si NMR spectroscopy to probing the covalency of lanthanide and s-block silanides,^46^ we identified that **1**–**4** would be an ideal family of molecules with which to probe and benchmark the covalency of the Th–P linkages using ^31^P NMR spectroscopy.

Here, we introduce ^31^P NMR spectroscopy to investigate molecular f-element covalency by the study of the Th–P linkages of **1**–**4** using solution and SS-Magic Angle Spinning (SS-MAS) NMR techniques. Through this combined approach, underpinned by quantum chemical techniques, we have been able to relate the isotropic chemical shift (δ_iso_) to its constituent individual components and then relate it to the shielding tensors and hence the Th–P covalency. A combined Molecular Orbital (MO), Natural Bond Orbital (NBO), and Natural Localized Molecular Orbital (NLMO) approach has enabled elucidation of the underlying factors that determine the observed trends, including a remarkably large chemical shift anisotropy (CSA) for the bridging phosphido center in **4**. This study has then permitted us to correlate the Th–P δ_iso_ values to Mayer Bond Orders (MBOs), but we find that this is very dependent on the ancillary ligands. This highlights an important difference between N- and P-ligands, which is that the former tend to be the dominant component of the ligand field with ancillary ligands in a secondary role, whereas trends with the softer P-ligands are clearly much more ancillary ligand dependent.

## Results and Discussion

### Experimental Solution ^31^P NMR Data

Compounds **1**–**4** were prepared as described previously.^52a^ The purity and stability of **1**–**4** were checked and confirmed by examination of their ^1^H, ^13^C{^1^H}, ^29^Si{^1^H}, ^31^P, and ^31^P{^1^H} NMR spectra recorded in C_6_D_6_ or D_8_-THF. As part of that process, regarding the ^31^P NMR data while the δ_iso_ values for **1** (−144.1 ppm) and **4** (553.5 ppm) were confirmed,^52^ it was discovered that the originally reported δ_iso_ values for **2** (150.8 ppm)^52a^ and **3** (24.5 ppm)^52a^ are in fact 198.8 and 145.7 ppm, respectively.^52b^ The original errors appear to be due to an isolated episode of referencing errors, but the δ_iso_ values for **1**–**4** of −144.1, 198.8, 145.7, and 553.5 ppm, Table 1 and Figures S1–S4, are now definitively confirmed by the solid-state magic angle spinning (SS-MAS) ^31^P NMR data (see below).

**Table 1:** Experimental and Computed NMR Properties for **1**–**4** and **1′**–**4′**^a^

**Property**	**1**	**2**	**3**	**4**	**1′**	**2′**	**3′**	**4′**
δ_iso(sol)_^b^	–144.1^d^	198.8	145.7	553.5	–	–	–	–
δ_iso(ss)_^c^	–138.9^d^	211.8	151.8	554.8	–	–	–	–
δ_iso(calc)_	–146.8	216.6	147.8	519.2	–149.9	209.0	167.5	551.1
δ_11(ss)_^e^	–83.7	539.9	441.3	1047.2	–	–	–	–
δ_22(ss)_^e^	–102.3	368.6	290.8	972.0	–	–	–	–
δ_33(ss)_^e^	–233.1	–274.6	–279.1	–357.2	–	–	–	–
Ω_(ss)_^e^	149.4	813.6	720.5	1404.4	–	–	–	–
κ_(ss)_^e^	0.75	0.58	0.58	0.89	–	–	–	–
δ_11(calc)_	–92.9	523.2	584.6	996.4	–90.6	511.8	589.4	1033.6
δ_22(calc)_	–142.5	404.4	130.7	959.3	–148.5	399.3	160.4	1011.3
δ_33(calc)_	–205.1	–277.7	–271.9	–398.1	–210.5	–284.1	–247.3	–391.8
Ω_(calc)_	112.2	800.9	856.5	1394.5	119.9	795.9	836.7	1425.4
κ_(calc)_	0.11	0.70	0.06	0.95	0.04	0.72	0.03	0.97
σ_iso(calc)_	491.3	127.9	196.7	–174.7	494.4	135.5	177.0	–206.6
σ^d^_(calc)_	963.2	968.7	968.5	973.9	963.4	969.2	969.5	974.9
σ^p^_(calc)_	–452.0	–823.7	–663.7	–993.9	–454.3	–817.9	–670.0	–1008.9
σ^so^_(calc)_	–20.0	–17.1	–108	–154.1	–14.7	–15.8	–122.5	–172.5

a Calculations at the B3LYP-HF50 TZ2P all-electron ZORA spin–orbit (SOR) level in a benzene solvent continuum and corrected for a σ_iso(calc)_ of 584.5 ppm for PH_3_ noting experimental δ_iso_ values of −266.1 and −240 ppm in the gas-phase and solution (C_6_D_6_); all δ and σ values are in ppm.

b In C_6_D_6_.

c SS-MAS conditions.

d Average of two molecules.

e Derived from simulation of the respective experimental SS-MAS ^31^P NMR spectrum.

### Experimental SS-MAS ^31^P NMR Data

**Figure 2:**
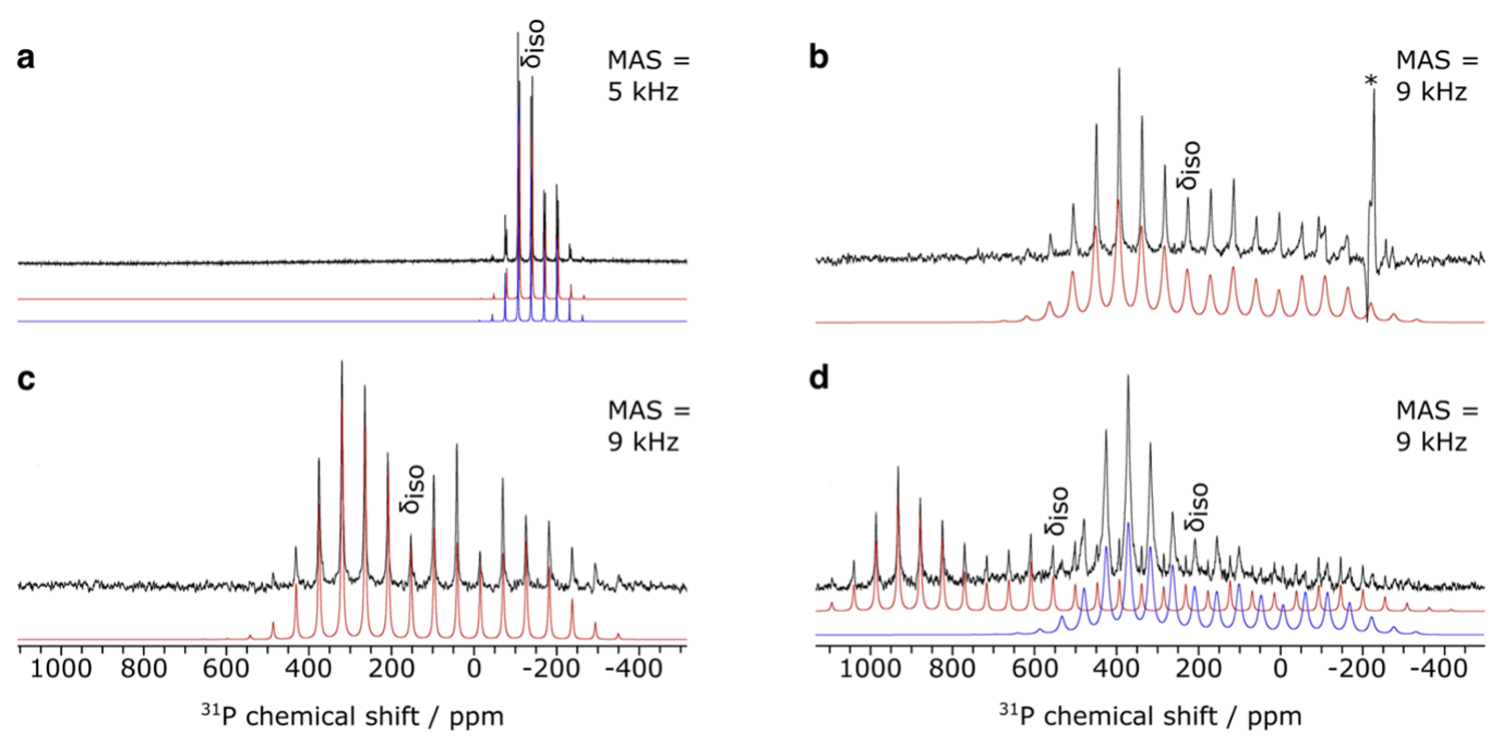
SS-MAS ^31^P NMR spectra for **1**–**4**. (a) complex **1**, MAS frequency = 5 kHz; (b) complex **2**, MAS frequency = 9 kHz; (c) complex **3**, MAS frequency = 9 kHz; (d) complex **4**, MAS frequency = 9 kHz. Simulations (red and blue) are provided for the experimental data (black). The position of the isotropic peak is indicated (δ_iso_) for each complex and a degradation product of **2** is highlighted by an asterisk (*). Note that there are two molecules in the asymmetric unit of **1** and that **4** has partially degraded into **2** during data acquisition.

To gain a fuller experimental characterization of the ^31^P chemical shift tensors in **1**–**4**, ^31^P NMR spectra of powdered samples of **1**–**4** were collected with MAS frequencies of 5 kHz (**1**) and 9 kHz (**2**–**4**), Figure 2 and Table 1, which taken together with a natural abundance of 100% *I* = 1/2 for the ^31^P nucleus provided satisfactory signal-to-noise ratios and hence reliable spectral simulations. The ^31^P SS NMR δ_iso_ values for **1**–**4** were determined to be −140.6/–137.1 (av. −138.9), 211.8, 151.8, and 554.8 ppm, respectively (with δ_iso_ values confirmed by also conducting the measurements with spinning frequencies of 2 (**1**) and 5 (**2**–**4**) kHz, Figure S5), which in each case are in good agreement (maximum Δ_sol/ss_ = 13 ppm) with the respective solution δ_iso_ values given that precise ^31^P δ_iso_ values are exquisitely sensitive to the chemical environment. Note for **1** the data quality permitted the extraction of two δ_iso_ values in the spectral simulation, which results from there being two independent molecules of **1** in the crystallographic asymmetric unit that exhibit slightly different Th–P distances of 2.982(2) and 3.003(2) Å.^52a^ Since the Th–P bond length and δ_iso_ values are rather similar we use the average SS-MAS δ_iso_ for **1**.

For **1**, the average chemical shift tensor values δ_11_, δ_22_, and δ_33_ of −83.7, −102.3, and −233.1 ppm are fairly similar to one another, producing a relatively small chemical shift tensor span (Ω, δ_11_–δ_33_) of 149.4 ppm, Figure 2a. The skew value (κ, [3(δ_22_–δ_iso_)]/δ_11_–δ_33_) of 0.75 is between the values expected for axial (1.00) and terminal double bond (0.5),^23^ reflecting the Th–PH_2_ single bond linkage. In contrast, reflecting the presence of the Th=PH double bond, the corresponding δ_11_, δ_22_, δ_33_, Ω, and κ values for **2** are 539.0, 368.6, −274.6, 813.6 ppm, and 0.58, Figure 2b, and this follows a similar pattern to that found for [Ti(=PC_6_H_2_-2,4,6-Pr^i^_3_)(Me){(DippNCBu^t^)_2_CH}] (Dipp = 2,6-diisopropylphenyl),^73^ which has corresponding values of 630, 430, −400, 1030 ppm, and 0.61. The δ_11_, δ_22_, δ_33_, Ω, and κ values of 441.3, 290.8, −279.1, 720.5 ppm, and 0.58 for **3**, Figure 2c, are at first glance surprisingly similar to those for **2**; however given the rather polar nature of Th–P bonds, this reflects the close relationship between a phosphinidene and phosphinidiide. On moving to **4** the presence of the bridging phosphide becomes apparent, with δ_11_, δ_22_, δ_33_, Ω, and κ values of 1047.2, 972.0, – 357.2, 1404.4 ppm, and 0.89, Figure 2d. The presence of two large, positive chemical shift tensors and one negative one along with a κ value approaching the linear ideal underscores the axial nature of the Th=P=Th linkage. Notably, the Ω value for **4** is relatively large,^74^ and while it is exceeded by terminal phosphido complexes such as [W(≡P){(Me_3_SiNCH_2_CH_2_)_3_N}] (Ω = 2008 ppm),^75^ [Mo(≡P){N(3,5-Me_2_C_6_H_3_)(Bu^t^)}_3_] (Ω = 2308 ppm),^76^ [Mo(≡P){N(Ph)(Bu^t^)}_3_] (Ω = 2311 ppm), and [Mo(≡P){(Me_3_SiNCH_2_CH_2_)_3_N}] (Ω = 2392 ppm),^75^ it is, as far as we are aware, the largest recorded Ω value for a bridging phosphido ligand. Indeed, the ^31^P SS NMR data for **1**–**4** demonstrate a CSA Ω ordering of (μ-P)^3–^ > (=PH)^2–^ > (μ-PH)^2–^ > (−PH_2_)^1–^.

We note that the δ_33_ values for **1**–**4** span the relatively narrow range of −233.1 to −357.2 ppm, though this is not surprising since the P-ligands have similar binding geometries. However, the larger variance for the δ_11_ and δ_22_ values for the different ligands implies that there are vacant orbitals of appropriate symmetry to couple with filled orbitals,^77^ and that the energy difference between these orbitals likely follows the trend **1** > **3** > **2** > **4**. The greater interaction between these orbitals for **4** induces a large NMR deshielding and thus large δ_11_ (and δ_22_). This is therefore investigated further with computational modeling (see below).

### Computational Benchmarking of the ^31^P NMR Spectroscopic Properties of **1**–**4** and **1′**–**4′**

With the solution and SS-MAS ^31^P NMR data on **1**–**4** confirmed we turned to the computational assessment of those data using Density Functional Theory (DFT, see Computational Details section for further details). Scalar Relativistic (SR) and two-component Spin–Orbit Relativistic (SOR) single point energy calculations for **1**–**4** were then acquired examining a range of functionals (BP86, SAOP, PBE0, and B3LYP, the latter two with a range of Hartree–Fock mixing), Tables S1–S4. Those data were then used to compute the SR and SOR ^31^P NMR δ_iso_ values in benzene solvent continuums, converting calculated σ to δ values using the calculated σ_iso_ values for PH_3_ in a benzene continuum.

**Figure 3:**
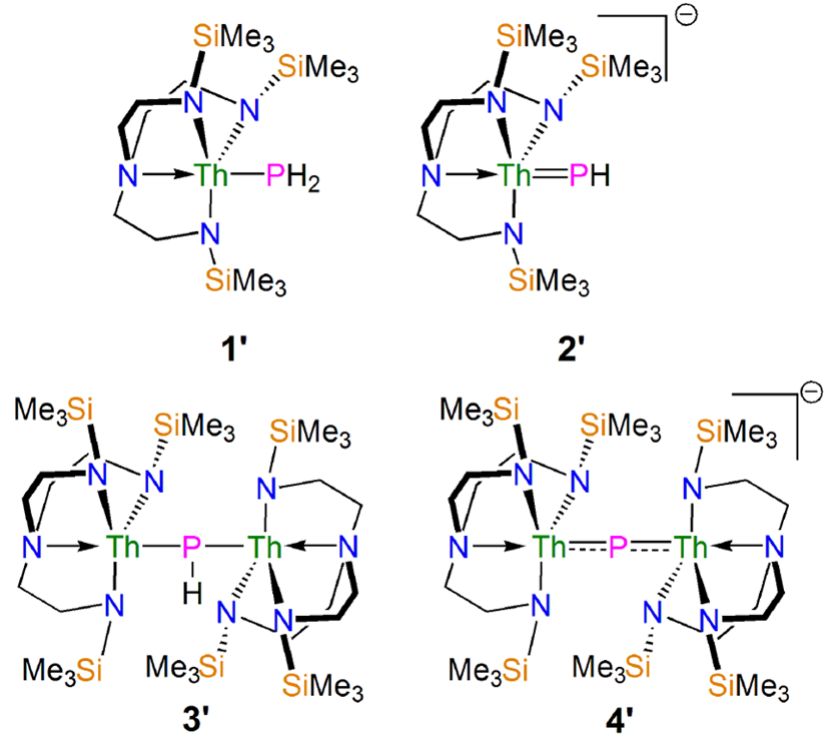
Truncated model complexes **1′**–**4′** used for the MO, NBO, and NLMO analyses.

It was determined that the B3LYP-HF50 SOR best reproduces the experimental ^31^P δ_iso_ data for **1**–**4**, Table 1. However, during the course of this study, it became apparent that memory limit issues for NBO and NLMO calculations prevented the NBO and NLMO data for **3** and **4** being computed. Since in that scenario changing the functional or basis sets was not appropriate due to large changes in computed δ_iso_ values, we truncated the SiPr^i^_3_ substituents of the Tren^TIPS^ ligand in **1**–**4** to SiMe_3_ (Tren^TMS^), referred to as **1′**–**4′**, Figure 3; the Pr^i^ Me groups were replaced by H atoms whose positions were optimized while keeping all the heavy atom positions fixed. The outcome of the truncation is that the computed SOR properties of **1′** and **2′** remain essentially unchanged on moving from **1** and **2**, Tables S1 and S2. However, we note that the computed data for **3**/**3′** and **4**/**4′** shift, Tables S3 and S4. For **3** good agreement was found (computed to within 4 ppm of experiment) but the computed ^31^P chemical shift of **4** was computed to be ∼− 34 ppm relative to experiment; however, **4′** is computed to within 3 ppm but then **3′** is computed to be ∼+20 ppm from experiment. Considering the computational constraints and that the computed ^31^P δ_iso_ changes between **1**–**4** and **1′**–**4′** are trivial when placed on the full δ_iso_ range of ^31^P chemical shifts, it was concluded that the truncated Tren^TMS^ data of **1′**–**4′** are most representative and practical to use, and so they are used in the analysis that follows. It should be noted, however, that given the sensitivity of ^31^P δ_iso_ values to a wide range of parameters, it is remarkable how good the agreement of the calculated to experimental δ_11_, δ_22_, δ_33_, Ω, and κ data are in terms of the breakdown for a given molecule, the consistently good agreement overall across all of **1′**–**4′**, and the fairly consistent parameters computed for **1**–**4** vs **1′**–**4′**.

For **1′** the SR calculation returns a ^31^P δ_iso_ value of −176.7 ppm, and this shifts to −149.9 ppm in the SOR calculation, the latter of which is in good agreement with the solution and SS δ_iso_ values of −144.1 and −138.9 (av) ppm, respectively. The computed δ_iso_ value for **1′** decomposes to δ_11_, δ_22_, δ_33_, Ω, and κ values of −90.6, −148.5, −210.5, 119.9 ppm, and 0.04, in fair agreement with the experimental SS values of −83.7, −102.3, −233.1, 149.4 ppm, and 0.75, respectively. However, we note a significant divergence of the experimental and calculated δ_22_ and thus κ values for **1′** (Δκ_exp-calc_ = 0.71), which likely reflects dynamic rotation of the PH_2_ group experimentally that is not captured by calculations on a static model. In passing, we note that a similar ^15^N NMR modeling averaging effect was found for the −NH_2_ unit of [Th(NH_2_){N(SiMe_3_)_2_}_3_].^36^

The SR calculation for **2′** gives a ^31^P δ_iso_ value of 191.3 ppm, and for the SOR calculation this moves to 209.0 ppm, which is between the solution and SS δ_iso_ values of 198.8 and 211.8 ppm, respectively. The ∼370 ppm shift to higher frequency for **2′** compared to **1′** can be seen to derive from δ_11_, δ_22_, and δ_33_ values of 511.8, 399.3, and −284.1 ppm, reflecting the Th=P double bond, resulting in a larger Ω value of 795.9 ppm and a κ value of 0.72, close to that expected for a terminal metal–ligand double bond. Overall, these values are in good agreement with those derived from the SS NMR data (539.0, 368.6, −274.6, 813.6 ppm, and 0.58, respectively).

The SR and SOR calculations for **3′** afford δ_iso_ values of 40.7 and 167.5 ppm, respectively, the latter of which is in reasonable agreement with the experimental solution and SS δ_iso_ values of 145.7 and 151.8 ppm, respectively. However, the modeling of **3′** is the poorest of **1′**–**4′**, and this can be understood when inspecting the computed δ_11_, δ_22_, δ_33_, Ω, and κ values of 589.4, 160.4, −247.3, 836.7 ppm and 0.03 (*cf*. experimental values of 441.3, 290.8, −279.1, 720.5 ppm, and 0.58); while the δ_33_ value is reproduced well, both the δ_11_ and δ_22_ values are significantly over- and underestimated, which leads to the variation in the Ω value and the large discrepancy in the κ value. The same issue was found with the computed data for **3** as well as **3′** suggesting that this is not a consequence of the Tren truncation, and given the finding for **1′** and [Th(NH_2_){N(SiMe_3_)_2_}_3_]^36^ it is likely that experimentally there is dynamic rotation of the P–H group that does not render δ_11_ and δ_22_ completely equivalent—due to the threefold (or pseudo sixfold) rotation axis created by the Tren^TIPS^ ligands—but which has the effect of reducing the difference between δ_11_ and δ_22_ experimentally whereas this effect is not captured by the calculations which represents the extreme static situation where the asymmetry of δ_11_ and δ_22_ would be exacerbated.

Lastly, for **4′** the SR and SOR calculations return δ_iso_ values of 387.3 and 551.1 ppm, respectively, where the latter is in very good agreement with the experimental solution and SS δ_iso_ values of 553.5 and 554.8 ppm, respectively. The axial Th=P=Th bonding environment in **4′** is reflected in the computed δ_11_, δ_22_, δ_33_, Ω, and κ values of 1033.6, 1011.3, −391.8, 1425.4 ppm, and 0.97, which is in good agreement with the experimental SS NMR data of 1047.2, 972.0, −357.2, 1404.4 ppm, and 0.89, respectively.

### Molecular Orbital, Natural Bond Order, and Natural Localized Molecular Orbital Benchmarking of **1′**–**4′**

Having established that the B3LYP-HF50 SOR calculations satisfactorily reproduce the key δ_iso_, δ_11_, δ_22_, δ_33_, Ω, and κ values for **1′**–**4′**, and hence **1**–**4**, we reanalyzed their electronic structures at that level of theory, Table 2; previously the BP86 functional at the SR level was used to elucidate the electronic structures of **1**–**4**, but we find that generally there is remarkably good agreement between the BP86 and B3LYP-HF50 models, and where there are significant differences this can be traced back to differences between NBO5 and NBO6, specifically cut-offs not returning components in the former that are included in the latter. In terms of MOs that are relevant to the following NMR discussion, we find (i) for **1′** a Th–P σ-bond as the HOMO along with the P-lone pair as HOMO–4; (ii) for **2′** Th=P π- (HOMO) and σ-bonds (HOMO–1) along with a P-lone pair (HOMO–5) that is the back-lobe of the P–H σ-bond; (iii) for **3′** Th–P dative π- (HOMO) and covalent σ-bonds (HOMO–1) along with a P-lone pair that is the back-lobe of the P–H σ-bond; (iv) for **4′** Th=P two π- (HOMO and HOMO–1) and a σ-bond (HOMO–2). In all cases the Th–P bonds involve variable 3s and 3p contributions from P and predominantly 5f and 6d contributions from Th along with modest Th 7s contributions.

**Table 2:** Computed Bond Orders, Charges, and NBO Data for **1′**–**4′**^a^

	**Mayer BI**	**Atomic charges**		**NBO Th–P σ-component**				**NBO Th–P π-component**			
**Entry**	**Th–P**	**Th**	**P**	**%Th**	**%P**	**Th 7s/7p/6d/5f**	**P** **32/3p**	**%Th**	**%P**	**Th72/7p/6d/5f**	**P 3s/3p**
**1′**	0.63	2.12	–0.81	12	88	26/1/54/19	38/62	–	–	–	–
**2′**	1.25	1.94	–1.22	16	84	14/0/64/22	41/59	17	83	0/0/75/25	0/100
**3′**	0.71	2.05	–1.60	11	89	21/0/57/22	44/56	8	92	0/0/71/29	0/100
				11	89	21/0/57/22	44/56	8	92	0/0/71/29	0/100
**4′**	1.27	1.89	–1.25	14	86	18/0/62/20	50/50	16^b^	84	0/0/70/30	0/100
				14	86	18/0/62/20	50/50	16^b^	84	0/0/70/30	0/100

a Calculations at the B3LYP-HF50 TZ2P all-electron ZORA spin–orbit (SOR) level in a benzene solvent continuum.

b 3-Center bond, the 16% is made up of 2 × 8% contributions from 2 × Th atoms.

The Th–P MBOs for **1′**–**4′** are computed to be 0.63, 1.25, 0.71, and 1.27. This reflects the polar covalent nature of these Th–P bonds, and that (i) the formal Th–P bond order doubles from **1′** to **2′**; (ii) in phosphinidiide **3′** the Th–P bonds are formally single but supplemented with dative π-bonding; (iii) that **4′** has formally double (Lewis) and pseudotriple (MO) Th–P bonds that are bridging rather than terminal. The Th and P MDC_q_ charges for **1′**–**4′** are 2.12/–0.81, 1.94/–1.22, 2.05/–1.60, and 1.89/–1.25. The variation in Th charge is reasonably small, but we note that the Th ion charges in **2′** and **4′** are lower than those in **1′** and **3′** reflecting the stronger donor power of terminal HP^2–^ and bridging P^3–^ compared to H_2_P^1–^ and bridging HP^2–^ and also that bridging P^3–^ is a stronger donor than terminal HP^2–^. The P charges also reflect that pattern of charge donation from P to Th. Overall, while there are some variations of the B3LYP data compared to the previous BP86 the agreement and trends remain good overall.

The MOs of **1′**–**4′** that describe the Th–P interactions are clear-cut, but the MOs often contain minor intrusions of orbital coefficients from other atoms, most notably the N-donors, so to provide a more localized and chemically intuitive model, we turned to NBO analyses. In general, though there are naturally variations between the previously reported BP86 and B3LYP-HF50 analysis presented here, the two sets of NBO data provide a consistent bonding picture in surprisingly good agreement with one another. In particular, the previously reported significant contributions of 7s character to the Th–P bonding, where Th bonding is generally thought to be dominated by 5f/6d character,^8^ is also consistently returned by the B3LYP NBO calculations as was the case for the prior BP86 calculations. In **1′** the Th–P bond consists of 12% and 88% Th and P character, respectively. The Th component is 26/1/54/19% 7s/7p/6d/5f character, and the P part is 38/62% 3s/3p. The Th=P double bond in **2′** is reflected in slightly larger Th contributions to the bonding, where the Th=P σ- and π-bonds are 16/84 and 17/83% Th/P character. The Th 7s/7p/6d/5f component of the Th=P σ-bond is 14/0/64/22% whereas for the π-bond it is 0/0/75/25%. The P 3s/3p contributions to the σ- and π-bonds are 41/59% and 0/100%, respectively. That the dianion charge of the (HP)^2–^ unit in **3′** is spread over two Th atoms but only one Th atom in **2′** is reflected by the NBO data of **3′**. Specifically, for **3′** two Th–P bonds with Th/P character of 11/89% are found, and then the two dative Th–P π-bonds are each 8/92% Th/P character. In the Th–P σ-bonds the Th character is 21/0/57/22% 7s/7p/6d/5f character and the P component is 44/56% 3s/3p character. The Th character in the Th–P bonds is 0/0/71/29% 7s/7p/6d/5f, and the P is 0/100% 3s/3p. For **4′** the Th=P σ-bonds and two π-bonds are 14/86 and 16/84% Th/P character, respectively. Note, the two π-bonds are represented as 3c2e bonds in the NBO analysis, and hence the Th/P character is a composite of 8/8/84% Th/Th/P character. Once again significant Th 7s contributions are found in the σ- but not π-bonds, with Th 7s/7p/6d/5f contributions of 18/0/62/20 and 0/0/70/30%, respectively. For the P contributions, these are 3s/3p 50/50 and 0/100%, respectively.

**Figure 4:**
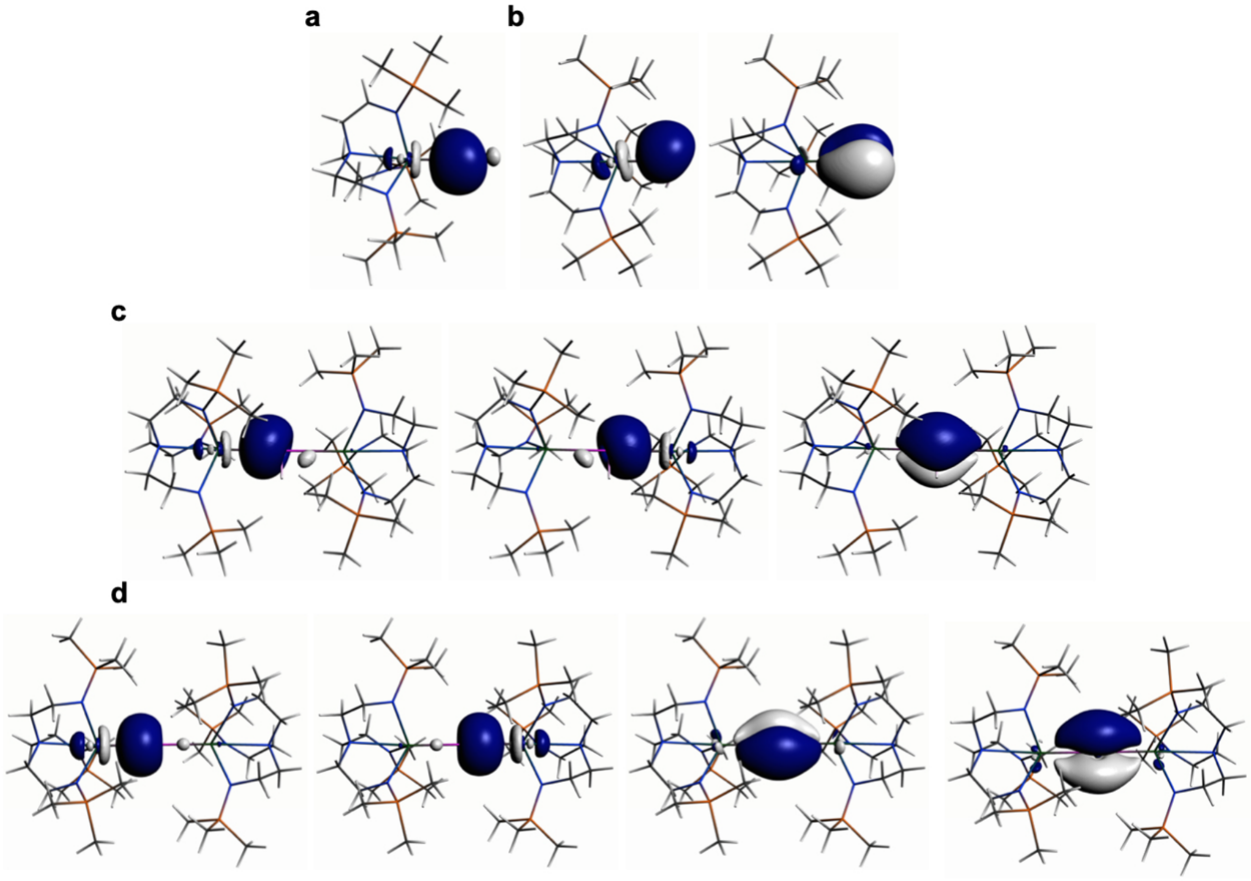
NLMO representations of: (a) the Th–P σ-bond in **1′**; (b) the Th–P σ- and π-bonds in **2′**; (c) the Th–P 2 × σ- and 1 × π-bond in **3′**; (d) the 2 × σ- and 2 × π-bonds in **4′**.

In all instances, the Th–P bonding interactions described by the NBO calculations for **1′**–**4′** exhibit electron occupancies of ≥1.82 electrons per orbital, and hence, the NBO orbitals are localized and thus the breakdowns are representative, especially as they are computed with a functional that reproduces the NMR parameters well. In order to further validate the NBO calculations and for the NLMO-NMR analysis below, we also performed the NLMO calculations, Figure 4 and Table S5. We find only relatively modest changes in the Th and P contributions to Th–P bonding between NBO and NLMO methods, giving confidence that this experimentally benchmarked analysis represents a quantification of the Th–P bonds in **1′**–**4′** and hence **1**–**4**.

### Computational Shielding Analysis of **1′**–**4′**

In order to develop the analysis of the nature of the Th–P bonds in **1**–**4**, it is necessary to translate the chemical shift tensor δ_iso_, δ_11_, δ_22_, δ_33_, Ω, and κ parameters discussion to shielding, since chemical shifts derive from the nuclear shielding being adjusted with respect to the shielding and actual chemical shift of a reference (for this study with respect to PH_3_, itself referenced to the IUPAC standard of 85% H_3_PO_4_). Thus, the isotropic chemical shift δ_iso_ derives from the isotropic shielding (σ_iso_), which in turn is composed of diamagnetic (σ^d^), paramagnetic (σ^p^), and spin–orbit (σ^so^) shielding components. It naturally follows that δ_11_, δ_22_, and δ_33_ derive from σ_11_, σ_22_, and σ_33_ parameters, Table 1.

Ramsey related σ_iso_ to σ^d^ and σ^p^ (eq 1).^78−80^ This does not map directly onto hybrid DFT (B3LYP); however when adjusted for σ^so^, eq 2, this establishes a framework through which to rationalize NMR shielding calculations.^81^
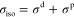
1
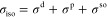
2

The σ^d^ range for **1′**–**4′** is 963.4–974.9 ppm, which is a relatively small variance. Even for the rather sensitive ^31^P nucleus this is to be expected because σ^d^ relates to tightly bound core electron density that responds little to perturbations in the valence region.^81^ Considering the large σ range (and hence δ range) of ^31^P NMR spectroscopy, a variance of 11.5 ppm can be considered to be negligible in terms of this discussion.

The σ^so^ values for **1′**–**4′** show considerably more variance than the σ^d^ values. For **1′**–**4′** the σ^so^ values are −14.7, – 15.8, – 122.5, and −172.5 ppm. Recalling that the σ_iso_ values for **1′**–**4′** are 494.4, 135.5, 177.0, and −206.5 ppm it is evident, given that σ^d^ shows little variance, that (i) σ^so^ is very similar for **1′** and **2′** yet their σ_iso_ values are different by ∼359 ppm; (ii) σ^so^ varies by ∼107 ppm from **2′** to **3′** but their σ_iso_ values are only ∼42 ppm apart; (iii) σ^so^ from **3′** to **4′** varies by 50 ppm but the σ_iso_ shifts by ∼384 ppm. Thus, while the σ^so^ values are significant and certainly cannot be isolated from the analysis they clearly are not the decisive factor in determining the overall σ_iso_ values. Indeed, we note that the σ^so^ values are ∼2–15% of the σ^p^+σ^so^ values (*vide infra*). The similar σ^so^ values for **1′** and **2′** that significantly increase for **3′** then **4′** suggests increasingly efficient transfer of spin–orbit character to the NMR nucleus, P, from the Th ions. The NBO analysis revealed not only substantial Th s character in the Th–P bonds of **1′**–**4′** but also large s contributions from the P centers, implying an efficient Fermi-contact pathway to transmit spin–orbit effects from Th to P by spin–dipole interactions. Spin–orbit effects are larger for 5f- than 6d-orbitals, and although the NBO analysis shows that the Th bonding is dominated by 6d contributions (51–78%) in each case, there are significant (19–30%) 5f-contributions. We note that the 5f-orbital contributions to the Th–P bonds are 19% and 22% for **1′** and **2′**, then increase to 29% for **3′**, and then 30% for **4′** which is also accompanied by the number and strength of Th–P interactions (i.e., single, double, single+dative, pseudotriple) increasing on going from **1′** to **4′**, which is in-line with the increasing σ^so^ for **1′** to **4′**.
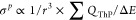
3

It is then logical to deduce that the σ^p^ component is likely the decisive factor for rationalizing the bonding in **1**–**4**. The σ^p^ parameter can be represented in reduced form as per eq 3,^37,48,78−81^ where *r* is the radial expansion of the shielding electrons from the NMR nucleus (here ^31^P), *Q*_ThP_ is the sum of the charge density and bond order matrix elements over the relevant atoms (here Th and P), and Δ*E* is the energy separation between the ground (filled) and excited (vacant) states in question.^22,78−80^

The σ^p^ term is inversely proportional to the occupied-virtual orbital[s] energy gap[s] that become magnetically coupled in the presence of an externally applied magnetic field, so unusually small or large energy gaps would produce enhanced or diminished magnetic coupling, respectively, and hence skew the analysis. Taking HOMO–LUMO gaps as indicative measures, the HOMO–LUMO gaps of **1′**–**4′** are computed to be 4.2–5.9 eV, which suggests that any disproportionate effects on Δ*E* and hence σ^p^ for **1′**–**4′** can be discounted.

Field-induced magnetic mixing of the ground state with low-lying, thermally inaccessible paramagnetic states, i.e. temperature independent paramagnetism (TIP), would result in ΣQ_ThP_ disproportionately distorting the values of σ^p^. In order to exclude this, we examined the variable temperature magnetism of **1**–**4** using SQUID magnetometry over the temperature range 1.8–290 K (Figures S6 and S7). Complex **4** persistently returned a negative susceptibility, that is, a diamagnetic response. Complexes **1**–**3** exhibit very weak TIP, with values of 1.1166 × 10^–4^, 3.3129 × 10^–4^, and 2.4409 × 10^–5^ cm^3^ mol^–1^ K, similar to some U(VI) complexes,^29,37^ with χ*T* vs *T* linear regression *R*^2^ values of 0.9970, 0.9995, and 0.9985, respectively, over the 85–290 K region. This suggests that any TIP effects are either absent (**4**) or negligible (**1**–**3**) and so the ΣQ_ThP_ term does not introduce a disproportionate effect on σ^p^ for **1**–**4**.

Turning to the *r*^3^ term, eq 3 shows that σ^p^ is inversely proportional to the cube of the radial expansion of the shielding electrons from the NMR nucleus. This is because as the NMR nucleus (here ^31^P) has its charge withdrawn to the coordinated nucleus (here Th) this renders the NMR nucleus more electron deficient, and deshielded, and hence its valence orbitals contract reducing *r* and hence the 1/*r*^3^ term becomes larger. Thus, the larger the Th–P bond order the larger 1/*r*^3^ will be and hence the larger σ^p^ will be.^48^ Taking all the above together, that the σ^p^ values for **1′**–**4′** are computed to be −454.3, −817.9, −670.0, and −1008.9 ppm (and these dominant terms contribute to generating σ_iso_ values that in turn produce calculated δ_iso_ values that match well to experimentally determined δ_iso_ values) is significant because it reflects the Th–P bonding environments of **1**–**4** of Th–P single bond, Th=P double bond, Th–P single bonds supplemented by dative π-bonds, and then Th–P pseudotriple (or in Lewis nomenclature Th=P double) bonds. Thus, the σ^p^ values of **1′**–**4′** are in-line with the bonding motifs and MBOs of **1**–**4** in terms of the formal bond multiplicities of each respective Th–P linkage.

**Figure 5:**
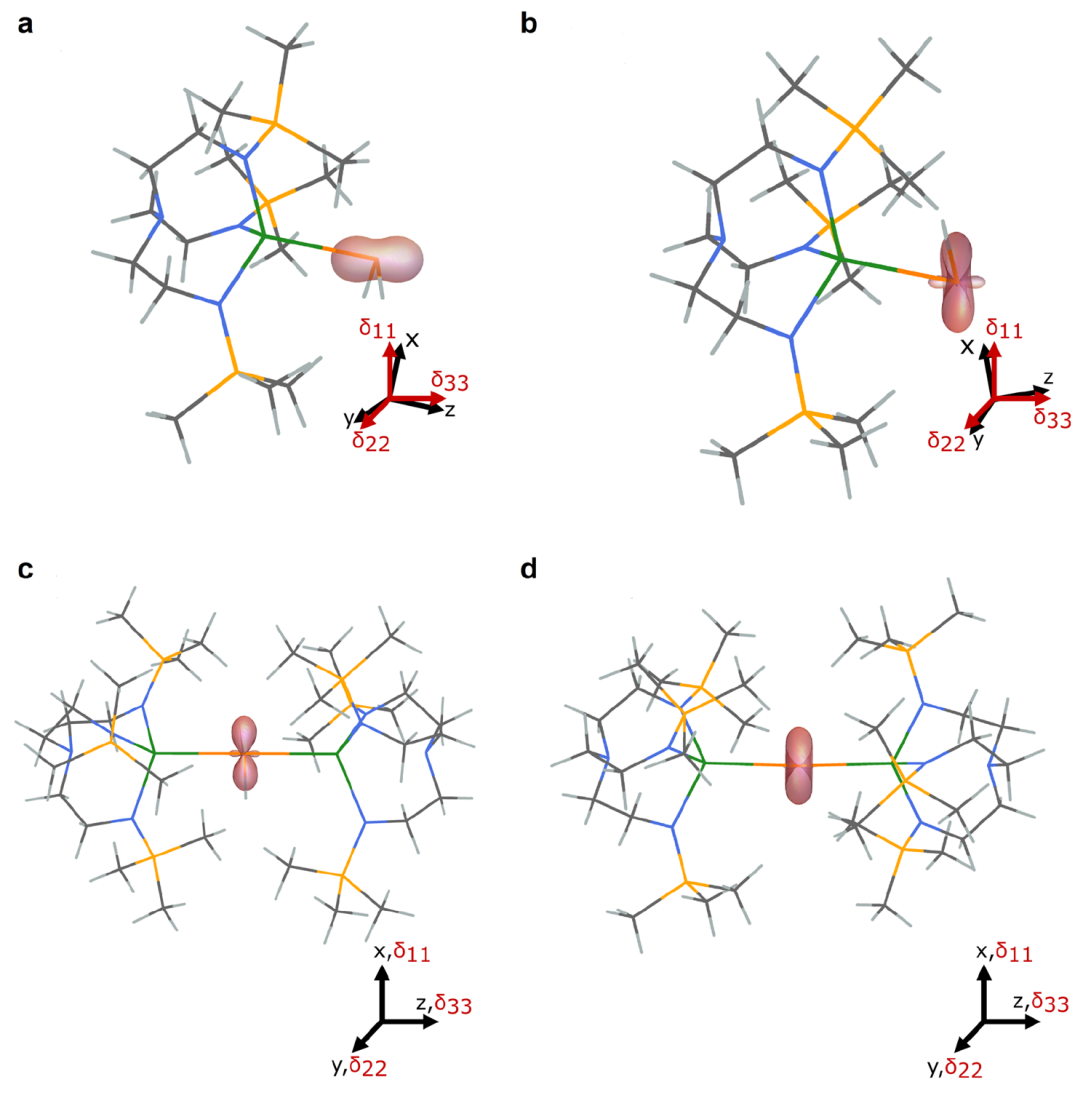
Plots of the δ_11_, δ_22_, and δ_33_^31^P tensor components for **1′**–**4′**. (a) complex **1′**; (b) complex **2′**; (c) complex **3′**; (d) complex **4′**. The shielding surface is represented using the ovaloid convention where the distance from the P atom to a point on the surface is proportional to the chemical shift when the magnetic field is aligned along that direction in space. The shading of the surface denotes the sign of the shift where orange is positive and light orange is negative. The principal axes (*x*, *y*, *z*) are provided, along with the directions of the tensor components (δ_11_, δ_22_, δ_33_); note that for **3** and **4**, these axes align fully.

Shielding effects can be rationalized by using the rotated orbital model,^77,82−86^ which describes the action of the angular momentum operator (*L*) on magnetically coupled occupied and virtual orbitals; this is intuitively visualized as a 90° rotation of an idealized occupied orbital to mix with a vacant orbital. The computed orientations of the ^31^P σ_11_, σ_22_, and σ_33_ shielding tensor principal components are shown in Figure 5 (after conversion to δ_11_, δ_22_, and δ_33_; see Figure S8 for depictions in another set of orientations). As shown, these components align or almost align along the principal axes (*x*, *y*, and *z*), and for **2′** and **3′** one of the principal axes is almost parallel to the P–H bonds in each case. Thus, for **1′**–**4′**, in each case the occupied Th–P σ-orbital magnetically couples to virtual π* Th–P orbitals via the action of the *L*_*x*_ or *L*_*y*_ angular momentum operators, which are perpendicular to the Th–P bond that, by convention, lies along the *z*-direction of the principal axes (Figure 5). Consequently, this induces a paramagnetic term (a principal component of σ^p^), perpendicular to the Th–P bond (*z*), along *x* or *y*. Since the P–H bonds are essentially perpendicular to the Th–P bonds in **1′**–**3′**, magnetic coupling of the Th–P occupied σ-orbital with virtual σ* P–H orbitals can also contribute to deshielding perpendicular to the Th–P–H plane. Likewise, occupied Th–P π-orbitals can mix with virtual σ* Th–P and P–H orbitals via the action of *L*_*x*_ and *L*_*z*_, respectively.

### Molecular Orbital Shielding Analysis of **1′**–**4′**

**Figure 6:**
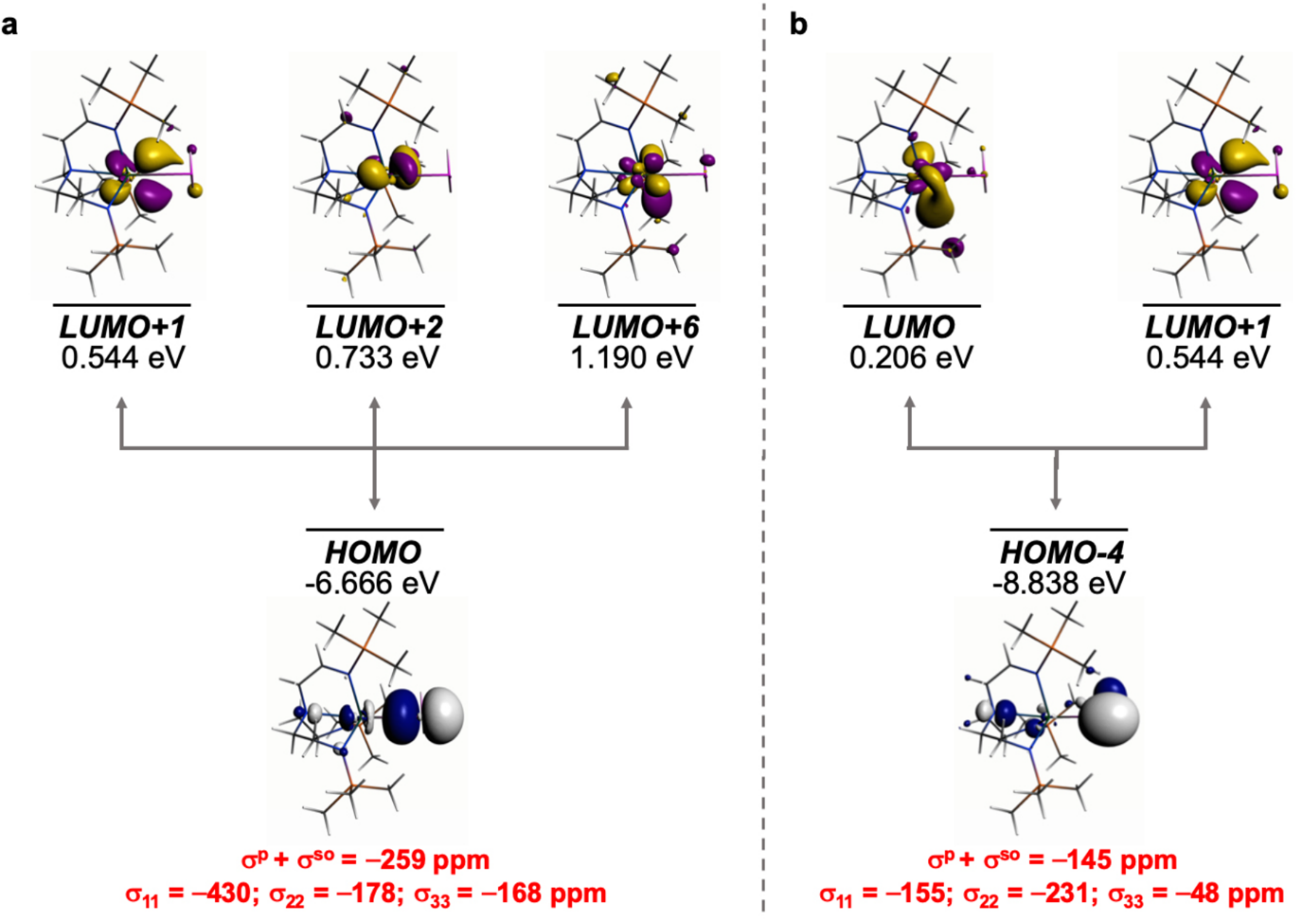
Dominant occupied and virtual MOs that contribute to the σ_iso_ and hence δ_iso_ of **1′**. (a) Magnetic coupling of the Th–P σ-bond with Th 5f/6d hybrids of pseudo π* character. (b) Magnetic coupling of the P-lone pair with Th 5f/6d hybrids. The isotropic shielding values for the individual bonding components are given in red, and each is broken down into its principal component contributions.

**Figure 7:**
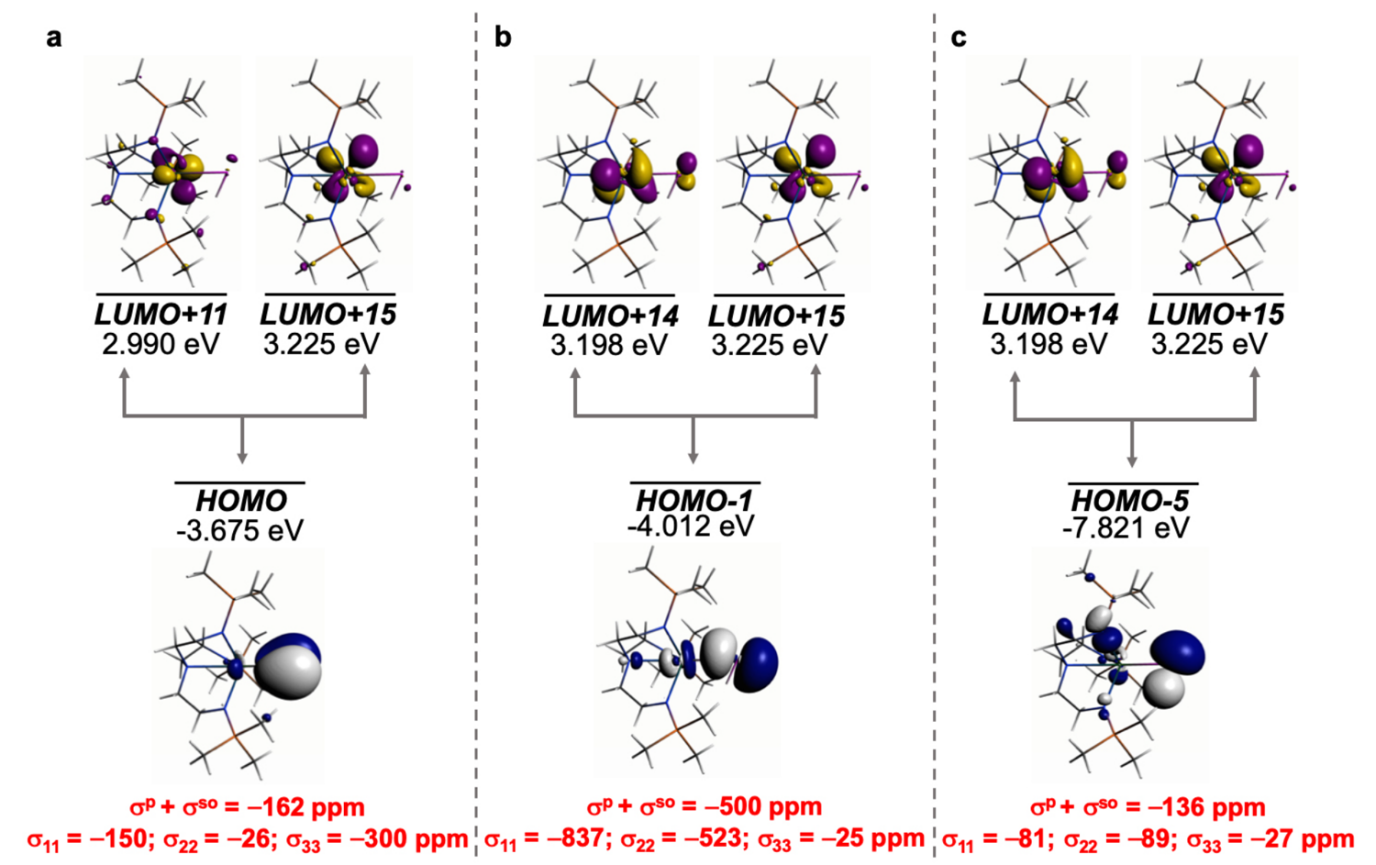
Dominant occupied and virtual MOs that contribute to the σ_iso_ and hence δ_iso_ of **2′**. (a) Magnetic coupling of the Th–P π-bond with Th 5f/6d hybrids of pseudo π* character. (b) Magnetic coupling of the Th–P σ-bond with Th=P π* and Th 5d/6d orbitals. (c) Magnetic coupling of the P-lone pair with Th=P π* and Th 5f/6d orbitals. The isotropic shielding values for the individual bonding components are given in red, and each is broken down into its principal component contributions.

**Figure 8:**
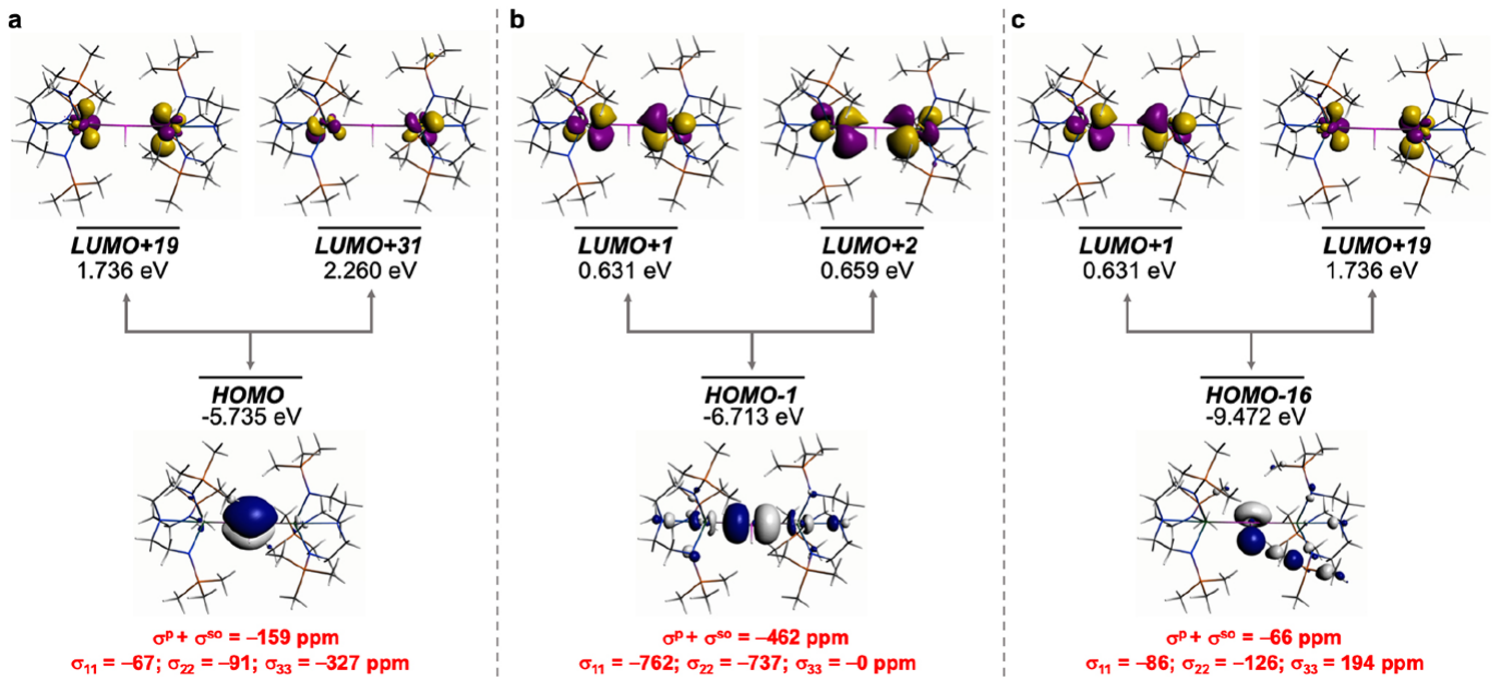
Dominant occupied and virtual MOs that contribute to the σ_iso_ and hence δ_iso_ of **3′**. (a) Magnetic coupling of the Th–P π-bond with Th 5f/6d hybrids. (b) Magnetic coupling of the Th–P σ-bond with Th=P π* and Th 6d orbitals. (c) Magnetic coupling of the P-lone pair with Th=P π* and Th 6d orbitals. The isotropic shielding values for the individual bonding components are given in red, and each is broken down into its principal component contributions.

**Figure 9:**
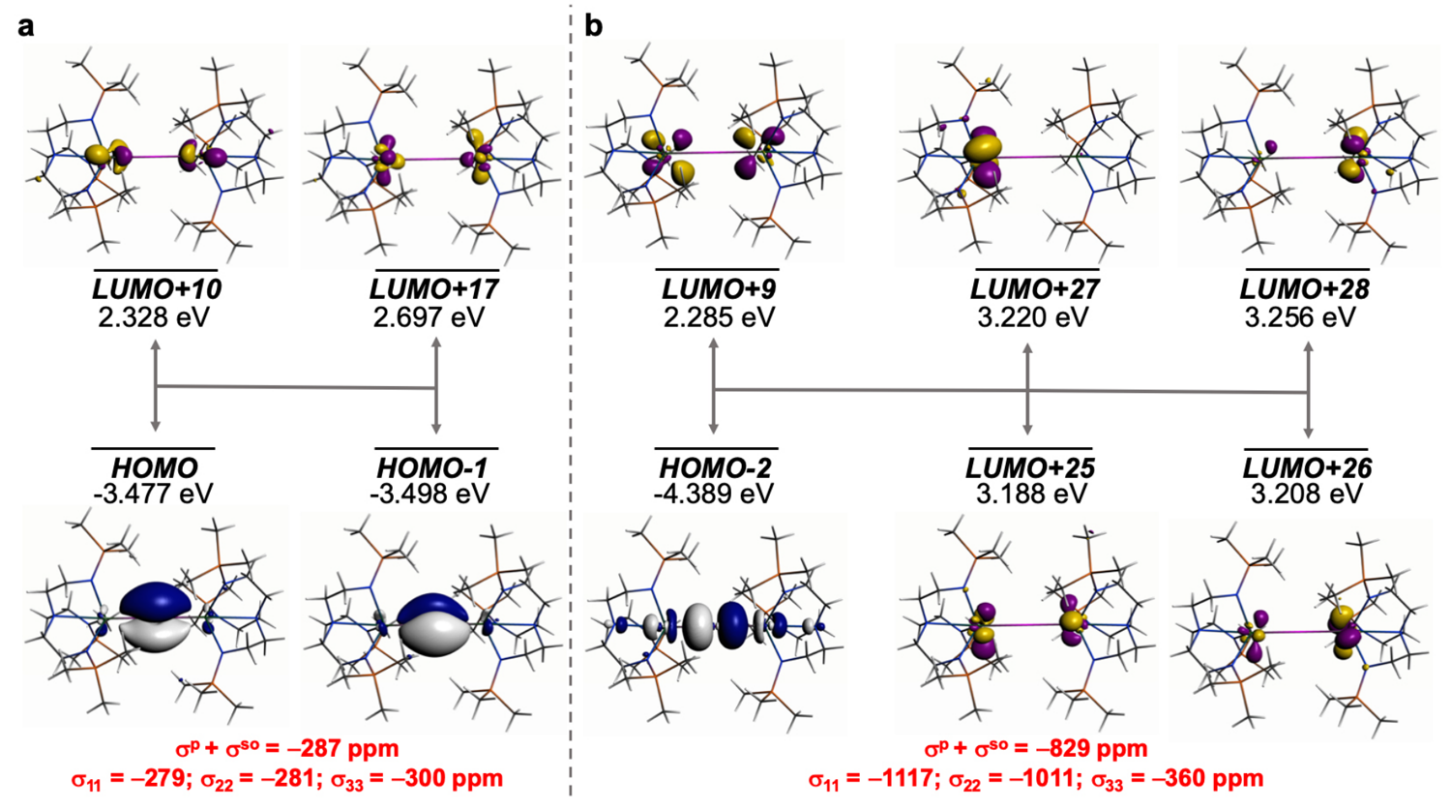
Dominant occupied and virtual MOs that contribute to the σ_iso_ and hence δ_iso_ of **4′**. (a) Magnetic coupling of the two Th–P π-bonds with Th 5f/6d hybrids. (b) Magnetic coupling of the Th–P σ-bond with Th=P π* and Th 5d/6d orbitals. The isotropic shielding values for the individual bonding components are given in red, and each is broken down into its principal component contributions.

MOs are often delocalized, and so their contributions to shielding can be distributed over numerous components, making them difficult to identify. Indeed, we note that the virtual MO manifolds of **1′**–**4′** become increasingly densely packed and extensively delocalized resulting in some MO combinations becoming impossible to identify. However, the principal components that contribute to the σ^p^+σ^so^ terms of **1′**–**4′** could be identified, thus permitting insight into the components that produce the observed δ_iso_ values and hence the Th–P bonding in **1**–**4**. The results of this analysis are presented in Figures 6–9, and where magnetic coupling combinations could be clearly identified they conform to the requirements that magnetic coupling of occupied and virtual orbitals must be symmetry allowed—since the angular momentum operators belong to the same irreducible representations as the rotational operators—and those rotationally orthogonal MO combinations should be spatially and energetically reasonably proximate.^81^

For **1′**, Figure 6, of the σ^p^+σ^so^ total of −469 ppm, −259 ppm is accounted for by field-induced magnetic coupling of the Th–P HOMO σ-bond with Th MOs of 6d- and 5f-parentage, one of which, LUMO+1, has the appearance of pseudo π*-character. This is supplemented by magnetic coupling of the Th–P HOMO–4 P-lone pair with the LUMO and LUMO+1 accounting for another −145 ppm, resulting in a value of −404 of the −469 ppm being found by this method.

As anticipated, the analysis for **2′** becomes more complicated than that of **1′**, Figure 7. The Th=P HOMO π-orbital magnetically couples with Th MOs of 6d- and 5f-character, the Th=P HOMO–1 σ-orbital magnetically couples with Th=P π* and Th 5f/6d LUMOs +14 and +15, and additionally, the P-lone pair (HOMO–5) also magnetically couples with LUMOs +14 and +15. We note that the Th=P σ-bond is the dominant contributor (−500 ppm), followed by the π-bond (−162 ppm) and then P-lone pair (−136 ppm). Altogether these three components account for −798 of the −834 ppm for the σ^p^+σ^so^ total for **2′**.

Perhaps unsurprisingly the analysis for the phosphinidiide **3′**, Figure 8, is similar to the phosphinidene **2′**. Specifically, the Th–P HOMO π-orbital magnetically couples with Th 6d-/5f-hybrids (LUMOs +19 and +31), the Th–P–Th HOMO–1 σ-linkage magnetically couples with pseudo π*-/6d-character of the LUMOs +1 and +2, and the P-lone pair HOMO–16 orbital magnetically couples with LUMOs +1 and +19. Similarly to **2′**, these MO combinations follow a pattern of the Th–P σ-bond being the largest contributor (−462 ppm), followed by the π-bond (−159 ppm), and then the P-lone pair (−66 ppm). This totals a value of −687 of −793 ppm for the σ^p^+σ^so^ total for **3′**.

The removal of the proton from **3′** to generate **4′** is reflected in a MO shielding analysis that is distinct to **1′**–**3′**, Figure 9. The two Th–P π-bonds (HOMO and HOMO–1) magnetically couple with vacant Th 6d/5f hybrids (LUMOs +10 and +17) contributing −287 ppm, which is substantial but not double the Th=P π-contribution in **2′** likely reflecting the bridging nature of the phosphido center in **4′**. However, the Th–P HOMO–2 Th–P–Th σ linkage makes a significant contribution of −829 ppm, magnetically coupling to a variety of virtual Th–P π*-/6d- and 5f-/6d-orbital hybrids (LUMOs +9, +25 to +28). In total, the magnetic coupling of these MOs generates a total of −1116 ppm out of −1181 ppm for the σ^p^+σ^so^ total for **4′**.

What is clear from this analysis is that as the strength and multiplicity of the Th–P bonding interactions build up from **1** to **3** to **2** to **4**, the σ^p^+σ^so^ totals likewise increase (*cf*. the 1/r^3^ argument above). Also, while there are clearly Th=P π-bonds present in **2** and **4** and that their contributions to the overall σ^p^+σ^so^ shieldings are not insignificant, the σ-bonds dominate the overall strength of the σ^p^+σ^so^ response in each case, which is then interleaved with the s-character of the Th and P bonding and the transmission of spin–orbit effects from Th to P which also reflects the 5f vs 6d contributions to the Th bonding.

As shown in Figures 6–9, the individual σ^p^+σ^so^ values for the Th–P σ, Th–P π, P–H σ, and P lone pair components of the Th–P bonding in **1′**–**4′** can be broken down into their individual σ_11_, σ_22_, and σ_33_ values, providing some insight into the orbitals that principally contribute to the chemical shielding tensors. Figures 6–9 clearly indicate that large deshielding in directions perpendicular to the Th–P bonds (*z* axis) is associated with the magnetic coupling of the occupied Th–P σ-bond with various virtual π*-type orbitals. A larger number of low lying π*-type orbitals are also associated with increased deshielding of the P nucleus. The rotated orbital model can provide significant insight when a common bonding motif with different substituents is examined, but here there are four different bonding motifs, and this combined with the complexity of the MO manifolds of **1′**–**4′** renders some MO combinations impossible to identify. Due to the restrictions of the MO approach, we conducted a NLMO-NMR analysis since this provides a fuller picture.

### Natural Localized Molecular Orbital Shielding Analysis of **1′**–**4′**

The NLMO analysis of **1′**–**4′** is compiled in Tables 3 and 4, and this methods describes Lewis and non-Lewis components of the bonding,^87,88^ though we note that the analysis shows the non-Lewis components are minor with the Lewis components dominating the shielding. This is the case for SR and SOR models, which, by difference, permits the spin–orbit shielding effects to be determined. In each case, the NLMOs are very similar to the corresponding NBOs, so since NLMOs are in essence NBOs that are expanded to achieve an orbital occupancy of 100% (2 electrons), by allowing other minor orbital coefficients to intrude, the NLMO analysis is valid.

**Table 3:**
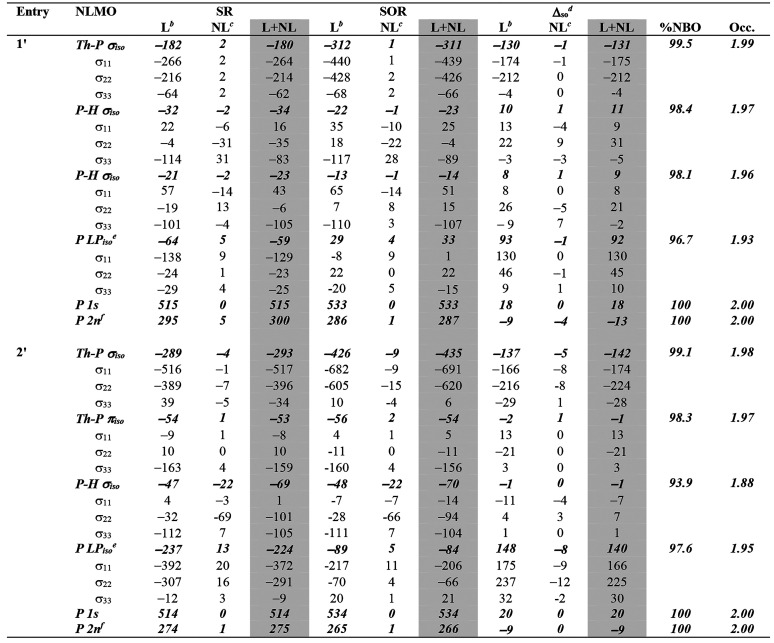
Natural Localized Molecular Orbital Contributions to the Principal ^31^P Nuclear Shielding Components (σ^d^ + [σ^p^ + σ^so^]) of **1′** and **2′**^a^

a B3LYP-HF50 TZ2P all-electron ZORA SR or SOR level in a benzene solvent continuum, all shielding parameters are in ppm.

b Lewis contribution of the NLMO.

c Non-Lewis contribution of the NLMO.

d Defined as σ(SOR) – σ(SR) to isolate the SO component.

e Lone pair.

f 2s+2p orbitals.

**Table 4:**
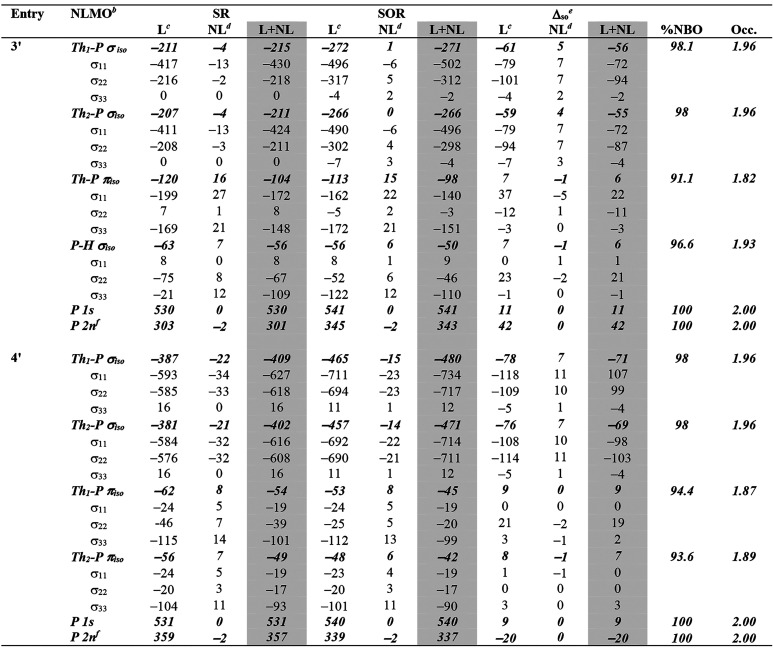
Natural Localized Molecular Orbital Contributions to the Principal ^31^P Nuclear Shielding Components (σ^d^ + [σ^p^ + σ^so^]) of **3′** and **4′**^a^

a B3LYP-HF50 TZ2P all-electron ZORA SR or SOR level in a benzene solvent continuum, all shielding parameters are in ppm.

b The Th_1_ and Th_2_ labels are used to distinguish the two slightly different sets of Th–P shielding data but the labeling is arbitrary.

c Lewis contribution of the NLMO.

d Non-Lewis contribution of the NLMO.

e Defined as σ(SOR) – σ(SR) to isolate the SO component.

f 2s+2p orbitals.

The NLMO analysis reveals that the principal NLMOs involved in providing the relevant shielding for **1′**–**4′** are, where present for a given molecule, the Th–P σ and π, P–H σ, P-lone pair, and core P orbitals (1s, 2s, 2p), which can be related back to the σ^p^ (Th–P) and σ^d^ (P-core) components of the MO shielding analysis. These NLMOs largely correlate to the corresponding NBOs, with percentage representations of ≥91.1 and electron occupancies of ≥1.87.

Focusing on the SOR data, for **1′**–**4′** it is clear that the largest negative shielding component of each Th–P bond derives from the σ-components, with Lewis + non-Lewis totals being −311, −435, −537, and −951 ppm, respectively. The shielding response from the Th–P π-bonds is then smaller, being −54, −98, and −87 ppm for **2′**–**4′**, respectively. The P–H σ-bond contributions vary from −26 ppm for **1′** to −70 ppm for **2′** to −50 ppm for **3′**. Arrayed against these negative shielding parameters are the P-components, which are substantial for the 1s contributions alone, being 533, 534, 541, and 540 ppm for **1′**–**4′**.

The Δ_so_ data reveal that the Th–P σ-bonds are most greatly affected by spin–orbit effects but the Th–P π-bonds are far less affected. This can be related back to the bonding analysis, which showed substantial P s-character in the Th–P σ-bonds, but virtually exclusive P p-character in the Th–P π-bonds. Thus, the presence of s-character permits spin-polar transmission of spin–orbit effects, but this is greatly reduced in the π-bonds. The P-lone pairs contain s-character, and notably, the Δ_so_ data reveal that they too exhibit large spin–orbit effects, whereas unsurprisingly, the core P orbitals are little affected by spin–orbit effects. Overall, these data are in agreement with the MO analysis and confirm that the δ_iso_ and hence shielding properties of the Th–P linkages in **1′**–**4′** are overwhelmingly dependent on the Th and P atoms with minor contributions from the P–H bonds where present.

Noting that the δ_iso_ and hence shielding properties of the Th–P linkages in **1′**–**4′** are dependent on the Th and P atoms it is then instructive to examine the NLMO breakdown of each (σ^d^ + [σ^p^ + σ^so^]) component of the Th–P bonds. The NLMO analysis does not explicitly identify the localized vacant orbitals that are magnetically coupled to the occupied orbitals, but using the MO shielding analysis above as a guide, the individual σ_11_, σ_22_, and σ_33_ values from the NLMO analysis can be inspected and rationalized; it is intuitively most straightforward to start with the axially symmetric **4′**.

For **4′**, Table 4, both Th–P σ-bonds (in reality the 3c2e Th–P–Th P 3p NBO interactions expressed as separate combinations in the NLMO framework) are aligned along the principal *z*-axis, and exhibit large σ_11_ and σ_22_ deshieldings that can be visualized as resulting from 90° rotation of those P 3p-orbitals into the *y* and *x* directions; this is the magnetic coupling of σ and π* Th–P bonds by the action of *L*_*x*_ and *L*_*y*_, respectively. The two Th–P π-bond P-orbitals are each aligned along the principal *x* and *y* axes, and each exhibits a large σ_33_ deshielding (along *z*), which is due to the magnetic coupling of these occupied orbitals with virtual π*-orbitals via the action of *L*_*z*_.

The presence of the P–H H atom in **3′** results in an asymmetry of the bonding at P which can be clearly seen in the NLMO data, Table 4. The two Th–P σ-bonds (again, in reality the 3c2e Th–P–Th P 3p NBO interaction expressed as separate combinations in the NLMO framework) are aligned along the principal *z*-axis. Their σ_11_ and σ_22_^31^P deshielding values contain contributions owing to the magnetic coupling of the occupied Th–P σ-orbital with virtual π*-type orbitals via the action of *L*_*x*_ and *L*_*y*_. However, unlike for **4′**, σ_11_ and σ_22_ are now no longer approximately equal, with each exhibiting a larger value (av. –499 ppm) and a smaller value (av. −305 ppm); this asymmetry results from the P–H σ-bond being aligned along the same axis as σ_11_ and the Th–P dative π-symmetry “lone pair” residing along the same axis as σ_22_. This can likewise be seen in the data for those two linkages, each of which contains a large σ_33_ deshielding that results from magnetic coupling of the P–H or P π 3p-orbitals from the principal *x-* and *y*-axes with the π*- and P–H σ*-vacant orbitals via the action of *L*_*z*_. Interestingly, the Th–P π-bond P 3p-orbital exhibits a significantly deshielded σ_11_ value, which is due to the magnetic coupling of this orbital with the Th–P σ* orbital (action of *L*_*x*_), but the corresponding σ_22_ deshielding value for the P–H σ-linkage due to magnetic coupling with the Th–P σ* orbital is far smaller.

For **2′**, the Th–P bond is aligned along the principal *z*-axis, and hence it exhibits large σ_11_ and σ_22_ deshielding values; the former results from rotation of the Th–P σ-bond into the Th–P π* orbital by the action of *L*_*x*_, and the latter results from orthogonal Th–P σ–π* magnetic coupling by the action of *L*_*y*_. The Th–P π-bond is aligned with the σ_22_ principal component, and it exhibits a large σ_33_ deshielding value that results from π–σ*(P–H)/π*(Th–P) magnetic coupling by the action of *L*_*z*_. The P–H bond is only slightly off the same axis as σ_11_, which is reflected by large σ_22_ and σ_33_ deshielding values that stem from magnetic coupling of the associated P 3p orbital with σ* (Th–P) and π* (Th–P) virtual orbitals, respectively, by the action of *L*_*y*_ and *L*_*z*_. The P lone pair is almost aligned with the direction of the σ_33_ principal component (and hence *z* axis), but deviates sufficiently for its σ_11_ and σ_22_ deshielding values to be rather different, and this is magnetic coupling of the P lone pair with the P–H π* (σ_11_) and σ* (σ_22_) bonds by the action of *L*_*x*_ and *L*_*y*_.

Lastly, for **1′** again the Th–P σ-bond is aligned along the principal *z*-axis (and the direction of the σ_33_ principal component), which is reflected in its σ_11_ and σ_22_ deshielding values being substantial via the action of *L*_*x*_ and *L*_*y*_. However, the two P–H bonds and P-lone pair are in between the principal *x*- and *y*-axes (and are not aligned with the directions of σ_11_ or σ_22_), which makes their σ_11_ and σ_22_ shielding data relatively uninformative, but the effect on σ_33_ of magnetic coupling of the P–H orbitals with Th–P π*-orbitals by the action of *L*_*z*_ is clear. Nevertheless, it is evident from the NLMO data that it is the Th–P σ-bond that is most important in determining the δ_iso_ value of **1′**.

### Correlating ^31^P NMR Spectroscopic Chemical Shift to Metal–Phosphorus Bond Order

**Figure 10:**
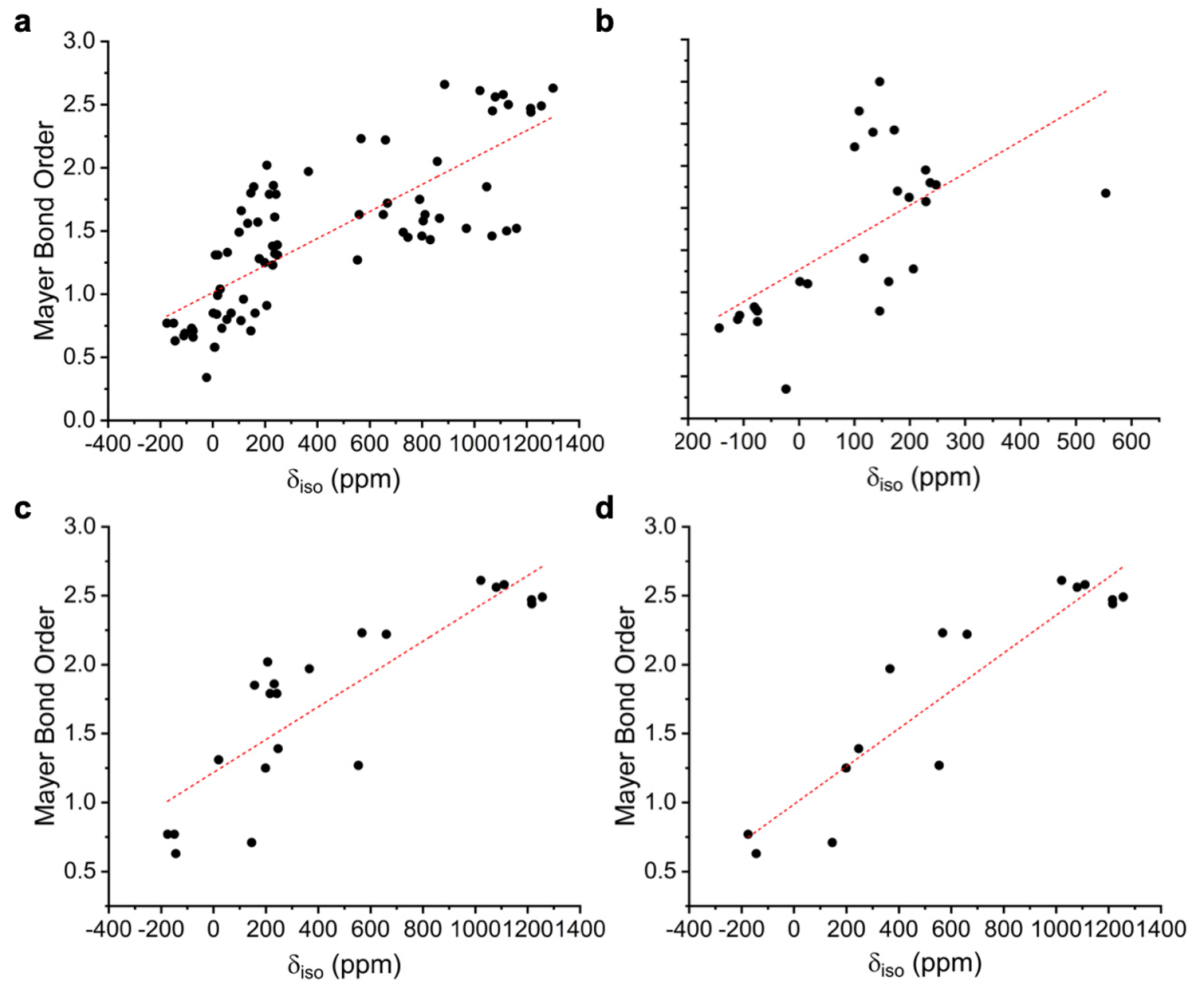
Correlations of experimental solution ^31^P NMR δ_iso_ data vs computed MBOs of representative metal P-ligand complexes, see Table S6 for the list of complexes. (a) Th and transition metal N-, Cp-, alkyl-, and aryloxide-ligands complexes **1**–**61**, linear regression: MBO = (0.0011 × δ_iso(exp)_) + 1.0133, R^2^ = 0.6122. (b) Th complexes **1**–**19** and **50**, linear regression: MBO = (0.0015 × δ_iso(exp)_) + 0.9072, R^2^ = 0.3860. (c) Th and transition metal N-ligand complexes **1**–**4**, **22**, **23**–**27**, **31**, **32**, **36**–**42**, **45**, and **46**, linear regression: MBO = (0.0011 × δ_iso(exp)_) + 1.2192, R^2^ = 0.6861. (d) Th and transition metal amide-ligand complexes **1**–**4**, **31**, **32**, **36**–**42**, **45**, and **46**, linear regression: MBO = (0.0014 × δ_iso(exp)_) + 0.9881, R^2^ = 0.8445.

With the bonding and shielding properties of **1′**–**4′**, and hence **1**–**4** determined, quantified, and rationalized, we sought to correlate the observed δ_iso_ values to the metal–phosphorus bond orders. We surveyed the literature and selected a range of representative compounds that were structurally characterized, have clear ^31^P NMR data, and cover the range of dative single bond phosphines, covalent single bond phosphanides, covalent double bond phosphinidenes, and covalent triple bond phosphidos. In addition to **1**–**4**, this adds an additional 57 complexes to the analysis, spanning Th, Sc, Ti, Zr, Nb, Mo, W, Re, Ru, Os, Co, Rh, Ir, and Ni metals, all computed at the same B3LYP level as **1**–**4**, Table S6.^50−56,59,60,67,70,73,75,76,85,89−117^ We thus plotted δ_iso_ values vs computed MBOs for a wide range of Th and groups 3–10 of the transition metals, Figure 10.

The plot of all compounds, Figure 10a, shows a general trend of increasing MBO with increasing δ_iso_, and hence increased deshielding, which as suggested by the analysis above will be most dependent on the σ^p^ property. However, linear regression shows the correlation to be *R*^2^ = 0.6122. It should be noted that this collection of compounds has a very wide range of ancillary ligands, where no trend is discernible in terms of ancillary ligand variations, and also as evidenced by the analysis of **1**–**4** and **1′**–**4′** σ^so^ is variable which will skew the results compared to an analysis based on σ^p^ alone. This contrasts to the situation we recently reported for amine, amide, imido, and nitride analogues,^37^ where those ligands are the strongest donors and so other ligands are very much in a secondary ancillary role and hence the *R*^2^ correlation is much better even for a wide range of coligands. Nevertheless, when considering all compounds examined in terms of the coordinated P-ligand, some clear trends emerge. This divides Figure 10a into four quadrants, as follows: (i) “top left” (MBO > ∼1.25; δ_iso_ < 500 ppm) early metal terminal phosphinidenes; (ii) “top right” (MBO > ∼1.5; δ_iso_ > 500 ppm) early metal terminal phosphidos; (iii) “bottom left” (MBO < ∼1; δ_iso_ < 500 ppm) bridging phosphinidiides and M–P single bonds; (iv) “bottom right” (MBO ∼ 1.2–1.7; δ_iso_ > 500 ppm) late d-block terminal phosphinidenes. Thus, in general, compounds with the most well-developed M–P multiple bonds that will exhibit the most covalency tend to sit above the dotted red least-squares fit line in Figure 10a, whereas those that are more polarized, or are bonded to electron-rich metals where population of antibonding orbitals may be becoming a factor, sit below the line. The results here for P-ligands suggests that P-complexes are much more varied as a consequence of the ancillary ligands, which reflects the relative hardness of N vs P in HSAB theory, that is the P-ligand environments are more sensitive to other ligands. To probe this hypothesis further we analyzed the data in more depth.

Figure 10b shows a plot of all of the Th complexes examined in this study. The *R*^2^ = 0.3860 is worse than the plot of all complexes. This reflects the wide range of ancillary ligands and also that with even greater numbers of compounds, metals, and ligands, averaging effects occur. This suggests that the ancillary ligands indeed play a significant role in determining how electrophilic the Th ions will be, hence directly modulating the Th–P bonds in question, which will then directly impact on the σ and then δ properties. When the scope of ancillary ligands is narrowed down to N-donor ligands, Figure 10c, the *R*^2^ value improves markedly to 0.6861, which supports the arguments presented above about ancillary ligand effects. Indeed, when the range of ancillary ligand types is narrowed further to only amides, Figure 10d, then *R*^2^ = 0.8445. Thus, the conclusion is clear, while modeling hard nitrides, imides, and amides can largely be done irrespective of the nature of any other ancillary ligands; because phosphorus is softer than nitrogen when the same analysis is applied to M–P complexes, the correlation is not as good because the ancillary ligands are not secondary but also effectively also primary drivers of σ_iso_ and hence δ_iso_. We thus conclude that when investigating the covalency of M–P bonds by ^31^P NMR spectroscopy it is necessary to bound correlations within the same family of ancillary ligands, whereas this is not necessary for M–N bonds. Nevertheless, the correlation equations provided in Figure 10 may form the basis for qualitative general assessments of δ_iso_ versus MBOs across all ligand types and may be regarded as more quantitative for amide ancillary ligand complexes.

## Summary and Conclusions

In summary, we have examined the solution and SS ^31^P NMR spectroscopy of a family of four Th complexes which exhibit Th–PH_2_, Th=PH, Th–P(H)–Th, and Th=P=Th linkages in **1**–**4**. Through modeling of SS data and computational analysis the δ_iso_ values have been decomposed into their constituent chemical shift tensors giving information about the chemical shift anisotropies of the P-environments in **1**–**4** and revealing a record CSA for a bridging phosphido center. The ^31^P SS NMR data for **1**–**4** demonstrate a CSA ordering of (μ-P)^3–^ > (=PH)^2–^ > (μ-PH)^2–^ > (−PH_2_)^1–^. Thus, this work has introduced ^31^P NMR spectroscopy for quantitative determination of metal–ligand covalency in f-element chemistry.

Inspection of the σ_iso_ data has enabled dissection into σ^d^, σ^p^, and σ^so^ contributions, revealing invariant σ^d^, but highly variable σ^p^ and σ^so^. This reflects the Th–P bond multiplicities and also the amount of s-character in the Th–P bonds that permits transmission of spin–orbit effects from Th to P by spin-polar effects. Shielding analysis has revealed the nature of the MOs that contribute to the σ_iso_, and hence δ_iso_, values by magnetic coupling of filled and orthogonal virtual orbitals where in each case the P δ_iso_ value is virtually exclusively due to the nature of the Th–P bonds. It is clear that the Th–P σ-bonds dominate over the σ tensors, as these are the most affected by spin orbit effects, but the π-bond contributions are not insignificant.

By experimentally benchmarking the data, the nature of the Th–P bonding has been quantified by NBO and NLMO methods, and it is interesting to note that although there are variations between experimentally benchmarked B3LYP-HF50-SOR and model BP86 calculations the data are remarkably similar between the two functionals, which confirms the unusual quite significant 7s contributions to the Th–P bonds of **1**–**4** where normally 6d/5f-orbital character (and 6d > 5f) is the dominant feature of Th chemical bonding. This suggests that BP86 is quite appropriate for computing coarse parameters, e.g., orbital % contributions and bond orders, but B3LYP is needed for detailed calculation of heavily spin–orbit dependent parameters like δ_iso_.

This study has permitted us to correlate the Th–P δ_iso_ values to MBOs over a wide range of metals spanning the early, mid, and late d-block in addition to Th. However, we find that the correlation is very much dependent on the ancillary ligands, highlighting an important difference between N- and P-ligands, which is that N-ligands tend to be the dominant component of the ligand field with ancillary ligands in a secondary role, whereas trends with the softer P-ligands are clearly much more ancillary ligand dependent. We thus conclude that when investigating the covalency of M–P bonds by ^31^P NMR spectroscopy, it is necessary to bound correlations within a given family of ancillary ligands, whereas this is not necessary for M–N bonds. Nonetheless, the correlation equations provided in this work may form the basis for qualitative initial assessments of δ_iso_ versus MBOs across all ligand types and may be regarded as more quantitative for amide ancillary ligand complexes.

## Experimental Section

### Experimental Details

The compounds [Th(PH_2_)(Tren^TIPS^)] (**1**, Tren^TIPS^ = {N(CH_2_CH_2_NSiPr^i^_3_)_3_}^3–^), [Th(PH)(Tren^TIPS^)][Na(12C4)_2_] (**2**, 12C4 = 12-crown-4 ether), [{Th(Tren^TIPS^)}_2_(μ-PH)] (**3**), and [{Th(Tren^TIPS^)}_2_(μ-P)][Na(12C4)_2_] (**4**) were prepared as described previously.^52a^ The formulations and purity were confirmed by ^1^H, ^13^C{^1^H}, ^29^Si{^1^H}, ^31^P, and ^31^P{^1^H} NMR spectra recorded in C_6_D_6_ or D_8_-THF; those spectra are essentially the same as the originals, but given the identified ^31^P δ_iso_ issue for **2** and **3** all ^31^P NMR spectra are provided in the Supporting Information.

Solution ^1^H, ^13^C{^1^H}, ^29^Si{^1^H}, and ^31^P{^1^H} NMR spectra were recorded on a Bruker AV III HD spectrometer operating at 400.07, 100.60, 79.48, and 161.95 MHz, respectively; chemical shifts are quoted in ppm and are relative to TMS (^1^H, ^13^C, and ^29^Si) and 85% H_3_PO_4_ (^31^P), respectively. Solid-state direct excitation ^31^P NMR spectra were recorded by using a Bruker 9.4 T (400 MHz ^1^H Larmor frequency) AVANCE III spectrometer equipped with a 4 mm HFXY MAS probe. Experiments were acquired at ambient temperature using various MAS frequencies. Samples were packed into 4 mm o.d. zirconia rotors in a glovebox, and sealed with a Kel-F rotor cap. The ^31^P (π/2)-pulse duration was 4 μs, and a Hahn-echo τ_r_–π–τ_r_ sequence of 2 rotor periods total duration was applied to ^31^P after the initial (π/2)-pulse to circumvent receiver dead-time. The signal was acquired for ∼10 ms and between 128 and 3744 transients were coadded, with repetition delays of 3.2 s. Magnitude phase correction was used for the ^31^P MAS NMR spectrum of **4** owing to the large spectral range of the spinning side bands. The degradation product of **2** exhibits an antiphase ^31^P NMR peak, likely due to evolution of the *J*_PH_ coupling under the employed echo. Spectral simulations were performed in the solid line-shape analysis (SOLA) module v2.2.4 in Bruker TopSpin v4.0.9. The ^31^P chemical shifts were referenced to 85% H_3_PO_4_ externally using ammonium dihydrogen phosphate (0.8 ppm). Care must be taken with air sensitive compounds to minimize sample decomposition during measurements, and due consideration must be given to if data are robust if any minor decomposition occurs. Static variable-temperature magnetic moment data were recorded in an applied dc field of 0.1 T on a Quantum Design MPMS 3 superconducting quantum interference device (SQUID) magnetometer by using recrystallized powdered samples. Care was taken to ensure complete thermalization of the sample before each data point was measured, and samples were immobilized in an eicosane matrix to prevent sample reorientation during measurements. Diamagnetic corrections were applied using tabulated Pascal constants, and measurements were corrected for the effect of the blank sample holders (flame-sealed Wilmad NMR tube and straw) and eicosane matrix.

### Computational Details

Restricted calculations were performed using the Amsterdam Density Functional (ADF) suite version 2017 with standard convergence criteria.^118,119^ Geometry optimizations were performed using coordinates derived from the respective crystal structures as the starting points and were either the full models **1**–**4** (for **1** with two molecules in the asymmetric unit we used the molecule with the Th–P distance of 2.9817 Å) with the noncoordinating cation components in **2** and **4** removed or the analogous truncated models **1′**–**4′**. Analogously, for the correlation study published crystallographic coordinates were used as the starting point for calculations on **5**–**50** (with any noncoordinating ions removed from the models). The H atom positions were optimized, but the non-H atom positions were constrained as a block. The DFT geometry optimizations employed Slater type orbital (STO) TZ2P polarization all-electron basis sets (from the Dirac and ZORA/TZ2P databases of the ADF suite). Scalar relativistic (SR) approaches (spin–orbit neglected) were used within the ZORA Hamiltonian^120−122^ for the inclusion of relativistic effects, and the local density approximation (LDA) with the correlation potential due to Vosko et al. was used in all of the calculations.^123^ Generalized gradient approximation corrections were performed using the functionals of Becke and Perdew.^124,125^ See the Supporting Information for final coordinates and energies, Tables S7–S71.

SR and two-component spin–orbit relativistic (SOR) ZORA TZ2P polarization all-electron single point energy calculations were then run on the geometry optimized coordinates. The conductor-like screening model (COSMO) was used to simulate solvent effects. The functionals screened included BP86, SAOP, B3LYP-HFXX (XX = 20 (default in ADF), 30, 40, and 50%), and PBE0-HFXX (XX = 25 (default in ADF) and 40%). Overall, the B3LYP-HF50 functional gave the closest agreement of computed NMR properties compared to experiment, so it was selected for further in-depth study. MDC_q_ charges and MBOs were computed within the ADF program.

NBO and NLMO analyses were carried out using NBO6.^126^ The MOs, NBOs, and NLMOs were visualized by using ADFView.

NMR shielding calculations were carried out using the NMR program within ADF.^87,88,127−131^ Calculated nuclear shieldings were converted to chemical shifts by subtraction from the calculated nuclear shielding of PH_3_ calculated at the same level (SR σ_iso_ = 571.7; SOR σ_iso_ = 584.5 ppm) and correcting for PH_3_ δ_iso_ = −240 ppm with respect to 85% H_3_PO_4_.^132−134^ MO contributions to the nuclear shieldings were calculated at the scalar and two-component spin–orbit levels, the former with the FAKESO key. Scalar and two-component spin–orbit NLMO-NMR calculations of the computed nuclear shieldings were carried out using NBO6 and ADF. These calculations used the Hartree–Fock RI scheme to suspend the dependency key and avoid numerical issues. Shielding tensors (converted to chemical shift tensors) were visualized using TensorView.^135^

## Data Availability

Data are provided in the Supporting Information or are available from the authors upon reasonable request.

## Supporting Information Available

Additional experimental and computational data associated with this manuscript (PDF)
