# Supplementary material for: 31P Nuclear Magnetic Resonance Spectroscopy as a Probe of Thorium–Phosphorus Bond Covalency: Correlating Phosphorus Chemical Shift to Metal–Phosphorus Bond Order
Source: J Am Chem Soc. 2023 Sep 28;145(40):21766–84. doi: 10.1021/jacs.3c02775 (PMC10571089; doi:10.1021/jacs.3c02775)
Supplement: Supplementary file 1 — ja3c02775_si_001.pdf [file ja3c02775_si_001.pdf]

– *Supporting Information* –

^{31}P Nuclear Magnetic Resonance Spectroscopy as a Probe of Thorium-Phosphorus Bond Covalency: Correlating Phosphorus Chemical Shift to Metal-Phosphorus Bond Order

Jingzhen Du,[1] Joseph Hurd,[2] John A. Seed,[1] Gábor Balázs,[3] Manfred Scheer,[3] Ralph W. Adams,[1]* Daniel Lee,[2]* Stephen T. Liddle[1]*

[1] Department of Chemistry, The University of Manchester, Oxford Road, Manchester, M13 9PL, UK.

[2] Department of Chemical Engineering, The University of Manchester, Oxford Road, Manchester, M13 9PL, UK.

[3] Institute of Inorganic Chemistry, University of Regensburg, Universitätsstr.31, 93053 Regensburg, Germany.

*Email: ralph.adams@manchester.ac.uk; daniel.lee@manchester.ac.uk; steve.liddle@manchester.ac.uk

Safety

Thorium (99.98% ^{232}Th) is a weak α-emitter (4.0816 MeV) with a half-life of 1.405×10^{10} years. Whilst most manipulations can be carried out safely using Schlenk lines or glove boxes with appropriate personal protective equipment, logging, and monitoring regime, particular care should be exercised when embarking on solid-state magic angle spinning (SS-MAS) NMR experiments. A full risk assessment and protocols for loading, unloading, and actions in the event of a rotor crash and contamination should be completed before undertaking SS-MAS-NMR experiments.

Figures

Solution-State ³¹P NMR Spectra

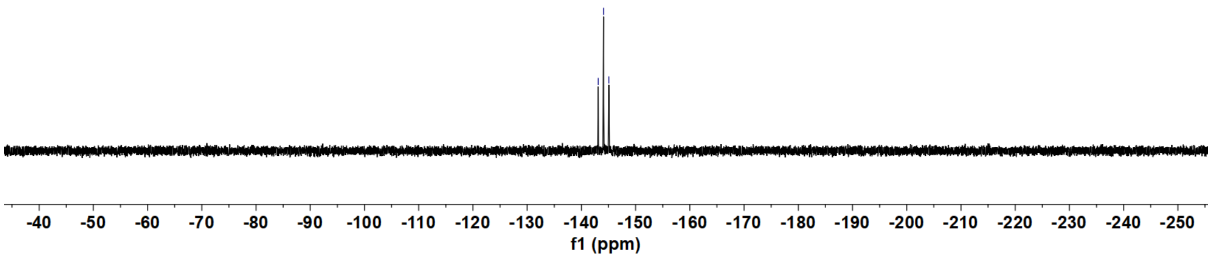

Figure S1. ^{31}P NMR (162 MHz, C_6D_6, 298 K) of [Th(PH_2)(Tren^{TIPS})] (**1**).

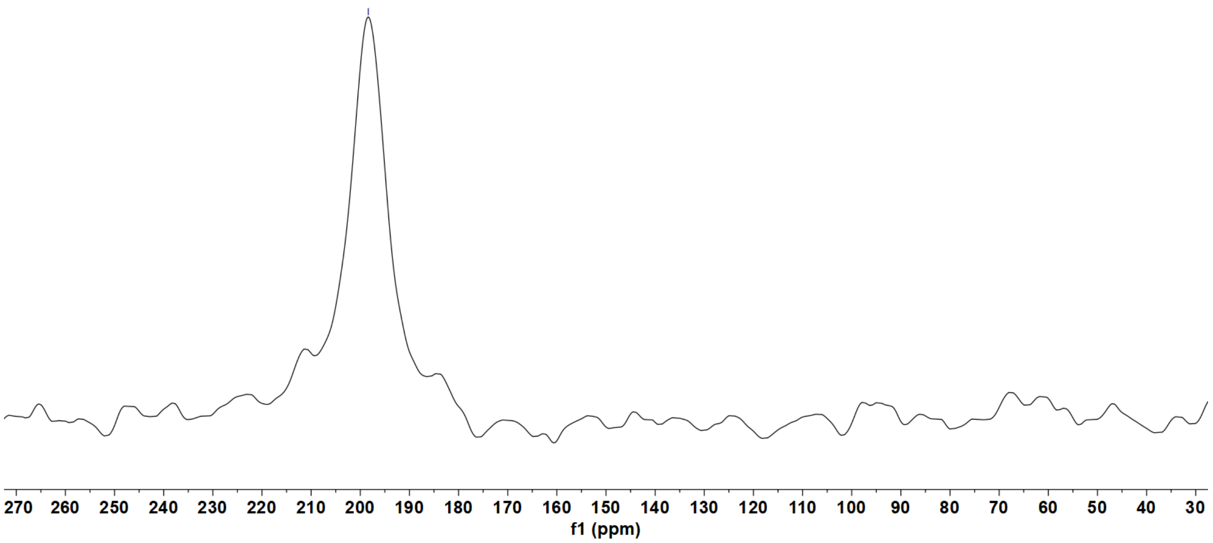

Figure S2. ^{31}P NMR (162 MHz, C_6D_6, 298 K) of [Th(PH)(Tren^{TIPS})][Na(12C4)$_2$] (**2**).

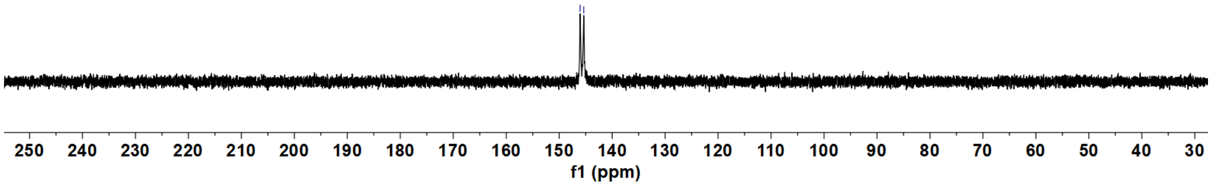

Figure S3. ^{31}P NMR (162 MHz, D_8-THF, 298 K) of [{Th(Tren^{TIPS})}$_2$(μ-PH)] (**3**).

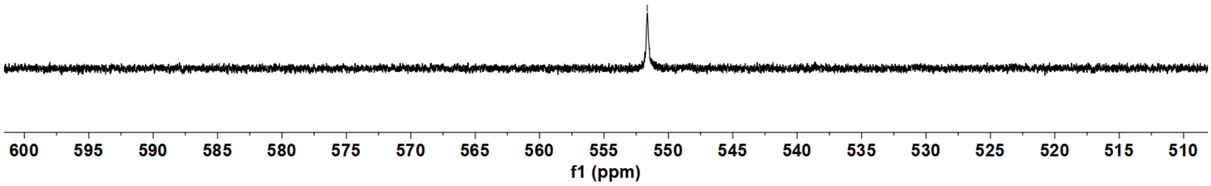

Figure S4. ^{31}P NMR (162 MHz, D_8-THF, 298 K) of [{Th(Tren^{TIPS})}$_2$(μ-P)][Na(12C4)$_2$] (**4**).

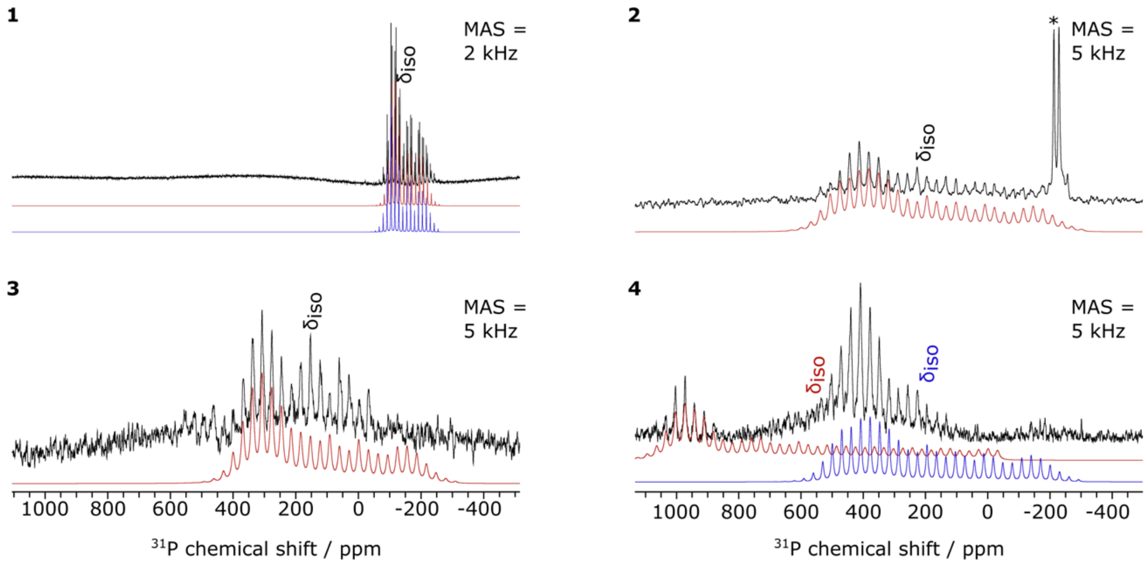

Figure S5. SS-MAS ^{31}P NMR spectra for **1**-**4**. Simulations (red and blue) are provided for the experimental data (black). The position of the isotropic peak is indicated (δ_{iso}) for each complex and a degradation product of **2** is highlighted by an asterisk (*). Note that there are two molecules in the asymmetric unit of **1** and that **4** has partially degraded into **2** during data acquisition.

SQUID Data

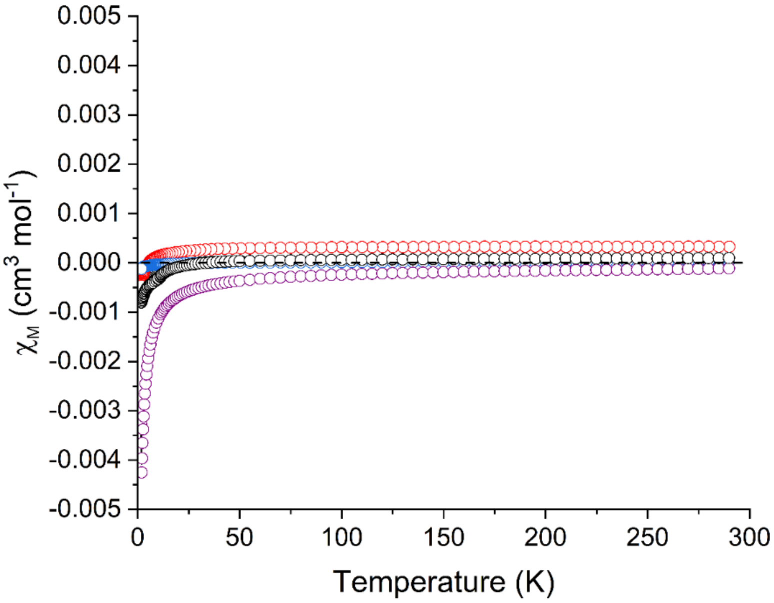

Figure S6. Magnetic susceptibility χ vs T for **1** (black circle), **2** (red circle), **3** (blue circle), and **4** (purple circle) over the temperature range 1.8-290 K.

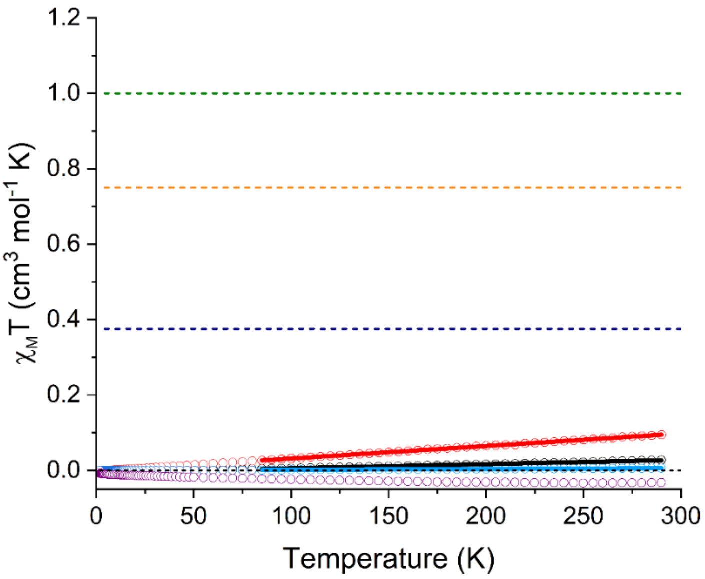

Figure S7. Magnetic susceptibility χT vs T for **1** (black circle), **2** (red circle), **3** (blue circle), and **4** (purple circle) over the temperature range 1.8-290 K. For reference, the dashed lines represent d^1 (S = ½, blue dashes), two non-correlated d^1-d^1 magnetic doublets (2 × S = ½, orange dashes), and triplet d^1-d^1 (S = 1, green dashes) expected values for g = 2 systems; the thick lines represent linear regressions (85-290 K).

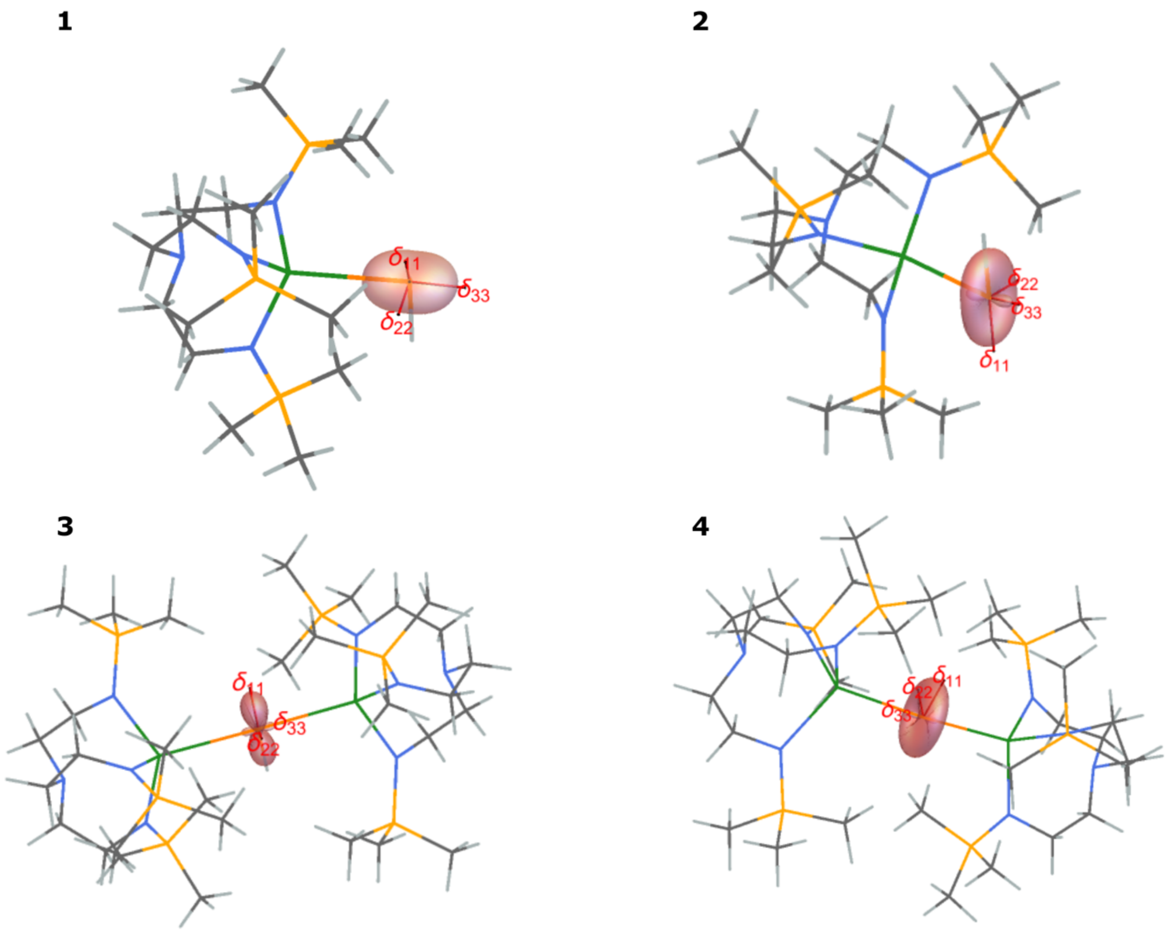

Figure S8. Plots of the δ_{11}, δ_{22}, and δ_{33} ^{31}P tensor components for **1'-4'**. The shielding surface is represented using the ovaloid convention where the distance from the P atom to a point on the surface is proportional to the chemical shift when the magnetic field is aligned along that direction in space. The shading of the surface denotes the sign of the shift where orange is positive and light orange is negative.

Tables

Table S1. Computed Scalar Relativistic (SR) and Spin-Orbit Relativistic (SOR) ^{31}P Isotropic Chemical Shift (δ_{iso}), Isotropic Shielding (σ_{iso}), Diamagnetic Shielding (σ^d), Paramagnetic Shielding (σ^p), and Spin-Orbit Shielding (σ^{so}) Values for 1 and 1' Computed With Various Functionals.

Cmpd	Functional	$\delta_{iso(expt)}$	$\delta_{iso(calc)}$	σ_{iso}	σ^d	σ^p	σ^{so}
**1**	BP86-SR		−139.3	476.9	964.9	−488.0	-
**1**	BP86-SOR		−122.7	473.1	964.8	−486.3	−5.4
**1**	B3LYP-HF20-SR		−154.7	485.3	964.0	−478.7	-
**1**	B3LYP-HF20-SOR		−131.6	475.0	964.0	−477.8	−11.2
**1**	B3LYP-HF30-SR		−163.9	494.9	963.8	−468.8	-
**1**	B3LYP-HF30-SOR		−137.7	481.6	963.7	−468.2	−14.0
**1**	B3LYP-HF40-SR		−172.1	503.4	963.5	−460.1	-
**1**	B3LYP-HF40-SOR		−142.7	486.9	963.5	−459.6	−16.9
**1**	B3LYP-HF50-SR	−144.1 (sol)	−179.3	511.0	963.3	−452.3	-
**1**	***B3LYP-HF50-SOR***	−138.9 (ss)	***−146.8***	***491.2***	***963.2***	***−452.0***	***−20.0***
**1'**	B3LYP-HF50-SR		−176.7	508.4	963.4	−455.0	-
**1'**	***B3LYP-HF50-SOR***		***−149.9***	***494.4***	***963.4***	***−454.3***	***−14.7***
**1**	PBE0-HF25-SR		−161.8	514.8	963.7	−448.9	-
**1**	PBE0-HF25-SOR		−138.0	503.9	963.7	−448.2	−11.5
**1**	PBE0-HF40-SR		−171.6	529.4	963.2	−433.8	-
**1**	PBE0-HF40-SOR		−142.9	513.7	963.1	−433.5	−16.0
**1**	SAOP-SR		−166.2	523.6	967.7	−444.1	-
**1**	SAOP-SOR		−147.9	518.1	967.7	−443.6	−6.0

Table S2. Computed Scalar Relativistic (SR) and Spin-Orbit Relativistic (SOR) ^{31}P Isotropic Chemical Shift (δ_{iso}), Isotropic Shielding (σ_{iso}), Diamagnetic Shielding (σ^d), Paramagnetic Shielding (σ^p), and Spin-Orbit Shielding (σ^{so}) Values for 2 and 2' Computed With Various Functionals.

Cmpd	Functional	$\delta_{iso(expt)}$	$\delta_{iso(calc)}$	σ_{iso}	σ^d	σ^p	σ^{so}
**2**	BP86-SR		283.0	54.6	967.9	−913.3	-
**2**	BP86-SOR		291.8	58.6	967.9	−897.9	−11.4
**2**	B3LYP-HF20-SR		272.8	70.6	967.9	−897.3	-
**2**	B3LYP-HF20-SOR		271.6	71.8	967.9	−883.6	−12.6
**2**	B3LYP-HF30-SR		238.8	92.2	968.2	−876.0	-
**2**	B3LYP-HF30-SOR		252.6	91.3	968.2	−863.2	−13.7
**2**	B3LYP-HF40-SR		217.9	113.4	968.5	−855.0	-
**2**	B3LYP-HF40-SOR		234.2	110.0	968.4	−843.2	−15.2
**2**	B3LYP-HF50-SR	198.8 (sol)	197.5	134.2	968.7	−834.5	-
**2**	***B3LYP-HF50-SOR***	211.8 (ss)	***216.6***	***127.9***	***968.7***	***−823.7***	***−17.1***
**2'**	B3LYP-HF50-SR		191.3	140.4	969.2	−828.7	-
**2'**	***B3LYP-HF50-SOR***		***209.0***	***135.5***	***969.2***	***−817.9***	***−15.8***
**2**	PBE0-HF25-SR		235.9	117.1	967.7	−850.6	-
**2**	PBE0-HF25-SOR		248.7	117.2	967.7	−838.0	−12.5
**2**	PBE0-HF40-SR		215.2	142.6	967.6	−825.0	-
**2**	PBE0-HF40-SOR		231.7	139.1	967.6	−813.7	−14.8
**2**	SAOP-SR		193.7	163.7	974.9	−811.2	-
**2**	SAOP-SOR		206.4	163.8	974.9	−801.7	−9.3

Table S3. Computed Scalar Relativistic (SR) and Spin-Orbit Relativistic (SOR) ^{31}P Isotropic Chemical Shift (δ_{iso}), Isotropic Shielding (σ_{iso}), Diamagnetic Shielding (σ^d), Paramagnetic Shielding (σ^p), and Spin-Orbit Shielding (σ^{so}) Values for 3 and 3' Computed With Various Functionals.

Cmpd	Functional	$\delta_{iso(expt)}$	$\delta_{iso(calc)}$	σ_{iso}	σ^d	σ^p	σ^{so}
**3**	BP86-SR		174.6	163.0	966.3	−803.4	-
**3**	BP86-SOR		256.5	93.9	966.3	−788.5	−83.8
**3**	B3LYP-HF20-SR		121.0	209.6	967.2	−757.7	-
**3**	B3LYP-HF20-SOR		216.1	127.3	967.2	−745.9	−94.1
**3**	B3LYP-HF30-SR		89.8	241.2	967.7	−726.5	-
**3**	B3LYP-HF30-SOR		191.1	152.8	967.7	−716.1	−98.9
**3**	B3LYP-HF40-SR		61.3	270.0	968.1	−698.1	-
**3**	B3LYP-HF40-SOR		168.4	175.8	968.1	−688.8	−103.5
**3**	B3LYP-HF50-SR	145.7 (sol)	35.3	296.4	968.5	−672.1	-
**3**	***B3LYP-HF50-SOR***	151.8 (ss)	***147.8***	***196.8***	***968.5***	***−663.7***	***−108.0***
**3'**	B3LYP-HF50-SR		40.7	291.8	969.5	−677.7	-
**3'**	***B3LYP-HF50-SOR***		***167.5***	***177.0***	***969.5***	***−670.0***	***−122.5***
**3**	PBE0-HF25-SR		83.8	269.2	967.4	−698.2	-
**3**	PBE0-HF25-SOR		181.3	184.6	967.4	−687.9	−95.0
**3**	PBE0-HF40-SR		47.3	310.5	967.7	−657.2	-
**3**	PBE0-HF40-SOR		154.9	215.9	967.7	−648.5	−103.3
**3**	SAOP-SR		71.0	286.4	930.2	−643.8	-
**3**	SAOP-SOR		151.1	219.1	930.2	−635.6	−75.5

Table S4. Computed Scalar Relativistic (SR) and Spin-Orbit Relativistic (SOR) ^{31}P Isotropic Chemical Shift (δ_{iso}), Isotropic Shielding (σ_{iso}), Diamagnetic Shielding (σ^d), Paramagnetic Shielding (σ^p), and Spin-Orbit Shielding (σ^{so}) Values for 4 and 4' Computed With Various Functionals.

Cmpd	Functional	$\delta_{iso(expt)}$	$\delta_{iso(calc)}$	σ_{iso}	σ^d	σ^p	σ^{so}
**4**	BP86-SR		557.9	−220.3	969.9	−1190.2	-
**4**	BP86-SOR		668.7	−318.3	969.8	−1161.4	−126.7
**4**	B3LYP-HF20-SR		498.8	−168.2	970.6	−1138.8	-
**4**	B3LYP-HF20-SOR		625.4	−282.0	970.6	−1113.4	−139.2
**4**	B3LYP-HF30-SR		455.4	−124.4	971.5	−1095.9	-
**4**	B3LYP-HF30-SOR		588.9	−245.0	971.5	−1072.2	−144.4
**4**	B3LYP-HF40-SR		413.8	−82.5	972.4	−1054.9	-
**4**	B3LYP-HF40-SOR		553.6	−209.4	972.4	−1032.5	−149.3
**4**	B3LYP-HF50-SR	553.3 (sol)	373.6	−41.9	973.3	−1015.1	-
**4**	***B3LYP-HF50-SOR***	554.8 (ss)	***519.2***	***−174.7***	***973.3***	***−993.9***	***−154.1***
**4'**	B3LYP-HF50-SR		387.3	−55.6	974.8	−1030.4	-
**4'**	***B3LYP-HF50-SOR***		***551.1***	***−206.5***	***974.9***	***−1008.9***	***−172.5***
**4**	PBE0-HF25-SR		437.3	−84.3	971.1	−1055.4	-
**4**	PBE0-HF25-SOR		567.7	−201.8	971.1	−1032.9	−140.0
**4**	PBE0-HF40-SR		386.9	−29.1	971.8	−1001.0	-
**4**	PBE0-HF40-SOR		528.9	−158.1	971.8	−980.5	−149.4
**4**	SAOP-SR		398.0	−40.6	932.3	−972.9	-
**4**	SAOP-SOR		513.1	−142.9	932.3	−955.3	−119.8

Table S5. Computed bond orders, charges, and NLMO data for 1'-4'.[a]

	NLMO Th-P σ-component				NLMO Th-P π-component			
**Entry**	**%Th**	**%P**	**Th 7s/7p/6d/5f**	**P 32/3p**	**%Th**	**%P**	**Th 72/7p/6d/5f**	**P 3s/3p**
**1'**	12	88	27/1/51/21	37/63	-	-	-	-
**2'**	16	84	13/0/66/21	43/57	18	82	0/0/78/22	0/100
**3'**	12	88	12/2/65/21	43/57	8	92	0/0/74/26	0/100
	12	88	12/2/65/21	43/57	8	92	0/0/74/26	0/100
**4'**	15	85	15/3/64/18	49/51	19[b]	81	0/0/77/33	0/100
	15	85	15/3/64/18	49/51	19[b]	81	0/0/77/33	0/100

[a] Calculations at the B3LYP-HF50 TZ2P all-electron ZORA spin-orbit (SOR) level in a benzene solvent continuum. [b] 3-Centre bond, the 19% is made up of 2 × 9.5% contributions from 2 × Th atoms.

Table S6. Mayer Bond Order Obtained Using B3LYP Method and Solution-State ^{31}P data (for the P atom highlighted in red) for Selected Thorium- and Transition-Metal-Phosphorus Complexes.

**Complex**	**Mayer Bond Order**	**^{31}P (ppm)**	**Ref.**[a]
[Th^{IV}(Tren^{TIPS})PH$_2$] (**1**)	0.63	-144.08	[52a]
[Th^{IV}(Tren^{TIPS})(PH)][Na(12C4)$_2$] (**2**)	1.25	198.81	[52a]
[{Th^{IV}(Tren^{TIPS})}$_2$(μ-PH)] (**3**)	0.71	145.71	[52a]
[{Th^{IV}(Tren^{TIPS})}$_2$(μ-P)][Na(12C4)$_2$] (**4**)	1.27	553.50	[52a]
[Th^{IV}(Cp^{ttt})$_2$(PMes*)] (**5**)	1.80	145.70	[56]
[Th^{IV}(Cp^{tt})$_2$(PMes*(μ-Cl)K(18C6)] (**6**)	1.66	133.5	[59]
[Th^{IV}(Cp^{tt})$_2$(PMes*)(ClK)]$_2$ (**7**)	1.56	108.8	[59]
[Th^{IV}(Cp^{tt})$_2$(PTrip)(ClK)]$_2$ (**8**)	1.49	100.6	[60]
[Th^{IV}(Cp*)$_2$(PTrip)(PHTrip)][K(2,2,2-cryptand)] (**9**)	1.57	177.85	[54]
[Th^{IV}(Cp*)$_2$(PTrip)(PHTrip)][K(2,2,2-cryptand)] (**9**)	0.67	-106.99	[54]
[Th^{IV}(Cp*)$_2$(μ-PTrip)(μ-PHTrip)K]$_2$ (**10**)	1.28	171.91	[54]
[Th^{IV}(Cp*)$_2$(μ-PTrip)(μ-PHTrip)K]$_2$ (**10**)	0.69	-110.54	[54]
[{Th^{IV}(Cp*)$_2$}$_2${μ-P[(2,6-CH$_2$CHCH$_3$)$_2$-4-^{i}PrC$_6$H$_2$]}] (**11**)	0.85	161.9	[50]
[Th^{IV}(Cp*)$_2$(PHTrip)$_2$] (**12**)	0.85	1.66	[50]
[{Th^{IV}(Cp*)$_2$}$_2$[μ-P[2,6-(CH$_2$)$_2$-4-(CH$_3$)C$_6$H$_2$)] (**13**)	0.91	206.6	[67]
[Th^{IV}(Cp*)$_2$(μ-PMes)(μ-PHMes)Na]$_2$ (**14**)	1.38	228.99	[70]
[Th^{IV}(Cp*)$_2$(μ-PMes)(μ-PHMes)Na]$_2$ (**14**)	0.66	-74.77	[70]
[Th^{IV}(Cp*)$_2$(μ-PMes)(μ-PHMes)K]$_2$ (**15**)	1.23	229.49	[70]
[Th^{IV}(Cp*)$_2$(μ-PMes)(μ-PHMes)K]$_2$ (**15**)	0.73	-80.52	[70]
[Th^{IV}(Cp*)$_2$(μ-PMes)(μ-PHMes)Rb]$_2$ (**16**)	1.32	237.16	[70]
[Th^{IV}(Cp*)$_2$(μ-PMes)(μ-PHMes)Rb]$_2$ (**16**)	0.72	-77.99	[70]
[Th^{IV}(Cp*)$_2$(μ-PMes)(μ-PHMes)Cs]$_2$ (**17**)	1.31	247.49	[70]
[Th^{IV}(Cp*)$_2$(μ-PMes)(μ-PHMes)Cs]$_2$ (**17**)	0.71	-75.16	[70]
[Th^{IV}(Cp*)$_2${P(Mes)(CH$_3$)}$_2$] (**18**)	0.96	117.5	[55]
[Th^{IV}(Cp*)$_2$(PHMes)$_2$] (**19**)	0.84	15.37	[51]
[Sc^{III}(PNP1)(μ-PDmp)(μ-Br)Li] (**20**)	1.31	9.8	[89]
[Sc^{III}(PNP1)(μ-PDmp)(μ-Br)Li(DME)] (**21**)	1.33	56.1	[89]
[Sc^{III}(L1){P(NHB^{Dipp})}{(μ-Cl)K(18C6)}] (**22**)	1.31	19.6 (-30 °C)	[89]
[Sc^{III}(L1){PH(NHB^{Dipp})}(Cl)] (**23**)	0.77	-149.6	[90]
[Ti^{IV}(^{Me}Nacnac)(PMes*)(CH$_2$^{t}Bu)] (**24**)	1.79	242/216	[91]
[Ti^{IV}(^{tBu}Nacnac)(PTrip)(CH$_2$^{t}Bu)] (**25**)	1.85	157	[73]

$[Ti^{IV}(^{tBu}Nacnac)(PTrip)(Me)]$ (**26**)	1.86	232	[73]
$[Ti^{IV}(^{tBu}Nacnac)(PTrip)\{CH_3B(C_6F_5)_3\}]$ (**27**)	2.02	207	[73]
$[Ti^{IV}(PNP1)(PTrip)(CH_2{}^tBu)]$ (**28**)	1.61	237	[92]
$[Ti^{IV}Cp_2(PDmp)(PMe_3)]$ (**29**)	1.46	1067.3	[93]
$[Ti^{IV}Cp_2(PDmp)(PMe_3)]$ (**29**)	0.58	8.0	[94]
$[Zr^{IV}Cp_2(PP^tBu_2)(PPhMe_2)]$ (**30**)	1.49	728.2	[94]
$[Zr^{IV}Cp_2(PP^tBu_2)(PPhMe_2)]$ (**30**)	0.58	6.5	[94]
$[Zr^{IV}(Tren^{DMBS})(PH)][K(B15C5)_2]$ (**31**)	1.39	246.75	[95]
$[Zr^{IV}(Tren^{DMBS})(PH_2)]$ (**32**)	0.77	-175.28	[95]
$[Mo^{II}(Cl)(L2)_2\}(\mu\text{-}P)Mo^{II}(L2)_2\}]$ (**33**)	1.52	1161.00	[96]
$[Mo^{II}(L2)_2\}(\mu\text{-}P)Mo^{II}(L2)_2\}][BAr^F{}_4]$ (**34**)	1.50	1123.27	[96]
$[W(ArO)_2(\mu\text{-}P)\{(\mu\text{-}POAr_2)(\mu\text{-}P)C^tBu\}]$ (**35**)	1.43	831.8	[97]
$[W^{VI}(Tren^{TMS})(\mu\text{-}P)GaCl_3)]$ (**36**)	1.97	366	[98]
$[W^{VI}(Tren^{TMS})(\mu\text{-}P)(AuCl)]$ (**37**)	2.23	567	[99]
$[\{W^{VI}(Tren^{TMS})(P)\}_2(\mu\text{-}Au)][Al\{OC(CF_3)_3\}_4]$ (**38**)	2.22	660	[99]
$[Nb^V(NAr'CH_2Bu^t)_3(P)][Na(12C4)]$ (**39**)	2.58	1110.2	[100]
$[Mo^{VI}(NAr'{}^tBu)_3(P)]$ (**40**)	2.44	1216	[76]
$[Mo^{VI}(NAr'Ad)_3(P)]$ (**41**)	2.47	1215	[101]
$[Mo^{VI}(NAr'{}^iPr)_3(P)]$ (**42**)	2.49	1256	[102]
$[Mo^{VI}(OCy^{Me})_3(P)]$ (**43**)	2.50	1130	[103]
$[Mo^{IV}(L2)_2(P)(Cl)]$ (**44**)	2.63	1300.73	[104]
$[W^{VI}(Tren^{TMS})(P)]$ (**45**)	2.56	1080	[75]
$[W^{VI}(NAr'{}^iPr)_3(P)]$ (**46**)	2.61	1021	[105]
$[W^{VI}(ODipp)_3(P)][Na(12C4)_2]$ (**47**)	2.66	886	[106]
$[Re^V(PyrPz)(PNP2)(P)]$ (**48**)	2.45	1069.4	[107]
$[Mo^{IV}(Cp)_2(PMes^*)]$ (**49**)	1.46	799.5	[108]
$[Th(Bn)_4(dmpe)]$ (**50**)	0.34	-23.6	[109]
$[Ir(Cp^*)(I^iPr_2Me_2)(PMes^*)]$ (**51**)	1.63	560.0	[110]
$[Ru(p\text{-}cymene)(PCy_3)(PMes^*)]$ (**52**)	1.63	811.4	[111]
$[Ru(p\text{-}cymene)(PCy_3)(PMes^*)]$ (**52**)	0.73	35.2	[111]
$[Os(p\text{-}cymene)(PPh_3)(PMes^*)]$ (**53**)	1.72	667.5	[112]
$[Os(p\text{-}cymene)(PPh_3)(PMes^*)]$ (**53**)	0.99	18.8	[112]
$[Os(PNP2)(Cl)(PCl)]$ (**54**)	2.05	858.4	[113]
$[Os(PNP2)(Cl)(PCl)]$ (**54**)	0.85	70.4	[113]
$[Ni(DTBPE)(PDmp)]$ (**55**)	1.52	969.6	[114]
$[Ni(DTBPE)(PDmp)]$ (**55**)	0.79	107.5	[114]
$[Rh(Cp^*)(I^iPr_2Me_2)(PMes^*)]$ (**56**)	1.45	745.9	[115]
$[Ir(PPh_3)(Cp^*)(PMes^*)]$ (**57**)	1.63	651.8	[116]
$[Ir(PPh_3)(Cp^*)(PMes^*)]$ (**57**)	1.04	27.9	[116]
$[Ir(CO)(Cp^*)(PMes^*)]$ (**58**)	1.58	804.6	[116]
$[Ru(L3)(P^tBu)][Na(1,4\text{-}dioxane)_2]_2$ (**59**)	1.85	1047	[85]
$[Ru(L3)(PNMe_2)][Na(1,4\text{-}dioxane)_2]_2$ (**60**)	1.75	791	[85]
$[Co(Cp)(PPh_3)(PMes^*)]$ (**61**)	1.60	866.9	[117]
$[Co(Cp)(PPh_3)(PMes^*)]$ (**61**)	0.80	54.0	[117]

[a] The reference number refers to the reference number in the paper.
Abbreviations:

$Tren^{TMS}$ = $[N(CH_2CH_2NSiMe_3)_3]^{3-}$
$Tren^{DMBS}$ = $[N(CH_2CH_2NSiMe_2Bu^t)_3]^{3-}$
$Tren^{TIPS}$ = $[N(CH_2CH_2NSi^iPr_3)_3]^{3-}$
12C4 = 12-crown-4
B15C5 = Benzyl 15-crown-5
Cp^{ttt} = 1,2,4-$(Me_3C)_3C_5H_2$
Cp^{tt} = 1,3-$(Me_3C)_2C_5H_3$
Cp^* = C_5Me_5
Mes = 2,4,6-$Me_3C_6H_2$
Mes* = 2,4,6-$(Me_3C)_3C_6H_2$
Trip = 2,4,6-$^iPr_3C_6H_2$
PNP1 = $N[2-P(^iPr)_2$-4-methylphenyl$]_2^-$
Dmp = 2,6-$Mes_2C_6H_3$
Dipp = 2,6-$^iPr_2C_6H_3$
L1 = $[MeC(NDipp)CHC(Me)NCH_2CH_2NMe_2]^-$
NHB^{Dipp} = B{N(Dipp)CHCHN(Dipp)}
^{Me}Nacnac = DippNC(Me)CHC(Me)NDipp
$^{Bu^t}$Nacnac = DippNC(Bu^t)CHC(Bu^t)NDipp
L2 = 1,4-$(2-P^iPr_2-C_6H_4)_2C_6H_4$
BAr^F_4 = Tetrakis-[3,5-bis(trifluoromethyl)phenyl]-borate
Ar = 2,6-$Me_2C_6H_3$
Ar' = 3,5-$Me_2C_6H_3$
PNP2 = $N[CH_2CH_2PBu^t_2]_2$
PyrPz = 3-(pyrazol-3-yl)pyridine
p-cymene = 4-methyl-iso-propylbenzene
DTBPE = 1,2-bis(di-tert-butylphosphino)ethane
L3 = octamethylcalix[4]pyrrole
$I^iPr_2Me_2$ = 1,3-diisopropyl-4,5-dimethyl-4,5-dihydroimidazol-2-ylidene

Table S7. Coordinates and energy for 1.

1.C	-1.354877	-1.001821	-6.219692
2.C	1.441017	1.021092	-5.026533
3.C	-1.704932	-1.088033	-4.745792
4.C	-2.793923	0.061431	-4.451610
5.C	0.915808	-2.268917	-4.034254
6.C	0.259277	-3.614356	-3.902854
7.C	0.716814	0.731732	-3.715881
8.C	-0.207172	2.003900	-3.436424
9.C	2.205570	-2.179940	-3.190132
10.C	-1.841690	-1.895395	-1.551078
11.C	0.049111	4.947710	-0.807039
12.C	-2.909694	-1.303374	-0.679266
13.C	-2.421799	5.012121	-0.357970
14.C	3.751140	-3.204100	0.118573
15.C	-1.037102	4.930170	0.271631
16.C	0.469618	-4.569724	0.263031
17.C	-2.616462	1.680567	0.352549
18.C	2.953044	-2.041895	0.733987
19.C	-3.071869	0.415193	1.058781
20.C	3.981449	-1.032742	1.336460
21.C	1.111117	-4.309742	1.672218
22.C	-2.114270	-1.777809	1.596628
23.C	0.978921	3.371696	1.945088
24.C	1.486813	4.687416	2.539610
25.C	-0.901254	-1.437787	2.440498
26.C	0.230252	-4.975290	2.710051

27.C	-2.020632	3.699623	2.891393
28.C	1.404137	2.195826	2.843901
29.C	-2.082465	5.143970	3.397468
30.C	2.080463	-2.444681	3.771800
31.C	3.316553	-3.307230	4.035282
32.C	-1.808889	2.747638	4.070659
33.C	2.246889	-1.058684	4.365029
34.H	-2.246696	-1.159689	-6.848227
35.H	-0.609184	-1.750946	-6.525432
36.H	-0.950240	-0.014743	-6.482578
37.H	0.731775	1.208953	-5.845079
38.H	-3.751416	-0.173914	-4.942538
39.H	2.095777	0.196355	-5.343698
40.H	2.072003	1.917880	-4.929023
41.H	1.193379	-2.106137	-5.091804
42.H	-0.625224	-3.722522	-4.548582
43.H	-2.170876	-2.061917	-4.529836
44.H	-0.872492	2.182371	-4.289886
45.H	-2.444673	1.017575	-4.858249
46.H	0.945413	-4.437834	-4.172419
47.H	-2.983513	0.194193	-3.378482
48.H	2.906786	-2.996669	-3.432683
49.H	2.743321	-1.232490	-3.329947
50.H	-0.066468	-3.812268	-2.869272
51.H	0.413628	2.902452	-3.307243
52.H	1.476584	0.680052	-2.911058
53.H	-0.843870	1.900717	-2.543000
54.H	-2.329405	-2.312945	-2.448253
55.H	1.973409	-2.273494	-2.116761
56.H	-0.093069	5.796127	-1.498238
57.H	2.138485	2.769675	-1.208523
58.H	0.021909	4.031660	-1.413698
59.H	-2.529468	5.927530	-0.964453
60.H	-3.405504	-0.484316	-1.219244
61.H	-1.384334	-2.762507	-1.042828
62.H	-2.607137	4.163858	-1.034225
63.H	4.500030	-2.817269	-0.588857
64.H	-2.810202	1.578636	-0.734185
65.H	1.060454	5.035812	-0.388795
66.H	-3.686827	-2.043981	-0.409719
67.H	0.999413	-4.064042	-0.555876
68.H	3.125843	-3.924473	-0.423173
69.H	0.448493	-5.646682	0.032343
70.H	2.487416	-1.511281	-0.127918
71.H	-3.231481	5.024843	0.388269
72.H	-0.571377	-4.216539	0.250486
73.H	3.189683	2.021147	0.437921
74.H	4.657953	-0.671567	0.547970
75.H	-0.899024	5.825656	0.904676
76.H	-3.272002	2.508221	0.672221

77.H	4.296109	-3.760480	0.896238
78.H	-4.157215	0.230087	0.935436
79.H	1.503234	3.231083	0.982747
80.H	-1.936838	-2.737824	1.094616
81.H	2.087473	-4.828715	1.673356
82.H	3.499541	-0.152349	1.781323
83.H	1.242122	5.558977	1.916814
84.H	4.593544	-1.513159	2.110408
85.H	-2.872672	0.527179	2.133113
86.H	-3.037487	-1.876293	2.199960
87.H	0.074373	-6.040060	2.468131
88.H	-2.361833	5.856292	2.609438
89.H	-3.025155	3.476623	2.481638
90.H	2.583028	4.666508	2.652668
91.H	1.051427	1.211299	2.489980
92.H	-0.770252	-4.516988	2.758777
93.H	2.502051	2.132365	2.908569
94.H	-1.078118	-0.485968	2.976908
95.H	-0.793103	-2.198976	3.232534
96.H	1.067937	4.871588	3.540935
97.H	4.216465	-2.894365	3.558004
98.H	0.654690	-4.937691	3.723651
99.H	-1.118935	5.474988	3.812342
100.H	3.198445	-4.340081	3.676553
101.H	-1.692844	1.699119	3.758601
102.H	1.021956	2.306059	3.868127
103.H	-2.828234	5.241710	4.204157
104.H	3.114373	-0.529984	3.946951
105.H	1.228936	-2.923518	4.289501
106.H	1.365870	-0.424537	4.194960
107.H	-0.908218	3.015883	4.641976
108.H	-2.656167	2.791543	4.774920
109.H	3.533016	-3.366536	5.115727
110.H	2.404853	-1.110063	5.456602
111.N	-0.796697	-0.883331	-1.904611
112.N	-2.287703	-0.729521	0.550594
113.N	-1.196594	1.930545	0.624968
114.N	0.295324	-1.341561	1.573968
115.P	2.746290	1.530430	-0.828645
116.Si	-0.314429	-0.808530	-3.566919
117.Si	-0.833435	3.425648	1.437370
118.Si	1.574815	-2.480957	1.897883
119.Th	0.147360	0.210836	-0.105016

Energy: –860.88081356 eV

Table S8. Coordinates and energy for 2.

1.C	0.098044	2.710424	-4.602852
2.C	1.286681	4.881293	-4.081250
3.C	0.876208	-3.626420	-3.941596
4.C	3.898438	2.345617	-3.547209

5.C	0.595703	3.648636	-3.526053
6.C	2.384030	0.331294	-3.338569
7.C	2.862251	1.619728	-2.664698
8.C	1.249536	-4.106488	-2.543646
9.C	2.674880	-3.655040	-2.212941
10.C	-1.217187	-1.322677	-2.332622
11.C	-1.363425	-6.005998	-1.937627
12.C	-1.595731	-4.525833	-1.622794
13.C	-2.477739	-0.735774	-1.705578
14.C	-2.056274	1.669981	-1.506678
15.C	2.380316	4.174902	-1.066147
16.C	-0.968283	2.533409	-0.890345
17.C	-2.695286	-4.427712	-0.568165
18.C	1.395595	5.164149	-0.445884
19.C	3.299061	3.584298	0.028517
20.C	0.732741	-3.941699	0.456625
21.C	-2.985805	0.441148	0.390191
22.C	1.054263	-5.432270	0.636078
23.C	-2.395553	-0.356925	1.538429
24.C	-0.085648	-3.471472	1.659039
25.C	0.703935	2.958832	2.727125
26.C	-2.845538	2.811374	3.138502
27.C	0.708585	1.707867	3.630952
28.C	-2.276858	1.834859	4.175997
29.C	-1.786698	-1.822886	4.589150
30.C	-0.741684	-0.732122	4.788835
31.C	0.660685	-1.364917	4.832782
32.C	-3.423666	1.205179	4.964302
33.C	1.075323	2.147858	5.066779
34.H	0.919863	2.349999	-5.242193
35.H	-0.626862	3.207659	-5.272742
36.H	0.655643	5.387872	-4.834824
37.H	2.238281	4.638528	-4.580632
38.H	1.592849	-3.994542	-4.697346
39.H	3.469306	2.645535	-4.516861
40.H	-0.125574	-3.960431	-4.258266
41.H	-0.393173	1.818933	-4.187754
42.H	1.869482	0.520858	-4.293271
43.H	0.890581	-2.527458	-3.997538
44.H	4.755265	1.685900	-3.766219
45.H	3.236619	-0.335033	-3.550434
46.H	1.513040	5.618616	-3.299811
47.H	4.298358	3.250578	-3.067182
48.H	-0.317562	4.040039	-3.032925
49.H	3.370010	-3.908012	-3.033173
50.H	-1.523866	-2.082064	-3.075939
51.H	-0.678908	-6.158195	-2.783705
52.H	-0.719591	-0.531547	-2.929522
53.H	1.241210	-5.212111	-2.550162
54.H	1.695969	-0.247432	-2.702833

55.H	-1.991275	-4.064895	-2.548779
56.H	-2.314258	-6.505916	-2.197041
57.H	-1.800907	1.480754	-2.557927
58.H	-3.201982	-0.388084	-2.471574
59.H	2.735948	-2.567438	-2.052550
60.H	3.020526	4.735273	-1.773387
61.H	3.367218	1.300139	-1.732000
62.H	-3.048797	2.167677	-1.485038
63.H	3.062488	-4.126809	-1.299166
64.H	-0.944657	-6.546642	-1.076617
65.H	0.749511	5.653477	-1.192186
66.H	-1.052292	3.553226	-1.306451
67.H	-2.968698	-1.518415	-1.112745
68.H	-3.652931	-4.827083	-0.946917
69.H	4.131404	3.005697	-0.393474
70.H	1.628154	-5.852284	-0.203186
71.H	-2.876820	-3.398576	-0.227616
72.H	1.922489	5.965556	0.102241
73.H	-2.438661	-5.017594	0.325145
74.H	0.736016	4.665762	0.279553
75.H	-1.191815	2.653136	0.188192
76.H	-4.011053	0.099228	0.138155
77.H	3.730938	4.388148	0.650288
78.H	1.684876	-3.377035	0.484271
79.H	2.753681	2.903769	0.694740
80.H	0.136571	-6.031777	0.742337
81.H	-3.056690	1.491800	0.698651
82.H	1.648512	-5.593958	1.551402
83.H	2.614045	0.689506	1.194879
84.H	-2.367460	-1.427248	1.247809
85.H	-1.066859	-3.967567	1.710437
86.H	-0.273783	-2.386219	1.677821
87.H	0.479342	2.727451	1.675368
88.H	-3.115156	-0.316150	2.378244
89.H	0.444941	-3.696840	2.597984
90.H	-2.078616	3.245459	2.484732
91.H	-3.582268	2.310288	2.493137
92.H	1.687617	3.454305	2.742713
93.H	-0.035015	3.699946	3.070744
94.H	1.511043	1.036700	3.267217
95.H	-3.377919	3.646112	3.628583
96.H	-1.659919	-2.320021	3.617215
97.H	0.991768	-1.700939	3.838577
98.H	-4.074305	0.587648	4.323641
99.H	-2.819353	-1.447452	4.632623
100.H	-1.711197	2.447863	4.905929
101.H	2.065916	2.631800	5.083029
102.H	-4.070345	1.983474	5.408598
103.H	1.428795	-0.667833	5.192656
104.H	-1.692883	-2.605509	5.362845

105.H	0.353498	2.884468	5.457500
106.H	0.671145	-2.242565	5.503053
107.H	1.107016	1.310899	5.777900
108.H	-3.072220	0.567158	5.786814
109.H	-0.924925	-0.276619	5.783540
110.N	-0.313357	-1.851846	-1.291432
111.N	0.377325	1.925454	-1.095132
112.N	-2.107049	0.374193	-0.794259
113.N	-1.046053	0.133420	1.911726
114.P	3.054810	-0.666501	0.850679
115.Si	1.495029	2.801647	-2.068787
116.Si	-0.012824	-3.538898	-1.240093
117.Si	-0.879621	0.691048	3.537131
118.Th	0.458960	-0.108246	0.106926

Energy: −857.98871763 eV

Table S9. Coordinates and energy for 3.

1.C	-0.000761	1.032082	-6.219798
2.C	-0.806187	-1.293458	-6.063177
3.C	1.595578	-0.803871	-6.054147
4.C	-2.158954	-4.804145	-5.622481
5.C	-3.503705	2.652499	-5.407343
6.C	-2.981072	5.075664	-5.126662
7.C	-2.382216	3.664683	-5.115809
8.C	0.485850	2.055121	-5.228738
9.C	4.694173	1.611357	-4.981334
10.C	-1.880043	-1.293810	-5.010873
11.C	2.148051	-1.640826	-4.910445
12.C	0.008095	-4.969011	-4.651883
13.C	4.964578	0.144121	-4.614234
14.C	6.475972	-0.067294	-4.449095
15.C	-1.461092	-4.683038	-4.272174
16.C	0.549017	5.370131	-4.200252
17.C	4.908086	-3.205512	-3.620713
18.C	-0.399958	4.831493	-3.113801
19.C	-3.749434	-3.303685	-3.064881
20.C	-4.381816	-4.636076	-2.743451
21.C	4.763744	-2.096141	-2.572241
22.C	-3.079423	1.376064	-2.249663
23.C	-4.143174	-2.283324	-2.051193
24.C	-2.350304	2.725908	-2.108919
25.C	3.030461	1.983291	-2.006963
26.C	0.353348	4.691899	-1.796565
27.C	-3.351268	3.806629	-1.678760
28.C	3.814976	0.692462	-1.742025
29.C	0.548441	-3.320327	-1.540518
30.C	-0.950655	-3.578588	-1.513697
31.C	4.132279	-2.666753	-1.310265
32.C	5.192946	1.040225	-1.169971
33.C	-1.426290	-4.875837	-0.852170

34.C	-5.131741	-0.387180	0.687016
35.C	1.864038	5.081681	1.432075
36.C	-3.738079	-0.290440	1.325288
37.C	-3.382901	1.194237	1.559714
38.C	3.001502	-4.072482	1.705856
39.C	-2.190173	4.576486	1.779034
40.C	-4.977795	-3.760628	1.957010
41.C	1.240224	3.745962	1.866276
42.C	4.626628	-0.467815	2.029455
43.C	2.346988	2.712626	2.004734
44.C	2.148152	-2.856357	2.078070
45.C	-3.579047	-3.189596	2.221023
46.C	0.687016	-3.275677	2.160459
47.C	-1.347090	5.100679	2.920529
48.C	-2.790924	-4.196984	3.064356
49.C	4.543275	-1.353710	3.248917
50.C	-2.306950	5.496395	4.054410
51.C	-5.030993	-1.127350	3.984925
52.C	5.262275	-0.656595	4.403475
53.C	-5.283954	0.322221	4.408523
54.C	1.084417	6.214457	4.821874
55.C	0.963017	4.694259	4.851440
56.C	-1.288943	2.445830	4.744893
57.C	-1.756583	-1.592114	4.880884
58.C	4.160830	-4.008297	5.175480
59.C	-5.127041	-2.047288	5.217594
60.C	2.932220	-3.091285	5.129449
61.C	2.328758	4.096780	5.185322
62.C	1.938723	0.043265	5.226894
63.C	1.694136	-3.946654	5.421506
64.C	-0.657639	1.741062	5.920377
65.C	-1.618971	-0.510756	5.930201
66.C	0.796603	-0.211118	6.178225
67.H	0.474010	1.151456	-7.213320
68.H	-1.205246	-1.061799	-7.069775
69.H	1.568053	-1.360840	-7.011199
70.H	-1.985412	-5.797025	-6.069541
71.H	-3.944016	2.828242	-6.402874
72.H	-1.083613	1.159505	-6.355818
73.H	-3.532128	5.257109	-6.064913
74.H	2.250734	0.066320	-6.194291
75.H	-0.357854	-2.294650	-6.109488
76.H	5.183996	1.876732	-5.933095
77.H	-1.772924	-4.066812	-6.344180
78.H	-1.681331	3.617829	-5.971028
79.H	-3.251869	-4.663595	-5.582309
80.H	0.382995	3.059702	-5.671201
81.H	7.018605	0.290382	-5.340351
82.H	4.659855	-0.463187	-5.488672
83.H	-3.159954	1.608544	-5.377773

84.H	0.079651	-5.905134	-5.229809
85.H	-2.700472	-1.951385	-5.350203
86.H	-2.219552	5.862640	-5.040390
87.H	0.418675	-4.172551	-5.290112
88.H	0.056738	5.500197	-5.175647
89.H	3.109042	-2.077506	-5.231611
90.H	3.623232	1.834538	-5.088837
91.H	1.571060	1.926007	-5.064781
92.H	-2.335022	-0.285725	-4.953182
93.H	-4.321610	2.746727	-4.677927
94.H	1.484980	-2.507002	-4.739572
95.H	6.745371	-1.122022	-4.307238
96.H	5.349775	-2.856487	-4.565827
97.H	-3.696440	5.222510	-4.303734
98.H	5.095659	2.293665	-4.217605
99.H	1.407581	4.697643	-4.346373
100.H	0.959708	6.351299	-3.909527
101.H	-4.081149	-2.978780	-4.066555
102.H	3.934619	-3.659982	-3.857063
103.H	0.680952	-5.075620	-3.788859
104.H	-1.792754	-5.555899	-3.674932
105.H	6.874597	0.491069	-3.588901
106.H	-4.083574	-5.436198	-3.434946
107.H	5.551385	-4.018213	-3.243184
108.H	-5.481946	-4.562017	-2.790325
109.H	-1.192478	5.589057	-2.969983
110.H	-3.960505	1.461730	-2.900829
111.H	3.597699	2.683716	-2.635978
112.H	-2.464170	0.572818	-2.690795
113.H	-4.130351	3.956532	-2.441434
114.H	5.787026	-1.765356	-2.310601
115.H	1.019463	-3.479810	-2.519560
116.H	2.066138	1.841872	-2.525265
117.H	-3.710324	-1.292736	-2.244491
118.H	-5.238044	-2.142801	-1.991686
119.H	-4.138181	-4.973772	-1.727337
120.H	5.790694	1.622101	-1.888237
121.H	1.116186	3.902802	-1.850977
122.H	0.873661	5.628016	-1.535095
123.H	-2.871595	4.779394	-1.501592
124.H	-1.271040	-5.765121	-1.483353
125.H	3.103849	-3.000898	-1.498047
126.H	-3.416840	1.000933	-1.275024
127.H	-1.618559	2.602199	-1.288237
128.H	-3.822708	-2.579795	-1.039337
129.H	4.696404	-3.541176	-0.945424
130.H	2.799125	2.499878	-1.063961
131.H	5.776545	0.148905	-0.900941
132.H	-0.311660	4.448978	-0.956444
133.H	1.073406	-3.981295	-0.834061

134.H	0.788486	-2.296766	-1.200784
135.H	3.250856	0.150220	-0.958007
136.H	-3.868674	3.528114	-0.747191
137.H	-2.493848	-4.830923	-0.605111
138.H	-1.331076	-2.797469	-0.817435
139.H	4.096571	-1.938222	-0.491166
140.H	5.101874	1.654176	-0.259783
141.H	-5.199060	0.241132	-0.215150
142.H	-0.888824	-5.061920	0.091684
143.H	-0.028765	1.480641	-0.099204
144.H	-5.386775	-1.408527	0.383521
145.H	2.448028	4.977505	0.502648
146.H	-2.998740	-0.668652	0.592773
147.H	-3.429378	1.765324	0.621964
148.H	2.674789	-4.509519	0.748930
149.H	-1.597705	4.308335	0.892269
150.H	1.108805	5.859862	1.251911
151.H	0.576972	3.408777	1.046967
152.H	2.792938	2.464076	1.030645
153.H	-5.588254	-3.138677	1.289711
154.H	-5.919871	-0.038976	1.373016
155.H	-4.910106	-4.760398	1.495486
156.H	4.174792	-0.919133	1.136942
157.H	-3.056615	-3.122397	1.246297
158.H	-2.937473	5.320248	1.453383
159.H	0.344900	-3.715950	1.213259
160.H	2.231378	-2.120256	1.256131
161.H	4.067136	-3.827598	1.600331
162.H	5.673431	-0.227742	1.779172
163.H	2.555281	5.464629	2.198499
164.H	-2.368248	1.365762	1.951357
165.H	-2.743734	3.677270	2.079682
166.H	-4.067996	1.674901	2.270995
167.H	4.109508	0.488955	2.197150
168.H	2.917841	-4.867814	2.462144
169.H	-0.841491	6.025430	2.585643
170.H	-2.730013	-5.171284	2.551386
171.H	-0.023530	-2.457289	2.372106
172.H	2.038208	1.748764	2.447674
173.H	-5.544059	-3.884265	2.892853
174.H	3.157223	3.074877	2.654656
175.H	5.106064	-2.282529	3.030617
176.H	0.520661	-4.026209	2.944709
177.H	-5.868525	-1.417897	3.322208
178.H	-1.765610	-3.864960	3.264507
179.H	-2.926588	6.357377	3.751492
180.H	-5.474139	0.984456	3.554155
181.H	-3.275738	-4.384695	4.035312
182.H	1.742800	6.556192	4.008996
183.H	6.319799	-0.471695	4.150419

184.H	-3.008256	4.681973	4.287068
185.H	4.136079	-4.761427	4.373976
186.H	3.088884	4.399111	4.449023
187.H	0.120337	6.722936	4.689844
188.H	4.814875	0.326501	4.614163
189.H	-2.270532	1.981003	4.525449
190.H	-1.802938	5.781041	4.989735
191.H	1.604874	-4.775578	4.703463
192.H	-4.920771	-3.101543	4.985879
193.H	-6.164751	0.390128	5.069524
194.H	-4.433351	0.743477	4.966699
195.H	5.109204	-3.462463	5.079672
196.H	-0.833450	-2.201857	4.884012
197.H	-1.542378	3.469589	5.062394
198.H	5.251468	-1.232664	5.340610
199.H	-2.549099	-2.293478	5.185596
200.H	2.045940	1.131507	5.068151
201.H	2.327384	2.998569	5.211018
202.H	-6.138377	-2.001272	5.655657
203.H	1.522860	6.589586	5.762275
204.H	0.754135	-3.378422	5.379578
205.H	0.304686	4.465506	5.713205
206.H	2.882441	-0.260029	5.712010
207.H	4.197097	-4.559207	6.129970
208.H	-4.431151	-1.738906	6.011734
209.H	3.040172	-2.389915	5.979542
210.H	2.685280	4.448109	6.168537
211.H	-2.515343	0.123281	5.912744
212.H	1.758045	-4.404014	6.423114
213.H	0.316975	2.203833	6.125231
214.H	0.683827	-1.294901	6.312710
215.H	-1.264110	1.830742	6.842830
216.H	-1.512848	-0.921581	6.952021
217.H	0.969217	0.232439	7.178502
218.N	0.248766	-0.329651	-5.683249
219.N	-0.258874	1.919451	-3.928818
220.N	-1.322399	-1.708799	-3.701675
221.N	2.270472	-0.812345	-3.690661
222.N	-1.994748	-1.006210	3.537239
223.N	-0.389318	2.394629	3.574210
224.N	1.696486	-0.656487	3.948388
225.N	-0.439023	0.322621	5.574131
226.P	0.086551	0.039543	-0.042711
227.Si	-1.319676	3.237242	-3.595420
228.Si	3.901261	-0.518813	-3.178937
229.Si	-1.938338	-3.200695	-3.069178
230.Si	-3.529561	-1.386823	2.841779
231.Si	0.076156	3.915349	3.359612
232.Si	2.791276	-1.955324	3.619103
233.Th	0.192143	-0.127075	-2.934193

234.Th	-0.142502	0.214305	2.840629

Energy: −1698.40509217 eV

Table S10. Coordinates and energy for 4.

1.C	4.628357	-0.017055	-6.429483
2.C	-3.970221	-1.066748	-5.862718
3.C	1.347914	1.296053	-5.249799
4.C	4.714282	0.120225	-4.900599
5.C	3.384532	-3.146882	-4.871550
6.C	2.467455	-1.929651	-4.853646
7.C	-2.944112	-1.271693	-4.760325
8.C	5.184659	1.539469	-4.547012
9.C	-2.113261	0.003078	-4.608922
10.C	1.074274	-2.352539	-4.357862
11.C	-6.365823	-2.462592	-3.827372
12.C	1.825263	0.870278	-3.860927
13.C	-5.026979	-3.067611	-3.365384
14.C	-1.827662	-4.196792	-3.206921
15.C	2.127827	2.112551	-3.014471
16.C	-5.309376	-4.022764	-2.195869
17.C	-1.694366	2.692151	-2.467793
18.C	-4.942237	0.294815	-2.311998
19.C	-2.101316	-2.923495	-2.377404
20.C	4.805067	-1.661558	-2.113007
21.C	-0.761723	4.964982	-2.049373
22.C	-4.472410	5.055105	-1.779473
23.C	5.934459	-0.920435	-1.469844
24.C	-6.042344	0.151601	-1.251349
25.C	-1.217218	3.656497	-1.381403
26.C	-2.192077	-3.253163	-0.886982
27.C	-3.612285	5.408545	-0.571343
28.C	1.613221	-3.554153	-0.804328
29.C	2.500845	4.713212	-0.382439
30.C	5.141445	1.914145	-0.184423
31.C	6.101151	0.820935	0.246669
32.C	-5.728326	2.036623	0.257847
33.C	-4.468116	6.129653	0.486944
34.C	4.431617	-5.293079	0.508492
35.C	3.523801	4.724098	0.745158
36.C	1.331236	-3.802936	0.688656
37.C	5.830858	-1.493344	0.923705
38.C	0.786820	-5.225358	0.872721
39.C	-6.064189	-0.210384	1.143382
40.C	-4.585054	2.607920	1.066724
41.C	-5.161028	-1.437887	1.345189
42.C	-0.914436	5.986309	1.432029
43.C	3.121645	5.937236	1.654430
44.C	-1.517593	4.576208	1.520815
45.C	3.967134	-4.827171	1.901148
46.C	4.718002	-1.483258	1.945400

47.C	6.122060	3.909854	2.583815
48.C	5.173223	-4.717846	2.826168
49.C	1.860802	2.530448	2.475942
50.C	-2.284043	4.451767	2.845276
51.C	-1.861631	-3.858637	2.802649
52.C	4.762118	3.357572	3.034852
53.C	-4.293367	-4.190806	3.248124
54.C	1.877651	1.069752	2.971166
55.C	-3.072652	-3.310460	3.568390
56.C	1.434589	3.466985	3.611453
57.C	2.009188	-3.186769	3.614492
58.C	-1.734318	0.927716	3.525111
59.C	-1.685472	-0.603313	3.687216
60.C	5.035230	2.194210	3.985171
61.C	1.655975	-4.481590	4.332759
62.C	-4.709432	-0.992294	4.581196
63.C	2.759950	-2.274598	4.567508
64.C	-4.872761	0.500756	4.838234
65.C	-1.081262	-0.994659	5.031345
66.C	-4.642807	-1.714198	5.939756
67.H	5.577147	0.289017	-6.904438
68.H	3.838261	0.621917	-6.852712
69.H	4.420971	-1.047722	-6.749465
70.H	-3.479430	-0.753111	-6.801477
71.H	-4.544588	-1.976841	-6.090615
72.H	2.353365	-1.585200	-5.899120
73.H	2.128590	1.853381	-5.792006
74.H	1.056774	0.441936	-5.878240
75.H	-4.692800	-0.273274	-5.612191
76.H	2.970083	-3.946494	-5.510297
77.H	-1.606067	0.261013	-5.553744
78.H	4.394420	-2.922364	-5.249702
79.H	6.184427	1.744559	-4.968113
80.H	0.471218	1.961028	-5.182438
81.H	-2.260781	-2.069956	-5.108746
82.H	4.503452	2.298668	-4.960151
83.H	-6.265975	-1.788497	-4.687431
84.H	0.715355	-3.236193	-4.911832
85.H	5.515617	-0.569564	-4.572157
86.H	-7.068271	-3.264279	-4.115702
87.H	0.328170	-1.557904	-4.484659
88.H	-4.658413	-3.692079	-4.204655
89.H	-2.753032	0.854654	-4.336588
90.H	-1.712408	-3.986053	-4.280199
91.H	3.498786	-3.573851	-3.863942
92.H	-1.347628	-0.079368	-3.824858
93.H	5.239859	1.708650	-3.462038
94.H	2.855666	2.774659	-3.506384
95.H	-6.851688	-1.896697	-3.019155
96.H	1.093871	-2.617058	-3.292631

97.H	-5.370181	0.083302	-3.308643
98.H	0.983406	0.360631	-3.351086
99.H	-2.635402	-4.938007	-3.103603
100.H	-1.024454	2.722436	-3.343182
101.H	5.167327	-2.076047	-3.071971
102.H	-0.898716	-4.685872	-2.872368
103.H	1.213583	2.695001	-2.832580
104.H	-2.709933	2.908610	-2.828282
105.H	-6.129483	-4.718677	-2.447930
106.H	0.069805	4.776796	-2.748927
107.H	-3.882277	4.661291	-2.617923
108.H	-1.573486	5.417478	-2.640459
109.H	-5.019503	5.939121	-2.154488
110.H	-4.624618	1.355600	-2.353866
111.H	-1.219254	-2.266369	-2.494924
112.H	-4.438909	-4.630015	-1.919384
113.H	6.160901	-0.027128	-2.069693
114.H	2.548476	1.886150	-2.021546
115.H	-1.655114	1.641606	-2.128926
116.H	-5.229671	4.296705	-1.532826
117.H	-6.968622	0.702123	-1.521673
118.H	6.861001	-1.528771	-1.418782
119.H	4.566835	-2.554710	-1.500602
120.H	-5.624352	-3.472956	-1.294893
121.H	-0.413843	5.725370	-1.338099
122.H	-6.298553	-0.913335	-1.177383
123.H	0.896332	-4.112179	-1.427807
124.H	5.003564	1.847843	-1.281906
125.H	-2.870411	6.155587	-0.912571
126.H	2.705797	3.936302	-1.129651
127.H	2.622288	-3.841824	-1.128435
128.H	2.465398	5.680512	-0.914760
129.H	1.439781	-2.493754	-1.064967
130.H	-0.345511	3.180977	-0.895201
131.H	-5.747712	2.536923	-0.720003
132.H	-1.568025	-4.126494	-0.638682
133.H	-3.207052	-3.469603	-0.529949
134.H	7.112341	0.951260	-0.193232
135.H	-4.910380	7.048640	0.061903
136.H	3.593843	-5.560792	-0.147772
137.H	-1.768027	-2.430706	-0.278997
138.H	5.616867	2.898843	-0.020735
139.H	5.012917	-4.512320	-0.006612
140.H	1.489644	4.523798	0.004703
141.H	4.535060	4.886956	0.341816
142.H	5.084838	-6.180238	0.587749
143.H	-0.072787	-5.400837	0.205864
144.H	5.861964	-2.482089	0.444226
145.H	-5.149875	-2.021145	0.404215
146.H	1.538604	-5.992176	0.625901

147.H	-6.710362	2.220183	0.742486
148.H	-0.205170	6.100620	0.602461
149.H	-5.310410	5.511997	0.833017
150.H	-7.119105	-0.491404	0.945542
151.H	3.207512	6.881289	1.089729
152.H	-3.890990	6.425529	1.372152
153.H	0.519416	-3.100275	0.957038
154.H	6.210119	0.870959	1.339063
155.H	6.825741	-1.308621	1.376961
156.H	-4.821840	3.650952	1.329389
157.H	-1.691410	6.756727	1.308048
158.H	6.038758	4.794127	1.936284
159.H	-0.678697	3.857245	1.577452
160.H	0.441461	-5.425303	1.894650
161.H	2.075281	5.848317	1.970610
162.H	-6.041978	0.387295	2.063824
163.H	6.704192	3.153280	2.035802
164.H	-1.992852	-3.742967	1.718562
165.H	-4.532822	2.075334	2.036200
166.H	1.084085	2.579168	1.688634
167.H	-5.649350	-2.099631	2.083535
168.H	3.333846	-5.640969	2.306447
169.H	-0.369047	6.236613	2.358717
170.H	-4.524588	-4.168994	2.172809
171.H	3.743610	6.035794	2.556292
172.H	5.931630	-4.025805	2.431549
173.H	4.659438	-0.464948	2.378417
174.H	5.673793	-5.696909	2.935616
175.H	1.330765	0.413385	2.263487
176.H	5.001284	-2.125575	2.795409
177.H	-3.112577	5.173797	2.911484
178.H	-2.234136	1.256640	2.600049
179.H	-2.707400	3.450564	2.988082
180.H	6.730518	4.202435	3.457449
181.H	-1.724939	-4.934420	3.004948
182.H	-0.927472	-3.350888	3.074221
183.H	2.887030	0.664859	3.121901
184.H	-1.010562	-0.970575	2.887235
185.H	1.317082	4.509705	3.288993
186.H	-4.096205	-5.244164	3.512302
187.H	5.471877	1.332089	3.456715
188.H	4.276325	4.153927	3.630420
189.H	4.910198	-4.375892	3.838076
190.H	-5.201324	-3.882977	3.789075
191.H	-1.617570	4.654437	3.700672
192.H	-0.724400	1.355313	3.495404
193.H	1.056537	-2.666924	3.390085
194.H	1.027350	-5.156961	3.738204
195.H	-4.876061	1.098362	3.915356
196.H	0.469443	3.150610	4.037614

197.H	1.355477	0.969697	3.934464
198.H	-5.638490	-1.339840	4.089817
199.H	2.980764	-1.297097	4.122119
200.H	2.561195	-5.045086	4.613340
201.H	4.129874	1.835270	4.493397
202.H	2.163120	3.462367	4.438209
203.H	-2.275573	1.404992	4.354513
204.H	5.755518	2.481605	4.772522
205.H	-2.858835	-3.436125	4.647087
206.H	3.716825	-2.713498	4.895698
207.H	1.110688	-4.276256	5.270898
208.H	-5.814738	0.715156	5.373756
209.H	-0.072069	-0.569274	5.157379
210.H	-0.989143	-2.083626	5.151606
211.H	-4.057922	0.888270	5.468167
212.H	2.169938	-2.094086	5.483011
213.H	-4.651920	-2.808360	5.842071
214.H	-1.687708	-0.622984	5.872832
215.H	-5.503273	-1.434907	6.573183
216.H	-3.734271	-1.442319	6.498510
217.N	3.612740	-0.797101	-2.267586
218.N	-3.815187	-0.597241	-1.952036
219.N	5.529223	-0.474542	-0.112279
220.N	-5.520281	0.597870	0.033756
221.N	-3.284710	2.482967	0.314938
222.N	3.845180	1.770016	0.521531
223.N	3.430068	-1.869168	1.328700
224.N	-3.802408	-1.028320	1.734047
225.P	-0.011343	-0.016959	-0.174359
226.Si	3.166881	-0.444656	-3.908477
227.Si	-3.537682	-1.895976	-3.059803
228.Si	-2.474413	3.973085	-0.002515
229.Si	3.480902	3.032138	1.649291
230.Si	2.744646	-3.360409	1.864861
231.Si	-3.327275	-1.457498	3.328349
232.Th	2.722093	-0.202728	-0.164004
233.Th	-2.732552	0.206525	-0.007975

Energy: −1695.95786058 eV

Table S11. Coordinates and energy for 1'.

1.C	0.640277	3.870484	-3.343239
2.C	3.133847	2.566712	-2.689730
3.C	-0.142188	0.795573	-2.625535
4.C	1.663812	-3.824333	-2.296138
5.C	-1.580516	0.579527	-2.243930
6.C	-1.586417	-1.735121	-1.453048
7.C	1.770901	3.834330	-0.282929
8.C	-0.881706	-2.517906	-0.366753
9.C	-2.833601	-0.010051	-0.224474
10.C	3.372146	-2.334425	0.011592

11.C	1.484040	-4.814125	0.370019
12.C	-2.496298	1.073452	0.754658
13.C	-3.327579	-0.506297	3.585582
14.C	-1.914200	2.180776	4.173873
15.C	-0.213135	-0.324045	4.146992
16.H	0.794338	3.298708	-4.268011
17.H	1.159450	4.831882	-3.471778
18.H	3.008116	1.938924	-3.584355
19.H	-0.114025	1.401643	-3.545848
20.H	-0.432963	4.088554	-3.246447
21.H	3.632395	3.495002	-3.007581
22.H	1.828793	-3.070792	-3.079790
23.H	-2.174032	0.157125	-3.077541
24.H	0.320803	-0.169703	-2.900249
25.H	2.395843	-4.630366	-2.450009
26.H	0.666422	-4.259914	-2.455019
27.H	-1.001550	-1.814833	-2.378591
28.H	3.819435	2.041078	-2.008810
29.H	-2.024156	1.548629	-1.976769
30.H	-2.602080	-2.122581	-1.661055
31.H	-3.699221	0.255976	-0.861255
32.H	2.410244	4.712495	-0.453307
33.H	-0.905023	-3.588359	-0.638868
34.H	3.563543	-1.402557	-0.542528
35.H	4.239980	-2.986041	-0.163158
36.H	2.293750	-5.514532	0.115794
37.H	0.545936	-5.275247	0.029852
38.H	0.862637	4.181168	0.230656
39.H	2.315292	3.168663	0.402955
40.H	-3.100967	-0.918552	0.332133
41.H	-2.239302	2.004725	0.218334
42.H	-1.460836	-2.451626	0.572818
43.H	3.352028	-2.094544	1.084107
44.H	1.440982	-4.743036	1.466457
45.H	-3.393391	1.320911	1.347525
46.H	3.648399	1.482123	1.262897
47.H	2.171087	2.206107	2.546683
48.H	-4.184722	-0.003082	3.116632
49.H	-3.253981	-1.519594	3.166332
50.H	-2.724463	2.774515	3.726771
51.H	0.002805	-1.331111	3.759293
52.H	-0.987029	2.765732	4.097176
53.H	0.697160	0.282497	4.033758
54.H	-3.553792	-0.602613	4.657285
55.H	-2.147475	2.055862	5.241642
56.H	-0.390471	-0.431968	5.227292
57.N	0.609707	1.446220	-1.505632
58.N	-1.647072	-0.306705	-1.058016
59.N	0.498082	-2.012278	-0.178522
60.N	-1.346249	0.637543	1.628119

61.P	2.782742	0.938011	2.267892
62.Si	1.436242	2.934779	-1.892684
63.Si	1.763554	-3.168803	-0.509640
64.Si	-1.700044	0.488396	3.329890
65.Th	0.546988	0.209920	0.434365

Energy: –472.91802636 eV

Table S12. Coordinates and energy for 2'.

1.C	1.498764	-2.145600	-4.339579
2.C	-1.168955	-3.323134	-3.556166
3.C	-1.099172	-0.182353	-2.532988
4.C	1.362618	-3.589516	-1.657861
5.C	2.696861	3.000690	-1.604728
6.C	-2.306290	-0.157640	-1.599591
7.C	0.209155	4.887710	-1.118228
8.C	-2.023331	1.846990	-0.217562
9.C	-0.927649	2.407583	0.670351
10.C	-2.617341	-0.275953	0.834916
11.C	2.254491	4.308683	1.116208
12.C	-1.831380	-1.463487	1.355330
13.C	0.247702	-3.226704	3.806918
14.C	1.371402	-0.379569	3.970268
15.C	-1.520871	-0.915792	4.728849
16.H	1.019317	-1.461297	-5.056581
17.H	1.748190	-3.072880	-4.878917
18.H	-1.682747	-2.662535	-4.270278
19.H	-0.900528	-4.241098	-4.101300
20.H	2.437007	-1.677755	-4.008728
21.H	-1.445568	-0.495714	-3.537175
22.H	-1.891983	-3.599283	-2.774038
23.H	-0.737634	0.856629	-2.669060
24.H	2.286362	2.732888	-2.589616
25.H	1.855383	-4.410208	-2.199697
26.H	3.438062	3.801203	-1.749241
27.H	-0.352250	4.510669	-1.985766
28.H	-3.148934	0.432509	-2.015493
29.H	0.804447	5.750030	-1.456109
30.H	-2.651985	-1.191414	-1.460291
31.H	2.150075	-2.998759	-1.157286
32.H	3.218812	2.112726	-1.206455
33.H	-1.886044	2.250917	-1.229944
34.H	0.730031	-4.034726	-0.875677
35.H	-0.516331	5.264437	-0.382457
36.H	-3.036725	2.139174	0.130813
37.H	-3.642029	-0.564212	0.522091
38.H	2.849259	5.191162	0.833313
39.H	-1.768505	-2.223536	0.549185
40.H	-1.125874	3.483635	0.834271
41.H	3.061567	0.401013	1.056652
42.H	2.937708	3.557830	1.538461

43.H	-2.715143	0.450950	1.654461
44.H	1.555295	4.612829	1.910072
45.H	-1.012255	1.946158	1.673497
46.H	-2.437590	-1.946060	2.147732
47.H	1.114409	-3.526370	3.200514
48.H	2.210273	-0.574696	3.281828
49.H	-0.594416	-3.881886	3.535260
50.H	1.156321	0.699114	3.943598
51.H	-2.462918	-1.465823	4.583613
52.H	0.487399	-3.412751	4.865188
53.H	-1.746244	0.159717	4.681114
54.H	1.707592	-0.627896	4.988803
55.H	-1.165643	-1.138818	5.746435
56.N	-0.036566	-1.049795	-1.989531
57.N	-1.897072	0.377745	-0.276865
58.N	0.419647	2.158599	0.079104
59.N	-0.481913	-1.068111	1.824981
60.P	3.493480	-0.706425	0.194788
61.Si	0.380249	-2.477969	-2.839682
62.Si	1.341091	3.538750	-0.382244
63.Si	-0.144895	-1.395418	3.486292
64.Th	0.790931	-0.174584	0.046770

Energy: –469.90047324 eV

Table S13. Coordinates and energy for 3'.

1.C	3.839208	1.364925	-5.330840
2.C	1.811940	3.376143	-4.294505
3.C	1.153990	0.400453	-4.015071
4.C	-3.916962	2.322689	-3.549111
5.C	-4.386797	-4.524006	-2.186080
6.C	4.484489	2.373173	-2.204554
7.C	-1.735384	-3.036894	-2.280634
8.C	5.703042	1.477896	-2.039085
9.C	5.072306	-1.607088	-1.624741
10.C	-5.589061	3.492121	-1.315820
11.C	3.132702	-4.616541	-1.241347
12.C	-5.510175	0.206356	-1.176256
13.C	6.105409	-0.698162	-1.014719
14.C	-2.457385	3.209621	-1.009732
15.C	-2.291014	-5.329407	-0.189893
16.C	-6.303851	0.110739	0.102458
17.C	6.026025	1.329779	0.386193
18.C	5.365365	-3.906896	0.710687
19.C	-5.827497	-2.136280	0.948810
20.C	-4.568729	-2.922878	1.220412
21.C	2.368608	-3.137632	1.303476
22.C	5.126634	1.001855	1.545539
23.C	1.505816	3.278176	1.694591
24.C	4.292416	4.344562	2.127317
25.C	-5.739595	-0.212711	2.461119
26.C	-4.696169	0.845509	2.745268

27.C	-0.890377	-0.751486	3.853646
28.C	3.420440	2.364061	4.105708
29.C	-1.838811	2.043647	4.575091
30.C	-3.364716	-0.406268	5.643673
31.H	3.369501	1.450691	-6.321827
32.H	4.647987	2.108543	-5.293699
33.H	4.293774	0.366215	-5.266304
34.H	1.365207	3.418553	-5.298813
35.H	0.573093	0.496733	-4.943857
36.H	2.569385	4.171396	-4.240174
37.H	-4.799314	1.846640	-4.001283
38.H	-3.822684	3.323955	-3.995115
39.H	1.523232	-0.635375	-3.973333
40.H	-3.034751	1.739052	-3.846926
41.H	1.024587	3.614940	-3.566126
42.H	0.461432	0.537634	-3.170653
43.H	4.658905	3.060946	-3.052266
44.H	-3.964980	-5.274252	-2.871490
45.H	5.762732	0.814715	-2.911842
46.H	-1.384199	-3.821374	-2.967469
47.H	-4.875695	-3.751302	-2.794668
48.H	-2.207682	-2.257544	-2.894880
49.H	4.765850	-1.215068	-2.611011
50.H	6.646858	2.052554	-1.986783
51.H	3.842957	-4.752631	-2.069656
52.H	-5.506768	4.473821	-1.805466
53.H	-6.117001	0.727060	-1.939675
54.H	-6.502224	3.018882	-1.703418
55.H	5.533170	-2.586915	-1.840193
56.H	-5.157308	-5.024688	-1.583530
57.H	7.028381	-0.642410	-1.621698
58.H	2.161530	-4.344170	-1.677520
59.H	-0.857487	-2.594253	-1.793296
60.H	-5.356109	-0.808571	-1.587279
61.H	4.394090	3.032147	-1.321903
62.H	-2.252374	4.167946	-1.507399
63.H	-1.600406	2.547300	-1.205475
64.H	-1.978740	-6.120333	-0.887550
65.H	3.017921	-5.591588	-0.745034
66.H	-5.733381	3.672617	-0.241036
67.H	6.381172	-1.095771	-0.028524
68.H	-7.311439	-0.319303	-0.052698
69.H	6.129915	-4.016531	-0.071095
70.H	-6.147592	-2.344725	-0.081143
71.H	0.110601	-1.445517	-0.186755
72.H	-2.495865	3.406721	0.071488
73.H	5.995425	2.414568	0.218152
74.H	-3.072383	-5.753967	0.456729
75.H	-6.432027	1.122494	0.509568
76.H	7.078876	1.051056	0.578850

77.H	-1.432757	-5.079050	0.447739
78.H	1.642393	3.662647	0.672837
79.H	5.237742	-4.896900	1.173202
80.H	4.276247	4.707630	1.089808
81.H	-4.815493	-4.001038	1.227032
82.H	1.427914	-2.778660	0.862690
83.H	5.762616	-3.232281	1.482053
84.H	-6.658637	-2.422393	1.620954
85.H	5.144306	-0.089630	1.728418
86.H	2.156862	-4.110230	1.771337
87.H	0.752886	2.477583	1.661233
88.H	-4.212881	-2.710566	2.246242
89.H	2.637637	-2.444983	2.114560
90.H	-4.832551	1.685612	2.038979
91.H	5.340671	4.167208	2.410044
92.H	5.560373	1.442769	2.461290
93.H	1.095348	4.103980	2.292536
94.H	-6.771748	0.157755	2.597906
95.H	3.915878	5.154623	2.769885
96.H	-5.585926	-1.053076	3.150876
97.H	-0.321038	-0.401950	2.978584
98.H	-4.898485	1.285305	3.739795
99.H	-1.125464	-1.814728	3.693757
100.H	-1.224660	2.445741	3.757532
101.H	4.487979	2.195973	4.308345
102.H	2.875522	1.483667	4.475167
103.H	3.117886	3.221163	4.725473
104.H	-2.669264	2.744295	4.744805
105.H	-0.225985	-0.698071	4.728722
106.H	-1.224303	2.035256	5.487428
107.H	-3.675414	-1.449528	5.491702
108.H	-4.255043	0.177834	5.915773
109.H	-2.686461	-0.385173	6.509418
110.N	3.270708	1.546791	-2.386396
111.N	5.521819	0.654337	-0.828611
112.N	-4.220437	0.880390	-0.920948
113.N	3.881746	-1.731286	-0.713727
114.N	-3.529475	-2.584562	0.226474
115.N	-5.532944	-0.694583	1.068747
116.N	3.749155	1.483391	1.285049
117.N	-3.324874	0.283331	2.650888
118.P	-0.040974	-0.015258	-0.063568
119.Si	2.549847	1.660568	-3.959585
120.Si	-4.058055	2.432389	-1.668545
121.Si	-2.966355	-3.829150	-1.100463
122.Si	3.711016	-3.292982	-0.003111
123.Si	3.184267	2.733118	2.345387
124.Si	-2.435950	0.287234	4.132098
125.Th	2.807964	0.311939	-0.483157
126.Th	-2.865247	-0.381857	0.470988

Energy: −923.85732019 eV

Table S14. Coordinates and energy for 4'.

1.C	0.037792	-2.087362	-5.391632
2.C	-1.553374	-4.535072	-4.646235
3.C	-3.284115	-1.782932	-4.094439
4.C	-4.850869	2.935136	-3.709032
5.C	-4.495579	-2.485219	-3.460130
6.C	4.283541	-0.825629	-2.896813
7.C	5.623177	1.935856	-2.697990
8.C	2.555434	1.699058	-2.698272
9.C	0.457772	-3.191414	-2.585964
10.C	-1.987302	2.838175	-2.548431
11.C	-5.681655	-0.705425	-2.323561
12.C	-5.287619	0.383927	-1.316702
13.C	-5.281930	-2.815456	-1.175704
14.C	-4.272976	3.719595	-0.783254
15.C	-4.120848	-3.461377	-0.452941
16.C	5.287834	0.397887	0.346633
17.C	5.755582	1.536883	1.222025
18.C	0.415645	5.581839	1.284496
19.C	3.553007	3.830832	1.656341
20.C	1.952739	-2.846191	2.051876
21.C	-1.920586	-4.012800	2.435747
22.C	4.576454	3.230204	2.567522
23.C	-4.881236	-3.287432	2.843216
24.C	-2.728119	-1.090541	2.844736
25.C	-0.492722	3.171832	2.898259
26.C	4.989998	0.984308	3.457178
27.C	4.397961	-2.946677	3.902528
28.C	1.674096	5.178582	4.024226
29.C	3.665599	0.522759	4.035879
30.C	1.907428	-2.102244	5.216959
31.H	-0.665084	-1.839750	-6.201586
32.H	0.808923	-2.748522	-5.815926
33.H	-2.298098	-4.324985	-5.427739
34.H	-0.764433	-5.149101	-5.106764
35.H	0.529107	-1.153880	-5.082559
36.H	-3.149838	-2.182439	-5.118806
37.H	-4.540078	2.344053	-4.582283
38.H	-5.388155	-2.463758	-4.117309
39.H	-4.697480	3.997909	-3.949259
40.H	-3.533588	-0.714670	-4.237174
41.H	-2.039751	-5.143751	-3.870219
42.H	4.338776	-0.755116	-3.993679
43.H	5.602343	1.974886	-3.797697
44.H	-5.930269	2.782903	-3.560709
45.H	2.483655	1.641718	-3.794579
46.H	-4.226282	-3.535184	-3.285578
47.H	-5.553437	-0.286673	-3.330087

48.H	-1.642323	2.321223	-3.456203
49.H	1.388229	-3.604835	-3.001162
50.H	-1.819501	3.914937	-2.699278
51.H	5.170271	-1.379329	-2.553313
52.H	6.600049	1.524420	-2.404208
53.H	3.396593	-1.419705	-2.636848
54.H	5.564306	2.969476	-2.327344
55.H	-6.742572	-1.015646	-2.219181
56.H	2.499695	2.761090	-2.416916
57.H	1.672253	1.193286	-2.272745
58.H	0.703893	-2.239619	-2.085187
59.H	-5.936805	-3.567469	-1.662501
60.H	0.105151	-3.884855	-1.808607
61.H	-1.346061	2.504818	-1.716163
62.H	-6.038581	1.197089	-1.351188
63.H	-4.157241	4.758627	-1.127064
64.H	-3.473975	-3.978017	-1.186876
65.H	-5.306118	3.602447	-0.423235
66.H	-5.889347	-2.279140	-0.433171
67.H	-5.358299	-0.027608	-0.291495
68.H	6.106403	0.124892	-0.348895
69.H	-3.600810	3.567815	0.072652
70.H	-4.515590	-4.262605	0.198389
71.H	-0.034256	5.125471	0.391571
72.H	5.850203	2.436963	0.598827
73.H	3.897117	3.724067	0.608575
74.H	5.148157	-0.501464	0.976954
75.H	1.240730	6.228211	0.949720
76.H	2.686784	-2.958979	1.239374
77.H	6.741990	1.339999	1.686386
78.H	-0.342327	6.228137	1.752790
79.H	3.529191	4.925297	1.825462
80.H	-2.140177	-4.987431	1.973773
81.H	1.109732	-2.257920	1.653138
82.H	-0.951252	-3.670786	2.048753
83.H	5.556256	3.744873	2.498364
84.H	-0.748074	2.533215	2.035401
85.H	-5.239902	-4.262060	2.481615
86.H	1.588633	-3.852700	2.301951
87.H	-5.684133	-2.551234	2.693732
88.H	-1.840398	-0.672393	2.340145
89.H	-3.530521	-0.339554	2.793741
90.H	5.072633	-3.029792	3.037852
91.H	5.562592	0.091966	3.169028
92.H	-1.374748	3.784122	3.136505
93.H	4.223940	3.325745	3.604231
94.H	-1.817467	-4.172440	3.519829
95.H	2.543006	5.799647	3.761859
96.H	-4.714030	-3.382779	3.926789
97.H	-0.309786	2.507630	3.755550

98.H	-2.476981	-1.240440	3.905391
99.H	5.598589	1.549465	4.193017
100.H	4.134807	-3.968072	4.218123
101.H	0.909704	5.850236	4.443825
102.H	3.064835	1.411893	4.308400
103.H	4.960789	-2.486710	4.728745
104.H	1.981595	4.483240	4.818413
105.H	3.855084	0.005583	4.996153
106.H	0.919225	-1.622348	5.208578
107.H	1.780355	-3.144186	5.546567
108.H	2.522628	-1.592865	5.973314
109.N	-2.087349	-1.945284	-3.254086
110.N	-4.786306	-1.843652	-2.162354
111.N	-3.914765	0.846052	-1.628612
112.N	4.038929	0.753455	-0.361768
113.N	-3.338325	-2.434339	0.324587
114.N	2.227701	3.197300	1.843469
115.N	4.727602	1.784314	2.263297
116.N	2.944755	-0.328663	3.058667
117.P	-0.081381	0.116610	0.026418
118.Si	-0.831687	-2.903065	-3.930617
119.Si	-3.807230	2.487806	-2.161508
120.Si	4.149354	0.890762	-2.079323
121.Si	-3.243464	-2.716440	2.024363
122.Si	0.997827	4.240113	2.488063
123.Si	2.784412	-1.991366	3.513841
124.Th	-2.352657	-0.862823	-1.141751
125.Th	2.270060	0.935585	1.169356

Energy: –921.01300304 eV

Table S15. Coordinates and energy for 5

1.C	-2.323400	-0.342189	-5.093301
2.C	-0.071473	-0.370758	-4.132811
3.C	-1.543639	-0.592379	-3.787927
4.C	-5.380481	0.973914	-3.379650
5.C	-1.658041	-2.079389	-3.371264
6.C	-4.866659	-1.368861	-2.723875
7.C	-2.029895	0.416223	-2.720543
8.C	-4.739442	0.093861	-2.292405
9.C	2.756785	1.692782	-2.390088
10.C	-1.218024	1.550872	-2.416727
11.C	-3.308934	0.640781	-2.063925
12.C	-0.345499	4.383191	-1.946852
13.C	7.917328	-1.448316	-1.412352
14.C	4.886524	2.478084	-1.494673
15.C	-1.925935	2.451722	-1.589014
16.C	-3.178414	1.878611	-1.365163
17.C	1.447203	-3.432431	-1.233940
18.C	-5.585596	0.250632	-1.021174
19.C	3.622884	1.699254	-1.117152

20.C	-1.402313	3.756847	-1.007967
21.C	-2.534211	4.751846	-0.785791
22.C	5.270132	-0.118222	-0.563840
23.C	3.925630	0.242041	-0.676990
24.C	7.190979	-1.697677	-0.086205
25.C	5.702551	-1.391341	-0.240436
26.C	2.911076	-0.754294	-0.410153
27.C	4.725313	-2.363253	-0.109042
28.C	3.359521	-2.110527	-0.199287
29.C	3.172803	-4.667267	-0.020921
30.C	7.459213	-3.129613	0.354304
31.C	2.416013	-3.327933	-0.046890
32.C	-2.162971	-2.676483	-0.079941
33.C	2.966936	2.494811	0.013965
34.C	-0.732655	3.424702	0.330980
35.C	7.777099	-0.763195	0.997523
36.C	-4.262207	-2.963598	1.239702
37.C	1.642787	-3.243786	1.291180
38.C	-2.770548	-2.694942	1.337151
39.C	-2.467117	-1.356825	1.985196
40.C	-4.536114	2.421047	2.207991
41.C	-2.084992	-3.828191	2.142571
42.C	-3.275009	-0.227730	2.063028
43.C	-1.218519	-0.984712	2.549404
44.C	-2.607427	0.853890	2.730584
45.C	-1.272242	0.361530	3.021213
46.C	-3.470593	2.005793	3.253504
47.C	0.451086	2.259850	3.291267
48.C	-2.746515	3.320586	3.630146
49.C	1.100923	-0.038132	3.818543
50.C	-0.094567	0.943863	3.840622
51.C	-4.209962	1.499080	4.503477
52.C	-0.507645	1.077701	5.327370
53.H	-1.920843	-0.977733	-5.898660
54.H	-2.221765	0.707491	-5.406407
55.H	-3.394378	-0.562227	-5.005579
56.H	0.245898	-1.131237	-4.861628
57.H	0.102681	0.611904	-4.592870
58.H	-4.827149	0.916901	-4.326722
59.H	-1.367466	-2.716266	-4.221654
60.H	-6.417471	0.650739	-3.571268
61.H	-4.389368	-1.569135	-3.687467
62.H	-5.401535	2.028560	-3.069668
63.H	0.571767	-0.463860	-3.245819
64.H	3.280038	1.170671	-3.204907
65.H	-5.934234	-1.614857	-2.835024
66.H	-2.663723	-2.372482	-3.065096
67.H	-0.780195	4.599825	-2.932808
68.H	-0.200526	1.686525	-2.760540
69.H	2.558687	2.726007	-2.718563

70.H	-0.961293	-2.295907	-2.550588
71.H	7.527401	-2.105627	-2.204201
72.H	5.446382	2.000615	-2.312499
73.H	7.794173	-0.410218	-1.751852
74.H	-4.452371	-2.060449	-1.977786
75.H	1.800278	1.185003	-2.213209
76.H	2.002961	-3.614872	-2.166606
77.H	0.512242	3.713854	-2.087947
78.H	4.590818	3.480469	-1.839827
79.H	8.997354	-1.641915	-1.306823
80.H	-3.046766	4.983390	-1.730601
81.H	0.028590	5.325747	-1.518349
82.H	-6.604682	-0.121667	-1.205768
83.H	0.867882	-2.508867	-1.361581
84.H	0.749296	-4.271623	-1.083421
85.H	3.782251	-4.817638	-0.924102
86.H	-5.678910	1.295453	-0.701478
87.H	5.568299	2.620915	-0.643264
88.H	6.023624	0.645643	-0.739167
89.H	-3.985622	2.350395	-0.815135
90.H	7.114743	-3.863303	-0.389690
91.H	-2.141240	5.693466	-0.371399
92.H	-2.674603	-1.926373	-0.716888
93.H	-2.283172	-3.632494	-0.611055
94.H	-5.165499	-0.322943	-0.185102
95.H	2.696201	3.505930	-0.331456
96.H	-3.286014	4.373006	-0.078947
97.H	2.436314	-5.484043	0.020995
98.H	8.541071	-3.281995	0.486475
99.H	5.045409	-3.381853	0.081014
100.H	-1.075579	-2.466103	-0.056226
101.H	0.117713	2.728485	0.184404
102.H	7.654223	0.295230	0.729953
103.H	8.854554	-0.954698	1.128817
104.H	2.064869	1.987975	0.377829
105.H	3.821737	-4.774042	0.860415
106.H	-4.778876	-2.210600	0.628775
107.H	-4.444831	-3.944363	0.774473
108.H	3.662665	2.598575	0.860844
109.H	6.970632	-3.361423	1.311823
110.H	-0.313531	4.306061	0.839613
111.H	-1.459315	2.985000	1.040917
112.H	-4.064206	2.791251	1.288569
113.H	1.044174	-2.325661	1.328112
114.H	7.277382	-0.925207	1.963868
115.H	0.982984	-4.119384	1.396846
116.H	-2.289018	-4.802952	1.672528
117.H	-4.317750	-0.195544	1.762684
118.H	-5.209594	1.598395	1.938780
119.H	-4.731629	-2.969257	2.234379

120.H	2.344736	-3.238393	2.138399
121.H	-0.998951	-3.692274	2.181573
122.H	-5.157978	3.230268	2.618641
123.H	0.893844	2.097117	2.298139
124.H	-0.351981	-1.626567	2.633658
125.H	-2.260746	3.788392	2.764199
126.H	-2.466819	-3.854880	3.173315
127.H	1.441284	-0.232452	2.791168
128.H	-0.301037	3.049803	3.215744
129.H	-3.492657	4.035163	4.010088
130.H	1.256085	2.630872	3.945824
131.H	-4.824908	0.618620	4.267166
132.H	-1.998020	3.184528	4.415803
133.H	1.939129	0.406901	4.375051
134.H	0.856295	-0.993336	4.303497
135.H	-4.871196	2.284750	4.904646
136.H	-3.505718	1.209380	5.295942
137.H	-1.321057	1.795072	5.488364
138.H	-0.837474	0.104697	5.720009
139.H	0.355509	1.412161	5.924667
140.P	1.126350	-0.283587	-0.246922
141.Th	-1.308315	0.334612	0.100488

Energy: –894.08394623 eV

Table S16. Coordinates and energy for 6

1.C	3.486143	2.025116	-5.669864
2.C	2.029191	2.096643	-5.340272
3.C	5.597658	1.488111	-4.783515
4.C	0.957538	-3.238420	-4.185304
5.C	0.482415	3.160615	-3.915981
6.C	6.249513	0.862323	-3.581705
7.C	2.475782	-1.452290	-3.271288
8.C	-0.011799	-1.112001	-3.302020
9.C	1.108263	-2.134112	-3.108036
10.C	0.335023	4.303011	-2.946972
11.C	-2.768126	0.982882	-2.126626
12.C	1.013503	-2.830197	-1.747811
13.C	-4.004141	3.022480	-1.605951
14.C	6.629364	1.167139	-1.279971
15.C	-0.129558	-3.503838	-1.246672
16.C	-3.302743	1.807724	-0.944341
17.C	0.965595	4.951630	-0.769499
18.C	2.085646	-3.112591	-0.868736
19.C	6.612179	2.211816	-0.210719
20.C	-1.787963	-5.721915	-0.223184
21.C	-8.216368	2.661159	0.183227
22.C	-9.003962	0.300022	0.304384
23.C	0.236080	-4.254628	-0.101902
24.C	-5.585423	1.430662	0.041371
25.C	-4.275354	0.954443	-0.081666

26.C	-2.166346	2.386251	-0.100192
27.C	1.605991	-4.012208	0.123265
28.C	1.636038	4.419732	0.466262
29.C	-8.009731	1.310979	0.906210
30.C	-6.581402	0.801451	0.770276
31.C	-0.630587	-5.254278	0.664456
32.C	-3.900756	-0.270256	0.578842
33.C	0.204820	-6.479202	1.063610
34.C	5.155846	3.581990	1.042167
35.C	-4.398233	-3.395591	0.969296
36.C	-6.216252	-0.401288	1.378485
37.C	-4.949865	-0.960367	1.312625
38.C	3.710022	3.800626	1.389269
39.C	4.613345	-0.766467	1.574575
40.C	-8.377672	1.530381	2.384688
41.C	-1.191535	-4.629537	1.944029
42.C	-4.756687	-2.328387	2.022607
43.C	4.324878	-3.188701	2.096258
44.C	-6.036446	-2.833880	2.708533
45.C	3.973880	-1.764165	2.557551
46.C	2.485374	-1.519278	2.692512
47.C	1.905459	-0.237613	2.803316
48.C	-3.703915	-2.257082	3.135913
49.C	1.493137	-2.423948	3.117876
50.C	0.052550	2.134110	3.093232
51.C	0.595393	-0.327466	3.321862
52.C	0.326028	-1.705719	3.505009
53.C	4.588642	-1.561674	3.945752
54.C	-0.176367	0.847666	3.901880
55.C	-1.681599	0.594793	4.024049
56.C	0.394164	1.053886	5.328240
57.H	3.630426	1.345646	-6.532621
58.H	1.461787	2.365669	-6.252119
59.H	3.856365	3.026885	-5.962510
60.H	5.810278	0.868227	-5.674985
61.H	6.001274	2.502166	-4.962784
62.H	1.050693	-2.809130	-5.198066
63.H	1.666681	1.112805	-4.992202
64.H	-0.175986	3.337912	-4.788007
65.H	2.547485	-0.967165	-4.256698
66.H	-0.024997	-3.724762	-4.110417
67.H	1.728722	-4.013991	-4.065928
68.H	0.013794	-0.703583	-4.327540
69.H	7.329628	0.723412	-3.779548
70.H	5.803987	-0.130993	-3.390111
71.H	0.673308	5.250863	-3.408925
72.H	0.167556	2.218537	-3.433506
73.H	3.294205	-2.184850	-3.205991
74.H	-1.001007	-1.567057	-3.157652
75.H	-0.732297	4.408486	-2.676611

76.H	-3.597541	0.668405	-2.779011
77.H	-2.070436	1.590327	-2.728385
78.H	0.075378	-0.279493	-2.591334
79.H	2.622614	-0.690631	-2.493987
80.H	-4.831155	2.722579	-2.265823
81.H	-3.264226	3.551347	-2.227709
82.H	7.676684	0.861517	-1.464844
83.H	-2.236305	0.088406	-1.777527
84.H	-1.116326	-3.486655	-1.700192
85.H	-8.022826	2.579051	-0.896161
86.H	-1.413461	-6.183636	-1.148718
87.H	1.408025	5.919887	-1.074245
88.H	6.065624	0.273507	-0.956244
89.H	-8.800695	0.136034	-0.764589
90.H	-4.387431	3.746396	-0.870944
91.H	3.109690	-2.762504	-0.969461
92.H	7.147311	3.116691	-0.555690
93.H	-0.104392	5.114970	-0.542675
94.H	-2.450226	-4.891369	-0.497509
95.H	-1.479704	2.971276	-0.735372
96.H	-5.839917	2.360802	-0.458358
97.H	-9.255998	2.997866	0.311802
98.H	-10.038776	0.665356	0.407856
99.H	0.641391	-6.973727	0.182993
100.H	-2.399079	-6.467875	0.308830
101.H	-7.557155	3.441688	0.589857
102.H	-5.223004	-3.505273	0.247745
103.H	-8.939060	-0.675476	0.806737
104.H	7.128521	1.827380	0.689219
105.H	5.596283	4.509609	0.630322
106.H	-1.587069	1.581713	0.371764
107.H	-3.493684	-3.108855	0.415754
108.H	-2.567122	3.046290	0.684875
109.H	4.168873	-0.856254	0.573030
110.H	1.189691	3.447982	0.737598
111.H	2.203683	-4.456059	0.911923
112.H	4.015333	-3.372302	1.061764
113.H	1.464207	5.128974	1.298857
114.H	-0.427003	-7.211462	1.591211
115.H	-4.229064	-4.372114	1.449296
116.H	5.697173	-0.955163	1.499118
117.H	1.027964	-6.208212	1.740812
118.H	-6.984022	-0.927812	1.941468
119.H	5.709336	3.319850	1.963998
120.H	3.255335	2.856983	1.738791
121.H	-6.865306	-2.987920	2.001672
122.H	-1.719718	-3.686209	1.733445
123.H	-9.414293	1.893391	2.474925
124.H	4.478089	0.272954	1.900923
125.H	5.413937	-3.342266	2.154889

126.H	3.637301	4.544680	2.204681
127.H	-1.908496	-5.308435	2.433886
128.H	-0.284942	2.012895	2.055196
129.H	-7.710745	2.270428	2.851675
130.H	-8.296203	0.600001	2.964678
131.H	-0.389675	-4.428968	2.668758
132.H	3.854895	-3.948104	2.737158
133.H	2.408857	0.695516	2.565855
134.H	-2.740422	-1.902198	2.750358
135.H	-5.820708	-3.808677	3.172485
136.H	-6.379745	-2.161617	3.509275
137.H	1.607807	-3.503056	3.191336
138.H	-2.139056	0.433630	3.037969
139.H	1.115177	2.420144	3.085807
140.H	-3.565604	-3.251467	3.591183
141.H	-0.510296	2.967237	3.542707
142.H	5.682127	-1.711429	3.918704
143.H	-4.035826	-1.565295	3.925579
144.H	-0.591377	-2.140941	3.890993
145.H	4.389835	-0.548014	4.324857
146.H	4.165377	-2.273263	4.669959
147.H	-2.168384	1.456135	4.507663
148.H	-1.888141	-0.289274	4.643854
149.H	1.476703	1.252470	5.300563
150.H	-0.101684	1.906919	5.822538
151.H	0.230307	0.159579	5.947485
152.Cl	1.472450	0.663483	-0.428435
153.K	3.413846	2.424444	-1.955155
154.O	4.190719	1.548769	-4.540582
155.O	1.841319	3.081078	-4.327496
156.O	6.057039	1.719378	-2.461029
157.O	1.111907	3.992024	-1.806837
158.O	5.254176	2.526063	0.090437
159.O	3.025737	4.269114	0.221118
160.P	-2.155909	-0.893021	0.516729
161.Th	0.334069	-1.536000	0.743560

Energy: −1024.7926326 eV

Table S17. Coordinates and energy for 7

1.C	3.434056	1.993084	-3.745430
2.C	1.611248	0.351597	-3.584606
3.C	-3.909849	2.419489	-3.386266
4.C	-1.881085	0.957815	-3.432272
5.C	-4.127346	0.018005	-2.901603
6.C	-3.207806	1.250190	-2.733426
7.C	2.466883	1.325106	-2.751759
8.C	1.620490	2.459792	-2.151643
9.C	-1.582479	-2.951292	-2.018786
10.C	7.376027	1.463168	-1.389681
11.C	-3.927211	-3.607321	-1.503411

12.C	3.211565	0.546302	-1.642527
13.C	4.593290	0.755677	-1.521249
14.C	7.678571	-0.927648	-0.868919
15.C	-2.995058	1.590034	-1.262241
16.C	-2.525078	-3.469651	-0.937544
17.C	6.912550	0.327180	-0.484245
18.C	5.393564	0.077545	-0.619138
19.C	-2.019696	2.499366	-0.774079
20.C	-2.036838	-4.897396	-0.589579
21.C	2.580377	-0.398006	-0.766492
22.C	-3.872876	1.354295	-0.190949
23.C	2.075517	-3.363202	-0.064614
24.C	4.797830	-0.940721	0.122785
25.C	3.425917	-1.231167	0.053156
26.C	-2.515036	-2.650595	0.339334
27.C	-2.292412	5.292081	0.678902
28.C	7.222028	0.688363	0.964657
29.C	-2.307452	2.833802	0.567356
30.C	2.931025	-2.469014	0.847722
31.C	-3.610601	-1.946458	0.912765
32.C	-3.445280	2.091121	0.935372
33.C	-0.177005	4.069550	1.175485
34.C	4.089292	-3.356303	1.339626
35.C	-1.707300	4.019443	1.305185
36.C	-1.496536	-2.668708	1.342864
37.C	2.138566	-2.069406	2.109297
38.C	-3.306358	-1.569583	2.239927
39.C	-5.562428	-0.517392	2.683427
40.C	-1.982230	-2.015439	2.479430
41.C	-2.100062	3.991798	2.787770
42.C	-4.271325	-1.059232	3.306221
43.C	-4.628562	-2.271771	4.201471
44.C	-3.639377	0.025306	4.182187
45.H	2.844959	2.444630	-4.557655
46.H	-4.107870	2.210984	-4.451482
47.H	-2.050548	0.694309	-4.488243
48.H	4.127631	1.271000	-4.200972
49.H	1.064156	0.902519	-4.365203
50.H	2.257133	-0.390292	-4.078223
51.H	-4.266616	-0.205267	-3.969919
52.H	4.023247	2.803728	-3.290858
53.H	-3.305096	3.338124	-3.333220
54.H	-1.216051	1.832485	-3.413924
55.H	-4.873433	2.629587	-2.898163
56.H	-1.339107	0.133138	-2.949700
57.H	0.882378	-0.180885	-2.961639
58.H	1.096505	3.008883	-2.951444
59.H	-1.579538	-3.634385	-2.883573
60.H	7.214447	1.231347	-2.451908
61.H	-5.121466	0.193991	-2.465382

62.H	-3.909153	-4.232674	-2.409111
63.H	-3.698389	-0.872367	-2.426392
64.H	-1.879274	-1.959520	-2.390518
65.H	5.058669	1.491349	-2.171002
66.H	7.464586	-1.221161	-1.907417
67.H	-4.368517	-2.641753	-1.785532
68.H	8.454569	1.636179	-1.252707
69.H	2.267778	3.184520	-1.629372
70.H	-0.554210	-2.857134	-1.648668
71.H	-2.084968	-5.541351	-1.482264
72.H	6.860468	2.408789	-1.164932
73.H	8.765055	-0.761963	-0.780144
74.H	0.876832	2.063078	-1.447746
75.H	-1.214825	2.925884	-1.367235
76.H	-4.605145	-4.083524	-0.779524
77.H	2.663254	-3.711329	-0.927265
78.H	7.421861	-1.781164	-0.225414
79.H	-2.013057	5.382981	-0.381478
80.H	-0.995511	-4.889136	-0.239422
81.H	1.198955	-2.819618	-0.439214
82.H	-4.754208	0.717903	-0.221339
83.H	-2.656797	-5.350215	0.198261
84.H	0.140182	4.065075	0.123122
85.H	4.743231	-3.681399	0.516848
86.H	-4.570142	-1.794404	0.424415
87.H	1.730640	-4.248878	0.492106
88.H	-3.391105	5.290838	0.736266
89.H	8.303573	0.848511	1.107833
90.H	5.429273	-1.551433	0.764767
91.H	6.708274	1.616120	1.263079
92.H	-1.923403	6.186722	1.207612
93.H	6.912715	-0.106754	1.657911
94.H	-0.520182	-3.133118	1.249920
95.H	0.212791	4.988604	1.641735
96.H	3.666245	-4.258862	1.804861
97.H	0.265405	3.204367	1.690281
98.H	-3.956137	2.132181	1.892899
99.H	4.709850	-2.866378	2.105188
100.H	-5.370525	0.333562	2.017362
101.H	-6.087580	-1.291320	2.105255
102.H	1.283105	-1.428242	1.864098
103.H	1.763615	-2.968345	2.624379
104.H	-3.192047	4.039514	2.907749
105.H	2.794661	-1.546074	2.827322
106.H	-6.248213	-0.176212	3.473961
107.H	-1.678366	4.866323	3.308261
108.H	-1.728564	3.086877	3.286052
109.H	-1.428999	-1.889505	3.406893
110.H	-5.076792	-3.080537	3.605851
111.H	-3.391600	0.927714	3.607009

112.H	-3.731124	-2.672596	4.694070
113.H	-2.709369	-0.321524	4.653238
114.H	-5.347817	-1.977329	4.984028
115.H	-4.333074	0.317546	4.987086
116.Cl	-0.237812	0.880330	3.001293
117.K	2.570073	1.387689	1.564158
118.P	0.756179	-0.324335	-0.461449
119.Th	-1.521966	0.036629	0.649404

Energy: −755.81824666 eV

Table S18. Coordinates and energy for 8

1.C	-0.959385	3.045212	-3.517601
2.C	-0.387824	0.626291	-3.354732
3.C	0.974255	-2.983598	-2.763005
4.C	3.201521	-4.078403	-2.545949
5.C	-3.064958	-2.508431	-2.636119
6.C	-0.805182	1.858805	-2.547867
7.C	2.945645	-0.747554	-2.245810
8.C	-2.171899	1.586415	-1.904883
9.C	2.146194	-3.219069	-1.816653
10.C	3.932812	0.085933	-1.685806
11.C	1.620461	2.417268	-1.752530
12.C	0.244968	2.228794	-1.504375
13.C	2.832581	-1.910505	-1.422277
14.C	-2.530265	-2.698950	-1.243428
15.C	6.893270	-0.235356	-0.619488
16.C	-2.580779	-4.174625	-0.849504
17.C	1.657391	-3.992471	-0.574785
18.C	2.234114	2.916511	-0.600472
19.C	4.430816	-0.505722	-0.506744
20.C	3.714685	-1.719794	-0.339561
21.C	-4.594528	-1.876897	-0.092064
22.C	-3.198948	-1.824796	-0.202729
23.C	0.029684	2.625417	-0.163487
24.C	5.652860	-0.025062	0.272253
25.C	5.542725	1.472845	0.595117
26.C	1.256254	3.085510	0.410431
27.C	-6.799766	-1.259411	0.972105
28.C	-5.294212	-1.114651	0.828520
29.C	-2.447703	-0.964279	0.627535
30.C	-7.524206	0.069306	1.207406
31.C	1.074973	5.329287	1.422230
32.C	5.855252	-0.795125	1.574218
33.C	-4.563463	-0.225354	1.609664
34.C	-3.164153	-0.117853	1.514720
35.C	-7.122574	-2.219324	2.123513
36.C	1.411948	3.845208	1.713915
37.C	2.833672	3.776349	2.264510
38.C	-3.166013	2.289905	2.307796
39.C	-2.470860	0.918154	2.390242

40.C	0.439153	3.343056	2.778438
41.C	-2.361415	0.433810	3.828974
42.H	-1.724002	2.819048	-4.278434
43.H	-1.141004	0.399032	-4.125300
44.H	-0.015736	3.268229	-4.038249
45.H	0.571617	0.780566	-3.870873
46.H	1.296819	-2.495888	-3.693756
47.H	3.583819	-3.560811	-3.438064
48.H	-2.480502	-3.095982	-3.361777
49.H	-1.269707	3.954754	-2.982114
50.H	2.760602	-5.036521	-2.866718
51.H	0.511534	-3.942791	-3.040810
52.H	2.418443	-0.572265	-3.179177
53.H	-3.026676	-1.454930	-2.948213
54.H	-4.112936	-2.838551	-2.726238
55.H	-0.301835	-0.259067	-2.705910
56.H	-2.906845	1.328558	-2.682583
57.H	2.124983	2.232609	-2.698191
58.H	0.206999	-2.359095	-2.288308
59.H	4.057512	-4.297865	-1.890722
60.H	4.269742	1.027323	-2.113657
61.H	6.815567	0.339405	-1.553709
62.H	-2.036809	-4.797462	-1.577325
63.H	-2.550155	2.478942	-1.384888
64.H	7.012166	-1.295603	-0.887962
65.H	-2.124636	0.760217	-1.185008
66.H	-1.470596	-2.400627	-1.232899
67.H	-3.619523	-4.540901	-0.806952
68.H	1.238631	-4.965572	-0.874972
69.H	-5.151075	-2.553091	-0.748450
70.H	7.804608	0.089933	-0.091659
71.H	3.284310	3.185324	-0.516801
72.H	5.426129	2.073565	-0.318152
73.H	-7.180355	-1.700897	0.036955
74.H	0.874603	-3.420476	-0.051484
75.H	-2.129531	-4.337972	0.140462
76.H	2.488409	-4.194527	0.118671
77.H	-7.296183	0.803945	0.422336
78.H	3.888603	-2.434702	0.461707
79.H	-0.933282	2.614833	0.338155
80.H	1.758417	5.750577	0.670052
81.H	-8.613853	-0.084132	1.227279
82.H	6.455857	1.823467	1.102104
83.H	0.048402	5.432469	1.041356
84.H	4.693226	1.679537	1.259316
85.H	6.008483	-1.868677	1.384801
86.H	-3.224822	2.643807	1.269545
87.H	3.570348	4.157785	1.542550
88.H	-6.650918	-3.201471	1.974922
89.H	-8.209093	-2.374056	2.224425

90.H	-7.243768	0.512402	2.174157
91.H	6.752064	-0.424527	2.094033
92.H	1.163746	5.928854	2.342963
93.H	4.997505	-0.678908	2.249517
94.H	-5.086916	0.418249	2.319310
95.H	-1.449164	1.015929	1.993860
96.H	3.111899	2.751221	2.539862
97.H	-0.601318	3.439689	2.445183
98.H	-4.192149	2.255154	2.702956
99.H	-6.749125	-1.813079	3.076433
100.H	-2.621874	3.040621	2.900087
101.H	2.915293	4.399467	3.168780
102.H	0.626445	2.290132	3.031097
103.H	0.545632	3.937582	3.699756
104.H	-1.897645	-0.565068	3.868245
105.H	-3.353237	0.342073	4.299927
106.H	-1.757807	1.120173	4.444647
107.Cl	2.492744	0.155829	2.575147
108.K	0.759840	-2.218107	3.292099
109.P	-0.603031	-0.975686	0.598996
110.Th	1.643348	0.280891	-0.018370

Energy: −701.64163444 eV

Table S19. Coordinates and energy for 9

1.C	1.716574	1.674985	-4.734988
2.C	3.925453	0.731506	-4.384530
3.C	0.101131	-1.727360	-4.212409
4.C	-2.498118	1.447949	-4.052219
5.C	-2.973743	-2.352815	-3.810460
6.C	2.671721	1.216277	-3.707243
7.C	3.830117	3.306329	-2.953783
8.C	-0.541692	-2.133061	-2.916243
9.C	5.156105	5.385832	-2.418314
10.C	-1.940826	-2.372396	-2.712115
11.C	2.915740	2.291469	-2.665548
12.C	4.684076	6.809596	-2.127265
13.C	-2.725004	1.516475	-2.562489
14.C	-4.889104	0.038166	-2.353149
15.C	4.098482	4.342447	-2.087769
16.C	6.459330	5.080567	-1.678038
17.C	-1.048431	3.442328	-2.098096
18.C	-3.730796	0.850330	-1.809160
19.C	0.140549	-2.567602	-1.754083
20.C	-2.046599	2.392561	-1.696088
21.C	1.634858	-2.645240	-1.571403
22.C	-2.102904	-2.898997	-1.414864
23.C	2.224262	2.294227	-1.422701
24.C	3.388049	4.362781	-0.889853
25.C	-3.393790	-3.336819	-0.790051
26.C	-0.817118	-3.021202	-0.822299

27.C	2.457833	3.375739	-0.552407
28.C	-3.648774	1.302726	-0.474800
29.C	-2.608739	2.253071	-0.403021
30.C	-0.505757	-3.702064	0.483433
31.C	-4.664990	1.001235	0.591849
32.C	1.728465	3.506808	0.780074
33.C	-2.222043	3.066495	0.802568
34.C	1.159846	4.902026	1.016285
35.C	2.656341	3.111596	1.936511
36.C	2.372510	-1.288710	2.335969
37.C	-3.875497	-4.369433	3.095451
38.C	1.172267	-0.655936	3.015398
39.C	-3.446188	-2.909852	3.174640
40.C	-1.161959	-1.697485	3.082642
41.C	0.181738	-1.658979	3.522310
42.C	-1.998190	-2.703859	3.619313
43.C	-4.391481	-2.155133	4.088381
44.C	0.753398	-2.891132	4.069318
45.C	-1.361128	-3.903321	4.190047
46.C	1.695058	0.248534	4.168142
47.C	1.241291	-5.964879	4.528478
48.C	-0.020290	-3.892901	4.561883
49.C	0.582054	-5.038582	5.376448
50.C	1.557085	-4.417472	6.579814
51.H	1.500446	0.885096	-5.475398
52.H	2.109965	2.545221	-5.289151
53.H	4.381003	1.506877	-5.022826
54.H	3.702574	-0.125881	-5.041595
55.H	0.233794	-2.595229	-4.884355
56.H	-0.496636	-0.987836	-4.764474
57.H	-3.070756	0.635751	-4.517651
58.H	-2.810250	2.385368	-4.542688
59.H	-2.863373	-3.245268	-4.452267
60.H	-2.874582	-1.479435	-4.470213
61.H	0.757213	1.979122	-4.290916
62.H	-1.438860	1.297274	-4.308512
63.H	1.090856	-1.286901	-4.043241
64.H	4.359360	3.283668	-3.912517
65.H	4.690424	0.408112	-3.661775
66.H	5.356625	5.309397	-3.500955
67.H	-3.998837	-2.362169	-3.422813
68.H	-4.741068	-0.232880	-3.404225
69.H	2.215784	0.376696	-3.151169
70.H	3.747437	7.035649	-2.656471
71.H	5.442417	7.548985	-2.433803
72.H	-0.470395	3.145993	-2.980855
73.H	-1.563127	4.391307	-2.340689
74.H	7.254098	5.800295	-1.940638
75.H	2.114907	-3.084846	-2.460673
76.H	-5.822970	0.624484	-2.297315

77.H	6.819691	4.068714	-1.912791
78.H	-5.064208	-0.890691	-1.790199
79.H	4.501460	6.956560	-1.052312
80.H	-4.240180	-3.208366	-1.478242
81.H	-0.322628	3.642957	-1.304822
82.H	6.304174	5.129760	-0.588769
83.H	2.078225	-1.651840	-1.399707
84.H	1.887415	-3.283735	-0.714671
85.H	-3.366454	-4.402244	-0.509499
86.H	3.561029	5.176035	-0.179340
87.H	-3.630004	-2.777711	0.129894
88.H	0.521500	5.232456	0.184256
89.H	-0.162114	-4.736914	0.303272
90.H	-5.558113	1.639090	0.464935
91.H	-5.008174	-0.042373	0.562106
92.H	-2.861709	3.961967	0.898913
93.H	0.905013	2.774072	0.750890
94.H	1.956262	5.653795	1.139433
95.H	-1.184419	3.410612	0.730588
96.H	0.279011	-3.195577	1.057793
97.H	-1.385172	-3.756737	1.134362
98.H	-4.267928	1.189911	1.596686
99.H	-2.308141	2.483541	1.730243
100.H	2.076672	-2.027355	1.578603
101.H	3.040677	2.092500	1.792578
102.H	0.554504	4.920068	1.936690
103.H	3.519601	3.795108	1.996355
104.H	-3.127505	-0.691421	1.972679
105.H	2.961331	-0.517314	1.817389
106.H	-3.531219	-2.483465	2.163999
107.H	-3.183586	-4.974892	2.494315
108.H	0.681468	0.000141	2.279415
109.H	-4.873648	-4.435348	2.632243
110.H	2.124789	3.147499	2.900526
111.H	3.039855	-1.786985	3.056230
112.H	0.556715	-6.388337	3.775150
113.H	-5.437693	-2.228217	3.744941
114.H	-3.959847	-4.832072	4.090637
115.H	2.434890	0.967012	3.790527
116.H	2.063295	-5.495832	3.960799
117.H	-4.128129	-1.088969	4.141942
118.H	1.824508	-2.882271	4.281722
119.H	-1.992667	-4.723389	4.531800
120.H	0.874349	0.809052	4.639446
121.H	1.684713	-6.821510	5.072204
122.H	-4.342183	-2.561176	5.111742
123.H	2.174039	-0.369375	4.944566
124.H	-0.249775	-5.522646	5.916076
125.H	2.444729	-3.947747	6.137244
126.H	1.877064	-5.232865	7.245550

127.H	0.994912	-3.667537	7.148469
128.P	1.100999	0.915924	-0.968629
129.P	-1.743240	-0.368060	1.967829
130.Th	-1.250973	-0.197950	-0.953771

Energy: −840.92757859 eV

Table S20. Coordinates and energy for 10

1.H	-6.339896	-4.858426	-4.045280
2.H	-2.512317	2.560972	-4.286041
3.H	-5.028186	-3.656548	-3.971904
4.H	-2.868127	-0.469597	-3.843372
5.H	-6.509870	-3.418842	-3.026789
6.H	-1.880742	3.875652	-3.289118
7.H	1.711014	3.388429	-3.482056
8.H	2.712931	0.582195	-3.346423
9.H	0.385305	-1.978556	-3.288734
10.H	0.260424	4.268513	-2.985851
11.H	-4.480866	-5.673702	-2.648540
12.H	-3.169218	2.825703	-2.668741
13.H	-6.777970	-6.370435	-2.036241
14.H	-3.575181	0.399666	-2.478886
15.H	-2.757189	-1.150898	-2.208679
16.H	-0.758219	-2.144872	-1.943948
17.H	-0.242038	-4.620683	-1.900773
18.H	1.454267	3.757916	-1.775695
19.H	2.860261	1.521324	-1.845676
20.H	2.606094	-0.220963	-1.768725
21.H	-2.246497	5.042022	-1.607796
22.H	-6.914120	-4.974992	-0.946258
23.H	-2.616203	-5.274509	-1.420643
24.H	0.960033	-1.828556	-1.622345
25.H	4.505122	3.364183	-1.278960
26.H	3.467647	-2.384429	-1.404231
27.H	-5.559101	0.025019	-1.250697
28.H	-5.728228	-6.245034	-0.604043
29.H	-0.534692	5.247736	-1.205827
30.H	-5.900438	-2.600778	-0.993767
31.H	-4.079992	4.065102	-1.036458
32.H	-0.764044	-5.980673	-0.877408
33.H	0.817513	-5.246093	-0.614798
34.H	-4.457557	2.398619	-0.571368
35.H	2.452198	-3.627522	-0.638931
36.H	-1.764373	6.147408	-0.317619
37.H	4.252814	4.868593	-0.361226
38.H	-6.761950	-0.837346	-0.269137
39.H	5.737814	3.931314	-0.133385
40.H	8.131653	0.125516	0.038402
41.H	4.157469	-3.481452	-0.189970
42.H	7.681080	-2.331805	0.110868
43.H	-6.221568	0.766911	0.228570

44.H	-0.147007	-3.160288	0.154058
45.H	-4.923435	3.737243	0.479136
46.H	9.130125	-1.908868	1.040417
47.H	6.430835	2.082268	0.760216
48.H	1.888837	-1.461447	0.395793
49.H	1.088794	4.783398	0.471215
50.H	2.867697	3.066030	0.622786
51.H	5.521450	-2.094977	0.788897
52.H	-3.982323	-0.023306	0.746325
53.H	-0.511323	-1.773942	1.279248
54.H	7.613437	-2.273434	1.884658
55.H	-1.508498	-5.514561	1.558432
56.H	9.369153	0.308062	2.179209
57.H	8.038322	1.480824	2.162123
58.H	0.412654	5.621077	1.874820
59.H	-6.059012	-1.935092	1.930195
60.H	0.165344	-4.980186	1.838695
61.H	1.910729	-3.501456	1.835503
62.H	3.872748	4.779401	2.156583
63.H	-4.551791	1.788858	2.002959
64.H	1.281710	4.079904	2.083845
65.H	3.582534	-3.141892	2.320127
66.H	5.241102	3.689954	2.458508
67.H	-1.196916	-3.916148	2.273487
68.H	-5.729932	-0.284165	2.514411
69.H	-4.474385	-1.541630	2.644944
70.H	7.890062	-0.034049	3.092162
71.H	2.242100	-2.019454	2.741064
72.H	3.572333	3.224022	2.942149
73.H	-3.284647	0.894822	2.863769
74.H	-3.859862	2.455191	3.488362
75.H	0.381262	2.474329	3.494662
76.H	-1.127674	1.655682	3.919183
77.H	-0.925960	3.373800	4.310906
78.C	-5.790986	-4.176899	-3.375133
79.C	-2.241306	2.841597	-3.254820
80.C	-2.715349	-0.216091	-2.780126
81.C	0.937418	3.455007	-2.700708
82.C	-1.188726	1.890136	-2.731328
83.C	-5.175377	-4.926826	-2.223571
84.C	-1.407513	0.509112	-2.589273
85.C	0.206399	2.141931	-2.563268
86.C	2.327392	0.685903	-2.316398
87.C	-0.141911	-0.118546	-2.350138
88.C	0.840165	0.898488	-2.335945
89.C	0.122748	-1.589959	-2.289187
90.C	-6.196792	-5.668175	-1.414774
91.C	-4.337896	-4.027440	-1.335816
92.C	-3.028976	-4.346547	-1.020427
93.C	-0.227249	-5.020038	-0.877540

94.C	-4.859225	-2.860379	-0.791217
95.C	-1.542468	5.169291	-0.778965
96.C	4.652258	3.844023	-0.301473
97.C	3.258679	-2.902782	-0.458160
98.C	-5.881460	-0.178970	-0.219833
99.C	-4.124063	3.374493	-0.186351
100.C	-2.214759	-3.523522	-0.224610
101.C	-4.099561	-2.005106	-0.005817
102.C	-0.814922	-4.032746	0.133475
103.C	-1.653769	4.079795	0.249730
104.C	-2.736075	-2.298321	0.259969
105.C	8.038685	-1.784867	0.993486
106.C	-2.809040	3.313659	0.545367
107.C	7.671186	-0.310426	0.942502
108.C	-4.762205	-0.791757	0.622252
109.C	5.702359	1.267606	0.816290
110.C	6.166994	-0.042543	0.870406
111.C	2.855094	-1.928464	0.651122
112.C	5.203595	-1.051406	0.828721
113.C	3.953865	3.065283	0.812371
114.C	4.348743	1.591093	0.838323
115.C	3.829017	-0.774070	0.803396
116.C	3.365602	0.561400	0.911130
117.C	-0.701261	3.873415	1.287134
118.C	0.584662	4.621987	1.434809
119.C	-0.837649	-4.641327	1.529681
120.C	8.272654	0.408686	2.154895
121.C	-2.581657	2.643283	1.766605
122.C	-5.284189	-1.156051	2.009771
123.C	2.641205	-2.689580	1.962402
124.C	4.177325	3.721258	2.164211
125.C	-1.283703	2.980057	2.226761
126.C	-3.618343	1.900013	2.566703
127.C	-0.709290	2.602480	3.551253
128.K	4.642556	0.346739	3.718891
129.Th	-0.690005	1.414036	-0.039690
130.P	-1.745286	-1.079251	1.224061
131.P	1.612199	0.954490	1.285601

Energy: −841.72653510 eV

Table S21. Coordinates and energy for 11

1.C	-4.082994	0.758541	-3.811308
2.C	3.501295	-0.033255	-3.782677
3.C	-1.518762	-1.001423	-3.591170
4.C	1.008531	1.815070	-3.327334
5.C	-3.875272	-0.347398	-2.788915
6.C	3.442006	1.087431	-2.792087
7.C	-2.727006	-1.151333	-2.690171
8.C	2.315950	1.935181	-2.589298
9.C	2.077376	-3.101457	-2.340133

10.C	5.935287	1.072251	-2.105968
11.C	4.531276	1.625973	-2.080878
12.C	-6.309644	-0.492977	-1.795948
13.C	-4.849897	-0.859936	-1.899564
14.C	-2.391631	3.713304	-1.734996
15.C	2.728339	2.990934	-1.740041
16.C	-2.965882	-2.159130	-1.720343
17.C	-5.482023	2.949021	-1.394119
18.C	4.098021	2.808828	-1.435306
19.C	-2.077918	-3.323209	-1.392958
20.C	1.900906	4.179417	-1.332141
21.C	-4.283806	-1.963547	-1.223283
22.C	1.936683	-1.981690	-1.298773
23.C	4.967944	3.862926	-0.794895
24.C	3.309207	-1.549744	-0.734721
25.C	-3.030021	3.056185	-0.538522
26.C	-4.372875	2.612243	-0.430279
27.C	-5.031421	-2.876066	-0.306374
28.C	0.985350	-2.354197	-0.155670
29.C	1.005999	-3.663630	0.312584
30.C	-0.783922	-6.356209	0.815293
31.C	0.119904	-1.388994	0.462690
32.C	-2.406716	2.875946	0.714733
33.C	-4.556988	2.115134	0.885378
34.C	-1.071219	3.458862	1.119277
35.C	-5.854272	1.591827	1.458357
36.C	0.190355	-4.120747	1.341911
37.C	6.138121	1.919459	1.757821
38.C	0.179428	-5.590016	1.707081
39.C	-3.352867	2.295566	1.597133
40.C	5.534446	-1.126626	1.802258
41.C	-0.687224	-1.854357	1.549996
42.C	-3.082344	-1.065034	1.760501
43.C	4.770119	1.329608	1.971771
44.C	-0.644547	-3.195184	1.940044
45.C	4.515773	-0.058620	2.046028
46.C	3.598664	2.007229	2.381896
47.C	-1.650946	-0.950174	2.326632
48.C	3.437603	3.492747	2.613339
49.C	3.207108	-0.244167	2.545595
50.C	2.637877	1.030298	2.745924
51.C	2.588214	-1.560626	2.909515
52.C	-0.126729	-5.873432	3.174057
53.C	-3.168053	2.119368	3.080332
54.C	1.328274	1.341351	3.420956
55.C	-1.620853	-1.200844	3.851170
56.H	-4.038661	0.352248	-4.834620
57.H	3.773162	0.348609	-4.781589
58.H	-1.638996	-1.606957	-4.505779
59.H	1.044167	2.379920	-4.275131

60.H	-5.062063	1.235032	-3.693675
61.H	2.537614	-0.546453	-3.888794
62.H	-3.317408	1.547627	-3.755320
63.H	-1.379152	0.039444	-3.908203
64.H	4.252223	-0.788146	-3.515112
65.H	6.421394	1.268598	-3.075845
66.H	0.776947	0.772043	-3.568512
67.H	2.650849	-2.739454	-3.204505
68.H	-0.595800	-1.325168	-3.095425
69.H	0.166144	2.198033	-2.739336
70.H	-6.564000	0.377058	-2.411729
71.H	1.102941	-3.463684	-2.706108
72.H	-2.977878	3.560323	-2.650159
73.H	-5.133586	3.010487	-2.431969
74.H	-6.934624	-1.329138	-2.149404
75.H	5.954532	-0.017405	-1.954408
76.H	2.213020	5.084886	-1.879011
77.H	-1.378797	3.330440	-1.924199
78.H	2.630872	-3.959835	-1.931999
79.H	-2.451258	-4.233737	-1.892371
80.H	5.158876	4.677404	-1.513946
81.H	6.569577	1.526329	-1.334141
82.H	1.452505	-1.122177	-1.807685
83.H	-1.050819	-3.159937	-1.735556
84.H	-6.307704	2.229225	-1.361330
85.H	-2.297702	4.802530	-1.592784
86.H	-5.905852	3.934960	-1.140559
87.H	4.071803	-1.598897	-1.533482
88.H	0.838061	4.024301	-1.550547
89.H	-6.629067	-0.280479	-0.765027
90.H	-5.731047	-3.515754	-0.873168
91.H	5.943699	3.469276	-0.491970
92.H	1.989877	4.417613	-0.261147
93.H	-2.036876	-3.534531	-0.316767
94.H	-0.554999	-6.210022	-0.249788
95.H	4.500201	4.321966	0.086067
96.H	1.685678	-4.378114	-0.158358
97.H	-4.354905	-3.546066	0.238526
98.H	3.624545	-2.270421	0.036833
99.H	-5.627931	-2.333174	0.441341
100.H	-0.363968	3.471573	0.282432
101.H	-0.745228	-7.437039	1.026309
102.H	6.642569	1.525363	0.863671
103.H	6.124049	-0.951077	0.889796
104.H	-6.597312	1.403203	0.673791
105.H	-1.819498	-6.019285	0.980334
106.H	-5.714810	0.655951	2.019903
107.H	-1.199572	4.498908	1.467217
108.H	1.194770	-5.972835	1.502040
109.H	6.114385	3.011403	1.674902

110.H	-3.354207	-2.129149	1.677091
111.H	5.071889	-2.116267	1.706952
112.H	-0.606383	2.883116	1.926493
113.H	2.524305	3.902404	2.154166
114.H	4.288149	4.062397	2.220022
115.H	6.253023	-1.185467	2.638830
116.H	2.853160	-2.358830	2.204265
117.H	-6.303037	2.318736	2.155658
118.H	6.789356	1.675738	2.613670
119.H	-1.275001	0.080907	2.163999
120.H	-3.800194	-0.615878	2.471872
121.H	-1.299527	-3.514468	2.751917
122.H	-0.015678	-6.946801	3.388224
123.H	-1.160908	-5.599190	3.431944
124.H	0.995023	2.362549	3.199461
125.H	0.530369	0.655642	3.116300
126.H	1.494605	-1.508500	2.947605
127.H	-2.125373	1.906310	3.347677
128.H	-3.789683	1.308231	3.480946
129.H	3.371848	3.715573	3.690522
130.H	-3.457564	3.040404	3.613980
131.H	0.544240	-5.320089	3.846369
132.H	2.938044	-1.880714	3.906602
133.H	-2.088720	-2.163166	4.104180
134.H	-0.598263	-1.196191	4.260268
135.H	-2.198596	-0.418942	4.364163
136.H	1.431345	1.267904	4.517406
137.P	0.018086	0.375271	-0.144153
138.Th	-2.794142	0.267339	-0.301357
139.Th	2.795603	0.773260	-0.027693

Energy: –909.25497969 eV

Table S22. Coordinates and energy for 12

1.C	2.041608	1.050457	-5.411845
2.C	4.217299	0.291149	-4.410524
3.C	2.782182	0.740358	-4.106601
4.C	-0.052225	-2.299666	-3.730404
5.C	3.677662	2.913128	-3.232160
6.C	2.688310	1.937525	-3.170020
7.C	4.932987	4.911196	-2.396704
8.C	3.694487	4.031336	-2.400755
9.C	4.733254	6.321467	-1.856170
10.C	-0.333808	-2.618428	-2.290312
11.C	1.624780	2.102329	-2.245934
12.C	-2.591915	-1.366880	-2.071157
13.C	6.018520	4.196220	-1.586920
14.C	1.458703	-4.606959	-1.612687
15.C	2.618514	4.197419	-1.531176
16.C	-1.487015	-2.256679	-1.579516
17.C	0.361732	-3.557404	-1.516551

18.C	1.578414	3.268165	-1.456494
19.C	-0.668557	4.307913	-1.398670
20.C	4.220024	-1.795238	-1.086284
21.C	-3.958337	2.136462	-0.662091
22.C	0.375183	3.556696	-0.565066
23.C	-1.519617	-2.976028	-0.381811
24.C	-0.377056	-3.781260	-0.331920
25.C	3.802923	1.097424	0.102028
26.C	3.432368	-1.462714	0.160163
27.C	-2.689909	-3.081726	0.547534
28.C	-3.095304	1.801504	0.553233
29.C	0.691016	4.308976	0.718670
30.C	3.200378	-0.171845	0.655467
31.C	-0.412842	-4.699927	1.039488
32.C	3.256145	-3.865219	1.147140
33.C	-7.033239	-2.335411	1.447179
34.C	2.942950	-2.396797	1.107541
35.C	-4.952400	0.331379	1.457238
36.C	-7.919839	-0.036507	1.878241
37.C	-3.586163	0.622651	1.392054
38.C	-2.977541	3.023976	1.470642
39.C	-6.910136	-1.089869	2.325267
40.C	2.550243	-0.286928	1.903366
41.C	-5.457997	-0.634204	2.324310
42.C	2.382913	-1.661429	2.180180
43.C	-2.676121	-0.111899	2.194001
44.C	2.318236	0.859716	2.852990
45.C	-4.556367	-1.277155	3.168518
46.C	-3.179304	-1.060152	3.118582
47.C	1.885960	-2.250138	3.473546
48.C	-2.286684	-1.826411	4.075743
49.C	-2.785537	-3.244530	4.420609
50.C	-2.089113	-1.023214	5.355638
51.H	2.040312	0.180823	-6.088725
52.H	2.526893	1.888859	-5.934985
53.H	0.997735	1.337818	-5.222328
54.H	4.743631	1.009759	-5.056413
55.H	4.205830	-0.669705	-4.947129
56.H	-0.397848	-3.127154	-4.372708
57.H	4.505418	2.783750	-3.933764
58.H	-0.576894	-1.397237	-4.060834
59.H	1.017776	-2.159329	-3.932090
60.H	5.282112	4.990759	-3.440407
61.H	4.814875	0.164949	-3.497140
62.H	2.268339	-0.094660	-3.606473
63.H	0.262048	0.263590	-3.274273
64.H	3.940030	6.859549	-2.394990
65.H	-2.228060	-0.579472	-2.743178
66.H	5.661960	6.902827	-1.950666
67.H	-3.347251	-1.952327	-2.622144

68.H	1.106095	-5.461938	-2.214252
69.H	6.225144	3.193516	-1.985291
70.H	-0.975880	3.711526	-2.269647
71.H	6.961349	4.767272	-1.592474
72.H	3.922074	-1.171871	-1.939664
73.H	2.390105	-4.267990	-2.095173
74.H	-0.253495	5.260232	-1.765102
75.H	4.465772	6.308812	-0.788772
76.H	4.100632	-2.845253	-1.380241
77.H	-3.109598	-0.872311	-1.239716
78.H	-3.466496	2.912236	-1.264950
79.H	-4.120840	1.263308	-1.309605
80.H	2.583913	5.076175	-0.885484
81.H	5.296498	-1.622466	-0.924468
82.H	5.698341	4.079747	-0.540149
83.H	-1.565001	4.536483	-0.803097
84.H	1.705135	-4.980383	-0.619878
85.H	3.922948	1.067103	-0.986547
86.H	-4.944559	2.529742	-0.369683
87.H	-0.065911	2.592441	-0.272020
88.H	3.557694	-4.255201	0.169552
89.H	-6.779186	-2.095031	0.402963
90.H	-3.218487	-4.034845	0.371040
91.H	-2.089543	1.559723	0.177070
92.H	3.204557	1.984261	0.341479
93.H	1.003426	5.347606	0.525698
94.H	4.802625	1.247110	0.545287
95.H	-3.415274	-2.274297	0.394824
96.H	-7.778084	0.234428	0.821070
97.H	-5.636780	0.882937	0.811281
98.H	0.446527	-5.374306	1.134204
99.H	-2.620579	3.902979	0.913945
100.H	-8.060939	-2.733758	1.462333
101.H	-1.325464	-5.304943	1.002729
102.H	1.491830	3.822979	1.295090
103.H	-0.201143	4.353573	1.358412
104.H	-8.945108	-0.421551	1.979152
105.H	-6.353849	-3.132373	1.780149
106.H	4.099511	-4.045550	1.835495
107.H	-2.405835	-3.058326	1.603462
108.H	2.420247	-4.475363	1.514978
109.H	-3.960131	3.272398	1.901887
110.H	-0.452232	-4.043254	1.915737
111.H	-7.841975	0.884189	2.474195
112.H	-2.277799	2.830906	2.295949
113.H	1.829995	1.715289	2.369251
114.H	-7.157035	-1.378036	3.361217
115.H	3.280967	1.212944	3.257482
116.H	-0.382608	-0.129779	3.238499
117.H	1.376838	-3.212850	3.326482

118.H	-3.022316	-3.832137	3.523234
119.H	-4.951083	-2.014299	3.871914
120.H	1.693722	0.569230	3.704745
121.H	-1.308713	-1.935203	3.581627
122.H	2.725093	-2.438740	4.164338
123.H	1.185238	-1.580560	3.986275
124.H	-2.012424	-3.782679	4.989388
125.H	-3.685803	-3.218227	5.052158
126.H	-1.693375	-0.019359	5.145128
127.H	-3.049240	-0.895474	5.879267
128.H	-1.392291	-1.527933	6.044511
129.P	0.325676	0.824720	-1.973886
130.P	-0.876268	0.196421	1.946761
131.Th	0.662651	-1.252252	-0.014177

Energy: −843.92236332 eV

Table S23. Coordinates and energy for 13

1.C	-1.444473	0.504253	-3.488074
2.C	-2.551638	-2.423953	-2.945716
3.C	-2.719776	0.164279	-2.772331
4.C	4.804675	-1.589054	-2.538720
5.C	0.120042	5.160118	-2.517488
6.C	-3.226736	-1.132585	-2.579994
7.C	-3.700297	2.563077	-2.475251
8.C	1.879101	-2.785382	-2.526670
9.C	-3.710392	1.066278	-2.317239
10.C	-5.513970	-2.169826	-1.980754
11.C	-4.541439	-1.032213	-2.050570
12.C	1.046600	2.866057	-2.132474
13.C	-4.815344	0.345692	-1.871830
14.C	0.088623	3.809692	-1.858651
15.C	2.113314	0.654267	-1.961406
16.C	-6.109592	0.974241	-1.441001
17.C	1.045104	1.594096	-1.579545
18.C	4.079459	-2.110940	-1.344967
19.C	2.770970	-2.601209	-1.325245
20.C	-0.898934	3.481270	-0.964230
21.C	3.838710	3.386741	-0.427522
22.C	0.029155	1.253088	-0.611357
23.C	-0.955355	2.263637	-0.302557
24.C	4.607928	-2.262090	-0.049521
25.C	6.167272	1.504676	0.231586
26.C	2.482122	-3.034803	-0.030633
27.C	6.055294	-2.094710	0.322212
28.C	1.229917	-3.753459	0.359824
29.C	3.789688	2.341686	0.654570
30.C	3.610540	-2.814631	0.757012
31.C	-2.050934	2.026809	0.653361
32.C	4.858439	1.506286	0.968288
33.C	-3.914010	-3.565197	1.103059

34.C	-6.134576	-1.314177	1.340874
35.C	-4.687940	-1.178597	1.728117
36.C	-3.726963	-2.191142	1.685153
37.C	2.774384	2.182888	1.627197
38.C	1.526079	3.003661	1.776644
39.C	4.536299	0.810278	2.158830
40.C	3.756485	-3.207831	2.201294
41.C	-4.130523	-0.084960	2.416266
42.C	-2.593363	-1.748371	2.364195
43.C	3.228669	1.215038	2.539751
44.C	-4.813626	1.203569	2.726771
45.C	-1.376399	-2.544820	2.712916
46.C	5.463967	0.019366	3.029884
47.C	-2.841949	-0.451049	2.814339
48.C	-1.935766	0.348712	3.715440
49.C	2.512358	0.734611	3.769883
50.P	-0.009557	-0.411037	0.200521
51.Th	-2.750806	-0.227769	-0.007784
52.Th	2.740005	-0.237226	0.234624
53.H	-1.591854	0.410968	-4.578072
54.H	-2.884393	-2.785446	-3.934253
55.H	0.873448	5.200253	-3.315161
56.H	5.328336	-2.394786	-3.082443
57.H	2.191626	-2.162443	-3.373903
58.H	-4.319191	2.860911	-3.339799
59.H	-5.752720	-2.526216	-2.997193
60.H	-1.113972	1.529389	-3.284976
61.H	-0.620064	-0.167635	-3.211877
62.H	4.125546	-1.108687	-3.254684
63.H	-0.853574	5.417731	-2.960396
64.H	-2.689005	2.947683	-2.642114
65.H	1.907616	-3.833634	-2.869556
66.H	-1.461103	-2.311356	-2.995546
67.H	1.830116	3.104561	-2.857151
68.H	-6.525638	1.601050	-2.245802
69.H	5.571103	-0.847097	-2.269469
70.H	1.678922	-0.326185	-2.229182
71.H	-2.771613	-3.231236	-2.230546
72.H	2.733545	1.020525	-2.787895
73.H	0.831858	-2.542771	-2.295787
74.H	0.360082	5.956032	-1.794696
75.H	-6.464085	-1.875282	-1.519999
76.H	-5.132697	-3.042968	-1.430306
77.H	-4.108923	3.078576	-1.594016
78.H	-6.868814	0.223538	-1.193204
79.H	4.251470	2.994376	-1.368229
80.H	6.064522	1.222411	-0.829003
81.H	-5.997901	1.633349	-0.564515
82.H	-1.665249	4.224545	-0.725030
83.H	2.846502	3.793403	-0.647641

84.H	4.483727	4.227766	-0.117775
85.H	6.633738	-2.977708	0.000895
86.H	6.519781	-1.225460	-0.160892
87.H	6.620677	2.508760	0.239698
88.H	0.341333	-3.295811	-0.096412
89.H	-3.185104	-3.805056	0.310450
90.H	1.267773	-4.808923	0.038342
91.H	6.893676	0.820676	0.685123
92.H	-6.297692	-2.086595	0.582693
93.H	-2.642094	2.926543	0.861087
94.H	-6.560516	-0.377588	0.958809
95.H	1.227978	3.482254	0.836773
96.H	6.204036	-1.993146	1.401890
97.H	-3.791927	-4.341897	1.874353
98.H	-4.914918	-3.694230	0.676521
99.H	1.079957	-3.754472	1.446962
100.H	-1.648040	1.621178	1.599222
101.H	-6.737717	-1.596555	2.220679
102.H	-5.559662	1.473714	1.964701
103.H	4.760110	-2.990355	2.583237
104.H	-1.242265	-3.403493	2.042876
105.H	0.673484	2.404739	2.125409
106.H	6.410670	-0.210229	2.526918
107.H	3.589416	-4.288145	2.336356
108.H	1.691054	3.802259	2.520572
109.H	-4.104453	2.038303	2.797377
110.H	3.035083	-2.702106	2.865267
111.H	-0.463970	-1.935564	2.656529
112.H	5.034512	-0.930279	3.382354
113.H	-0.884050	0.272671	3.404070
114.H	-5.353254	1.157491	3.688872
115.H	5.718875	0.597464	3.934420
116.H	-1.451391	-2.944980	3.739142
117.H	1.425784	0.863774	3.683323
118.H	-2.205837	1.411758	3.736867
119.H	2.706909	-0.327809	3.981305
120.H	2.833065	1.293838	4.665847
121.H	-2.001014	-0.021333	4.752788

Energy: −799.57792205 eV

Table S24. Coordinates and energy for 14

1.C	-0.254411	2.115509	-4.632389
2.C	-1.817321	-0.770634	-4.158287
3.C	1.391094	-1.241634	-3.892282
4.C	-0.177438	2.467253	-3.181685
5.C	2.410396	2.511774	-3.070690
6.C	-1.036957	-1.143140	-2.929831
7.C	-2.704982	2.889992	-2.721201
8.C	0.358415	-1.386913	-2.816791
9.C	1.034664	2.732362	-2.496176

10.C	-1.245661	2.908793	-2.353047
11.C	-1.657105	-1.582734	-1.726071
12.C	-3.136998	-1.670769	-1.499369
13.C	0.602689	-1.974248	-1.550225
14.C	0.715312	3.378320	-1.273593
15.C	-0.688505	3.461245	-1.187919
16.C	1.923739	-2.530509	-1.098306
17.C	-0.642872	-2.095230	-0.877253
18.C	4.667088	1.071020	-0.376133
19.C	1.683134	4.028048	-0.307518
20.C	-1.445761	4.290623	-0.168932
21.C	-5.184420	0.672831	0.451928
22.C	-0.865547	-2.781681	0.448158
23.C	4.613514	0.032279	0.713377
24.C	5.801974	-0.560366	1.145507
25.C	3.374429	-0.346133	1.296013
26.C	-4.525844	-0.211636	1.482304
27.C	5.844168	-1.525478	2.133788
28.C	-5.292551	-1.189384	2.089592
29.C	-3.176193	-0.044334	1.855350
30.C	7.139899	-2.117287	2.598405
31.C	3.410324	-1.339561	2.305940
32.C	4.624733	-1.903065	2.691813
33.C	-4.782618	-2.065486	3.047722
34.C	-2.644158	-0.907998	2.847226
35.C	2.146302	-1.800069	2.997604
36.C	-5.629594	-3.126954	3.700029
37.C	-3.447277	-1.909798	3.399250
38.C	-1.243682	-0.721135	3.366726
39.H	0.004590	2.998486	-5.242306
40.H	-1.258965	1.803918	-4.938481
41.H	-1.171179	-0.451725	-4.982789
42.H	0.447543	1.322379	-4.921481
43.H	1.043016	-0.621643	-4.727437
44.H	-2.390155	-1.641552	-4.518582
45.H	1.653517	-2.225985	-4.314623
46.H	2.636850	3.265298	-3.844329
47.H	-2.551098	0.028727	-3.978828
48.H	2.332171	-0.807938	-3.521602
49.H	-3.029680	3.860102	-3.133419
50.H	-2.924222	2.132309	-3.484480
51.H	2.513906	1.528608	-3.551664
52.H	3.186022	2.589133	-2.301011
53.H	-3.348372	2.678516	-1.854957
54.H	-3.531074	-2.621257	-1.899494
55.H	-3.682972	-0.865541	-2.010563
56.H	2.072577	-3.547542	-1.502111
57.H	2.771726	-1.919509	-1.433386
58.H	4.053025	0.769674	-1.235883
59.H	5.697946	1.237679	-0.717353

60.H	1.827715	5.091184	-0.565901
61.H	-3.397621	-1.636251	-0.434484
62.H	-1.286602	5.364234	-0.361896
63.H	2.659384	3.532230	-0.315691
64.H	-4.596173	0.714851	-0.474907
65.H	-2.525109	4.107629	-0.215262
66.H	4.250635	2.026188	-0.022786
67.H	1.991560	-2.592397	-0.006888
68.H	-6.189804	0.306323	0.209175
69.H	-1.012587	-3.865012	0.298374
70.H	6.736056	-0.241389	0.671189
71.H	1.319230	3.990534	0.728771
72.H	-5.282606	1.708307	0.813513
73.H	-3.078606	2.123376	0.632368
74.H	-1.133484	4.098088	0.867597
75.H	-1.755830	-2.403902	0.965358
76.H	-0.001407	-2.663497	1.111864
77.H	7.841587	-2.263681	1.765085
78.H	-6.343125	-1.281702	1.797272
79.H	1.351239	-2.000676	2.270592
80.H	6.988205	-3.089725	3.086019
81.H	7.642840	-1.462778	3.329409
82.H	-5.695567	-4.032757	3.076232
83.H	-0.500432	-0.613959	2.559311
84.H	4.614886	-2.674075	3.469239
85.H	-6.657099	-2.775395	3.868066
86.H	1.756035	-1.015974	3.667356
87.H	2.328244	-2.700204	3.600180
88.H	-3.013131	-2.564511	4.160795
89.H	-0.936504	-1.564753	3.996996
90.H	-1.205289	0.185674	4.001098
91.H	-5.211881	-3.427608	4.669833
92.Na	0.249613	1.959919	2.752849
93.P	1.820710	0.497947	0.831380
94.P	-2.089187	1.253836	1.163485
95.Th	-0.114582	0.671953	-0.993240

Energy: –622.26606464 eV

Table S25. Coordinates and energy for 15

1.C	0.556082	-1.890304	-3.689773
2.C	-2.617036	-2.412339	-3.336581
3.C	-0.470688	-0.972323	-3.066731
4.C	1.037302	1.134046	-3.007008
5.C	-1.839365	-1.215245	-2.843690
6.C	-0.213321	0.351070	-2.724504
7.C	-2.415018	-0.042239	-2.347912
8.C	-1.401367	0.926324	-2.273717
9.C	-3.882298	0.155545	-2.104309
10.C	-1.599553	2.372834	-1.945128
11.C	-0.867309	-4.736801	-1.538968

12.C	5.434322	4.539806	-1.108290
13.C	4.570377	2.159275	-1.101292
14.C	3.933283	-0.236275	-1.178512
15.C	4.404656	3.486511	-0.744608
16.C	3.659159	1.167762	-0.752984
17.C	-0.736244	-3.847267	-0.332444
18.C	1.857196	-3.777767	-0.228049
19.C	3.263655	3.816694	-0.040881
20.C	2.514675	1.503850	0.023954
21.C	-3.245334	-3.887093	0.316563
22.C	0.481764	-3.483330	0.291120
23.C	2.318206	2.869879	0.339237
24.C	-8.131326	2.788950	1.040723
25.C	7.927027	1.206604	1.034260
26.C	-1.803489	-3.516874	0.546887
27.C	7.809076	-0.291940	1.180551
28.C	-6.698749	2.299394	1.159001
29.C	-5.629593	3.171801	1.110407
30.C	-6.419327	0.954761	1.313649
31.C	-4.308541	2.740329	1.194270
32.C	-3.219904	3.787287	1.197164
33.C	1.116021	3.322186	1.127062
34.C	6.281121	-2.030184	1.470357
35.C	-5.129392	0.468855	1.404808
36.C	-4.034705	1.362656	1.322631
37.C	-4.889414	-0.997309	1.600327
38.C	0.173129	-2.961528	1.554925
39.C	-1.233526	-2.961487	1.727125
40.C	4.929508	-2.369678	2.027693
41.C	1.175947	-2.660021	2.637561
42.C	-1.927413	-2.676915	3.017517
43.H	0.793726	-1.576623	-4.719803
44.H	-3.261011	-2.115774	-4.180677
45.H	0.204622	-2.928229	-3.743999
46.H	0.947208	1.644714	-3.982232
47.H	-1.956161	-3.206916	-3.699959
48.H	1.928262	0.496723	-3.063615
49.H	-4.387467	0.487278	-3.028105
50.H	1.506643	-1.890804	-3.134932
51.H	-1.651478	2.978364	-2.866928
52.H	-3.278107	-2.842677	-2.569675
53.H	-0.068731	-4.569332	-2.274366
54.H	1.227350	1.904953	-2.251580
55.H	6.246030	4.111836	-1.710647
56.H	-1.826924	-4.611582	-2.049467
57.H	4.724863	-0.275014	-1.940109
58.H	4.985172	5.358649	-1.689680
59.H	5.443902	1.877646	-1.698862
60.H	-4.373144	-0.776388	-1.790661
61.H	-4.080489	0.907186	-1.330313

62.H	-0.802810	-5.795273	-1.231758
63.H	3.021107	-0.709161	-1.567374
64.H	-0.776228	2.782061	-1.343612
65.H	-2.535446	2.529204	-1.396931
66.H	1.888181	-3.789880	-1.326248
67.H	5.886489	4.996425	-0.213710
68.H	4.240934	-0.869459	-0.332834
69.H	-3.532665	-4.409534	-0.590871
70.H	-8.657659	2.294844	0.209999
71.H	8.108771	-0.785173	0.235315
72.H	7.237410	1.583916	0.266020
73.H	3.087924	4.862878	0.231477
74.H	2.206883	-4.769065	0.111013
75.H	2.579320	-3.027103	0.115296
76.H	8.950859	1.476893	0.740905
77.H	-8.167482	3.872061	0.864681
78.H	6.378198	-2.402526	0.430883
79.H	-2.479587	3.624664	0.403756
80.H	-5.820892	4.244387	1.005589
81.H	-4.309416	-1.433328	0.770958
82.H	0.184662	3.021905	0.626086
83.H	-3.993610	-3.695043	1.077662
84.H	1.113091	4.411043	1.268672
85.H	-7.247912	0.240107	1.371482
86.H	-3.649487	4.789348	1.065531
87.H	-8.705521	2.580373	1.957113
88.H	-1.602801	1.907725	1.423828
89.H	8.488076	-0.669063	1.970734
90.H	-5.834133	-1.552352	1.678771
91.H	7.717569	1.721240	1.985821
92.H	4.120969	-1.851967	1.488796
93.H	7.074223	-2.529680	2.062685
94.H	4.742935	-3.448348	1.942556
95.H	-2.667006	3.777634	2.149608
96.H	1.068405	2.826746	2.110526
97.H	2.085373	-2.210953	2.224841
98.H	-4.303322	-1.171094	2.514987
99.H	4.860329	-2.111087	3.095935
100.H	-2.996608	-2.905835	2.948246
101.H	1.446235	-3.585333	3.175941
102.H	-1.841073	-1.626249	3.331587
103.H	0.771120	-1.954419	3.374215
104.H	-1.516414	-3.307782	3.822545
105.K	4.431683	1.185643	2.312549
106.O	6.478240	-0.643842	1.488596
107.P	1.449150	0.195112	0.731844
108.P	-2.334189	0.696243	1.502746
109.Th	-0.756087	-1.004612	-0.303535

Energy: −705.89549614 eV

Table S26. Coordinates and energy for 16

1.C	-1.974314	0.650539	-4.392463
2.C	0.228116	3.119019	-3.899756
3.C	0.927444	-0.737517	-3.930096
4.C	-1.545003	-0.203706	-3.226228
5.C	-0.282150	-0.840767	-3.043262
6.C	0.166925	3.058417	-2.396811
7.C	-3.920429	-0.463638	-2.237986
8.C	-2.438249	-0.733485	-2.273130
9.C	-2.199200	4.076862	-2.052831
10.C	2.630713	2.294334	-2.072833
11.C	-0.417513	-1.770312	-1.990385
12.C	-0.860364	3.570132	-1.578354
13.C	0.577470	-2.834719	-1.601277
14.C	1.285124	2.753136	-1.570843
15.C	-1.747699	-1.698329	-1.510079
16.C	6.709414	1.331601	-0.459922
17.C	-2.361777	-2.642120	-0.509359
18.C	3.666739	-0.925089	-0.446690
19.C	-5.692154	2.121855	-0.082140
20.C	-0.372111	3.626608	-0.248253
21.C	7.275421	0.040058	0.159496
22.C	0.959547	3.130612	-0.247070
23.C	5.996783	2.000321	0.708807
24.C	-5.542495	0.878603	0.761716
25.C	3.264447	-1.884783	0.647499
26.C	-6.681059	0.139430	1.047233
27.C	-1.010878	4.378335	0.896642
28.C	4.145961	-2.903753	1.003311
29.C	1.911026	3.170155	0.921369
30.C	6.631501	-0.026916	1.541151
31.C	-4.290159	0.466684	1.283518
32.C	2.014691	-1.742665	1.308275
33.C	-6.662798	-1.002942	1.834927
34.C	-7.946242	-1.692765	2.212430
35.C	3.841194	-3.822265	2.003089
36.C	4.777639	-4.952851	2.351163
37.C	-4.254843	-0.720488	2.044146
38.C	-5.424058	-1.432171	2.292576
39.C	1.683579	-2.716551	2.290613
40.C	2.598082	-3.711690	2.625355
41.C	-2.950636	-1.210639	2.634620
42.C	0.331724	-2.708865	2.955902
43.H	-2.643860	0.075295	-5.053711
44.H	-1.124026	0.976978	-5.000770
45.H	0.970523	-1.583259	-4.636794
46.H	0.927353	0.179764	-4.533053
47.H	-0.766103	3.160792	-4.358722
48.H	0.764617	4.030855	-4.214672
49.H	0.766782	2.269706	-4.341516

50.H	-2.533127	1.548299	-4.089205
51.H	1.867825	-0.758851	-3.359885
52.H	-4.455352	-1.182634	-2.882600
53.H	-2.539072	3.554589	-2.957719
54.H	3.234833	3.147459	-2.429033
55.H	-2.159442	5.151202	-2.300225
56.H	-4.161719	0.540576	-2.612423
57.H	2.545592	1.592732	-2.914062
58.H	0.369027	-3.767303	-2.155166
59.H	1.610099	-2.546135	-1.833728
60.H	5.989520	1.096691	-1.254144
61.H	-2.976051	3.947144	-1.288015
62.H	-4.336713	-0.553713	-1.226982
63.H	3.199414	1.791069	-1.281731
64.H	7.488124	1.975438	-0.890041
65.H	2.864386	-0.840167	-1.192166
66.H	-2.679407	-3.573217	-1.009432
67.H	-5.005048	2.111989	-0.940416
68.H	4.593010	-1.252910	-0.940389
69.H	-6.719400	2.212641	-0.459836
70.H	7.011499	-0.835494	-0.446741
71.H	0.535089	-3.064557	-0.530619
72.H	8.370860	0.065889	0.237433
73.H	-3.249065	-2.212282	-0.027695
74.H	3.805384	0.089940	-0.049678
75.H	5.110784	2.582085	0.426589
76.H	-1.646520	-2.923183	0.274530
77.H	-5.463111	3.030556	0.496281
78.H	-7.638402	0.489377	0.646824
79.H	5.093962	-2.998742	0.463635
80.H	-3.310622	2.566241	0.440404
81.H	-2.091555	4.496116	0.756572
82.H	2.461110	4.127686	0.944496
83.H	-0.574096	5.387769	0.976162
84.H	-8.668544	-1.688985	1.383574
85.H	6.685154	2.655746	1.277293
86.H	2.632901	2.345186	0.869406
87.H	5.806667	-4.741573	2.028432
88.H	6.207575	-1.017025	1.766746
89.H	4.473432	-5.893217	1.863283
90.H	-0.871890	3.872733	1.861505
91.H	1.379918	3.067843	1.877107
92.H	7.361700	0.238117	2.330708
93.H	-7.774634	-2.737730	2.506408
94.H	-2.122777	-1.169120	1.912207
95.H	-8.430684	-1.190652	3.066035
96.H	-0.460355	-2.868796	2.210529
97.H	-5.361554	-2.342848	2.897365
98.H	-3.045932	-2.237773	3.010120
99.H	4.798142	-5.149874	3.433057

100.H	2.319387	-4.445280	3.389014
101.H	-2.646582	-0.562619	3.473209
102.H	0.256405	-3.489759	3.725485
103.H	0.105655	-1.726856	3.400056
104.O	5.560262	0.920702	1.539608
105.P	0.956970	-0.300822	1.016998
106.P	-2.748740	1.440877	1.109311
107.Rb	3.784269	-0.289123	3.425342
108.Th	-0.595351	0.803762	-0.826607

Energy: –703.35655251 eV

Table S27. Coordinates and energy for 17

1.C	4.246597	3.318660	-4.820316
2.C	3.382011	2.691037	-3.743935
3.C	2.067619	3.118635	-3.536404
4.C	3.844031	1.662733	-2.958330
5.C	0.684386	-1.659004	-2.752712
6.C	-7.862036	0.339771	-2.086774
7.C	1.256722	2.558556	-2.553857
8.C	-2.299510	-0.778965	-2.546771
9.C	-0.150355	3.062298	-2.398702
10.C	7.103725	3.495948	-1.613005
11.C	3.073236	1.070456	-1.953994
12.C	-5.582706	1.226822	-1.448273
13.C	1.747884	1.543556	-1.707387
14.C	-0.280255	-2.152074	-1.715299
15.C	-1.641177	-1.773881	-1.644621
16.C	-6.695984	0.436320	-1.130933
17.C	3.678145	0.000831	-1.097022
18.C	-3.305082	2.182497	-0.956269
19.C	7.567308	3.035401	-0.265945
20.C	-0.077932	-3.213895	-0.810755
21.C	1.218333	-4.011504	-0.722709
22.C	-4.469341	1.302120	-0.595830
23.C	-2.273681	-2.606258	-0.699251
24.C	-3.743712	-2.710493	-0.429414
25.C	-6.692361	-0.214892	0.088779
26.C	-1.311854	-3.498837	-0.175214
27.C	-4.453311	0.550076	0.593097
28.C	-1.631151	-4.724832	0.663041
29.C	-5.614313	-0.172401	0.957673
30.C	6.922617	1.752578	1.607389
31.C	-5.723756	-0.902092	2.254841
32.C	5.756657	1.135546	2.304121
33.C	2.842060	-1.651087	2.269312
34.C	1.430311	-1.428607	2.743420
35.C	0.763893	-3.911213	2.982222
36.C	0.464149	-2.439840	3.010937
37.C	0.898310	-0.187456	3.181473
38.C	1.666206	1.103060	3.267982

39.C	-0.669584	-1.825696	3.576077
40.C	-0.404625	-0.432297	3.688524
41.C	-1.885524	-2.522254	4.079351
42.C	-1.222527	0.573925	4.478190
43.H	4.047423	2.873091	-5.808002
44.H	4.057561	4.397504	-4.912266
45.H	5.315421	3.177022	-4.610576
46.H	1.659284	3.918251	-4.161928
47.H	0.485812	-2.150274	-3.722098
48.H	-2.524910	-1.237676	-3.525412
49.H	-7.571196	-0.147003	-3.030407
50.H	4.859987	1.285294	-3.117684
51.H	-0.359156	3.896547	-3.082643
52.H	-8.255451	1.334665	-2.345008
53.H	7.959896	3.893512	-2.176591
54.H	0.611728	-0.576153	-2.907461
55.H	6.656923	2.688743	-2.225833
56.H	-5.570000	1.796018	-2.382432
57.H	-0.871119	2.256986	-2.598434
58.H	-1.648881	0.084390	-2.741504
59.H	1.725839	-1.884740	-2.490186
60.H	-8.686246	-0.242301	-1.653082
61.H	-3.248091	-0.411070	-2.137373
62.H	6.369930	4.320297	-1.564565
63.H	-3.430677	2.621404	-1.954448
64.H	1.416956	-4.524187	-1.676969
65.H	4.676447	-0.288829	-1.451961
66.H	-0.351531	3.376961	-1.362904
67.H	3.028917	-0.884358	-1.057765
68.H	-4.185541	-3.518325	-1.039352
69.H	-2.347805	1.639899	-0.928516
70.H	-4.280898	-1.786986	-0.670762
71.H	8.543312	2.589004	-0.096300
72.H	2.093864	-3.377362	-0.515940
73.H	-3.204081	3.001437	-0.225927
74.H	1.172933	-4.782543	0.055433
75.H	3.753791	0.329678	-0.045694
76.H	-7.571742	-0.799296	0.377826
77.H	-2.238572	-5.432287	0.074984
78.H	-3.957759	-2.954405	0.620777
79.H	-0.725111	-5.253873	0.975822
80.H	7.896716	2.016735	2.003144
81.H	-2.214036	-4.488160	1.565411
82.H	5.158093	0.514979	1.622260
83.H	2.928818	-2.526105	1.610388
84.H	-6.705891	-1.384063	2.351150
85.H	3.210302	-0.781714	1.711896
86.H	1.362219	-4.207499	2.111149
87.H	-4.952501	-1.681314	2.356479
88.H	5.075642	1.875195	2.776817

89.H	2.326417	1.220712	2.397852
90.H	-3.410508	-0.161469	2.713779
91.H	-5.595175	-0.224358	3.113202
92.H	6.097657	0.481582	3.119013
93.H	-0.144795	-4.523691	2.988855
94.H	3.526369	-1.822816	3.118487
95.H	-2.053055	-3.482661	3.572789
96.H	1.344754	-4.194310	3.877132
97.H	0.995243	1.972301	3.286976
98.H	2.278700	1.131437	4.185873
99.H	-2.793726	-1.919129	3.942074
100.H	-1.304381	1.542005	3.966843
101.H	-2.244271	0.219459	4.653971
102.H	-1.809034	-2.745545	5.157630
103.H	-0.757727	0.745147	5.462986
104.Cs	3.599861	3.671819	-0.007531
105.O	6.480672	2.395061	0.421201
106.P	0.816017	1.002649	-0.237618
107.P	-2.947910	0.613672	1.613966
108.Th	-0.565693	-0.994337	0.832694

Energy: –699.92350653 eV

Table S28. Coordinates and energy for 18

1.C	-1.145069	-1.009752	-3.575519
2.C	1.736121	0.262480	-3.226381
3.C	-0.048939	-1.534278	-2.693373
4.C	1.240811	-0.974683	-2.527202
5.C	-1.210977	-3.767064	-2.046005
6.C	-0.069820	-2.790491	-2.027717
7.C	2.587507	3.608411	-1.695239
8.C	2.013360	-1.869742	-1.779048
9.C	-1.690950	2.073069	-1.727171
10.C	3.498003	-1.770555	-1.512553
11.C	1.227826	-2.999040	-1.476237
12.C	-4.417258	-0.926004	-1.338049
13.C	4.998282	3.398653	-1.150500
14.C	7.478576	3.402015	-0.724003
15.C	3.711285	2.892222	-0.987191
16.C	1.757345	-4.298503	-0.937756
17.C	6.105940	2.826874	-0.541893
18.C	-6.091623	0.656443	-0.381502
19.C	-4.739354	0.322235	-0.548441
20.C	-7.948010	2.168268	0.393597
21.C	3.502406	1.769222	-0.154336
22.C	-6.496940	1.812760	0.266688
23.C	-3.749771	1.166044	-0.019780
24.C	5.892750	1.703353	0.241522
25.C	4.613573	1.170242	0.459911
26.C	-5.506227	2.628699	0.792979

27.C	-4.151248	2.328371	0.677529
28.C	-3.169704	3.300041	1.285458
29.C	4.496365	-0.012797	1.393726
30.C	-1.025053	-4.391161	1.453181
31.C	1.304359	2.514245	1.455066
32.C	-3.168536	-2.139173	1.744454
33.C	-0.725023	-2.967496	1.830131
34.C	-1.690844	-1.957386	2.006048
35.C	0.514151	-2.482186	2.340453
36.C	1.801954	-3.245771	2.461971
37.C	-1.085821	-0.864477	2.636279
38.C	0.277482	-1.177711	2.854230
39.C	-1.787419	0.346148	3.190281
40.C	1.261998	-0.377848	3.657821
41.H	-1.053801	-1.431598	-4.591783
42.H	2.052348	0.016896	-4.254786
43.H	-1.107822	0.081196	-3.676483
44.H	-2.141449	-1.272170	-3.200361
45.H	0.957221	1.031410	-3.303069
46.H	-1.411058	-4.111559	-3.073771
47.H	2.597182	0.707411	-2.714723
48.H	2.959342	4.137187	-2.583021
49.H	-2.241612	1.767515	-2.627997
50.H	-4.529320	-0.739899	-2.418156
51.H	4.066970	-2.266608	-2.316910
52.H	1.794204	2.910430	-1.989248
53.H	5.136574	4.281543	-1.781920
54.H	8.063788	2.822989	-1.455987
55.H	-0.616846	2.111555	-1.954618
56.H	2.268916	-4.858791	-1.738792
57.H	7.435117	4.437463	-1.086130
58.H	-2.154790	-3.334881	-1.677885
59.H	-0.995790	-4.657267	-1.445010
60.H	3.841579	-0.730444	-1.463362
61.H	-2.015212	3.090276	-1.460768
62.H	-5.100519	-1.746022	-1.076449
63.H	-3.381904	-1.240145	-1.164285
64.H	-8.490891	1.982498	-0.544022
65.H	2.116707	4.359274	-1.040365
66.H	-6.848046	-0.009522	-0.809097
67.H	0.964342	-4.950100	-0.553999
68.H	3.782139	-2.262277	-0.572548
69.H	8.045784	3.395530	0.217375
70.H	2.494336	-4.158729	-0.136594
71.H	-8.081002	3.225199	0.658278
72.H	6.745419	1.228144	0.737219
73.H	-8.442343	1.569384	1.174850
74.H	-2.815902	4.030426	0.539920
75.H	-1.767415	-4.472227	0.648950
76.H	-5.796399	3.543841	1.317736

77.H	-3.357513	-2.779289	0.872318
78.H	5.298087	-0.741762	1.210155
79.H	-0.130514	-4.941353	1.140168
80.H	1.454440	3.532702	1.066698
81.H	3.521929	-0.501348	1.281162
82.H	-3.679994	-1.184027	1.576174
83.H	-3.636718	3.867243	2.101753
84.H	0.237288	2.397768	1.689164
85.H	-2.278757	2.782539	1.661368
86.H	1.745662	-4.222403	1.969161
87.H	2.664549	-2.706869	2.039261
88.H	-1.442499	-4.931294	2.319975
89.H	4.585337	0.312135	2.442615
90.H	1.891526	2.414846	2.379064
91.H	-3.654658	-2.624393	2.607812
92.H	-2.706521	0.577088	2.639762
93.H	-1.148996	1.238133	3.166950
94.H	2.045069	-3.431688	3.520949
95.H	2.292459	-0.516585	3.309665
96.H	1.045197	0.696229	3.626926
97.H	-2.066738	0.174156	4.243987
98.H	1.228950	-0.683516	4.718361
99.P	-1.947343	0.910025	-0.314924
100.P	1.765081	1.252631	0.186637
101.Th	0.081295	-0.964735	0.057219

Energy: –661.27833919 eV

Table S29. Coordinates and energy for 19

1.C	-0.996404	1.824843	-4.321217
2.C	-2.355492	-0.894426	-4.080125
3.C	0.741865	-1.681776	-3.951876
4.C	1.876429	2.187765	-3.147917
5.C	-1.601362	-1.415540	-2.870875
6.C	-0.682581	2.049514	-2.853701
7.C	-0.278054	-1.878603	-2.850310
8.C	0.613239	2.283046	-2.353484
9.C	4.916869	-0.124942	-1.932943
10.C	-3.105248	2.337802	-1.971829
11.C	-1.597319	2.399168	-1.850206
12.C	-2.221911	-1.836507	-1.678554
13.C	-0.076495	-2.612962	-1.639153
14.C	-3.675762	-1.688311	-1.362010
15.C	1.063309	-3.519050	-1.337857
16.C	0.514919	2.784953	-1.050716
17.C	-1.300770	-2.566811	-0.918994
18.C	4.832580	0.549383	-0.586695
19.C	-0.863706	2.873947	-0.717417
20.C	5.930785	1.271496	-0.140125
21.C	1.621502	3.367695	-0.248043
22.C	3.693767	0.434716	0.239847

23.C	-1.603841	-3.372601	0.292310
24.C	-1.420169	3.618113	0.443405
25.C	5.962175	1.888747	1.093670
26.C	7.208027	2.581475	1.596371
27.C	3.697945	1.094067	1.484826
28.C	4.824677	1.796363	1.890495
29.C	-4.887858	0.116896	2.005650
30.C	2.508696	1.027795	2.399493
31.C	-2.559344	-0.749615	2.603506
32.C	-3.960327	-0.791507	2.772939
33.C	-0.270011	-1.649209	3.211647
34.C	-1.764090	-1.630864	3.362255
35.C	-4.510361	-1.665772	3.700308
36.C	-2.361271	-2.480162	4.283885
37.C	-3.739380	-2.505750	4.477650
38.C	-4.356480	-3.373001	5.550857
39.H	-0.732640	2.741869	-4.875266
40.H	-0.413979	1.014057	-4.772950
41.H	-2.992183	-1.708266	-4.467107
42.H	-1.692337	-0.599463	-4.895315
43.H	0.762230	-2.546556	-4.635229
44.H	-2.057206	1.642069	-4.501855
45.H	0.522488	-0.798736	-4.564448
46.H	-3.027713	-0.060448	-3.849606
47.H	1.879384	1.334363	-3.840860
48.H	2.018388	3.094142	-3.761964
49.H	1.757984	-1.562851	-3.551465
50.H	-3.430399	1.589828	-2.705461
51.H	5.815909	0.197359	-2.473751
52.H	4.038183	0.096511	-2.555031
53.H	2.755145	2.094469	-2.497908
54.H	-3.518747	3.305951	-2.299029
55.H	4.964163	-1.220691	-1.831350
56.H	-4.086690	-0.723787	-1.692693
57.H	-4.265538	-2.472812	-1.867234
58.H	0.757891	-4.568154	-1.484426
59.H	1.918023	-3.338296	-2.000669
60.H	-3.582071	2.090104	-1.013794
61.H	2.831241	-1.402261	-1.216792
62.H	6.809454	1.336068	-0.789018
63.H	1.835570	4.395578	-0.587943
64.H	-3.865527	-1.781463	-0.285579
65.H	2.557024	2.799600	-0.329532
66.H	1.432628	-3.423961	-0.307140
67.H	-2.069215	-4.330652	0.003134
68.H	-1.220343	4.696364	0.329075
69.H	7.964194	2.667570	0.805182
70.H	-2.507446	3.500179	0.524171
71.H	1.365632	3.434489	0.817146
72.H	-2.297883	-2.871444	0.979061

73.H	-0.697176	-3.620652	0.858869
74.H	-4.685676	0.086972	0.925709
75.H	6.990654	3.593866	1.967348
76.H	-0.992315	3.303995	1.405665
77.H	-2.784766	1.389617	1.333651
78.H	7.660749	2.022314	2.429655
79.H	-5.935237	-0.166485	2.171625
80.H	1.587832	1.396093	1.923588
81.H	-4.772836	1.166269	2.319816
82.H	0.048439	-1.849285	2.177801
83.H	4.813320	2.283623	2.870112
84.H	2.304782	-0.013690	2.692441
85.H	2.672468	1.617333	3.310448
86.H	-5.597180	-1.672675	3.827602
87.H	0.156833	-0.669967	3.477859
88.H	0.186800	-2.411884	3.854883
89.H	-1.722393	-3.141362	4.877056
90.H	-4.026885	-4.419207	5.469276
91.H	-5.452684	-3.359885	5.493973
92.H	-4.071280	-3.021607	6.554455
93.P	2.273051	-0.648154	-0.152826
94.P	-1.710519	0.491997	1.562781
95.Th	-0.379995	0.105481	-0.952425

Energy: –624.71537747 eV

Table S30. Coordinates and energy for 20

1.C	0.350072	3.910535	-4.342131
2.C	-0.689019	3.027143	-4.071395
3.C	-0.072391	-5.446057	-3.652702
4.C	1.337744	4.097839	-3.408243
5.C	-0.819262	-0.623694	-3.318312
6.C	-0.739098	2.325416	-2.872412
7.C	-2.020714	0.123878	-2.827981
8.C	-3.229143	-0.552881	-2.604117
9.C	-1.972728	1.499067	-2.593836
10.C	-0.180893	-4.535393	-2.445138
11.C	4.020664	2.209566	-2.414999
12.C	2.812330	-1.282174	-2.493700
13.C	-4.376702	0.097449	-2.211818
14.C	1.331384	3.400692	-2.211881
15.C	-5.667034	-0.658709	-2.014079
16.C	-3.116493	2.177118	-2.146547
17.C	-4.309157	1.460498	-1.983554
18.C	-1.433940	-4.206821	-1.911823
19.C	-3.070690	3.633328	-1.789533
20.C	0.927708	-3.966051	-1.841707
21.C	0.302933	2.463020	-1.931226
22.C	3.435608	-2.039762	-1.329551
23.C	3.635139	3.068974	-1.238131
24.C	2.374983	3.679126	-1.177586

25.C	4.685566	-1.344253	-0.813477
26.C	-1.556228	-3.413156	-0.792418
27.C	0.835021	-3.147287	-0.704413
28.C	4.521757	3.198611	-0.175257
29.C	2.090504	4.542573	-0.107543
30.C	0.760390	5.261326	-0.025585
31.C	-0.429775	-2.900464	-0.131728
32.C	3.373855	-4.739098	0.653997
33.C	4.232638	3.996388	0.921496
34.C	3.009930	4.673869	0.937739
35.C	3.023859	-3.378211	1.235843
36.C	-3.453464	0.327860	1.328884
37.C	5.203250	4.089554	2.083836
38.C	-1.356865	-2.589234	2.061500
39.C	-1.683721	-3.943049	2.206809
40.C	-2.611376	2.431857	2.316876
41.C	2.172272	-3.525144	2.498288
42.C	-2.815208	0.942091	2.581319
43.C	-2.494685	-4.389553	3.235162
44.C	-1.844259	-1.702804	3.048673
45.C	-2.679184	-2.174258	4.061368
46.C	-3.023861	-3.530972	4.172818
47.C	-0.850284	0.379612	4.757818
48.C	-3.930412	-4.023785	5.272346
49.C	-0.453699	1.806736	4.969825
50.C	0.160769	-0.577774	5.319157
51.H	0.371647	4.457095	-5.285887
52.H	-1.497054	2.894690	-4.793735
53.H	-0.677997	-5.073571	-4.492156
54.H	-0.503079	-0.264500	-4.310296
55.H	0.966035	-5.529345	-3.998925
56.H	2.139404	4.815351	-3.598938
57.H	-0.429113	-6.461226	-3.421110
58.H	0.027136	-0.472469	-2.633693
59.H	3.575491	-1.067813	-3.258595
60.H	4.330157	2.840154	-3.262997
61.H	-6.466977	-0.262848	-2.657755
62.H	3.176480	1.604068	-2.762465
63.H	2.006358	-1.849953	-2.972600
64.H	-3.254262	-1.631364	-2.780197
65.H	-2.828571	4.267990	-2.654964
66.H	-1.020028	-1.699671	-3.387860
67.H	-2.338294	-4.589649	-2.393092
68.H	-5.545201	-1.723770	-2.249364
69.H	1.912805	-4.170956	-2.267989
70.H	4.859180	1.548397	-2.166518
71.H	2.385495	-0.327207	-2.152327
72.H	-5.200445	1.998464	-1.646695
73.H	3.717169	-3.050745	-1.672288
74.H	5.398786	-1.188415	-1.638150

75.H	-4.035130	3.967993	-1.385006
76.H	-6.023222	-0.584574	-0.975475
77.H	-2.295289	3.828010	-1.033709
78.H	0.547659	5.805926	-0.955116
79.H	-0.052405	4.532651	0.116741
80.H	4.443376	-0.361386	-0.382140
81.H	-2.543868	-3.178826	-0.394918
82.H	5.471738	2.660365	-0.210691
83.H	4.050842	-4.673282	-0.210783
84.H	5.204637	-1.926708	-0.040036
85.H	2.468449	-5.277667	0.336698
86.H	-2.778052	0.417810	0.464951
87.H	0.748498	5.969323	0.812171
88.H	3.879071	-5.356266	1.414383
89.H	-4.378901	0.871188	1.084483
90.H	3.950934	-2.848238	1.516246
91.H	-3.700746	-0.731253	1.472457
92.H	-1.296418	-4.665417	1.488019
93.H	6.052325	4.742537	1.831928
94.H	2.761261	5.314581	1.786332
95.H	-1.896799	2.570287	1.490443
96.H	5.607696	3.101344	2.337808
97.H	-3.569601	2.885132	2.022933
98.H	1.198837	-3.985459	2.280396
99.H	4.717605	4.500508	2.977014
100.H	1.990267	-2.556250	2.983320
101.H	2.692315	-4.167769	3.226266
102.H	-2.721002	-5.457837	3.296216
103.H	-2.236052	2.979126	3.188698
104.H	-3.516982	0.817108	3.428875
105.H	0.410841	2.084581	4.346581
106.H	-3.089517	-1.469649	4.790213
107.H	-1.264340	2.516142	4.765286
108.H	-4.084434	-5.108331	5.199885
109.H	1.154919	-0.427692	4.871485
110.H	-4.921534	-3.545849	5.228273
111.H	-0.127062	-1.628216	5.180359
112.H	-1.817267	0.202127	5.262630
113.H	-3.516988	-3.813484	6.270849
114.H	-0.152258	1.971258	6.020068
115.H	0.259268	-0.395209	6.402338
116.Li	2.371491	2.274580	0.782476
117.N	-0.524140	-2.102581	1.034075
118.P	0.345816	1.481442	-0.410607
119.P	2.208726	-2.226029	0.036473
120.P	-1.435000	0.131148	2.801509
121.Sc	0.631964	-0.284420	1.094612
122.Br	2.692277	0.589397	2.592723

Energy: –809.58828755 eV

Table S31. Coordinates and energy for 21

1.C	2.713994	-2.777527	-6.890900
2.C	2.516943	-2.076624	-5.550855
3.C	3.557903	-1.354404	-4.971240
4.C	-1.763153	-0.302673	-4.943733
5.C	1.312258	-2.080017	-4.876737
6.C	-1.780996	-1.505786	-4.016540
7.C	3.405674	-0.671576	-3.776196
8.C	1.818621	2.774744	-3.761360
9.C	1.130108	-1.388264	-3.686322
10.C	-3.127724	-1.676582	-3.313709
11.C	2.179768	-0.667602	-3.098757
12.C	-0.574226	-4.241954	-2.857001
13.C	3.353383	4.326917	-2.723736
14.C	1.998750	3.656304	-2.630110
15.C	-0.427075	-3.064241	-1.921934
16.C	-5.425900	0.986176	-1.312940
17.C	-6.290032	-3.107967	-1.053682
18.C	3.078255	0.457569	-1.161565
19.C	0.839329	-3.178033	-1.063382
20.C	4.118012	-0.400204	-0.851814
21.C	3.141608	1.788584	-0.698759
22.C	-5.096989	2.809640	-0.186551
23.C	0.676351	5.216060	-0.188756
24.C	-5.172166	-2.849991	-0.075972
25.C	5.189961	0.024933	-0.100706
26.C	-4.059510	-3.648825	-0.058522
27.C	4.238603	2.170525	0.060989
28.C	1.701369	4.168966	0.199917
29.C	5.314688	1.329197	0.359080
30.C	-5.249516	-1.791214	0.832817
31.C	-3.005749	-3.422630	0.840958
32.C	-1.848740	-4.366167	0.900879
33.C	6.475903	1.781019	1.174107
34.C	-4.490150	3.386550	1.271869
35.C	1.494639	3.657477	1.521394
36.C	-4.231782	-1.524914	1.722627
37.C	-3.071394	-2.338381	1.715217
38.C	-2.609826	3.417349	2.467915
39.C	2.577940	-1.509104	2.363788
40.C	-0.964467	-1.138701	2.522250
41.C	-4.344820	-0.412702	2.735937
42.C	-1.986541	-2.118380	2.709247
43.C	2.325032	-0.222887	3.076877
44.C	3.337640	0.691485	3.285751
45.C	-0.030524	-0.981943	3.574932
46.C	1.038204	0.042626	3.587354
47.C	-2.018361	-2.893994	3.860925
48.C	3.121374	1.897078	3.928870
49.C	4.250518	2.894218	4.109842

50.C	0.813458	1.252027	4.274263
51.C	1.862710	2.133714	4.429577
52.C	-0.103829	-1.792333	4.697701
53.C	-1.082396	-2.749206	4.864627
54.C	-0.529388	1.572681	4.810509
55.H	2.723231	-2.055521	-7.722685
56.H	1.907367	-3.496874	-7.087237
57.H	3.667502	-3.323402	-6.920570
58.H	-2.552262	-0.404712	-5.706649
59.H	4.528610	-1.315237	-5.474122
60.H	-0.802273	-0.203568	-5.466563
61.H	0.472662	-2.631397	-5.308362
62.H	1.909370	3.330858	-4.711199
63.H	-1.588091	-2.410595	-4.618310
64.H	-1.948687	0.628606	-4.389533
65.H	-3.934276	-1.756626	-4.060278
66.H	3.440147	4.857380	-3.686115
67.H	2.572171	1.973692	-3.798341
68.H	0.832914	2.284903	-3.779225
69.H	0.261998	-4.299676	-3.570496
70.H	4.251670	-0.117412	-3.370842
71.H	-1.513908	-4.226361	-3.427918
72.H	-3.163129	-2.571329	-2.678507
73.H	1.210716	4.429429	-2.649576
74.H	4.163485	3.583197	-2.679888
75.H	-3.338113	-0.807101	-2.674880
76.H	-0.565702	-5.182288	-2.280628
77.H	-5.983373	-2.882794	-2.087153
78.H	3.533537	5.066989	-1.928580
79.H	-5.019664	1.510917	-2.185036
80.H	1.744444	-3.141812	-1.686777
81.H	-6.503154	1.146952	-1.190096
82.H	-5.179782	-0.079028	-1.332317
83.H	-6.603889	-4.161786	-1.037723
84.H	-7.172724	-2.495354	-0.827298
85.H	-1.295502	-3.008076	-1.246812
86.H	0.933233	5.754553	-1.109672
87.H	4.065989	-1.437398	-1.183702
88.H	-4.624247	3.367187	-1.022635
89.H	-3.990847	-4.501913	-0.740632
90.H	0.841404	-4.137820	-0.524741
91.H	-6.191107	2.999990	-0.191957
92.H	-0.322246	4.770562	-0.322482
93.H	0.888606	-2.369312	-0.321805
94.H	5.976528	-0.699379	0.136775
95.H	-1.808062	-5.010680	0.012609
96.H	2.700526	4.639597	0.128235
97.H	7.381911	1.906624	0.558793
98.H	4.282700	3.196939	0.438405
99.H	0.596316	5.967596	0.613799

100.H	-6.151910	-1.173376	0.856196
101.H	-0.894567	-3.834827	0.999748
102.H	-4.555399	4.490153	1.325760
103.H	6.278538	2.743878	1.661670
104.H	6.729376	1.054140	1.960049
105.H	-1.926405	-5.027490	1.778809
106.H	0.519705	3.151097	1.622506
107.H	1.920195	-1.597578	1.486555
108.H	2.240300	2.909810	1.819480
109.H	-5.107219	2.946143	2.079025
110.H	3.617316	-1.580401	2.019471
111.H	1.523272	4.460586	2.279396
112.H	-1.587937	3.023430	2.518911
113.H	-5.250887	0.184112	2.569119
114.H	-2.567785	4.523793	2.482228
115.H	-3.467413	0.250222	2.692943
116.H	4.334372	0.458127	2.902458
117.H	2.365814	-2.377163	3.006874
118.H	4.354081	3.544112	3.226146
119.H	-3.151732	3.085994	3.375473
120.H	-4.381160	-0.815809	3.759408
121.H	-2.819591	-3.630773	3.965120
122.H	5.212547	2.385897	4.256986
123.H	-1.321137	1.295147	4.102430
124.H	4.072397	3.547520	4.974674
125.H	1.673861	3.074641	4.959125
126.H	-0.622666	2.643345	5.042085
127.H	0.643910	-1.644235	5.481972
128.H	-1.116526	-3.368569	5.761472
129.H	-0.742831	1.020543	5.741016
130.N	1.934895	0.040911	-1.905783
131.O	-4.741246	1.591797	-0.056761
132.O	-3.194751	2.952705	1.330834
133.P	-0.433402	-1.409741	-2.753171
134.P	1.672037	2.794839	-1.010109
135.P	-0.877830	-0.112203	1.019589
136.Sc	0.045711	0.614372	-1.042215
137.Br	-1.882509	2.091208	-1.978856
138.Li	-2.825421	1.252322	0.251739

Energy: –907.04718334 eV

Table S32. Coordinates and energy for 22

1.C	2.170738	-6.403815	-4.601867
2.C	3.515413	-6.300198	-4.519570
3.C	-5.260482	4.693454	-3.945748
4.C	1.376145	-5.624145	-3.797920
5.C	4.107613	-5.390530	-3.630265
6.C	-0.074134	1.911644	-3.876777
7.C	-3.368137	-1.826237	-3.755547
8.C	2.202106	1.172830	-3.767545

9.C	-3.470226	3.063567	-3.610489
10.C	0.303671	3.339217	-3.553961
11.C	0.437101	-0.415457	-3.538399
12.C	-5.442386	-0.553059	-3.300303
13.C	-4.504599	3.865838	-2.928005
14.C	1.932857	-4.740589	-2.901226
15.C	3.326580	-4.609100	-2.831078
16.C	-4.137463	-1.029649	-2.722090
17.C	0.084531	5.847789	-2.428973
18.C	-3.605263	5.982597	-1.808447
19.C	-0.191092	-4.078496	-2.056647
20.C	5.230155	-3.599108	-1.778667
21.C	-3.914905	4.619703	-1.747462
22.C	0.557681	4.664982	-1.604177
23.C	5.115027	2.104624	-1.376602
24.C	-4.457310	-3.143613	-1.343892
25.C	-4.343658	-1.755778	-1.405709
26.C	-3.034033	6.633522	-0.740855
27.C	-0.764273	-3.176619	-1.017367
28.C	5.482836	-2.523665	-0.760425
29.C	-3.633832	3.963235	-0.536544
30.C	4.610073	3.069564	-0.326760
31.C	-5.451058	2.370636	-0.278019
32.C	0.921703	4.953927	-0.293810
33.C	-4.684837	-3.787293	-0.158190
34.C	-5.675003	1.059785	-0.181442
35.C	5.697925	4.110958	-0.041698
36.C	-4.456634	-1.039563	-0.196844
37.C	-2.745549	5.966715	0.410973
38.C	-3.046453	4.609271	0.553989
39.C	1.601995	4.157168	0.638372
40.C	4.175909	2.344975	0.933219
41.C	-4.783952	-3.076416	1.018745
42.C	-4.670281	-1.689232	1.030678
43.C	2.832157	2.258445	1.330342
44.C	-0.961513	-2.897016	1.313163
45.C	5.252767	-2.029934	1.527786
46.C	5.138925	1.727400	1.724135
47.C	2.159160	4.867822	1.842985
48.C	-2.780928	3.884682	1.851126
49.C	-4.750311	-0.890006	2.315503
50.C	2.472512	1.578650	2.506092
51.C	-4.013278	3.867443	2.720311
52.C	-1.615322	4.437935	2.659085
53.C	-0.492655	-3.454267	2.618624
54.C	4.877277	-2.632290	2.831427
55.C	4.805070	1.049096	2.874526
56.C	-3.358696	-0.427170	2.764394
57.C	1.050800	1.488476	2.984864
58.C	0.580851	0.037885	3.039406

59.C	3.483078	0.974597	3.256389
60.C	-5.431234	-1.616164	3.470549
61.C	1.538219	-3.689641	3.871287
62.C	2.914060	-3.482163	3.924096
63.C	0.883715	2.141688	4.363237
64.C	0.877159	-4.229958	4.950845
65.C	3.615850	-3.819482	5.067272
66.C	2.938370	-4.367220	6.140449
67.C	1.601999	-4.564536	6.101561
68.H	1.707483	-7.104900	-5.297396
69.H	4.163441	-6.915334	-5.145483
70.H	-5.727113	4.032056	-4.690481
71.H	-0.100119	1.763550	-4.976940
72.H	-4.596949	5.379276	-4.498471
73.H	2.228828	1.090720	-4.872183
74.H	-3.152003	-1.196582	-4.631271
75.H	0.447605	-0.619759	-4.628734
76.H	-3.912028	2.414468	-4.384908
77.H	-5.273528	0.013520	-4.230728
78.H	-2.735861	3.713861	-4.117265
79.H	-3.934209	-2.697941	-4.123286
80.H	1.164969	3.667560	-4.165289
81.H	0.293237	-5.714158	-3.860836
82.H	5.193018	-5.327510	-3.582577
83.H	-6.057022	5.301042	-3.490088
84.H	-0.544729	3.974456	-3.846152
85.H	-6.104230	-1.401249	-3.546601
86.H	-1.071894	1.701252	-3.469333
87.H	2.566334	2.163467	-3.473680
88.H	0.678094	5.962604	-3.348638
89.H	2.873412	0.419237	-3.339133
90.H	-2.407739	-2.189507	-3.363129
91.H	1.117645	-1.111594	-3.035014
92.H	-0.576543	-0.569534	-3.146290
93.H	-0.586623	-3.798819	-3.047153
94.H	-2.920148	2.423060	-2.904637
95.H	-3.827969	6.541231	-2.720571
96.H	-0.967686	5.717599	-2.723343
97.H	5.664984	-3.304660	-2.747413
98.H	-5.982939	0.105492	-2.606568
99.H	-5.233525	3.156043	-2.503933
100.H	-3.524691	-0.145721	-2.480255
101.H	5.350602	2.630469	-2.315935
102.H	-4.385896	-3.727784	-2.264168
103.H	0.159572	6.777012	-1.855563
104.H	-0.465707	-5.126111	-1.848226
105.H	5.667296	-4.558547	-1.458380
106.H	4.375900	1.320671	-1.590821
107.H	6.039724	1.605667	-1.040961
108.H	-2.810525	7.700242	-0.822231

109.H	5.950159	4.664072	-0.959916
110.H	4.919388	-1.610283	-1.026013
111.H	-1.864756	-3.185735	-1.108906
112.H	-0.439526	-2.128206	-1.149332
113.H	6.562664	-2.275675	-0.759511
114.H	3.733831	3.591601	-0.733244
115.H	-6.166576	3.186230	-0.251881
116.H	-4.788658	-4.874504	-0.141018
117.H	-6.617791	0.532460	-0.079580
118.H	0.771242	5.987121	0.014282
119.H	6.623996	3.637800	0.319617
120.H	5.382917	4.843937	0.715085
121.H	-2.284823	6.501427	1.243517
122.H	6.190061	1.793166	1.430390
123.H	4.640060	-1.136034	1.316067
124.H	-2.062927	-2.973557	1.272399
125.H	6.310645	-1.706680	1.580011
126.H	-0.708465	-1.828265	1.200939
127.H	3.255732	4.940725	1.787028
128.H	-2.521860	2.847884	1.583287
129.H	-4.970504	-3.611707	1.951262
130.H	1.757109	5.885054	1.915852
131.H	-5.334969	0.017216	2.097257
132.H	-0.699220	4.500095	2.057120
133.H	-2.791497	0.055845	1.954917
134.H	-4.871696	3.399628	2.216429
135.H	0.687176	-0.433137	2.054874
136.H	0.407716	2.015267	2.265996
137.H	1.930895	4.329961	2.772126
138.H	-4.309256	4.891431	3.006009
139.H	-0.768655	-4.518990	2.706406
140.H	5.456567	-3.553358	3.014754
141.H	-1.829778	5.440972	3.063846
142.H	-6.436680	-1.977922	3.206983
143.H	-2.772873	-1.290386	3.122400
144.H	5.585498	0.599502	3.493065
145.H	-1.418416	3.783830	3.519587
146.H	-0.966343	-2.893177	3.440324
147.H	-0.480150	-0.008887	3.324441
148.H	5.081210	-1.913230	3.640937
149.H	-3.828375	3.305920	3.650840
150.H	-3.444703	0.280725	3.603528
151.H	-4.842674	-2.481150	3.815474
152.H	1.162180	-0.546233	3.769638
153.H	-5.534032	-0.937048	4.329533
154.H	3.215606	0.448313	4.175484
155.H	1.191671	3.196872	4.369261
156.H	-0.168568	2.098185	4.682625
157.H	-0.197407	-4.392280	4.917839
158.H	4.691001	-3.662388	5.122677

159.H	1.479935	1.617875	5.126709
160.H	3.503450	-4.632280	7.036075
161.H	1.074564	-4.991253	6.955333
162.B	-3.351973	1.340686	-0.365152
163.Cl	2.226651	-0.326465	-0.608177
164.K	2.318260	-3.150209	0.330411
165.N	0.841049	0.943218	-3.255003
166.N	0.610369	3.455739	-2.123606
167.N	-4.078137	2.608775	-0.385380
168.N	-4.452274	0.385848	-0.234403
169.N	1.818383	2.848639	0.473664
170.O	1.231522	-3.943126	-2.046118
171.O	3.797861	-3.721188	-1.896505
172.O	-0.370577	-3.667253	0.263964
173.O	5.084242	-3.009412	0.506686
174.O	0.929188	-3.321767	2.690283
175.O	3.477271	-2.940879	2.792227
176.P	-1.532514	0.924774	-0.444973
177.Sc	0.689314	1.598653	-0.973640

Energy: −1184.96156830 eV

Table S33. Coordinates and energy for 23

1.C	3.709400	-0.151686	-5.065642
2.C	1.391496	0.664479	-4.561283
3.C	2.724866	0.182629	-3.968131
4.C	1.508008	-4.921500	-3.646964
5.C	-0.251178	-3.172997	-3.632163
6.C	3.643939	2.468434	-3.420093
7.C	3.304376	1.183853	-2.991584
8.C	0.951123	-3.718542	-2.887469
9.C	4.225290	3.369639	-2.563085
10.C	-0.582212	3.862326	-2.321686
11.C	3.562857	0.861202	-1.651713
12.C	0.604281	-4.069661	-1.456959
13.C	4.497809	3.031622	-1.263357
14.C	-0.320282	-5.069636	-1.173832
15.C	4.128599	-1.479040	-1.125440
16.C	-3.276085	-2.160430	-0.870856
17.C	4.187149	1.762686	-0.779969
18.C	3.500829	-2.617681	-0.793527
19.C	1.133544	3.530090	-0.761956
20.C	1.203786	-3.410837	-0.377246
21.C	-0.792574	4.658565	-0.050784
22.C	-0.625704	-5.401562	0.119246
23.C	4.516497	1.361796	0.646570
24.C	-5.653197	-1.018147	0.800939
25.C	-3.317771	-1.928956	0.636160
26.C	-6.570208	-0.032559	1.048749

27.C	5.824563	1.982414	1.155040
28.C	-4.287365	-0.812867	0.972205
29.C	0.904087	-3.741266	0.949662
30.C	-0.023130	-4.751815	1.171008
31.C	-5.103827	3.978815	1.379548
32.C	-6.150367	1.209879	1.464090
33.C	-3.641454	-3.247775	1.353264
34.C	-0.225474	4.279172	1.411420
35.C	-3.876772	0.447259	1.413640
36.C	-4.794224	1.470769	1.645882
37.C	3.369223	1.691677	1.589471
38.C	-4.364580	2.859194	2.099028
39.C	1.571818	-3.034488	2.096425
40.C	2.709926	-3.904667	2.673392
41.C	-1.961544	0.506518	2.830273
42.C	-0.248569	2.322052	2.853712
43.C	0.568196	-2.704336	3.226357
44.C	-4.483966	3.000998	3.603970
45.C	-0.809300	1.142478	3.311066
46.C	-2.669281	-0.439362	3.769230
47.C	0.653339	3.063926	3.816712
48.H	3.295813	-0.923059	-5.733523
49.H	3.943279	0.727888	-5.687135
50.H	0.997951	-0.083922	-5.265760
51.H	1.516855	1.607621	-5.117408
52.H	4.659785	-0.532874	-4.662496
53.H	1.799455	-4.628502	-4.667364
54.H	0.019661	-2.918271	-4.668891
55.H	3.457604	2.753433	-4.457811
56.H	0.757883	-5.722733	-3.736176
57.H	-1.063300	-3.915884	-3.680394
58.H	0.640351	0.807405	-3.771820
59.H	2.514160	-0.738409	-3.409526
60.H	2.393439	-5.348256	-3.152287
61.H	4.484007	4.367022	-2.926492
62.H	-0.647362	-2.264537	-3.156753
63.H	1.726296	-2.942036	-2.865731
64.H	-0.248294	3.014316	-2.930757
65.H	-0.040920	4.771406	-2.648077
66.H	-1.656443	3.996595	-2.499297
67.H	-0.807788	-5.598226	-1.996349
68.H	5.179748	-1.311028	-1.333128
69.H	1.556219	2.924054	-1.571912
70.H	-3.044809	-1.235547	-1.416874
71.H	-4.249877	-2.524509	-1.236329
72.H	-2.515664	-2.913326	-1.125600
73.H	1.568595	4.543499	-0.826154
74.H	4.985462	3.755766	-0.608687
75.H	3.914239	-3.612282	-0.666735
76.H	-0.605715	-1.282203	-0.719814

77.H	-0.390591	5.640521	-0.363145
78.H	-1.890680	4.700901	-0.032990
79.H	-1.351716	-6.192811	0.318019
80.H	-5.033963	3.862611	0.289043
81.H	1.418844	3.057957	0.179311
82.H	6.663011	1.785353	0.471912
83.H	-6.000312	-1.994099	0.452768
84.H	-7.634709	-0.229596	0.903967
85.H	4.642243	0.270052	0.658150
86.H	-4.609079	-3.654619	1.021399
87.H	5.742510	3.072702	1.282286
88.H	-2.313015	-1.611905	0.951243
89.H	-2.868811	-3.995006	1.125910
90.H	-6.882847	1.998346	1.644168
91.H	-6.170126	4.015067	1.650051
92.H	-4.674432	4.955505	1.653716
93.H	2.431771	1.212426	1.272972
94.H	0.848548	4.495732	1.470637
95.H	3.210799	2.781792	1.634452
96.H	6.081976	1.562960	2.139147
97.H	3.465550	-4.141579	1.911018
98.H	-3.298953	2.960371	1.849643
99.H	1.997651	-2.093824	1.726123
100.H	-0.279991	-5.036522	2.192708
101.H	-0.730069	4.930027	2.145037
102.H	-3.693841	-3.130272	2.446000
103.H	3.595188	1.347559	2.611348
104.H	2.311219	-4.855054	3.061145
105.H	-0.273706	-2.109525	2.848031
106.H	3.216099	-3.383735	3.501271
107.H	1.590494	3.377940	3.335866
108.H	-2.760805	-1.437168	3.320809
109.H	0.171894	-3.616375	3.695410
110.H	-5.527643	2.866606	3.932062
111.H	-4.160091	4.002198	3.933849
112.H	-3.870017	2.262692	4.138471
113.H	-3.692536	-0.093794	3.973472
114.H	1.068729	-2.122706	4.015418
115.H	0.156004	3.974870	4.183834
116.H	-0.436011	0.779182	4.267331
117.H	0.899239	2.441559	4.683276
118.H	-2.130153	-0.527273	4.719102
119.B	1.891417	-0.965400	-0.877276
120.Cl	-3.138334	1.912122	-1.410109
121.N	3.193658	-0.438564	-1.174204
122.N	-0.340147	3.585560	-0.930839
123.N	2.141381	-2.361694	-0.631499
124.N	-2.471337	0.759597	1.616863
125.N	-0.554149	2.855467	1.670975
126.P	0.198580	-0.137324	-1.003258

127.Sc	-1.276903	1.668935	0.061339

Energy: –839.25433813 eV

Table S34. Coordinates and energy for 24

1.Ti	-0.528361	-0.901447	-0.267977
2.P	0.720297	0.328674	1.033563
3.N	-0.508341	-0.174954	-2.185551
4.N	-2.527479	-0.537510	-0.004953
5.C	1.957086	-1.277934	-5.450222
6.C	0.729954	-1.875128	-5.267967
7.C	-2.496611	-1.642271	-4.849792
8.C	2.427239	-0.386119	-4.508841
9.C	-1.292004	-3.768369	-4.275046
10.C	-0.071467	-1.567121	-4.165974
11.C	-1.402607	-2.273407	-3.989458
12.C	-1.044784	1.766835	-3.618296
13.C	1.669744	-0.028497	-3.393088
14.C	0.390827	-0.595061	-3.256412
15.C	2.796525	2.211652	-2.995448
16.C	-1.287784	0.893543	-2.404818
17.C	2.292832	0.908199	-2.370654
18.C	-5.554615	-2.160219	-1.950613
19.C	-3.847639	-3.977045	-1.728452
20.C	3.442281	0.202476	-1.644976
21.C	-2.364131	1.271632	-1.584366
22.C	-4.194162	-2.537459	-1.352296
23.C	0.138087	3.459267	-0.563841
24.C	-3.024709	0.569740	-0.564596
25.C	-0.097128	-2.938377	-0.243417
26.C	-4.359539	1.110730	-0.112317
27.C	0.274311	-5.424094	0.022966
28.C	-4.190171	-2.340044	0.160217
29.C	5.390081	5.056402	0.972295
30.C	0.050003	-4.092599	0.755921
31.C	-5.022840	-3.137245	0.937391
32.C	-3.404686	-1.366872	0.798173
33.C	0.236858	3.627464	0.960915
34.C	0.150476	5.139495	1.247232
35.C	1.571912	3.020037	1.468208
36.C	-1.219543	-4.206690	1.595280
37.C	2.610934	3.916761	1.704517
38.C	1.232263	-3.825978	1.681382
39.C	-0.984364	2.997100	1.642845
40.C	3.661109	-1.140335	1.773649
41.C	1.815968	1.625761	1.710103
42.C	4.952005	4.600519	2.371782
43.C	3.847215	3.550979	2.216154
44.C	-5.084532	-2.992558	2.310197
45.C	-3.460324	-1.198839	2.194261
46.C	3.026960	1.253662	2.377072

47.C	4.001543	2.229594	2.584231
48.C	6.185333	4.040332	3.094466
49.C	4.424117	5.800826	3.167225
50.C	-4.307661	-2.031064	2.927432
51.C	3.333387	-0.170763	2.916574
52.C	-2.644249	-0.152552	2.936561
53.C	-3.541633	0.803120	3.728357
54.C	4.561761	-0.173206	3.846486
55.C	2.171988	-0.683603	3.775950
56.C	-1.614849	-0.809290	3.853502
57.H	2.565045	-1.527222	-6.321454
58.H	0.377981	-2.607801	-5.995359
59.H	-2.233887	-1.695983	-5.918152
60.H	-1.049291	-3.965915	-5.329464
61.H	-3.453271	-2.169731	-4.713305
62.H	-2.660370	-0.585616	-4.596510
63.H	3.419253	0.051096	-4.634060
64.H	-1.011004	1.169701	-4.539681
65.H	-2.244483	-4.272961	-4.067189
66.H	-0.515840	-4.240571	-3.657013
67.H	3.611107	2.029522	-3.712706
68.H	-1.826844	2.526581	-3.716504
69.H	-0.072892	2.273034	-3.535881
70.H	2.004189	2.756703	-3.529195
71.H	-5.540609	-2.274608	-3.045275
72.H	-1.697110	-2.152985	-2.938657
73.H	-3.901224	-4.111695	-2.817673
74.H	3.191209	2.873879	-2.211484
75.H	4.263059	-0.036245	-2.340262
76.H	-5.829712	-1.119832	-1.727462
77.H	-6.353207	-2.807630	-1.557501
78.H	-2.849394	2.198260	-1.881794
79.H	-3.438218	-1.871692	-1.786605
80.H	1.535880	1.149312	-1.615616
81.H	-2.839619	-4.258589	-1.396624
82.H	-4.556633	-4.688370	-1.279484
83.H	3.106551	-0.733731	-1.180743
84.H	0.988921	3.947092	-1.062597
85.H	3.841371	0.847033	-0.848345
86.H	-0.792171	3.915932	-0.939366
87.H	-0.917394	-3.183144	-0.965592
88.H	-0.573869	-5.662410	-0.639303
89.H	-4.700109	1.919256	-0.768145
90.H	0.134302	2.396325	-0.829901
91.H	0.827013	-2.848524	-0.867335
92.H	1.185238	-5.391056	-0.596065
93.H	-5.126209	0.326149	-0.086277
94.H	5.784435	4.211000	0.389367
95.H	4.550333	5.488556	0.408991
96.H	-5.643064	-3.893467	0.452759

97.H	0.382575	-6.255727	0.739801
98.H	6.179494	5.822711	1.043359
99.H	0.857618	5.730935	0.648598
100.H	-4.278297	1.504980	0.911749
101.H	-0.858329	5.489424	0.981109
102.H	-2.106395	-4.388036	0.968535
103.H	2.176747	-3.776399	1.117354
104.H	4.530463	-0.782093	1.202657
105.H	2.814071	-1.250830	1.086121
106.H	2.444069	4.966573	1.478717
107.H	-1.904020	3.482429	1.279117
108.H	-1.051363	1.924149	1.428024
109.H	0.315919	5.369818	2.309949
110.H	-1.140222	-5.044529	2.308212
111.H	3.903858	-2.134365	2.181162
112.H	-1.402236	-3.291274	2.175072
113.H	1.110687	-2.870093	2.207389
114.H	6.637393	3.204758	2.541568
115.H	1.331381	-4.628306	2.431417
116.H	3.577503	6.288226	2.665076
117.H	-2.090717	0.431197	2.192767
118.H	-5.743382	-3.630769	2.900836
119.H	-0.935133	3.131091	2.734304
120.H	6.948889	4.826802	3.188371
121.H	5.215530	6.557507	3.290466
122.H	4.929604	1.934653	3.060800
123.H	-4.299248	1.283250	3.092111
124.H	5.499208	0.051767	3.317497
125.H	1.237784	-0.748695	3.204957
126.H	-0.928521	-1.452159	3.288515
127.H	5.941088	3.689464	4.107718
128.H	4.087241	5.492990	4.168195
129.H	-4.357577	-1.914616	4.011358
130.H	2.403400	-1.687583	4.164357
131.H	4.671551	-1.181122	4.273467
132.H	-2.937336	1.597443	4.191211
133.H	-4.075837	0.280935	4.536800
134.H	-1.009851	-0.043447	4.360374
135.H	4.453081	0.532204	4.683339
136.H	-2.101593	-1.423160	4.627656
137.H	2.002811	-0.014538	4.633790

Energy: −889.01699025 eV

Table S35. Coordinates and energy for 25

1.C	-1.648526	-1.472408	-4.863106
2.C	-0.470549	2.164439	-4.392233
3.C	0.662596	-1.501012	-3.944573
4.C	-0.540139	4.427515	-3.455020
5.C	-0.819358	-1.367614	-3.582354
6.C	-0.093704	3.012353	-3.172506

7.C	-4.065357	0.765043	-2.981403
8.C	1.400164	2.980944	-3.001047
9.C	-1.118554	-3.738774	-2.835081
10.C	-1.248594	-2.384759	-2.538188
11.C	-4.551126	-1.596867	-2.367163
12.C	-1.534328	-4.724869	-1.961841
13.C	7.798420	-1.658633	-1.558193
14.C	2.373506	-4.114901	-1.933598
15.C	-0.805788	2.438070	-1.944760
16.C	-4.375459	-0.182301	-1.810478
17.C	-5.732779	0.212942	-1.219418
18.C	-1.832538	-2.036454	-1.295512
19.C	2.073001	-3.089251	-0.847726
20.C	-2.051579	-4.371314	-0.737597
21.C	-3.205578	-0.022487	-0.781120
22.C	7.026108	-2.177185	-0.333102
23.C	4.525183	-2.516419	-0.570960
24.C	4.910237	2.592081	-0.472583
25.C	3.199707	-2.077738	-0.631283
26.C	5.594716	-1.661886	-0.343914
27.C	2.947933	-0.695325	-0.465025
28.C	-2.181439	-3.031044	-0.367185
29.C	5.315342	-0.313082	-0.177831
30.C	4.025696	0.198318	-0.239705
31.C	3.755844	1.685642	-0.072903
32.C	-3.369248	0.947369	0.184356
33.C	1.716036	-3.784253	0.457518
34.C	7.766611	-1.826398	0.930083
35.C	0.312977	4.891756	0.622726
36.C	-2.692622	-2.702799	1.023009
37.C	-4.196814	-2.926338	1.172014
38.C	3.319967	2.006844	1.353561
39.C	-0.793369	4.268421	1.470948
40.C	-2.631406	1.354819	1.387374
41.C	-1.587005	5.379860	2.177742
42.C	-4.566946	2.793759	2.160641
43.C	-1.942810	-3.481041	2.102489
44.C	-0.206817	3.264206	2.453555
45.C	-0.455809	1.876712	2.406172
46.C	-3.574582	1.690682	2.567633
47.C	-4.360118	0.399265	2.875398
48.C	1.336950	-1.204933	3.122690
49.C	-0.011392	-0.504906	3.286389
50.C	0.175855	1.004211	3.302021
51.C	0.636411	3.747856	3.454200
52.C	-2.919084	2.140017	3.882823
53.C	1.003128	1.558623	4.279189
54.C	1.229174	2.912505	4.370777
55.C	-0.722959	-1.007650	4.542949
56.H	-1.367767	-0.671229	-5.563136

57.H	-1.465149	-2.432309	-5.370207
58.H	-0.041059	2.591630	-5.314250
59.H	-2.728508	-1.397376	-4.682961
60.H	0.928588	-0.780104	-4.731730
61.H	-0.048492	4.833406	-4.356134
62.H	-1.562609	2.117614	-4.526079
63.H	0.886519	-2.505284	-4.332931
64.H	-0.090045	1.141186	-4.291653
65.H	1.912555	3.357184	-3.902524
66.H	-4.839826	0.659620	-3.757624
67.H	-0.686902	-4.027729	-3.794989
68.H	-1.629327	4.482791	-3.618872
69.H	-3.094321	0.540316	-3.438315
70.H	-5.388316	-1.579379	-3.083156
71.H	-0.967838	-0.367628	-3.151756
72.H	1.308432	-1.305893	-3.079684
73.H	-3.672531	-1.969497	-2.898490
74.H	-0.299910	5.104231	-2.618169
75.H	2.657574	-3.637595	-2.881891
76.H	7.291296	-1.924952	-2.496264
77.H	-4.051867	1.815239	-2.654945
78.H	1.753649	1.959515	-2.801126
79.H	-1.435722	-5.776321	-2.236070
80.H	1.721329	3.613401	-2.157641
81.H	8.816863	-2.077746	-1.585676
82.H	1.487558	-4.740334	-2.114095
83.H	-6.520204	-0.016471	-1.951973
84.H	-1.905352	2.555739	-2.073570
85.H	7.886644	-0.562166	-1.525816
86.H	3.194207	-4.789473	-1.641244
87.H	-4.801813	-2.327716	-1.586952
88.H	5.272941	2.380896	-1.489283
89.H	-5.819063	1.286672	-1.000688
90.H	-0.552691	3.080943	-1.061482
91.H	1.188326	-2.510185	-1.158589
92.H	6.971264	-3.275732	-0.411032
93.H	4.732694	-3.580966	-0.710154
94.H	4.589241	3.644006	-0.440696
95.H	2.901766	1.912470	-0.731808
96.H	-5.964151	-0.346967	-0.301628
97.H	6.140841	0.384083	-0.017559
98.H	-2.349381	-5.151068	-0.034072
99.H	-0.110510	5.553249	-0.147348
100.H	5.764406	2.499587	0.216897
101.H	0.922502	4.129335	0.121251
102.H	-4.326735	1.450843	0.134463
103.H	8.785568	-2.244704	0.920119
104.H	0.882966	-4.487392	0.305478
105.H	-4.780457	-2.281927	0.501625
106.H	7.864356	-0.736339	1.049438

107.H	2.575662	-4.352642	0.849898
108.H	-4.463860	-3.970787	0.945653
109.H	-1.483400	3.743932	0.796773
110.H	0.988964	5.495979	1.247057
111.H	1.417785	-3.058033	1.221508
112.H	-2.493826	-1.636413	1.198129
113.H	7.254130	-2.208156	1.825525
114.H	-2.046571	6.049245	1.434912
115.H	-5.290252	2.468750	1.401613
116.H	3.054383	3.071153	1.448116
117.H	-4.052371	3.688235	1.783382
118.H	2.445803	1.414553	1.651348
119.H	-2.137825	-4.562254	2.037000
120.H	4.137749	1.798403	2.063035
121.H	-4.926195	0.041191	2.006905
122.H	-4.520176	-2.717270	2.203268
123.H	-0.858056	-3.334061	2.026614
124.H	1.820768	-0.928428	2.177773
125.H	-0.931846	5.995533	2.811168
126.H	-2.387972	4.983841	2.815753
127.H	-0.623140	-0.758276	2.410012
128.H	-5.147498	3.093274	3.045601
129.H	-2.269559	-3.156731	3.101086
130.H	-3.687272	-0.407410	3.199404
131.H	1.203500	-2.295861	3.139853
132.H	0.829509	4.820681	3.504646
133.H	-5.069334	0.594545	3.694620
134.H	-2.400455	3.100835	3.801268
135.H	2.022747	-0.952689	3.945360
136.H	-2.209901	1.407388	4.280478
137.H	-3.722837	2.259388	4.626389
138.H	-0.793272	-2.104651	4.528270
139.H	-1.741817	-0.607972	4.641830
140.H	1.487478	0.889741	4.992456
141.H	1.881520	3.313606	5.146986
142.H	-0.169518	-0.726364	5.451868
143.N	-1.993798	-0.656484	-0.921432
144.N	-1.324512	1.358854	1.352231
145.P	1.256937	-0.055570	-0.509873
146.Ti	-0.712250	0.819600	-0.597993

Energy: –943.59910953 eV

Table S36. Coordinates and energy for 26

1.Ti	-1.008932	0.277376	0.846213
2.P	1.077457	0.698341	0.453114
3.N	-2.098051	1.109711	-0.659571
4.N	-1.391450	-1.663017	0.466023
5.C	-2.223958	0.237222	-4.157640
6.C	0.241704	0.616131	-4.166003
7.C	-0.902856	3.211501	-3.489058

8.C	-1.028519	0.697873	-3.336655
9.C	-0.996462	4.469753	-2.950236
10.C	-0.023173	-2.688983	-2.999647
11.C	-1.874578	-4.336590	-2.635586
12.C	-1.227055	2.074072	-2.740172
13.C	3.876025	-0.373785	-2.605929
14.C	-4.523659	2.406135	-2.066750
15.C	-1.090370	-3.190084	-2.011264
16.C	-5.502643	0.173039	-1.772444
17.C	-1.396272	4.635588	-1.650513
18.C	-1.713177	2.267995	-1.444852
19.C	3.247185	-0.719817	-1.269443
20.C	-4.649596	1.240087	-1.073564
21.C	3.439688	-2.191883	-0.933918
22.C	-1.764625	3.547015	-0.859489
23.C	0.450612	-4.674485	-0.705997
24.C	-0.394994	-3.566236	-0.713064
25.C	7.852126	1.630155	-0.058023
26.C	-3.319614	0.603413	-0.550070
27.C	-4.686413	-3.269879	0.001113
28.C	3.754445	0.163703	-0.136997
29.C	5.122632	0.299173	0.076385
30.C	-5.423558	1.761656	0.152083
31.C	-3.525703	-0.604219	0.169272
32.C	1.189999	-5.013085	0.406751
33.C	-0.502200	-2.803208	0.454069
34.C	-2.162853	3.790892	0.582701
35.C	-3.207476	4.907214	0.745067
36.C	-2.732030	-1.728555	0.516921
37.C	2.864655	0.850544	0.721028
38.C	7.148748	1.279549	1.254672
39.C	5.648880	1.110022	1.067895
40.C	-3.586384	-2.929696	1.033933
41.C	-2.843034	-4.239844	1.313238
42.C	-0.925136	4.109936	1.420563
43.C	7.764561	0.048461	1.870763
44.C	1.117396	-4.217883	1.528946
45.C	0.294550	-3.092625	1.577458
46.C	3.375892	1.658451	1.765184
47.C	4.753401	1.776479	1.900605
48.C	-4.206199	-2.490021	2.354725
49.C	2.529739	3.923910	2.478190
50.C	2.465653	2.422745	2.708594
51.C	0.280153	-2.270800	2.852137
52.C	-1.564020	0.875136	2.840237
53.C	1.675792	-1.796413	3.240596
54.C	-0.348112	-3.043247	4.026019
55.C	2.725007	2.068912	4.162953
56.H	0.180451	1.233830	-5.074716
57.H	-2.420114	0.929450	-4.992439

58.H	-2.039724	-0.758472	-4.589418
59.H	0.421291	-0.414703	-4.496947
60.H	-0.546469	3.092476	-4.512540
61.H	-0.487293	-2.374342	-3.945843
62.H	1.118053	0.947473	-3.592810
63.H	-0.729202	5.339747	-3.553162
64.H	-3.140131	0.171670	-3.557161
65.H	-2.396517	-3.996221	-3.542959
66.H	3.426707	-0.975219	-3.411634
67.H	0.693760	-3.491446	-3.230335
68.H	-1.213247	-5.163601	-2.934521
69.H	-3.968492	2.137618	-2.973197
70.H	3.740994	0.688275	-2.857934
71.H	4.957948	-0.580970	-2.614789
72.H	-4.973850	-0.279970	-2.623075
73.H	-5.544231	2.683411	-2.375291
74.H	-0.913632	0.005494	-2.489618
75.H	0.539815	-1.844215	-2.584417
76.H	-6.415946	0.646103	-2.163596
77.H	-2.627575	-4.751172	-1.950566
78.H	-4.058423	3.296883	-1.635765
79.H	-1.782720	-2.360976	-1.815271
80.H	3.043951	-2.832198	-1.737669
81.H	0.537252	-5.281445	-1.609458
82.H	-1.430727	5.638893	-1.222236
83.H	-5.818625	-0.639869	-1.108586
84.H	2.161926	-0.531347	-1.340470
85.H	7.777276	0.805442	-0.782165
86.H	-4.254077	-3.475228	-0.987353
87.H	7.416291	2.525203	-0.524104
88.H	4.509549	-2.429885	-0.813281
89.H	5.804532	-0.241854	-0.586118
90.H	8.923466	1.818798	0.113517
91.H	-6.372595	2.208540	-0.182653
92.H	-5.432820	-2.474691	-0.115455
93.H	2.925353	-2.463096	-0.001918
94.H	-4.103863	4.736757	0.132498
95.H	-5.221679	-4.173037	0.331121
96.H	-2.794148	5.886832	0.463912
97.H	1.840931	-5.888188	0.390889
98.H	-4.568708	-0.754102	0.417495
99.H	-2.420494	-4.695156	0.409966
100.H	-4.858320	2.536272	0.686814
101.H	-5.664256	0.964538	0.868786
102.H	7.642661	-0.825006	1.210760
103.H	-2.604518	2.866501	0.979172
104.H	-0.454380	5.042165	1.072674
105.H	7.290204	2.123476	1.950139
106.H	2.299610	4.178809	1.434119
107.H	-0.180028	3.306171	1.347299

108.H	-3.521668	4.977588	1.797046
109.H	8.844647	0.184373	2.043747
110.H	-3.574165	-4.950046	1.731335
111.H	-2.037027	-4.131764	2.044990
112.H	1.723306	-4.465766	2.402197
113.H	-4.848320	-1.604080	2.257905
114.H	7.297319	-0.202377	2.834363
115.H	-1.192462	4.240008	2.480305
116.H	5.155223	2.421170	2.688404
117.H	-4.825404	-3.305190	2.761401
118.H	2.148010	-1.223921	2.432406
119.H	1.440594	2.093475	2.470291
120.H	3.532554	4.321604	2.704986
121.H	-0.335848	-1.374920	2.686050
122.H	-1.849583	1.940626	2.858268
123.H	-3.431174	-2.257151	3.098768
124.H	1.809905	4.448792	3.125330
125.H	-2.444726	0.296432	3.180241
126.H	-0.767260	0.751056	3.590737
127.H	2.332146	-2.642147	3.495952
128.H	-1.387034	-3.339454	3.829083
129.H	1.618120	-1.149355	4.128356
130.H	0.224967	-3.957407	4.244526
131.H	2.653293	0.985462	4.332191
132.H	3.727450	2.389909	4.489818
133.H	1.993300	2.565566	4.819555
134.H	-0.344632	-2.420660	4.933389

Energy: –870.56755009 eV

Table S37. Coordinates and energy for 27

1.Ti	-0.416105	0.488322	-0.938564
2.P	1.561727	0.854485	-1.702061
3.F	-0.767329	-6.013820	0.163803
4.F	0.319602	-3.613655	0.522180
5.F	2.347827	-0.841283	1.218230
6.F	-2.770915	-6.886012	1.787497
7.F	4.743163	-1.368061	2.179584
8.F	-3.125042	-0.822942	2.317387
9.F	-3.590398	-5.309697	3.857148
10.F	-4.552517	0.672593	3.968749
11.F	-2.503458	-2.959551	4.282915
12.F	5.045704	-2.741742	4.523072
13.F	0.398562	-3.095887	4.903403
14.F	0.655247	-0.249076	5.149855
15.F	2.848699	-3.581665	5.865466
16.F	-3.455776	1.609344	6.294585
17.F	-0.839640	1.083369	6.830509
18.N	-1.547822	-0.123128	-2.435114
19.N	-1.191538	2.359042	-0.679518
20.C	-0.828990	-1.720816	-6.278384

21.C	-1.486070	-0.515868	-6.132389
22.C	6.986108	2.657446	-5.630872
23.C	-1.971415	2.342849	-5.885887
24.C	1.964026	1.274095	-5.901157
25.C	2.931407	-0.947104	-5.204669
26.C	-0.375655	-2.391555	-5.159585
27.C	-1.718374	0.038191	-4.871496
28.C	-3.907272	1.325692	-4.694821
29.C	-2.383902	1.403548	-4.750363
30.C	2.370130	0.391438	-4.720526
31.C	7.020446	2.244125	-4.175177
32.C	4.619719	1.410913	-4.358532
33.C	7.960976	1.063070	-3.978312
34.C	3.388396	1.050771	-3.811986
35.C	-0.596219	-1.904876	-3.871112
36.C	0.755834	4.278045	-3.733276
37.C	5.654284	1.921275	-3.588551
38.C	-1.296068	-0.691958	-3.739107
39.C	-1.576548	5.136959	-3.416014
40.C	-3.875429	-2.034089	-3.212786
41.C	-0.504516	4.192947	-2.878363
42.C	-0.753917	-4.158419	-2.734311
43.C	-0.131005	-2.753564	-2.691781
44.C	1.388284	-2.881666	-2.624411
45.C	3.172030	1.240391	-2.426818
46.C	5.427037	2.077745	-2.218222
47.C	-3.785352	-1.331701	-1.848825
48.C	-2.742481	-0.162519	-1.769032
49.C	4.220485	1.747883	-1.614706
50.C	-5.213587	-0.848225	-1.523890
51.C	-0.189291	4.486494	-1.423157
52.C	-4.622702	3.170745	-1.162074
53.C	0.427290	5.695213	-1.099624
54.C	-3.372049	-2.363241	-0.784782
55.C	-2.998472	0.809566	-0.794665
56.C	-2.479190	2.138785	-0.509693
57.C	-0.499217	3.605230	-0.379475
58.C	4.032719	1.977905	-0.126349
59.C	-3.574521	3.130031	-0.035405
60.C	0.772124	6.000517	0.200811
61.C	3.699042	3.440791	0.136822
62.C	-3.146968	4.584211	0.213225
63.C	5.235396	1.531235	0.703435
64.C	-0.114193	3.871834	0.946741
65.C	-1.229511	-5.219373	1.143853
66.C	0.528157	5.083942	1.207334
67.C	-4.222784	2.605354	1.260583
68.C	-0.398703	-0.817751	1.080894
69.C	-0.687800	-3.968415	1.363346
70.C	-2.230074	-5.674954	1.970031

71.C	-0.324690	2.899062	2.099374
72.C	-1.072291	-3.095668	2.375085
73.C	2.389752	-1.544498	2.378110
74.C	1.024085	2.295836	2.506940
75.C	3.661230	-1.800443	2.849502
76.C	-2.641206	-4.869236	3.011390
77.C	1.220999	-1.936209	3.012466
78.C	-2.059895	-3.635289	3.195823
79.C	-1.000623	3.553694	3.313676
80.C	-2.479376	-0.334422	3.407690
81.C	-1.141789	-0.656141	3.621752
82.C	3.823456	-2.495738	4.027661
83.C	1.456275	-2.660106	4.177236
84.C	-3.264839	0.415766	4.266621
85.C	2.703943	-2.925590	4.697528
86.C	-0.642003	-0.107000	4.799604
87.C	-2.721303	0.889177	5.435004
88.C	-1.400875	0.623209	5.704136
89.B	-0.324219	-1.620933	2.537864
90.H	-0.654233	-2.133137	-7.272862
91.H	-1.816069	0.021698	-7.021372
92.H	-2.383229	2.015815	-6.851981
93.H	6.691469	1.822262	-6.284228
94.H	2.823443	1.488973	-6.555567
95.H	1.200660	0.767142	-6.509136
96.H	7.984006	2.982337	-5.960665
97.H	6.285481	3.485797	-5.808506
98.H	-0.880949	2.409074	-5.988864
99.H	3.801164	-0.789419	-5.862017
100.H	-2.366093	3.351099	-5.702703
101.H	2.174133	-1.500504	-5.779025
102.H	1.557221	2.235497	-5.562017
103.H	-4.307370	0.826754	-5.591746
104.H	4.770972	1.262252	-5.429188
105.H	0.155010	-3.336949	-5.282128
106.H	7.599204	0.182547	-4.532112
107.H	8.972755	1.300004	-4.343608
108.H	-4.341279	2.336714	-4.654171
109.H	0.520900	4.038946	-4.780429
110.H	-1.759429	4.951168	-4.483673
111.H	3.259204	-1.575586	-4.367288
112.H	1.467209	0.190722	-4.120676
113.H	7.419855	3.091067	-3.590990
114.H	-4.188141	-1.355205	-4.016092
115.H	-4.267471	0.777278	-3.816160
116.H	-2.035382	1.843137	-3.804848
117.H	1.183968	5.291468	-3.720963
118.H	-0.400555	-4.711321	-3.617009
119.H	-2.945357	-2.515735	-3.520125
120.H	-1.262148	6.187214	-3.316225

121.H	8.040254	0.778099	-2.919877
122.H	1.782198	-3.357077	-3.534258
123.H	1.529996	3.582099	-3.383378
124.H	-4.640986	-2.820760	-3.128832
125.H	-2.534197	5.029879	-2.887134
126.H	-0.877548	3.162872	-2.938754
127.H	-1.850225	-4.138863	-2.774654
128.H	1.871551	-1.903506	-2.505877
129.H	-5.528181	-0.018796	-2.172835
130.H	-4.182967	3.529754	-2.103827
131.H	-0.455384	-4.730470	-1.847965
132.H	0.650144	6.407354	-1.895439
133.H	6.235305	2.474478	-1.598863
134.H	1.675762	-3.509112	-1.769326
135.H	-5.908876	-1.682037	-1.694224
136.H	-0.466982	-2.262096	-1.766864
137.H	-5.078373	2.191120	-1.351731
138.H	-5.425636	3.868691	-0.880048
139.H	-2.366363	-2.760112	-0.965527
140.H	-4.072535	-3.211861	-0.815455
141.H	-2.683627	5.050041	-0.663745
142.H	-5.347771	-0.544383	-0.477378
143.H	2.790624	3.752759	-0.397467
144.H	4.526256	4.089561	-0.193601
145.H	-3.887351	0.602188	-0.212574
146.H	1.258026	6.949300	0.431307
147.H	-3.404646	-1.941598	0.227693
148.H	-4.060401	5.153260	0.446268
149.H	3.168221	1.367604	0.181221
150.H	5.518561	0.493635	0.484770
151.H	6.112500	2.171716	0.524015
152.H	0.160791	-1.376213	0.305273
153.H	-2.465071	4.697762	1.061275
154.H	-1.467261	-0.725182	0.839011
155.H	3.541845	3.618131	1.211346
156.H	-4.740477	1.646728	1.136165
157.H	0.087019	0.167918	1.231206
158.H	-4.973173	3.334747	1.598947
159.H	5.001043	1.598427	1.775417
160.H	1.533801	1.827401	1.653706
161.H	-0.980359	2.083574	1.764600
162.H	-3.483554	2.491404	2.064034
163.H	0.833543	5.313675	2.228648
164.H	1.691360	3.078854	2.896670
165.H	-1.962138	4.018802	3.057751
166.H	0.899580	1.541957	3.295017
167.H	-0.361665	4.332726	3.754237
168.H	-1.181342	2.801607	4.093019

Energy: −1133.67225630 eV

Table S38. Coordinates and energy for 28

1.Ti	-0.753995	0.065049	0.632892
2.P	-2.245508	1.055657	-1.207900
3.P	1.070299	1.240654	0.234533
4.P	0.469087	-2.174920	1.101526
5.N	-2.158151	-1.430893	0.174727
6.C	-3.194836	0.730924	-3.844252
7.C	2.798324	0.112841	-3.738149
8.C	1.663423	2.340272	-3.501386
9.C	-1.483948	-0.925621	-3.015131
10.C	6.581257	4.175234	-2.468583
11.C	-1.967814	0.520822	-2.962906
12.C	7.482166	1.824942	-2.280330
13.C	2.184571	1.110573	-2.755491
14.C	4.430130	1.927899	-2.025277
15.C	-1.507040	3.616825	-1.969366
16.C	6.705851	2.937381	-1.575309
17.C	3.137739	1.567633	-1.668715
18.C	-2.675774	2.847083	-1.340413
19.C	5.322481	2.497524	-1.131622
20.C	-1.014498	-6.984565	-0.785306
21.C	2.079994	-2.412166	-1.132952
22.C	-2.561229	-4.992631	-0.846590
23.C	-5.050050	0.715501	-0.742983
24.C	-2.857758	-3.661239	-0.626397
25.C	-1.328161	-5.543853	-0.491906
26.C	-3.750630	0.215488	-0.649257
27.C	-7.519438	0.631590	-0.208402
28.C	-6.122160	0.080715	-0.122053
29.C	2.711389	1.761154	-0.323913
30.C	-3.020626	3.436256	0.024558
31.C	-1.935651	-2.798249	-0.010039
32.C	-0.405616	-4.697727	0.105970
33.C	4.882670	2.740605	0.173708
34.C	-3.496232	-0.968349	0.069701
35.C	-0.687581	-3.350793	0.354531
36.C	2.113011	-2.615493	0.379530
37.C	-5.846792	-1.067075	0.623556
38.C	3.595601	2.383049	0.590414
39.C	-4.566069	-1.585434	0.731071
40.C	3.238637	-1.807822	1.013719
41.C	3.177185	2.668929	2.029032
42.C	3.681970	3.998202	2.565192
43.C	-1.622436	0.772216	2.392305
44.C	3.588066	1.539127	2.957266
45.C	0.592773	-2.664228	2.895974
46.C	1.418081	-3.925566	3.166355
47.C	-0.728905	2.970437	3.250083
48.C	-0.809695	-2.829744	3.481305
49.C	-1.191265	1.564113	3.626109

50.C	-0.041524	0.839132	4.312303
51.C	-2.372371	1.691129	4.590489
52.H	-2.969485	0.422710	-4.877305
53.H	3.577217	0.582353	-4.358103
54.H	2.028478	-0.266561	-4.426871
55.H	0.919931	2.057149	-4.263645
56.H	2.488786	2.855737	-4.016889
57.H	-3.517799	1.781355	-3.883440
58.H	-1.251788	-1.200313	-4.056145
59.H	6.025443	3.936972	-3.388458
60.H	-4.042567	0.123630	-3.493954
61.H	6.981519	1.513819	-3.209267
62.H	7.572823	4.554912	-2.764270
63.H	3.249977	-0.745974	-3.224926
64.H	-1.147851	1.172050	-3.308062
65.H	8.493306	2.166530	-2.550909
66.H	4.745369	1.778124	-3.061085
67.H	-1.313959	3.320752	-3.007714
68.H	1.199654	3.059047	-2.813840
69.H	-2.249278	-1.623642	-2.648637
70.H	-0.575623	-1.075752	-2.416685
71.H	6.043471	4.984859	-1.955377
72.H	1.329750	0.633049	-2.249664
73.H	7.580592	0.936663	-1.640241
74.H	-0.940569	-7.169820	-1.868645
75.H	-1.732169	4.694425	-1.970547
76.H	-3.556545	2.925571	-1.999645
77.H	-3.308687	-5.622145	-1.337269
78.H	3.062666	-2.651855	-1.566574
79.H	1.327532	-3.046162	-1.620355
80.H	-7.951432	0.485567	-1.211165
81.H	-1.795114	-7.656592	-0.398621
82.H	-0.587815	3.461966	-1.384910
83.H	-5.234482	1.636980	-1.301345
84.H	1.857410	-1.361785	-1.365800
85.H	7.264736	3.224340	-0.669961
86.H	-3.822511	-3.275152	-0.952860
87.H	-7.542271	1.711437	-0.001508
88.H	-0.060844	-7.288742	-0.334100
89.H	-8.186256	0.139670	0.511170
90.H	-3.338874	4.483946	-0.092759
91.H	0.573762	-5.099273	0.376686
92.H	-3.832262	2.893412	0.527531
93.H	-2.140660	3.427113	0.681685
94.H	5.565510	3.218094	0.879387
95.H	2.266904	-3.686110	0.594548
96.H	4.206437	-2.138001	0.605937
97.H	-6.662975	-1.560616	1.157868
98.H	3.126174	-0.740122	0.781854
99.H	-4.382285	-2.474891	1.333226

100.H	3.428447	4.834166	1.896861
101.H	3.285181	-1.920439	2.105604
102.H	-2.383871	1.348924	1.808691
103.H	2.075748	2.702002	2.023059
104.H	4.774246	4.001324	2.707107
105.H	0.964840	-4.807401	2.689407
106.H	0.046527	2.931333	2.471587
107.H	3.146780	0.583984	2.643408
108.H	-2.164900	-0.143289	2.712520
109.H	2.460033	-3.843787	2.830993
110.H	-1.566411	3.573438	2.864157
111.H	4.683669	1.418461	2.969114
112.H	-1.310860	-3.706293	3.046098
113.H	3.232378	4.202798	3.548676
114.H	-1.445060	-1.953833	3.303248
115.H	1.081682	-1.802501	3.378920
116.H	0.820640	0.763421	3.634560
117.H	3.259164	1.740151	3.988892
118.H	1.437702	-4.120261	4.250659
119.H	-0.322815	3.498307	4.128336
120.H	-3.219707	2.208700	4.113081
121.H	-0.745542	-2.982154	4.569696
122.H	-0.334301	-0.179446	4.612866
123.H	-2.729405	0.701055	4.919404
124.H	0.278212	1.370782	5.224137
125.H	-2.094970	2.262983	5.493552
126.H	-1.931089	-6.538781	7.562129

Energy: –803.24960806 eV

Table S39. Coordinates and energy for 29

1.C	0.427157	-1.844048	-2.806044
2.C	-0.471111	-2.918702	-2.714217
3.C	3.099902	0.772158	-2.683444
4.C	-0.211869	-0.701770	-2.333613
5.C	-1.664047	-2.439943	-2.174340
6.C	-3.113054	2.041315	-2.014177
7.C	-1.495748	-1.063867	-1.930060
8.C	3.529828	0.662911	-1.236600
9.C	0.558692	4.106352	-1.121506
10.C	4.724493	0.004936	-0.955910
11.C	1.652565	3.262193	-0.993746
12.C	2.002339	-3.419606	-0.661283
13.C	-0.672799	3.674430	-0.670559
14.C	-3.297747	1.938903	-0.519160
15.C	1.003190	-4.362889	-0.375975
16.C	1.528738	1.999020	-0.426848
17.C	-4.578807	1.725109	-0.023008
18.C	2.798567	1.234604	-0.184386
19.C	-0.846607	2.399751	-0.121091
20.C	0.252934	1.511182	-0.009284

21.C	5.222915	-0.112024	0.329642
22.C	6.508796	-0.851483	0.612935
23.C	-2.226091	2.094402	0.379827
24.C	-3.040031	-4.206447	0.636351
25.C	2.138203	-2.556998	0.456402
26.C	-3.515110	-1.467131	0.728415
27.C	0.515789	-4.111951	0.888602
28.C	3.330882	1.190372	1.120359
29.C	-4.849399	1.710810	1.336907
30.C	4.514495	0.499660	1.358398
31.C	1.203612	-2.997967	1.397382
32.C	-6.260128	1.497244	1.827272
33.C	-2.486951	2.100831	1.761643
34.C	-3.793971	1.911752	2.213778
35.C	2.681731	1.929916	2.254923
36.C	-1.884523	-2.628681	2.710512
37.C	-1.387568	2.363383	2.756648
38.H	3.645491	1.584768	-3.189670
39.H	3.325222	-0.151845	-3.232868
40.H	1.438871	-1.891099	-3.196566
41.H	-0.274389	-3.942501	-3.017809
42.H	2.034682	0.994519	-2.788054
43.H	-3.852535	1.427761	-2.545875
44.H	-3.253977	3.080007	-2.352456
45.H	-2.111062	1.740260	-2.336871
46.H	0.201781	0.297466	-2.281008
47.H	-2.571638	-3.015278	-2.022303
48.H	5.287489	-0.434008	-1.785775
49.H	0.675150	5.103008	-1.549245
50.H	2.593775	-3.374804	-1.570392
51.H	-2.232590	-0.394179	-1.505429
52.H	2.643188	3.602081	-1.304333
53.H	0.683521	-5.165302	-1.034813
54.H	-5.399617	1.584632	-0.733027
55.H	-1.537468	4.338955	-0.729151
56.H	7.242916	-0.704092	-0.191327
57.H	-3.429464	-4.241809	-0.390667
58.H	-3.893897	-1.471405	-0.302304
59.H	6.336252	-1.935597	0.701768
60.H	-2.380483	-5.074856	0.773187
61.H	2.841752	-1.739975	0.567619
62.H	-3.178580	-0.452100	0.973503
63.H	-6.930283	2.294824	1.473175
64.H	-3.889687	-4.296255	1.331251
65.H	6.963180	-0.514223	1.553623
66.H	-6.672958	0.544904	1.462660
67.H	-4.336327	-1.755911	1.402145
68.H	-0.225959	-4.703778	1.414204
69.H	2.464791	2.972565	1.981883
70.H	4.913085	0.472526	2.376025

71.H	1.037118	-2.535430	2.364579
72.H	-6.306862	1.485658	2.923837
73.H	-0.830287	3.275986	2.501942
74.H	1.723752	1.457597	2.526408
75.H	-0.658165	1.535974	2.763309
76.H	-1.208709	-3.435329	3.020515
77.H	3.330535	1.931648	3.140628
78.H	-3.985028	1.929980	3.290245
79.H	-1.435187	-1.667606	2.997738
80.H	-2.845996	-2.754018	3.230916
81.H	-1.794952	2.475749	3.769882
82.P	-2.127901	-2.625068	0.897618
83.P	0.246882	-0.150161	0.785394
84.Ti	-0.014100	-2.189573	-0.495768

Energy: −571.32571057 eV

Table S40. Coordinates and energy for 30

1.C	3.003251	3.803194	-2.807456
2.C	-1.983245	0.557100	-2.204645
3.C	0.720603	3.392774	-1.957343
4.C	0.957916	-1.277260	-1.779964
5.C	2.209506	3.491212	-1.527321
6.C	-0.068437	-2.253128	-1.529827
7.C	-5.180740	-2.385236	-1.134089
8.C	-4.020642	-1.675483	-1.277583
9.C	5.391829	2.469702	-0.845827
10.C	2.066107	-1.628743	-0.977199
11.C	2.353015	4.601195	-0.516028
12.C	0.410982	-3.152733	-0.579089
13.C	-5.906559	-2.264124	-0.025950
14.C	-3.596304	-0.786502	-0.255787
15.C	1.738798	-2.789708	-0.240373
16.C	4.346761	1.880842	0.088261
17.C	4.726115	0.457358	0.386849
18.C	-2.497766	1.766035	0.287970
19.C	-5.536332	-1.380510	1.019353
20.C	-4.386547	-0.642032	0.865179
21.C	4.287778	2.638638	1.367124
22.C	-0.395542	-2.564076	2.467750
23.C	-1.397102	-1.577280	2.459009
24.C	0.816906	-1.944813	2.865708
25.C	-0.812645	-0.378232	2.835314
26.C	0.564953	-0.600589	3.085514
27.H	2.910265	2.989495	-3.541488
28.H	2.615158	4.727506	-3.270646
29.H	4.071684	3.959681	-2.615995
30.H	-1.736376	-0.320729	-2.811467
31.H	0.579548	2.589784	-2.697130
32.H	-2.950195	0.964688	-2.530520
33.H	0.405702	4.342752	-2.420264

34.H	-1.196385	1.307014	-2.358110
35.H	0.927090	-0.432854	-2.461326
36.H	-5.500435	-3.057618	-1.934386
37.H	-3.425815	-1.780719	-2.186332
38.H	5.385165	1.981817	-1.832429
39.H	-1.039335	-2.312713	-2.012583
40.H	5.256757	3.549691	-0.992591
41.H	1.890867	5.532239	-0.894244
42.H	0.074817	3.191146	-1.091040
43.H	3.009900	-1.096746	-0.968663
44.H	6.400555	2.332319	-0.414278
45.H	4.862243	-0.135880	-0.530573
46.H	3.405696	4.837711	-0.306845
47.H	-6.828033	-2.840987	0.091905
48.H	-3.412663	2.155406	-0.181207
49.H	-0.130214	-4.015273	-0.200568
50.H	1.862539	4.346389	0.435828
51.H	-1.655307	2.453576	0.135907
52.H	2.389384	-3.312773	0.455075
53.H	5.684797	0.430273	0.935438
54.H	3.971965	-0.043304	1.012403
55.H	4.127144	3.715066	1.228538
56.H	-2.654997	1.689043	1.370342
57.H	-6.160552	-1.283408	1.907417
58.H	-4.098785	0.054923	1.654615
59.H	5.239584	2.525374	1.921217
60.H	3.478859	2.270495	2.019551
61.H	-0.533947	-3.616258	2.238165
62.H	-2.443155	-1.750599	2.224159
63.H	1.774257	-2.444883	2.987995
64.H	-1.305529	0.582602	2.948368
65.H	1.276350	0.149640	3.410725
66.P	2.695654	1.741367	-0.936963
67.P	-2.026696	0.151814	-0.414035
68.P	1.260862	1.300767	0.670635
69.Zr	0.246446	-0.969965	0.608729

Energy: –447.05845290 eV

Table S41. Coordinates and energy for 31

1.C	-4.055856	1.584152	-3.916437
2.C	-2.672832	-1.388181	-3.576741
3.C	3.412584	-1.370363	-3.517808
4.C	-0.588638	0.783713	-3.558848
5.C	3.539654	1.042823	-2.977268
6.C	5.555030	-0.395719	-2.711124
7.C	4.038671	-0.351367	-2.576418
8.C	-3.433357	1.363828	-2.539208
9.C	-2.929184	2.687981	-1.987551
10.C	-4.557669	0.866277	-1.575848
11.C	1.463777	-2.390304	-0.994171

12.C	4.520905	-2.141270	-0.146459
13.C	-2.339977	-1.452698	-0.359351
14.C	-1.513582	-2.708406	-0.156277
15.C	0.852576	-3.096929	0.199222
16.C	4.027400	0.758220	0.304316
17.C	-2.465702	1.899477	2.096343
18.C	-0.533201	-2.417933	2.083070
19.C	0.225230	-1.294792	2.732815
20.C	0.123936	3.269427	3.618852
21.C	-2.404399	-0.263793	4.151537
22.C	-0.415099	2.050423	4.361402
23.C	0.774561	1.321783	4.992404
24.C	-1.355500	2.521130	5.474979
25.H	-3.312721	1.929371	-4.654442
26.H	-2.847148	-1.103167	-4.627004
27.H	3.701405	-1.159667	-4.566675
28.H	-4.524880	0.671118	-4.318268
29.H	-0.885076	1.029144	-4.593610
30.H	-4.843318	2.361610	-3.864508
31.H	3.822846	1.263595	-4.026020
32.H	5.861414	-0.134486	-3.744027
33.H	0.240580	0.060921	-3.615633
34.H	-1.931010	-2.204201	-3.586199
35.H	2.314422	-1.344695	-3.470226
36.H	3.734531	-2.402938	-3.298753
37.H	-3.618501	-1.810820	-3.200342
38.H	-0.202110	1.693426	-3.076670
39.H	2.447186	1.126082	-2.906484
40.H	5.967238	-1.394819	-2.493788
41.H	-2.200218	3.167228	-2.659334
42.H	3.963435	1.837289	-2.345665
43.H	6.052622	0.322395	-2.039072
44.H	-3.771999	3.395970	-1.854663
45.H	-4.994120	-0.093821	-1.898544
46.H	-5.377729	1.607941	-1.542767
47.H	0.741059	-2.401269	-1.831376
48.H	2.324097	-2.986167	-1.346089
49.H	1.250436	1.592325	-1.359800
50.H	-2.417760	2.569714	-1.022537
51.H	4.491945	-3.036398	-0.788451
52.H	-1.199619	-3.079161	-1.141450
53.H	-3.248144	-1.718817	-0.929361
54.H	-4.176522	0.744044	-0.552617
55.H	5.580575	-1.851278	-0.054574
56.H	0.601154	-4.159019	-0.020820
57.H	3.365169	1.628590	0.162125
58.H	5.067032	1.061132	0.093170
59.H	-2.082446	-3.519268	0.347473
60.H	4.186178	-2.447632	0.858766
61.H	-2.705164	-1.094776	0.621803

62.H	1.593663	-3.092185	1.011531
63.H	3.974887	0.486040	1.371604
64.H	-1.917593	2.480821	1.338030
65.H	-3.179259	1.256873	1.557753
66.H	-1.603612	-2.280240	2.281821
67.H	-0.243922	-3.418028	2.471583
68.H	-3.053713	2.606827	2.704245
69.H	1.310021	-1.435041	2.566060
70.H	0.756184	2.988418	2.761256
71.H	-0.685440	3.904983	3.226105
72.H	-2.964744	-0.943164	3.488834
73.H	0.094572	-1.358832	3.828284
74.H	0.732877	3.896892	4.299022
75.H	1.493066	0.996592	4.225718
76.H	-3.153682	0.316676	4.714752
77.H	-1.868140	-0.886910	4.885250
78.H	-2.248694	3.030368	5.076371
79.H	1.310986	1.990634	5.692971
80.H	0.467158	0.431459	5.567377
81.H	-0.838265	3.246551	6.133008
82.H	-1.701201	1.691112	6.111925
83.N	-1.519251	-0.426917	-1.039231
84.N	1.829178	-1.013547	-0.616303
85.N	-0.314542	-2.344923	0.616911
86.N	-0.239126	-0.017585	2.163940
87.P	0.632603	2.514482	-0.421682
88.Si	-2.034467	0.077325	-2.594941
89.Si	3.511439	-0.685776	-0.759389
90.Si	-1.308438	0.888726	3.145470
91.Zr	0.174305	0.138295	0.084189

Energy: −557.22955317 eV

Table S42. Coordinates and energy for 32

1.C	-0.099025	-0.634369	-3.086706
2.C	-4.418803	1.937827	-2.788895
3.C	-0.973575	0.541721	-2.707593
4.C	-3.529032	-1.522297	-2.515118
5.C	2.136204	-0.057069	-2.282109
6.C	2.377155	3.563945	-1.868135
7.C	1.174240	-2.279744	-1.780423
8.C	-4.417894	1.230209	-1.446947
9.C	-5.759548	0.586521	-1.207619
10.C	2.678810	0.445258	-0.970726
11.C	0.228210	-2.772961	-0.738984
12.C	-4.083288	2.212595	-0.349212
13.C	0.042221	3.634125	-0.009341
14.C	4.433111	3.334281	0.605014
15.C	-3.425777	-0.910391	0.443372
16.C	2.993263	3.532591	1.067702
17.C	2.790077	-3.476560	1.145427

18.C	2.812523	5.031414	1.353045
19.C	-0.097538	-5.017386	2.103188
20.C	2.810329	2.787199	2.379486
21.C	0.290383	-3.776105	2.863837
22.C	1.882376	-1.145222	2.821306
23.C	-0.963730	-3.083635	3.365982
24.C	1.129997	-4.204482	4.063968
25.H	0.376784	-0.502156	-4.077526
26.H	-4.624025	1.250992	-3.626299
27.H	-3.392184	-1.210412	-3.562806
28.H	-1.769102	0.667038	-3.461083
29.H	-0.735446	-1.527462	-3.147236
30.H	-3.460103	2.440541	-2.992795
31.H	-5.202229	2.717969	-2.811655
32.H	1.875294	0.809759	-2.903929
33.H	2.874362	-0.661532	-2.842630
34.H	-0.388473	1.476823	-2.736617
35.H	1.641324	3.424442	-2.677002
36.H	1.119582	-2.860226	-2.721420
37.H	-4.569123	-1.872012	-2.423170
38.H	-2.887027	-2.402153	-2.348163
39.H	3.320700	3.104476	-2.198760
40.H	-6.016326	-0.151895	-1.983024
41.H	2.564890	4.648514	-1.800528
42.H	-6.563978	1.347699	-1.224206
43.H	2.199589	-2.364405	-1.397711
44.H	3.570331	1.063396	-1.165592
45.H	-0.808416	-2.752082	-1.118601
46.H	-0.685563	3.117054	-0.654655
47.H	-4.785068	3.068549	-0.361687
48.H	-5.826148	0.086173	-0.231209
49.H	0.436163	-3.836059	-0.530237
50.H	-3.069823	2.623668	-0.462530
51.H	4.666214	3.899877	-0.309600
52.H	3.038060	-0.399844	-0.357527
53.H	0.036642	4.696288	-0.305306
54.H	2.944917	5.651774	0.451587
55.H	4.672320	2.275677	0.415322
56.H	-4.149398	1.767865	0.652719
57.H	-4.385944	-1.444996	0.504161
58.H	2.556662	-4.298258	0.451108
59.H	3.490515	-2.799055	0.630736
60.H	-2.648334	-1.658932	0.673745
61.H	-0.336526	3.573821	1.018128
62.H	5.130270	3.680252	1.389451
63.H	-3.408546	-0.178191	1.261838
64.H	-0.755180	-4.796689	1.247144
65.H	1.819049	5.257782	1.770419
66.H	0.774572	-5.572531	1.721424
67.H	3.563389	5.366361	2.090965

68.H	3.348780	-3.923530	1.983626
69.H	-1.933976	2.091228	1.761713
70.H	2.969150	1.705399	2.266367
71.H	2.424291	-0.398782	2.219984
72.H	-0.656294	-5.712778	2.757352
73.H	-1.616523	-2.765042	2.539891
74.H	1.802626	2.924031	2.800568
75.H	3.529694	3.152634	3.135341
76.H	-0.090035	1.958970	2.722193
77.H	1.067344	-0.618307	3.340663
78.H	2.580550	-1.498385	3.596241
79.H	2.049249	-4.735043	3.768797
80.H	-1.551366	-3.766261	4.007460
81.H	-0.741502	-2.187500	3.964553
82.H	0.550393	-4.897613	4.701330
83.H	1.422042	-3.353912	4.699146
84.N	0.892510	-0.838340	-2.038130
85.N	-1.518108	0.301619	-1.340273
86.N	1.610955	1.194925	-0.263493
87.N	0.365009	-1.924022	0.466648
88.P	-1.142488	1.063416	2.359273
89.Si	-3.167316	-0.176636	-1.269193
90.Si	1.746756	2.928029	-0.235264
91.Si	1.288408	-2.568671	1.780295
92.Zr	-0.072152	0.064908	0.102700

Energy: −560.16084697 eV

Table S43. Coordinates and energy for 33

1.C	2.452751	-1.613610	-5.981385
2.C	1.973708	-2.754963	-5.355379
3.C	2.857338	-0.518419	-5.227997
4.C	-2.376857	1.742325	-4.793418
5.C	5.565011	0.929819	-4.280494
6.C	1.013622	1.929383	-4.261716
7.C	1.928019	-2.816816	-3.970516
8.C	-4.580499	1.009322	-3.827785
9.C	-3.479833	2.071476	-3.787753
10.C	2.810390	-0.558852	-3.828185
11.C	2.364835	2.304219	-3.656831
12.C	2.382613	-1.745318	-3.205547
13.C	-2.026447	4.843787	-2.958816
14.C	4.964983	1.169955	-2.895610
15.C	2.170321	3.511272	-2.731722
16.C	5.386557	2.555791	-2.399838
17.C	-1.637122	3.646015	-2.090987
18.C	-5.012622	3.996203	-1.556738
19.C	2.494344	-1.825788	-1.731074
20.C	-4.257146	2.880610	-1.165337
21.C	3.798455	-1.620701	-1.161480
22.C	-6.027508	4.478888	-0.736062

23.C	1.401150	-2.017574	-0.844182
24.C	-1.360633	4.113981	-0.664395
25.C	-4.283557	-0.231811	-0.277643
26.C	-4.575928	2.239716	0.036210
27.C	-3.584049	-1.430269	-0.043007
28.C	-6.316959	3.849322	0.464865
29.C	-1.382892	-3.615506	0.121694
30.C	3.967302	-1.452430	0.217300
31.C	-0.708273	-4.794339	0.403528
32.C	-3.848073	0.985372	0.363758
33.C	-5.605545	2.719252	0.847156
34.C	1.569137	-1.864642	0.566728
35.C	5.754559	1.503859	0.891494
36.C	-2.470964	-1.487032	0.822313
37.C	2.824161	-1.471043	1.097033
38.C	4.608269	2.428752	1.295978
39.C	-1.841172	-2.788139	1.157915
40.C	1.741597	3.948176	1.314489
41.C	-2.783416	0.944935	1.287898
42.C	-2.039969	-0.253821	1.422323
43.C	-0.491814	-5.170444	1.727456
44.C	1.884430	2.716723	2.209683
45.C	5.011284	3.287766	2.495398
46.C	-1.710907	-3.206987	2.501186
47.C	-4.954107	-3.401232	2.779542
48.C	3.050304	-1.081904	2.513920
49.C	0.522070	2.105896	2.528650
50.C	-1.004368	-4.392784	2.757410
51.C	3.251984	0.278727	2.806936
52.C	3.124007	-2.041255	3.528810
53.C	-4.097023	-3.398746	4.052082
54.C	3.533375	0.652607	4.127963
55.C	-3.833719	-4.825180	4.524555
56.C	3.399140	-1.652376	4.836606
57.C	3.611768	-0.311755	5.130493
58.C	-0.274800	-1.839659	5.157797
59.C	-1.618328	-2.540584	5.330219
60.C	-2.393784	-1.965837	6.518568
61.H	2.485387	-1.561588	-7.071212
62.H	1.624597	-3.600935	-5.949833
63.H	-2.813812	1.620173	-5.797992
64.H	3.182924	0.386119	-5.742272
65.H	5.154111	1.625919	-5.028705
66.H	1.084768	1.203311	-5.078765
67.H	-5.007813	0.953616	-4.842301
68.H	-1.621180	2.535011	-4.859463
69.H	5.403428	-0.093106	-4.642003
70.H	0.533675	2.835843	-4.660905
71.H	6.652646	1.106963	-4.246494
72.H	-1.878356	0.799844	-4.525471

73.H	3.069564	2.573607	-4.464205
74.H	-3.920288	3.054023	-4.031106
75.H	-2.274207	4.570903	-3.994080
76.H	-4.169010	0.020784	-3.576698
77.H	1.573903	-3.716972	-3.467736
78.H	0.345942	1.497239	-3.500569
79.H	-5.403393	1.238588	-3.135073
80.H	1.715362	4.341925	-3.294562
81.H	5.105641	3.335829	-3.122670
82.H	-1.181564	5.550287	-3.003023
83.H	-2.875803	5.395310	-2.531339
84.H	-4.815314	4.492260	-2.506825
85.H	6.481251	2.597344	-2.288361
86.H	3.105018	3.880407	-2.292915
87.H	5.395392	0.423905	-2.208157
88.H	-0.715717	3.200372	-2.496475
89.H	1.499734	3.241401	-1.903650
90.H	4.657970	-1.564393	-1.828242
91.H	4.939257	2.809865	-1.432338
92.H	-6.602024	5.353640	-1.046211
93.H	0.405598	-2.176891	-1.255597
94.H	-5.124064	-0.201216	-0.966851
95.H	-1.529612	-3.285717	-0.909852
96.H	-0.559657	4.869679	-0.663420
97.H	-3.883857	-2.333447	-0.573282
98.H	-0.333348	-5.415035	-0.412010
99.H	-2.254930	4.577753	-0.220626
100.H	5.502362	0.918161	-0.000132
101.H	-1.048538	3.281320	-0.022781
102.H	4.397585	3.105744	0.450349
103.H	6.663406	2.086488	0.674498
104.H	1.361384	3.661916	0.324868
105.H	4.953174	-1.251512	0.632362
106.H	-7.114694	4.231106	1.104742
107.H	2.690414	4.484629	1.174227
108.H	0.703155	-1.952413	1.220652
109.H	5.997497	0.803203	1.705000
110.H	-5.846959	2.196790	1.774259
111.H	1.024903	4.658045	1.756053
112.H	0.059316	-6.084404	1.956828
113.H	0.016931	1.782952	1.606969
114.H	-4.442063	-3.931969	1.963101
115.H	-2.475363	1.856343	1.798925
116.H	5.883100	3.907786	2.229561
117.H	-1.156002	-0.262536	2.052326
118.H	-5.179930	-2.382013	2.435077
119.H	4.218923	3.972595	2.825002
120.H	-5.909001	-3.919257	2.964163
121.H	5.312894	2.667336	3.351268
122.H	-0.114833	2.850750	3.031546

123.H	2.365467	3.010116	3.157073
124.H	0.610716	1.236885	3.195321
125.H	2.964630	-3.091484	3.281857
126.H	-3.281588	-5.400278	3.766849
127.H	-0.863317	-4.724148	3.787479
128.H	-4.788311	-5.348394	4.702003
129.H	3.664333	1.701701	4.392627
130.H	0.303857	-2.255281	4.323174
131.H	-4.669710	-2.869546	4.835300
132.H	-3.264089	-4.863865	5.465054
133.H	-0.414851	-0.764361	4.966828
134.H	-1.440939	-3.614086	5.513081
135.H	3.449262	-2.401512	5.628468
136.H	3.823553	-0.006625	6.157000
137.H	0.336758	-1.935258	6.069352
138.H	-2.656053	-0.910994	6.343161
139.H	-3.325494	-2.514606	6.714238
140.H	-1.786456	-2.012075	7.436609
141.Cl	-1.889551	-1.308824	-2.792573
142.Mo	-2.078782	0.204425	-0.791590
143.Mo	2.318299	0.026659	-0.502190
144.P	3.116365	0.883526	-2.706964
145.P	-2.806015	2.195930	-2.058297
146.P	0.124768	0.609921	-0.748756
147.P	3.008953	1.468681	1.409886
148.P	-2.628776	-2.270684	3.796316

Energy: –985.97578230 eV

Table S44. Coordinates and energy for 34

1.C	3.309018	5.158443	-4.400942
2.C	4.500249	4.467501	-4.254029
3.C	-0.876448	3.536787	-4.400671
4.C	2.144271	4.690577	-3.804877
5.C	-0.321624	1.123834	-4.002408
6.C	4.532543	3.305222	-3.492810
7.C	-0.591841	2.467576	-3.339833
8.C	2.152455	3.515209	-3.040959
9.C	3.365877	2.829696	-2.887415
10.C	-4.405724	-4.595603	-2.420076
11.C	-4.465462	-3.223313	-2.249764
12.C	2.942506	0.384495	-2.401375
13.C	6.296787	-5.152316	-1.810110
14.C	3.402095	1.657075	-1.975476
15.C	-3.704261	-5.381687	-1.511131
16.C	3.811142	-5.273964	-1.485655
17.C	-3.366559	-0.092588	-1.558604
18.C	2.846318	-0.673592	-1.443719
19.C	0.086080	4.456092	-1.337389
20.C	-3.790087	-2.613437	-1.192245
21.C	5.121487	-4.816476	-0.886236

22.C	-0.211754	-2.886579	-1.264636
23.C	-3.462872	1.229730	-1.021073
24.C	-3.955869	-1.176205	-0.860105
25.C	-1.317886	4.291073	-0.755268
26.C	3.698227	1.886290	-0.615124
27.C	-3.013776	-4.784952	-0.459601
28.C	1.078182	4.973927	-0.295070
29.C	-3.027854	-3.395423	-0.310407
30.C	6.663424	-2.611230	0.261920
31.C	3.235489	-0.479189	-0.095424
32.C	-4.175124	1.480641	0.182475
33.C	-4.746778	3.909619	0.413112
34.C	-4.775170	-0.912509	0.299160
35.C	-0.274788	-2.958043	0.263111
36.C	3.559799	0.824855	0.334941
37.C	6.635646	-1.166765	0.748154
38.C	3.892140	-2.778481	0.772674
39.C	0.271920	-4.299947	0.734735
40.C	-4.882152	0.393529	0.794900
41.C	-4.676151	5.107919	1.110507
42.C	-4.129785	2.768392	0.916590
43.C	7.064183	-3.570546	1.383513
44.C	3.193227	-1.565109	0.927023
45.C	3.786023	-3.728908	1.797330
46.C	-3.990040	5.172343	2.314480
47.C	-3.452645	2.819026	2.154364
48.C	2.489135	-1.323577	2.108712
49.C	-2.187239	-3.456650	2.423761
50.C	-0.297634	3.006313	2.602913
51.C	-3.653296	-3.567144	2.814240
52.C	-3.399067	4.029477	2.843118
53.C	3.068412	-3.484996	2.962419
54.C	2.437388	-2.264486	3.126790
55.C	-0.997988	1.976968	3.469748
56.C	-1.364756	-2.835260	3.547275
57.C	-0.083930	0.804933	3.805077
58.C	-4.751433	0.077540	4.061691
59.C	-3.410157	0.750082	4.276574
60.C	-3.579807	1.834497	5.354259
61.H	3.280242	6.079148	-4.984730
62.H	5.413927	4.837117	-4.720325
63.H	-0.030110	3.649755	-5.091761
64.H	-1.748799	3.229678	-4.997890
65.H	-1.167350	0.839941	-4.648127
66.H	0.571958	1.168623	-4.644273
67.H	1.228484	5.267520	-3.923177
68.H	-1.110145	4.520303	-3.969486
69.H	5.468787	2.763544	-3.352078
70.H	-4.934316	-5.062453	-3.252050
71.H	2.689610	0.225380	-3.446440

72.H	-0.169490	0.325928	-3.261770
73.H	6.220722	-4.604820	-2.762201
74.H	-5.057856	-2.612063	-2.932991
75.H	-1.486914	2.356830	-2.706827
76.H	6.301000	-6.227664	-2.043677
77.H	3.619456	-4.773453	-2.447561
78.H	-2.742968	-0.283179	-2.429644
79.H	0.043673	5.180460	-2.166616
80.H	3.824373	-6.358524	-1.676622
81.H	-3.684024	-6.465752	-1.628171
82.H	7.267832	-4.909712	-1.359059
83.H	2.503800	-1.654997	-1.756844
84.H	-0.803436	-3.686500	-1.731801
85.H	-2.075763	4.164928	-1.540888
86.H	0.830256	-3.018590	-1.591874
87.H	-0.563626	-1.920708	-1.652543
88.H	-2.952025	2.053919	-1.508727
89.H	2.957171	-5.068321	-0.825798
90.H	7.414156	-2.681147	-0.545141
91.H	2.078995	5.131299	-0.722797
92.H	-5.286479	3.853868	-0.532739
93.H	3.982888	2.883115	-0.292097
94.H	5.280805	-5.338368	0.071380
95.H	-1.596675	5.190316	-0.186272
96.H	6.370364	-0.468169	-0.058773
97.H	-1.376695	3.433552	-0.071966
98.H	0.730542	5.941672	0.099188
99.H	-2.456199	-5.412690	0.234914
100.H	-0.309458	-5.146644	0.339690
101.H	-5.151877	6.001301	0.703896
102.H	1.297044	-4.415239	0.354574
103.H	1.171247	4.273959	0.545079
104.H	7.126812	-4.614988	1.048489
105.H	-5.298601	-1.736215	0.776780
106.H	7.626753	-0.875054	1.130042
107.H	1.582525	2.169304	0.579611
108.H	0.375272	-2.161981	0.666296
109.H	8.057811	-3.295346	1.772716
110.H	3.819813	0.997063	1.376121
111.H	5.918235	-1.036988	1.571921
112.H	-5.476029	0.591063	1.680527
113.H	4.296384	-4.685880	1.693053
114.H	0.326731	-4.392101	1.826653
115.H	6.361062	-3.517872	2.226983
116.H	0.018966	2.574425	1.646744
117.H	-4.255573	-4.072246	2.047013
118.H	-1.799791	-4.462221	2.201763
119.H	1.965822	-0.374901	2.212932
120.H	-3.922787	6.116706	2.855571
121.H	-0.918120	3.887977	2.405492

122.H	-4.090382	-2.574921	2.995178
123.H	0.616046	3.351803	3.115331
124.H	0.159707	0.234841	2.898869
125.H	-4.707480	-0.708558	3.298889
126.H	-0.302183	-2.741657	3.285972
127.H	-3.754110	-4.142496	3.748285
128.H	3.021953	-4.244889	3.743684
129.H	-2.861994	4.104064	3.787765
130.H	-5.523380	0.807510	3.772854
131.H	-1.741060	-1.829517	3.783103
132.H	1.894231	-2.039212	4.044743
133.H	0.856934	1.172393	4.244966
134.H	-1.434217	-3.446161	4.461010
135.H	-1.330283	2.456669	4.399460
136.H	-0.530944	0.113475	4.532333
137.H	-2.710661	-0.012458	4.663078
138.H	-5.093389	-0.388556	4.998128
139.H	-4.259588	2.628445	5.015029
140.H	-2.640695	2.294313	5.683669
141.H	-4.034532	1.377360	6.246574
142.P	0.719571	2.874048	-2.081714
143.P	5.063661	-2.978596	-0.634069
144.P	-0.448523	0.454756	0.020761
145.P	-1.976262	-2.457051	0.871388
146.P	-2.556035	1.284540	2.677708
147.Mo	1.560752	1.056954	-0.699875
148.Mo	-2.632562	-0.099246	0.593911

Energy: –980.49755235 eV

Table S45. Coordinates and energy for 35

1.C	1.620435	1.593040	-6.362423
2.C	1.598759	0.292456	-6.014674
3.C	1.820732	2.560050	-5.399085
4.C	1.753088	-0.125018	-4.704815
5.C	-1.123594	4.668144	-4.440464
6.C	-1.793442	2.206050	-4.381837
7.C	1.816448	-1.590044	-4.335292
8.C	-0.935095	5.880668	-3.845529
9.C	1.961593	2.229027	-4.045577
10.C	-1.551521	3.541925	-3.732493
11.C	1.904596	0.869614	-3.719618
12.C	2.218461	3.281397	-3.020449
13.C	-1.186222	6.022524	-2.546375
14.C	-1.733419	3.733980	-2.348159
15.C	-1.586719	4.989935	-1.756106
16.C	-4.031728	-0.413075	-1.621323
17.C	1.813704	-3.642410	-1.456941
18.C	-3.188176	-2.762492	-1.429154
19.C	4.924226	-1.205588	-1.145128
20.C	-3.232914	-1.377091	-0.747115

21.C	1.290025	-4.581472	-0.412575
22.C	-1.796614	-0.823161	-0.556861
23.C	-1.893528	5.166235	-0.280904
24.C	1.905032	-5.793552	-0.135356
25.C	4.976183	-0.788387	0.303456
26.C	0.140011	-4.231463	0.349382
27.C	-4.936401	2.639800	0.599706
28.C	1.366027	-6.641455	0.822698
29.C	-3.931659	-1.528664	0.604686
30.C	6.174361	-0.798264	1.019696
31.C	3.816721	-0.392280	0.992092
32.C	-0.439504	-5.121126	1.228836
33.C	0.193195	-6.317467	1.473034
34.C	-4.066082	2.973137	1.773095
35.C	-1.770277	-4.831235	1.896250
36.C	-2.829140	2.334521	1.965665
37.C	6.207028	-0.444295	2.347621
38.C	3.826410	0.033100	2.312056
39.C	2.007639	-2.921195	2.433132
40.C	-4.477844	3.928671	2.679304
41.C	5.054188	-0.039608	2.984146
42.C	2.601210	0.515380	3.005399
43.C	-2.004461	2.641319	3.061903
44.C	-0.332970	-2.021081	3.067521
45.C	0.794487	-2.838283	3.309101
46.C	-0.655289	2.003170	3.284864
47.C	-3.692565	4.241829	3.785852
48.C	-2.533436	-0.944568	3.762339
49.C	-2.467372	3.626033	3.938701
50.C	-1.373861	-1.872972	3.976972
51.C	0.799421	-3.566185	4.506902
52.C	-1.283787	-2.607656	5.176684
53.C	-0.216861	-3.450028	5.421854
54.H	1.505275	1.887682	-7.407024
55.H	1.472534	-0.475295	-6.783560
56.H	1.873017	3.614118	-5.679365
57.H	-0.958481	4.556265	-5.515714
58.H	-1.899927	2.320620	-5.468120
59.H	1.859486	-2.212727	-5.237417
60.H	-0.606385	6.731267	-4.445541
61.H	-0.963962	1.509381	-4.195982
62.H	-2.703263	1.734901	-3.987434
63.H	2.699095	-1.804328	-3.718998
64.H	0.931253	-1.888445	-3.756444
65.H	2.541786	4.215071	-3.497037
66.H	1.313442	3.507307	-2.434431
67.H	-3.572949	-0.295285	-2.615179
68.H	-1.068775	7.003623	-2.074579
69.H	2.990508	2.969746	-2.304822
70.H	-2.672487	-2.710087	-2.400890

71.H	2.601718	-4.119843	-2.053161
72.H	1.010800	-3.323351	-2.134065
73.H	-5.052978	-0.798917	-1.770491
74.H	4.417884	-0.455599	-1.766730
75.H	5.937853	-1.353275	-1.537587
76.H	-4.217242	-3.114009	-1.611229
77.H	4.374935	-2.150233	-1.269540
78.H	-4.122801	0.582003	-1.163360
79.H	2.235825	-2.730986	-1.009764
80.H	-2.674080	-3.505547	-0.810317
81.H	2.804229	-6.078521	-0.684899
82.H	-4.427774	2.827718	-0.357206
83.H	-1.780681	6.218298	0.009310
84.H	-2.917417	4.850211	-0.041311
85.H	-1.218438	4.567619	0.348347
86.H	-5.854913	3.239296	0.617171
87.H	7.089187	-1.100164	0.504916
88.H	-4.945379	-1.935222	0.456345
89.H	-5.225216	1.579032	0.601065
90.H	1.856786	-7.593218	1.034076
91.H	-4.015717	-0.566888	1.124132
92.H	-2.441090	-4.276894	1.229705
93.H	-3.388845	-2.225238	1.258687
94.H	1.905283	-2.362914	1.499730
95.H	-0.252165	-7.019525	2.180272
96.H	-2.261786	-5.774634	2.168187
97.H	2.241770	-3.965553	2.187386
98.H	-5.441665	4.420505	2.532044
99.H	7.148419	-0.474954	2.898024
100.H	2.234535	1.447969	2.551900
101.H	-1.647149	-4.240343	2.814665
102.H	2.879330	-2.506541	2.960421
103.H	-0.605654	0.988487	2.881036
104.H	0.136002	2.589457	2.790211
105.H	-2.478616	-0.421699	2.806157
106.H	1.774604	-0.203157	2.944586
107.H	-3.484727	-1.495746	3.795976
108.H	5.086077	0.260128	4.034106
109.H	2.805974	0.714249	4.064259
110.H	-4.026898	4.989005	4.506401
111.H	-0.419681	1.971720	4.356668
112.H	-2.573667	-0.192126	4.563276
113.H	-1.841524	3.882881	4.797788
114.H	1.653226	-4.215618	4.712489
115.H	-2.084473	-2.502993	5.912040
116.H	-0.179688	-4.018483	6.352857
117.O	2.065056	0.480580	-2.409789
118.O	-2.103567	2.658821	-1.555469
119.O	-0.499646	-3.025552	0.061763
120.O	2.630851	-0.433222	0.292504

121.O	-2.494211	1.309830	1.098436
122.O	-0.397433	-1.239317	1.898258
123.P	-0.834759	-0.380357	-2.086262
124.P	0.679140	2.271949	-0.009629
125.P	-0.395482	-1.429401	0.285611
126.W	1.193172	0.157135	-0.757218
127.W	-1.350755	1.329180	-0.465237

Energy: −884.65735946 eV

Table S46. Coordinates and energy for 36

1.W	-0.746750	0.173899	0.015656
2.Ga	3.711001	-0.462332	0.072260
3.Cl	4.619515	0.617836	-1.560568
4.Cl	4.130903	-2.475709	-0.668550
5.Cl	4.146894	-2.566600	-0.133780
6.Cl	4.523114	0.253892	1.940574
7.P	1.399290	-0.132397	0.042911
8.Si	-0.062332	-0.176286	-3.241494
9.Si	0.332083	3.003863	1.441760
10.Si	-0.472753	-2.596723	1.874634
11.N	-1.133498	0.299800	-1.908038
12.N	-2.971341	0.491410	-0.012591
13.N	-1.418247	-1.444567	0.909900
14.N	-0.950077	1.846440	1.031159
15.C	-1.099582	-0.416729	-4.766970
16.C	1.191198	1.172211	-3.575565
17.C	0.758387	-1.821358	-2.944060
18.C	-2.450925	0.800534	-2.366315
19.C	-3.243578	1.346850	-1.200367
20.C	-3.573105	-0.866334	-0.125376
21.C	1.460205	3.358128	0.002960
22.C	0.427632	-3.792703	0.751989
23.C	-2.860136	-1.780884	0.844929
24.C	-3.300901	1.165403	1.274743
25.C	-2.290720	2.267188	1.502340
26.C	-0.462618	4.635003	1.852378
27.C	-1.645990	-3.558501	2.951329
28.C	1.284856	2.407944	2.937912
29.C	0.692335	-1.750358	3.055536
30.H	-0.436695	-0.733692	-5.588988
31.H	-1.621306	0.488067	-5.114183
32.H	-1.853077	-1.212360	-4.647699
33.H	1.812094	0.905577	-4.447086
34.H	1.088034	-2.244533	-3.908534
35.H	0.701489	2.133889	-3.796414
36.H	-2.334048	1.604418	-3.111197
37.H	-3.016933	0.000540	-2.873844
38.H	1.869684	1.316181	-2.722001
39.H	1.642984	-1.741089	-2.296142
40.H	0.065743	-2.543858	-2.483544

41.H	-4.324910	1.390553	-1.420808
42.H	-3.410926	-1.205855	-1.155836
43.H	-2.900213	2.358378	-0.952220
44.H	0.890888	3.539830	-0.922551
45.H	2.042569	4.270006	0.220026
46.H	-4.661100	-0.832778	0.061074
47.H	2.178603	2.552810	-0.203119
48.H	-0.269076	-4.323117	0.082699
49.H	1.170895	-3.277110	0.125032
50.H	-3.013314	-2.820717	0.513056
51.H	-1.020122	5.061519	1.003173
52.H	-2.609924	3.192338	0.992699
53.H	-4.336447	1.549106	1.266616
54.H	0.957816	-4.555248	1.346836
55.H	-3.324746	-1.714138	1.844113
56.H	0.342570	5.349639	2.090368
57.H	-3.218886	0.408543	2.064080
58.H	-2.397574	-4.151355	2.407435
59.H	-2.276993	2.505950	2.578157
60.H	-1.134676	4.616042	2.723547
61.H	1.603260	-1.364540	2.577828
62.H	1.798394	1.455044	2.740639
63.H	2.058792	3.141449	3.217623
64.H	-1.060894	-4.269117	3.557999
65.H	0.199186	-0.906763	3.563762
66.H	-2.183260	-2.908977	3.661129
67.H	0.625892	2.267515	3.809955
68.H	1.000331	-2.469826	3.834294

Energy: –213.72220598 eV

Table S47. Coordinates and energy for 37

1.C	-1.023372	1.092242	-3.521964
2.C	-0.654750	-2.688396	-2.585998
3.C	-2.531204	3.427390	-2.408664
4.C	0.458927	3.233917	-1.916964
5.C	-1.333962	-4.691497	-0.407400
6.C	1.419753	-3.518870	-0.496323
7.C	-2.791840	1.610943	0.051421
8.C	3.075969	2.139800	0.777302
9.C	-1.926120	-2.223566	1.318306
10.C	3.360394	-0.806928	1.549258
11.C	-2.687500	1.422094	1.586405
12.C	-2.835304	-1.010952	1.643388
13.C	-0.031090	1.004939	3.062885
14.C	-1.323313	0.154405	3.160172
15.C	2.960762	1.338822	3.660194
16.H	-0.977823	1.689952	-4.448302
17.H	-1.912267	0.445378	-3.592918
18.H	-2.321163	3.939418	-3.361810
19.H	-0.136298	0.445405	-3.510988

20.H	-0.533385	-3.594489	-3.203977
21.H	0.058999	-1.941280	-2.957988
22.H	-1.671207	-2.302532	-2.760625
23.H	0.514826	3.952837	-2.752191
24.H	-3.502129	2.920937	-2.526346
25.H	1.360065	2.608268	-1.963478
26.H	-2.648849	4.214895	-1.647184
27.H	-0.945270	-5.496444	-1.052125
28.H	1.687528	-4.425796	-1.064933
29.H	0.495139	3.811499	-0.980124
30.H	2.100559	-2.721058	-0.821159
31.H	-2.413128	-4.614613	-0.615609
32.H	-3.552755	0.925590	-0.363515
33.H	-3.156472	2.628628	-0.142921
34.H	2.983605	1.876224	-0.284489
35.H	-1.211922	-5.030078	0.633398
36.H	1.624835	-3.726096	0.566085
37.H	3.344471	-1.058659	0.480578
38.H	4.144360	2.335337	0.971759
39.H	2.535505	3.086292	0.938617
40.H	-3.497982	-0.824987	0.789145
41.H	-2.567525	-3.052760	0.990714
42.H	4.418502	-0.723954	1.851051
43.H	2.923482	-1.651993	2.103824
44.H	-3.675426	1.389100	2.076955
45.H	-2.106433	2.251921	2.007257
46.H	-1.416291	-2.570789	2.234922
47.H	-3.448844	-1.171975	2.546493
48.H	-0.286629	2.079433	3.033658
49.H	-1.050991	-0.877324	3.415120
50.H	4.051607	1.494473	3.683087
51.H	2.496756	2.291577	3.959917
52.H	-2.026987	0.538996	3.918299
53.H	0.548163	0.851843	3.983624
54.H	2.732922	0.590943	4.436453
55.Au	2.914755	-0.240779	-2.720543
56.Cl	4.589113	-0.379088	-4.283344
57.N	-1.471717	1.382173	-0.557234
58.N	-0.959763	-1.840986	0.276532
59.N	-1.942959	0.160507	1.813501
60.N	0.728947	0.599462	1.869802
61.P	1.294297	-0.106920	-1.208061
62.Si	-1.118261	2.238163	-2.056843
63.Si	-0.375880	-3.119191	-0.787322
64.Si	2.479205	0.799653	1.924743
65.W	-0.280081	0.023138	0.261420

Energy: –396.48816114 eV

Table S48. Coordinates and energy for 38

1.C	3.201706	-1.917912	-4.997460

2.C	-4.730539	-0.594833	-4.334605
3.C	1.758078	-0.080666	-3.246859
4.C	5.529053	-1.741071	-2.917298
5.C	-2.293072	0.561376	-3.028658
6.C	1.749748	-2.977672	-2.635770
7.C	6.599972	-2.029448	-1.947166
8.C	-6.169005	0.728168	-1.978820
9.C	-3.202375	-2.080140	-2.021277
10.C	6.970342	0.257646	-1.187385
11.C	-6.739688	1.838179	-1.122328
12.C	2.653560	2.714194	-0.581210
13.C	6.389439	1.274868	-0.228632
14.C	-1.393210	3.396005	-0.406830
15.C	7.155074	-1.439909	0.410840
16.C	-7.264328	0.447158	0.683211
17.C	5.092487	3.883672	0.710137
18.C	-5.323199	3.611547	0.855926
19.C	6.243062	-2.642837	1.022651
20.C	-6.516663	2.786449	1.112910
21.C	-2.812038	5.458610	1.095430
22.C	2.421868	-3.942919	1.336129
23.C	-3.646554	-2.886436	1.661990
24.C	-6.547798	-0.126175	2.028938
25.C	3.182108	1.895985	2.224215
26.C	-1.737916	3.085136	2.517326
27.C	4.769043	-4.249640	3.140184
28.C	3.140495	-1.711858	3.405568
29.C	-5.479333	-2.204456	3.972562
30.C	-2.930837	-0.681895	3.760649
31.H	2.263002	-1.960532	-5.573734
32.H	3.813201	-1.119332	-5.448180
33.H	3.721539	-2.873620	-5.174647
34.H	-4.103467	-1.157327	-5.045176
35.H	-4.874140	0.410726	-4.758317
36.H	-5.709516	-1.096943	-4.293575
37.H	5.407650	-2.612125	-3.579224
38.H	5.826097	-0.909785	-3.582932
39.H	0.964257	-0.191246	-4.006472
40.H	-1.726650	0.142925	-3.878856
41.H	2.346071	0.808414	-3.528718
42.H	-2.582949	1.588359	-3.303809
43.H	-6.156083	1.080562	-3.020142
44.H	0.877638	-3.114671	-3.298135
45.H	7.601312	-1.981834	-2.417088
46.H	2.299797	-3.933670	-2.625722
47.H	-2.680493	-2.630865	-2.823689
48.H	6.633118	0.503831	-2.204413
49.H	1.260409	0.141799	-2.294244
50.H	-6.818401	-0.164998	-1.969844
51.H	-1.605921	0.622209	-2.174839

52.H	6.477982	-3.040849	-1.533672
53.H	-3.994926	-2.741060	-1.636411
54.H	-6.270935	2.786496	-1.421802
55.H	-7.832666	1.944762	-1.260477
56.H	1.364204	-2.808489	-1.620211
57.H	8.077017	0.275312	-1.186329
58.H	3.072777	2.858660	-1.589915
59.H	-1.855490	3.647031	-1.375864
60.H	-2.476519	-1.918532	-1.213457
61.H	6.525199	2.270892	-0.673490
62.H	1.888892	1.929053	-0.642544
63.H	5.381384	4.186201	-0.307968
64.H	-0.985429	2.380439	-0.489440
65.H	-7.209450	-0.325549	-0.085103
66.H	8.201991	-1.751503	0.247212
67.H	2.142266	3.652140	-0.302675
68.H	-5.462106	4.215625	-0.059814
69.H	-0.534416	4.078186	-0.278061
70.H	-3.283329	5.884040	0.194431
71.H	6.438535	-3.562866	0.441313
72.H	6.942341	1.292634	0.727161
73.H	-8.312962	0.744323	0.862187
74.H	-7.451154	3.345801	0.913846
75.H	2.841410	-4.632767	0.586146
76.H	7.108729	-0.595436	1.100068
77.H	4.493246	4.706346	1.132868
78.H	1.679567	-3.307155	0.835383
79.H	-1.825321	5.940287	1.193022
80.H	6.006001	3.798227	1.318798
81.H	-2.802757	-2.593097	1.023879
82.H	-4.453280	-3.266115	1.015445
83.H	-5.216867	4.343253	1.671217
84.H	6.604666	-2.838828	2.042753
85.H	-3.406165	5.783143	1.965202
86.H	-6.541164	2.468231	2.165125
87.H	-7.035846	-1.080052	2.276978
88.H	1.890512	-4.553816	2.086036
89.H	-3.313384	-3.719381	2.304429
90.H	5.246701	-5.007618	2.499112
91.H	2.392287	1.151477	2.058305
92.H	-6.768377	0.562683	2.865206
93.H	2.698372	2.798082	2.638767
94.H	-1.452636	2.023867	2.485085
95.H	-0.815279	3.666911	2.686580
96.H	3.857937	1.504280	3.000733
97.H	2.410202	-1.022003	2.962870
98.H	-6.277705	-2.806972	3.510855
99.H	-2.376862	3.229003	3.404911
100.H	5.549712	-3.810195	3.781306
101.H	-2.079689	-0.323588	3.166371

102.H	4.077271	-4.787680	3.809242
103.H	3.971877	-1.117828	3.816468
104.H	2.651377	-2.230082	4.248128
105.H	-5.957305	-1.445299	4.612016
106.H	-4.921707	-2.880238	4.642048
107.H	-3.331877	0.171484	4.330931
108.H	-2.546373	-1.415242	4.490546
109.Au	-0.036503	-0.331676	0.365520
110.N	4.216422	-1.397589	-2.255965
111.N	-4.795166	0.409844	-1.501093
112.N	6.481959	-1.092664	-0.850465
113.N	4.947033	0.970174	-0.019767
114.N	-6.426799	1.593826	0.298149
115.N	-4.062946	2.792187	0.719035
116.N	4.862413	-2.227971	0.990723
117.N	-5.137680	-0.273272	1.765775
118.P	2.185731	-0.677023	0.100653
119.P	-2.239837	0.185340	0.441206
120.Si	2.767935	-1.590451	-3.218390
121.Si	-3.794473	-0.483709	-2.656548
122.Si	3.994777	2.303815	0.650697
123.Si	-2.544431	3.621326	0.980258
124.Si	3.781629	-2.974740	2.160288
125.Si	-4.269888	-1.461835	2.729049
126.W	4.286784	-0.864294	-0.353197
127.W	-4.287112	0.861349	0.356444

Energy: −777.08918089 eV

Table S49. Coordinates and energy for 39

1.C	1.888910	-0.976710	-3.637690
2.C	4.346142	3.711122	-3.046195
3.C	-2.240884	-2.757087	-2.694948
4.C	1.292485	-3.233185	-2.618644
5.C	-3.917428	-0.893240	-2.411981
6.C	-2.050230	3.339298	-2.421595
7.C	-5.769700	0.805819	-2.189223
8.C	1.519652	-1.875422	-2.470642
9.C	0.621956	-5.487803	-1.755868
10.C	-2.810994	-1.578853	-1.940765
11.C	3.864762	3.138672	-1.729330
12.C	-4.510309	0.123126	-1.679809
13.C	2.530283	2.763351	-1.602219
14.C	0.931960	-4.034952	-1.545833
15.C	-0.134121	4.577086	-1.402222
16.C	-1.066145	3.351450	-1.256844
17.C	-0.275974	2.036171	-1.320006
18.C	1.407681	-1.314120	-1.191350
19.C	4.699997	2.967648	-0.662844
20.C	-2.269560	-1.193661	-0.705290
21.C	-3.964419	0.467600	-0.470437

22.C	2.017084	2.213664	-0.397525
23.C	0.794649	-3.452431	-0.304986
24.C	1.039517	-2.074131	-0.101946
25.C	-1.820905	3.481390	0.070507
26.C	-2.818735	-0.145997	0.039126
27.C	4.275935	2.436673	0.544999
28.C	2.935696	2.064118	0.644781
29.C	5.209484	2.230528	1.695472
30.C	3.369856	-3.314681	1.734798
31.C	-3.005936	0.873517	2.270130
32.C	-4.773882	-0.938976	2.554724
33.C	1.110105	-2.221115	2.358090
34.C	2.582705	-2.477879	2.775613
35.C	3.320190	-1.153527	2.941232
36.C	-3.681291	-0.132764	3.236890
37.C	-2.644425	-1.075923	3.840667
38.C	2.544936	-3.225428	4.108937
39.C	-4.275874	0.754894	4.354063
40.H	2.409644	-1.535319	-4.428682
41.H	4.248606	2.978181	-3.861815
42.H	0.986824	-0.530238	-4.084761
43.H	-2.728738	-2.871182	-3.673590
44.H	1.408760	-3.687759	-3.609153
45.H	3.760957	4.597363	-3.335362
46.H	2.532825	-0.152035	-3.306238
47.H	-4.352014	-1.184792	-3.374896
48.H	-5.765671	0.883286	-3.285417
49.H	5.402067	4.009429	-2.989293
50.H	-1.524103	3.277361	-3.389045
51.H	1.319810	-5.950274	-2.469373
52.H	-1.159071	-2.652214	-2.850304
53.H	-2.655363	4.260845	-2.432428
54.H	-5.871731	1.817489	-1.774490
55.H	-2.738305	2.482006	-2.356564
56.H	1.871127	2.919382	-2.455207
57.H	0.379570	4.582488	-2.375924
58.H	-2.396017	-3.690556	-2.131851
59.H	0.203129	1.948364	-2.311257
60.H	-6.669308	0.237852	-1.902375
61.H	-0.392885	-5.624970	-2.163202
62.H	-0.727711	5.504955	-1.333596
63.H	0.676350	-6.052891	-0.815459
64.H	-1.016881	1.215735	-1.297975
65.H	5.750910	3.269327	-0.762627
66.H	1.654131	-0.261785	-1.047897
67.H	0.628903	4.598535	-0.612343
68.H	-1.424532	-1.748474	-0.294280
69.H	-4.435384	1.277084	0.088514
70.H	-2.355463	4.445318	0.112004
71.H	-2.557445	2.676314	0.186906

72.H	0.494410	-4.075069	0.538462
73.H	-1.127883	3.434626	0.923891
74.H	3.431013	-2.800364	0.766632
75.H	6.037719	2.954241	1.676591
76.H	2.557319	1.660416	1.588630
77.H	2.910837	-4.302857	1.568726
78.H	5.655267	1.222948	1.672850
79.H	-4.370770	-1.552891	1.736492
80.H	-3.791040	1.512273	1.832599
81.H	4.399271	-3.477880	2.098438
82.H	-5.557292	-0.289681	2.130041
83.H	3.367347	-0.611682	1.986723
84.H	4.687366	2.329155	2.656028
85.H	0.600470	-3.201251	2.286973
86.H	-2.351397	1.523759	2.859555
87.H	-5.259083	-1.619823	3.276698
88.H	4.353547	-1.329739	3.287732
89.H	0.628281	-1.688679	3.186725
90.H	-2.201825	-1.716337	3.064333
91.H	2.815705	-0.492756	3.663226
92.H	-4.989930	1.487976	3.943069
93.H	2.018281	-4.190534	4.017120
94.H	3.565901	-3.435485	4.467322
95.H	-1.819066	-0.518752	4.308550
96.H	-3.112492	-1.727730	4.599104
97.H	-3.481206	1.306726	4.877573
98.H	2.031738	-2.633209	4.882998
99.H	-4.815331	0.144244	5.097490
100.N	0.699371	1.782426	-0.238168
101.N	0.872998	-1.417306	1.157166
102.N	-2.138947	0.273814	1.208185
103.P	0.376469	1.182305	3.130479
104.Nb	-0.080614	0.404746	1.138754

Energy: –688.31532023 eV

Table S50. Coordinates and energy for 40

1.Mo	-0.050469	1.725917	0.358062
2.P	-0.111542	3.800186	0.787750
3.N	0.170173	1.622179	-1.593641
4.N	-1.775278	1.028617	0.995171
5.N	1.496134	1.048396	1.364678
6.C	1.198373	1.887134	-3.803720
7.C	-3.933665	-0.575064	-3.428270
8.C	-1.690663	-1.793803	-3.226489
9.C	-0.490733	3.497030	-3.073059
10.C	-2.457724	-0.620199	-3.045102
11.C	0.333096	-3.135764	-2.968490
12.C	-0.394382	-1.872933	-2.776005
13.C	0.650119	2.606504	-2.653781
14.C	-1.840673	0.490109	-2.512814

15.C	4.821326	-0.616721	-1.982820
16.C	1.756846	3.431758	-2.073479
17.C	0.211122	-0.727644	-2.245011
18.C	-0.491980	0.435355	-2.136918
19.C	3.751015	-0.876134	-0.927158
20.C	3.367361	-2.192516	-0.582835
21.C	3.135377	0.175039	-0.284591
22.C	-1.973106	-3.851872	0.160614
23.C	-3.363510	2.781006	0.340165
24.C	2.324480	-2.426279	0.281629
25.C	1.958182	-3.820681	0.568818
26.C	-1.830090	-1.378082	0.604741
27.C	2.133481	-0.067638	0.664399
28.C	1.720373	-1.340176	0.926361
29.C	-1.723984	-2.703338	1.043693
30.C	-4.199872	0.700846	1.155603
31.C	-3.118503	1.672929	1.317287
32.C	-1.642164	-0.347082	1.476703
33.C	3.342681	2.545814	2.063651
34.C	-1.472614	-2.947321	2.372921
35.C	2.295890	1.577068	2.549601
36.C	-3.095294	2.220085	2.721025
37.C	-1.296777	-0.596813	2.811480
38.C	2.911416	0.469852	3.281473
39.C	-1.193616	-1.891259	3.270431
40.C	1.356918	2.267505	3.489951
41.C	-0.788755	-2.169318	4.714741
42.H	1.596267	2.605083	-4.538923
43.H	-4.077184	-0.825338	-4.489705
44.H	0.453747	1.270803	-4.330303
45.H	-2.166595	-2.675485	-3.665386
46.H	2.028608	1.223891	-3.514407
47.H	1.127352	-3.035764	-3.726515
48.H	-0.331535	-3.943637	-3.301235
49.H	-4.355937	0.421656	-3.254357
50.H	-4.517719	-1.299787	-2.842269
51.H	4.519274	-1.018494	-2.961060
52.H	-2.398773	1.411624	-2.354876
53.H	5.008152	0.457407	-2.099193
54.H	0.836790	-3.466527	-2.048347
55.H	5.771737	-1.100156	-1.712844
56.H	1.253529	-0.757473	-1.925913
57.H	3.863035	-3.034785	-1.074446
58.H	-1.379408	-3.798701	-0.763814
59.H	3.400522	1.203244	-0.525183
60.H	-3.026827	-3.896541	-0.160841
61.H	-2.075217	-1.163954	-0.436072
62.H	2.463228	-4.523208	-0.106830
63.H	-4.227230	0.283949	0.136466
64.H	-1.745578	-4.804801	0.655928

65.H	0.874241	-3.988392	0.486367
66.H	-5.171416	1.189208	1.333603
67.H	2.229225	-4.108311	1.598094
68.H	0.922901	-1.507972	1.650946
69.H	-4.135736	-0.146437	1.855346
70.H	-1.411079	-3.978600	2.732370
71.H	3.650853	-0.089802	2.688493
72.H	-1.086550	0.243151	3.471489
73.H	2.160936	-0.253209	3.638068
74.H	3.439234	0.855426	4.168514
75.H	0.150348	-2.739856	4.760532
76.H	-1.554691	-2.762372	5.235740
77.H	-0.646605	-1.235036	5.270877
78.H	-0.143423	4.272600	-3.775583
79.H	-1.281377	2.927166	-3.581370
80.H	2.168586	4.101253	-2.845332
81.H	-0.926053	4.001675	-2.197801
82.H	2.574774	2.793396	-1.711368
83.H	1.421020	4.052708	-1.234941
84.H	-3.328338	2.408442	-0.693164
85.H	-2.627909	3.588769	0.429518
86.H	-4.362204	3.214163	0.508250
87.H	4.096531	2.045480	1.439463
88.H	2.877579	3.352109	1.477461
89.H	3.874487	3.005143	2.913208
90.H	-4.028422	2.763568	2.943750
91.H	-2.255816	2.919647	2.847275
92.H	0.890821	3.152938	3.042161
93.H	-3.000384	1.416886	3.465423
94.H	0.552295	1.590550	3.809212
95.H	1.901401	2.592394	4.390755

Energy: –630.28182701 eV

Table S51. Coordinates and energy for 41

1.Mo	-0.006594	0.914784	0.097696
2.P	-0.021711	3.013775	0.321866
3.N	-1.018918	0.648444	-1.587228
4.N	1.930664	0.500471	-0.002736
5.N	-0.922149	0.293375	1.744008
6.C	-0.255973	2.768196	-5.721717
7.C	-1.665940	3.010831	-5.218549
8.C	-2.370509	1.684024	-5.032154
9.C	0.509770	1.894977	-4.722434
10.C	-0.215340	0.574200	-4.523053
11.C	2.863303	-1.669871	-4.386965
12.C	-1.625718	0.817095	-4.022325
13.C	-1.620451	3.715116	-3.872495
14.C	0.561262	2.626871	-3.386520
15.C	2.640719	-1.750962	-2.898497
16.C	-0.840320	2.851667	-2.857500

17.C	-0.903109	-4.248931	-2.568093
18.C	-1.595231	1.541380	-2.651983
19.C	2.592519	-2.980518	-2.267349
20.C	2.450742	-0.617236	-2.152258
21.C	-1.635254	-3.016247	-2.040902
22.C	-0.985029	-1.778298	-2.001254
23.C	-2.939959	-3.092334	-1.593637
24.C	-1.669190	-0.643165	-1.585005
25.C	-3.633035	-1.983621	-1.143191
26.C	-3.012691	-0.761791	-1.167347
27.C	2.332104	-3.107719	-0.916755
28.C	-5.032664	-2.130795	-0.603710
29.C	2.148854	-0.702105	-0.775601
30.C	2.320454	-4.474836	-0.235342
31.C	2.073477	-1.951522	-0.172997
32.C	3.209433	1.275361	0.162983
33.C	5.660909	1.132952	0.669573
34.C	4.347384	0.357984	0.679549
35.C	4.451114	3.229013	1.088683
36.C	3.115755	2.454616	1.127125
37.C	-1.320848	-4.672809	1.372709
38.C	5.577238	2.336527	1.583897
39.C	-3.462532	2.070477	1.551788
40.C	-1.046031	-2.157523	1.545499
41.C	-0.627998	-3.433809	1.936914
42.C	-4.510500	1.126966	2.130163
43.C	4.054315	-0.101766	2.094349
44.C	-0.462573	-1.028781	2.107068
45.C	-3.840969	-0.197493	2.458073
46.C	2.852945	2.009266	2.550990
47.C	-2.329989	2.264612	2.538939
48.C	5.286499	1.876717	2.999557
49.C	0.416692	-3.541043	2.834253
50.C	3.971081	1.091799	3.031612
51.C	-1.641341	0.949042	2.891183
52.C	0.580431	-1.185845	3.045687
53.C	-5.076661	1.758641	3.406049
54.C	1.036752	-2.433371	3.382967
55.C	-2.730200	0.009062	3.469241
56.C	-2.901474	2.882916	3.833335
57.C	2.215488	-2.603007	4.306786
58.C	-3.963982	1.965366	4.415637
59.C	-3.315279	0.634516	4.731205
60.H	-0.286936	2.275153	-6.710712
61.H	-2.219508	3.631210	-5.945182
62.H	0.272748	3.727963	-5.865533
63.H	-2.436005	1.142907	-5.993535
64.H	-0.256960	0.013934	-5.475525
65.H	1.533485	1.715850	-5.095319
66.H	1.993944	-2.057615	-4.938849

67.H	-3.411549	1.848253	-4.699572
68.H	3.734371	-2.265339	-4.696374
69.H	3.026229	-0.635794	-4.713447
70.H	-1.124944	4.697705	-3.961516
71.H	-2.156153	-0.141758	-3.917069
72.H	0.338873	-0.054612	-3.810141
73.H	-2.643384	3.907055	-3.503706
74.H	-0.649561	-4.125525	-3.630994
75.H	1.070687	3.600919	-3.499825
76.H	2.770657	-3.885228	-2.858195
77.H	-1.523587	-5.149616	-2.472859
78.H	1.147916	2.054347	-2.649796
79.H	2.498835	0.366675	-2.619912
80.H	-2.637231	1.780988	-2.362775
81.H	0.048727	-1.687647	-2.332682
82.H	0.038464	-4.407933	-2.027065
83.H	-0.795601	3.395655	-1.899887
84.H	-3.444364	-4.064257	-1.599661
85.H	-5.674382	-2.691518	-1.298703
86.H	2.469505	-5.282211	-0.964184
87.H	-3.539557	0.132298	-0.832883
88.H	3.500438	1.662153	-0.833166
89.H	-5.498948	-1.154714	-0.424249
90.H	5.907505	1.452199	-0.359353
91.H	-5.033463	-2.678429	0.350624
92.H	4.659915	3.579116	0.062569
93.H	1.372513	-4.643086	0.291476
94.H	4.443773	-0.513942	0.014646
95.H	3.124147	-4.543650	0.512184
96.H	-1.311436	-4.662640	0.275405
97.H	6.478916	0.461658	0.988176
98.H	-3.063453	1.676237	0.602991
99.H	2.311677	3.130220	0.792469
100.H	1.849769	-2.019011	0.891045
101.H	-1.857153	-2.039006	0.828065
102.H	6.530930	2.892669	1.560859
103.H	-3.914660	3.050591	1.315170
104.H	-5.324644	0.972520	1.400491
105.H	4.360227	4.130217	1.719858
106.H	-2.373876	-4.707142	1.687864
107.H	-0.834825	-5.592884	1.722918
108.H	-3.440541	-0.653274	1.540095
109.H	3.111672	-0.668531	2.123280
110.H	-1.584626	2.960414	2.120227
111.H	4.851366	-0.792811	2.426281
112.H	1.878029	1.497962	2.605288
113.H	-4.581665	-0.908327	2.869204
114.H	-5.563100	2.721287	3.165658
115.H	6.114869	1.245016	3.369513
116.H	0.763711	-4.538596	3.123567

117.H	2.774784	2.896968	3.204339
118.H	5.224056	2.743848	3.681691
119.H	-3.326564	3.874374	3.598821
120.H	-5.860992	1.108955	3.835860
121.H	1.039362	-0.294435	3.474545
122.H	-0.896463	1.147673	3.686390
123.H	-2.275926	-0.959142	3.729648
124.H	3.097053	-2.969658	3.759915
125.H	3.766569	0.753717	4.062590
126.H	-2.092802	3.049183	4.566554
127.H	2.493553	-1.654921	4.782411
128.H	2.001835	-3.332342	5.101465
129.H	-4.375443	2.410696	5.338606
130.H	-4.053304	-0.060913	5.170615
131.H	-2.525442	0.766502	5.492877

Energy: −885.06158748 eV

Table S52. Coordinates and energy for 42

1.Mo	-1.378916	1.322443	0.067084
2.H	-0.879539	-2.960412	-5.302766
3.H	-2.484337	3.068362	-4.301295
4.H	-2.242256	-2.742939	-4.190295
5.H	-2.055893	1.351569	-4.374177
6.H	0.049203	3.758590	-4.143540
7.H	0.620444	2.078825	-4.152963
8.H	-0.996891	-3.962090	-3.851604
9.H	1.518293	-2.981137	-3.678107
10.H	-3.101733	1.957795	-3.056144
11.H	-2.042915	-0.588918	-3.120878
12.H	3.844790	-0.718075	-3.116195
13.H	3.532139	-2.412333	-2.680295
14.H	0.994615	3.135985	-2.772184
15.H	-1.331810	3.338273	-2.189691
16.H	4.088557	2.531038	-1.762070
17.H	1.704029	0.766765	-1.606959
18.H	3.582080	-1.150526	-1.417499
19.H	-3.799859	-1.140880	-1.527120
20.H	-4.415836	0.480611	-1.110651
21.H	-5.410824	-0.964371	-0.781729
22.H	5.429100	2.196832	-0.652387
23.H	1.244252	-3.472716	-0.837018
24.H	4.421997	3.640833	-0.431985
25.H	-0.494890	-1.778961	-0.616785
26.H	1.743170	3.054272	-0.121239
27.H	1.127636	-4.819907	0.324029
28.H	2.306602	-3.522405	0.588414
29.H	4.988126	0.384649	0.727302
30.H	-3.750664	-1.761469	0.856304
31.H	-4.602523	1.104643	1.450839
32.H	-5.627894	-0.344930	1.596005

33.H	4.363579	-1.896125	1.711742
34.H	-1.050413	4.420743	1.653871
35.H	0.732879	4.454889	1.702460
36.H	-4.212857	-0.147679	2.652996
37.H	1.183807	-0.321846	2.383364
38.H	2.789073	-2.032629	2.541273
39.H	0.787676	-3.948949	2.891080
40.H	4.109218	-1.098233	3.270802
41.H	-0.203175	4.824623	3.169396
42.H	-2.234861	2.517437	3.037861
43.H	-2.277496	-1.010897	3.193707
44.H	0.782806	2.537203	3.188907
45.H	-1.204212	1.287008	3.794245
46.H	-1.238015	2.941295	4.442939
47.H	-1.785089	-3.501810	4.841721
48.H	-0.031915	-3.345733	5.079887
49.H	-1.091094	-1.924700	5.263366
50.P	-2.864057	2.827930	0.137764
51.C	-1.165272	-2.946696	-4.239289
52.C	-2.231256	2.191039	-3.683931
53.C	0.219124	2.875754	-3.506323
54.C	-0.376251	-1.927139	-3.484499
55.C	1.031805	-2.077832	-3.299758
56.C	-0.969901	-0.740747	-3.010184
57.C	-1.042698	2.484346	-2.820463
58.C	1.756726	-1.138917	-2.622801
59.C	3.268938	-1.373699	-2.445888
60.C	-0.208583	0.192977	-2.343900
61.C	1.134867	0.013683	-2.148512
62.C	-4.383083	-0.571384	-0.791116
63.C	4.400429	2.575593	-0.706666
64.C	3.487818	1.798869	0.167654
65.C	1.291361	-3.736754	0.225585
66.C	2.152313	2.171646	0.374812
67.C	-0.564731	-1.970871	0.454900
68.C	-3.753126	-0.687445	0.603543
69.C	3.938421	0.673021	0.858622
70.C	0.250000	-2.957213	1.029340
71.C	1.330169	1.409686	1.174013
72.C	-1.504017	-1.329759	1.196546
73.C	-4.589466	0.017981	1.634848
74.C	3.123856	-0.110712	1.611242
75.C	1.865170	0.276243	1.768643
76.C	-0.167513	4.187039	2.271710
77.C	3.622507	-1.356444	2.317436
78.C	0.143445	-3.188407	2.439823
79.C	-1.564477	-1.572826	2.589835
80.C	-0.163404	2.729842	2.649637
81.C	-0.756866	-2.530006	3.180806
82.C	-1.255415	2.349368	3.513930

83.C	-0.920834	-2.840353	4.682075
84.N	-0.797940	1.406359	-1.820155
85.N	-2.323239	-0.293656	0.573056
86.N	-0.036761	1.862852	1.405934

Energy: −575.66495113 eV

Table S53. Coordinates and energy for 43

1.C	2.590321	-0.153901	-4.060257
2.C	1.418609	0.813864	-4.038823
3.C	1.706784	1.998142	-3.145784
4.C	2.954739	-0.591604	-2.662079
5.C	3.241405	0.594965	-1.761340
6.C	2.098447	1.604307	-1.734030
7.C	2.436876	2.809794	-0.883062
8.C	-1.553994	-4.063187	-0.250412
9.C	-0.043337	-4.090023	-0.267248
10.C	-2.915797	3.821706	0.335940
11.C	-3.582363	2.455899	0.350713
12.C	-1.437347	3.704049	0.618104
13.C	2.096931	-3.132489	0.635026
14.C	0.585842	-3.040496	0.629514
15.C	-2.088148	-4.182859	1.167440
16.C	-3.323398	1.737688	1.654632
17.C	-1.173152	2.980287	1.924943
18.C	-1.506266	-3.109937	2.055989
19.C	-1.848920	1.614627	1.989776
20.C	0.010552	-3.131404	2.039003
21.C	-1.626519	0.943384	3.328369
22.H	1.185349	1.161907	-5.057939
23.H	2.355668	-1.027607	-4.690145
24.H	3.463046	0.338679	-4.531294
25.H	0.518690	0.291643	-3.673016
26.H	2.539809	2.589605	-3.569531
27.H	0.837553	2.675088	-3.096035
28.H	3.829924	-1.261721	-2.678391
29.H	2.125650	-1.180687	-2.235110
30.H	4.148473	1.118817	-2.112805
31.H	3.329757	3.319535	-1.273322
32.H	0.342153	-3.957278	-1.292043
33.H	-1.949232	-4.874324	-0.882942
34.H	1.603217	3.526154	-0.877704
35.H	3.445505	0.271172	-0.727359
36.H	-3.086016	4.321549	-0.631702
37.H	-1.906642	-3.118402	-0.696953
38.H	-3.192406	1.848375	-0.483110
39.H	2.492407	-3.067344	-0.388518
40.H	-0.950023	3.155427	-0.205584
41.H	0.314942	-5.078831	0.075537
42.H	2.640831	2.503131	0.152959
43.H	-4.667642	2.552240	0.185912

44.H	-0.964182	4.699293	0.645781
45.H	-3.387262	4.466885	1.102112
46.H	2.423903	-4.087180	1.072309
47.H	-3.189322	-4.131383	1.168810
48.H	2.529818	-2.315176	1.229548
49.H	-1.827036	-5.179319	1.573473
50.H	-3.769187	0.728813	1.644336
51.H	-1.859686	-2.121858	1.716031
52.H	-0.092352	2.842722	2.094284
53.H	-3.808967	2.285371	2.483913
54.H	0.374755	-4.070345	2.493688
55.H	0.428891	-2.307417	2.640322
56.H	-1.550241	3.585401	2.768876
57.H	-1.864821	-3.224474	3.092096
58.H	-2.122046	-0.037421	3.356396
59.H	-0.552221	0.797017	3.511152
60.H	-2.033759	1.560391	4.142425
61.O	0.927375	0.943224	-1.175148
62.O	0.191060	-1.757843	0.064048
63.O	-1.279489	0.780367	0.940695
64.P	1.768382	0.377213	1.872725
65.Mo	0.332067	0.070633	0.351020

Energy: −417.28775991 eV

Table S54. Coordinates and energy for 44

1.C	2.014430	-0.245054	-3.034603
2.C	4.113705	-1.599125	-2.813088
3.C	-2.777564	-0.041986	-2.290817
4.C	2.704284	-1.369748	-2.266776
5.C	-4.734330	-1.432469	-1.541049
6.C	-3.222472	-1.235185	-1.442099
7.C	5.591855	1.737916	-0.667037
8.C	4.900736	2.943767	-0.662857
9.C	4.896491	0.546274	-0.501544
10.C	0.516257	2.566596	-0.736115
11.C	3.533561	2.958648	-0.443135
12.C	-0.890759	2.554950	-0.456542
13.C	3.506167	0.535083	-0.325851
14.C	2.823247	1.767037	-0.268762
15.C	3.177877	-3.696748	0.071373
16.C	1.374552	1.796210	-0.013012
17.C	3.687663	-2.274466	0.321856
18.C	-2.852105	-3.831465	0.685001
19.C	-1.389858	1.754586	0.522206
20.C	-2.828684	1.660832	0.825542
21.C	-3.446186	0.398925	0.886110
22.C	-3.569359	2.816195	1.103173
23.C	-4.793164	0.336523	1.260186
24.C	-3.329804	-2.471170	1.201278
25.C	-4.902986	2.729944	1.475881
26.C	0.891241	0.945251	1.061019

27.C	-5.512589	1.486868	1.567548
28.C	-0.517075	0.915744	1.325092
29.C	3.927964	-1.998516	1.804957
30.C	-3.136164	-2.343589	2.712435
31.H	1.963488	-0.500939	-4.104498
32.H	4.040888	-1.873328	-3.877864
33.H	-3.087318	-0.195816	-3.336125
34.H	4.729109	-0.690072	-2.754888
35.H	2.564734	0.703334	-2.943995
36.H	-5.008811	-1.564623	-2.599926
37.H	0.983427	-0.084694	-2.691548
38.H	4.648249	-2.417160	-2.309252
39.H	2.103290	-2.283174	-2.393837
40.H	-1.686016	0.080343	-2.286346
41.H	-3.234773	0.895615	-1.941351
42.H	-2.705548	-2.129115	-1.825073
43.H	0.891928	3.174860	-1.559637
44.H	-5.287802	-0.558006	-1.168575
45.H	-5.089326	-2.324437	-1.005468
46.H	6.673855	1.723057	-0.802313
47.H	5.439721	3.883328	-0.795253
48.H	3.083917	-3.923144	-0.998682
49.H	-1.564320	3.154220	-1.070183
50.H	5.454605	-0.387874	-0.523712
51.H	2.994182	3.904248	-0.384872
52.H	-3.057951	-3.968792	-0.384420
53.H	4.646256	-2.159755	-0.207196
54.H	3.877696	-4.424332	0.510328
55.H	2.191981	-3.848314	0.532874
56.H	-1.770070	-3.949682	0.832825
57.H	-4.405494	-2.381723	0.983999
58.H	-3.066880	3.782895	1.061158
59.H	-3.366743	-4.634475	1.234891
60.H	-5.302173	-0.624120	1.321181
61.H	-5.462960	3.635947	1.712483
62.H	-6.555913	1.406207	1.874905
63.H	4.324881	-0.988782	1.978010
64.H	1.597347	0.752633	1.865688
65.H	4.654520	-2.721259	2.207233
66.H	2.998002	-2.105750	2.384058
67.H	-0.878147	0.682036	2.324029
68.H	-2.076442	-2.459210	2.985578
69.H	-3.485393	-1.377034	3.099859
70.H	-3.699245	-3.137636	3.226730
71.P	2.550899	-1.028921	-0.442055
72.P	-2.523155	-1.077474	0.283387
73.P	0.353160	-2.209494	2.022547
74.Cl	-0.211151	-2.576732	-1.536086
75.Mo	0.071954	-1.019204	0.315125

Energy: –498.48696398 eV

Table S55. Coordinates and energy for 45

1.W	0.072648	-0.006530	-0.026887
2.P	2.097711	-0.184599	-0.760363
3.Si	-0.201155	0.705107	-3.259150
4.Si	0.869544	-3.162666	0.633573
5.Si	1.749769	2.244756	1.749113
6.N	-0.906514	0.527065	-1.657141
7.N	-0.283862	-1.834008	0.633778
8.N	-2.122695	0.186789	0.769478
9.N	0.262849	1.388556	1.359665
10.C	-1.492603	1.187411	-4.470223
11.C	0.498705	-0.896682	-3.878125
12.C	1.069125	2.033004	-3.304621
13.C	-2.371075	0.629206	-1.604771
14.C	1.381102	-3.625446	-1.070286
15.C	-2.840139	0.982729	-0.221149
16.C	2.375525	3.218605	0.300425
17.C	-2.632923	-1.176357	0.874672
18.C	0.108564	-4.662648	1.367119
19.C	-1.543217	-2.100405	1.341709
20.C	2.349170	-2.781585	1.684365
21.C	-0.943911	1.898705	2.024197
22.C	-2.023244	0.853291	2.063884
23.C	3.048954	1.108507	2.381614
24.C	1.445453	3.469826	3.080933
25.H	-1.020689	1.256580	-5.464383
26.H	0.814870	-0.794187	-4.930264
27.H	-2.314688	0.459844	-4.564822
28.H	-1.945219	2.173388	-4.274956
29.H	1.391506	2.208909	-4.345340
30.H	-0.248368	-1.705953	-3.837972
31.H	1.371886	-1.221843	-3.296092
32.H	0.673471	2.986098	-2.918614
33.H	1.964081	1.782581	-2.718321
34.H	-2.748336	1.398962	-2.294765
35.H	-2.840902	-0.317921	-1.934375
36.H	0.510672	-3.831996	-1.713389
37.H	1.977679	-2.843236	-1.559999
38.H	1.993869	-4.542862	-1.042885
39.H	2.712847	2.572342	-0.521498
40.H	-3.936461	0.860437	-0.119851
41.H	1.597940	3.888596	-0.101120
42.H	-2.955781	-1.488832	-0.129432
43.H	-2.608472	2.040572	-0.028539
44.H	3.228019	3.850295	0.603137
45.H	-0.744365	-5.060066	0.792980
46.H	-1.886450	-3.133549	1.182561
47.H	0.869923	-5.460278	1.380768
48.H	2.956347	-1.966602	1.266932

49.H	-3.519188	-1.225226	1.537404
50.H	2.995774	-3.671346	1.771810
51.H	-1.315807	2.808659	1.513978
52.H	3.415293	0.415078	1.611797
53.H	-0.226079	-4.532771	2.408798
54.H	-2.996055	1.285141	2.370874
55.H	-1.404759	-2.000929	2.436137
56.H	2.055118	-2.488410	2.705226
57.H	3.913724	1.691369	2.742855
58.H	0.696012	4.235795	2.824321
59.H	-1.751578	0.091975	2.809873
60.H	-0.736271	2.207803	3.059573
61.H	2.388700	4.007887	3.272590
62.H	2.683701	0.505007	3.227818
63.H	1.144617	3.022494	4.042417

Energy: –389.76866904 eV

Table S56. Coordinates and energy for 46

1.C	-2.647429	1.797000	-4.267684
2.C	-1.322672	-0.264665	-3.758495
3.C	-0.004138	-2.346469	-3.377388
4.C	-2.133989	0.771574	-3.290709
5.C	2.342427	0.746958	-3.271263
6.C	-0.840051	-1.221903	-2.880708
7.C	-2.456793	0.805804	-1.984879
8.C	2.317326	1.288988	-1.874911
9.C	-1.191180	-1.186388	-1.520720
10.C	3.510456	1.551464	-1.177187
11.C	1.112730	1.571092	-1.229448
12.C	-2.054076	-0.215140	-1.100864
13.C	3.516851	-2.204945	-0.880978
14.C	1.525233	-3.577144	-0.180613
15.C	2.270038	-2.369100	-0.104132
16.C	-4.864785	0.069542	0.182733
17.C	3.486734	2.043917	0.110766
18.C	1.045339	2.036765	0.066734
19.C	-3.772640	-2.082102	0.274319
20.C	-0.408358	-5.106625	0.452045
21.C	-1.956439	4.037688	0.405524
22.C	0.363778	-3.790305	0.553152
23.C	4.778088	2.310514	0.850252
24.C	-3.714407	-0.641824	0.719702
25.C	-0.514608	3.780955	0.750049
26.C	2.280300	2.265859	0.743904
27.C	1.793648	-1.366778	0.760719
28.C	-0.075572	-2.778043	1.401530
29.C	0.624483	-1.580066	1.493396
30.C	-0.079643	4.411969	2.038430
31.C	0.553236	-0.572556	3.770104
32.C	2.041754	-0.285979	3.917000

33.C	0.223799	-1.911237	4.425840
34.H	-1.872556	2.096325	-4.986816
35.H	-1.047691	-0.311881	-4.815205
36.H	-3.489812	1.391596	-4.849659
37.H	0.376350	-2.162255	-4.391451
38.H	-3.007110	2.700192	-3.757998
39.H	1.764320	1.382574	-3.957544
40.H	3.368441	0.678570	-3.656046
41.H	1.892687	-0.254968	-3.314772
42.H	-0.584479	-3.282347	-3.415229
43.H	0.850425	-2.538555	-2.714241
44.H	3.501487	-2.790297	-1.811170
45.H	4.469079	1.364769	-1.670351
46.H	-3.064563	1.618119	-1.583115
47.H	0.174860	1.406209	-1.766522
48.H	-4.909576	0.023291	-0.916763
49.H	3.695886	-1.153078	-1.136702
50.H	1.879816	-4.363868	-0.853520
51.H	-0.808172	-1.935420	-0.830516
52.H	-3.794702	-2.164243	-0.822423
53.H	4.391868	-2.550051	-0.305491
54.H	-2.209334	3.586444	-0.563554
55.H	-0.000247	-5.744875	-0.341242
56.H	0.098699	4.205801	-0.064254
57.H	5.361415	3.101557	0.355286
58.H	-1.471167	-4.928483	0.237203
59.H	-2.149101	5.120135	0.339950
60.H	-4.876175	1.130322	0.480223
61.H	-5.795357	-0.381653	0.561630
62.H	-2.912510	-2.659582	0.640553
63.H	-4.687425	-2.560198	0.659772
64.H	5.415330	1.414728	0.884165
65.H	2.313809	-0.412998	0.848535
66.H	-2.626307	3.615778	1.168593
67.H	-0.353157	-5.664920	1.397777
68.H	4.590775	2.630766	1.882188
69.H	2.260926	2.610398	1.777830
70.H	-3.719608	-0.596298	1.815779
71.H	-0.315101	5.488114	2.043064
72.H	-0.990738	-2.897369	1.981884
73.H	1.001807	4.310367	2.195587
74.H	-0.602286	3.944509	2.888582
75.H	2.303987	0.683611	3.470556
76.H	2.649460	-1.063365	3.430317
77.H	0.792837	-2.736365	3.974288
78.H	-0.849563	-2.139429	4.351638
79.H	-0.018967	0.221639	4.263154
80.H	2.320535	-0.259683	4.981795
81.H	0.483788	-1.870305	5.494675
82.N	-2.346398	-0.071788	0.344091

83.N	-0.231899	2.313849	0.711648
84.N	0.138547	-0.509454	2.342683
85.P	-2.240133	1.518940	3.103163
86.W	-1.080707	0.764752	1.494121

Energy: −576.32560108 eV

Table S57. Coordinates and energy for 47

1.C	2.822932	0.041619	-3.970304
2.C	-0.241560	2.549363	-3.862532
3.C	1.728501	-2.208056	-3.793655
4.C	-3.211981	0.180440	-3.029784
5.C	-1.483947	4.504662	-2.893450
6.C	2.260766	-0.890218	-3.020071
7.C	-0.551627	3.269731	-2.594508
8.C	4.436380	-1.829149	-2.066846
9.C	1.282393	4.935498	-2.017124
10.C	-4.448820	-1.739264	-1.957250
11.C	0.740651	3.660025	-1.895605
12.C	3.120472	-1.403779	-1.862885
13.C	-3.452400	-0.547905	-1.737351
14.C	2.502055	5.257389	-1.478618
15.C	-1.581642	-4.113747	-1.365989
16.C	5.200685	-2.332776	-1.027131
17.C	1.461990	2.700214	-1.149745
18.C	3.229597	4.298992	-0.805818
19.C	4.884026	1.642336	-0.676443
20.C	0.696042	-4.969018	-0.654140
21.C	2.738843	3.003182	-0.623954
22.C	-3.899975	0.392655	-0.630566
23.C	-0.485237	-3.865450	-0.536105
24.C	2.583549	-1.520928	-0.547113
25.C	-5.255487	0.514834	-0.268074
26.C	3.609495	1.974640	0.074481
27.C	-2.972079	1.232050	0.027895
28.C	4.659618	-2.451683	0.238991
29.C	-5.676385	1.441893	0.676678
30.C	3.335730	-2.070637	0.512531
31.C	-3.401503	2.207328	0.959860
32.C	-0.779762	-3.646818	0.945619
33.C	-4.771664	2.275608	1.265541
34.C	-2.858783	4.645822	1.355175
35.C	3.976622	2.424984	1.523306
36.C	-1.176176	-2.375980	1.437882
37.C	-2.438643	3.189588	1.605683
38.C	-0.738597	-4.722468	1.842744
39.C	2.741523	-2.272234	1.901145
40.C	2.794129	-3.650999	2.445488
41.C	-1.622675	-2.239439	2.773086
42.C	-3.844654	-1.012725	2.933346
43.C	3.418439	-1.237159	2.914815

44.C	-1.109567	-4.585368	3.161832
45.C	-2.323847	2.970722	3.128285
46.C	-2.255574	-0.932615	3.253740
47.C	-1.562301	-3.356514	3.609558
48.C	-2.074504	-0.605341	4.700167
49.H	2.092238	0.290893	-4.755479
50.H	1.014467	-1.923748	-4.583191
51.H	0.330984	3.190263	-4.554419
52.H	3.713323	-0.355160	-4.490835
53.H	-1.158440	2.233300	-4.387367
54.H	2.578923	-2.732947	-4.255313
55.H	-2.818021	-0.500240	-3.802703
56.H	-1.041969	5.161756	-3.658166
57.H	0.359064	1.648737	-3.675448
58.H	3.123868	0.992061	-3.503330
59.H	-2.450340	4.147913	-3.277697
60.H	-4.144222	0.624185	-3.417930
61.H	1.234125	-2.890635	-3.091054
62.H	4.872775	-1.745785	-3.065703
63.H	-2.485263	0.992632	-2.900392
64.H	-4.023486	-2.448283	-2.681720
65.H	0.725409	5.697008	-2.566441
66.H	1.374698	-0.435790	-2.561926
67.H	-5.407150	-1.385978	-2.368646
68.H	-1.292588	-4.187272	-2.429753
69.H	-1.667486	5.096594	-1.987023
70.H	-1.101425	2.598885	-1.920961
71.H	2.902243	6.267657	-1.592820
72.H	1.042962	-5.018782	-1.697884
73.H	4.681107	1.300773	-1.701341
74.H	6.231295	-2.644541	-1.211005
75.H	-2.503505	-0.991036	-1.403436
76.H	-2.111638	-5.055673	-1.123273
77.H	-2.339058	-3.318321	-1.301749
78.H	-4.646802	-2.274787	-1.019884
79.H	5.551749	2.518789	-0.742805
80.H	-5.993432	-0.131521	-0.744699
81.H	0.003544	-2.934596	-0.861248
82.H	4.214400	4.548851	-0.402892
83.H	0.335860	-5.971387	-0.378199
84.H	5.431782	0.837843	-0.162443
85.H	1.541963	-4.692026	-0.019098
86.H	3.021825	1.053899	0.169633
87.H	-2.957542	4.861504	0.281048
88.H	-6.735816	1.503363	0.935802
89.H	5.262061	-2.872480	1.048408
90.H	-1.446158	3.028319	1.166332
91.H	4.557207	3.361216	1.509806
92.H	-0.423144	-5.701551	1.480306
93.H	-2.109838	5.336487	1.772851

94.H	-3.825523	4.872859	1.831669
95.H	-5.109550	3.019501	1.991966
96.H	2.286922	-4.378148	1.797092
97.H	4.590286	1.654044	2.012462
98.H	1.688193	-1.969257	1.840945
99.H	-4.011203	-1.203429	1.866274
100.H	3.071999	2.584279	2.126337
101.H	3.829403	-4.007843	2.591898
102.H	3.283504	-0.210956	2.556099
103.H	-1.852119	-0.120843	2.638527
104.H	4.494816	-1.450330	3.018220
105.H	-4.327047	-0.061683	3.202666
106.H	2.293081	-3.693819	3.424757
107.H	-4.290563	-1.829045	3.523255
108.H	-1.957765	1.963804	3.360461
109.H	-1.614485	3.691724	3.563734
110.H	-3.300368	3.113092	3.619472
111.H	-1.063416	-5.438018	3.842572
112.H	2.943816	-1.324731	3.903835
113.H	-1.894396	-3.254090	4.645597
114.H	-2.546380	0.360270	4.936729
115.H	-1.011224	-0.530850	4.976871
116.H	-2.540162	-1.354406	5.361447
117.O	0.922335	1.454871	-1.035045
118.O	1.294285	-1.148914	-0.353687
119.O	-1.654907	1.135085	-0.303833
120.O	-1.197225	-1.311830	0.592664
121.P	0.618885	0.921989	2.107131
122.W	0.014334	0.217257	0.178137

Energy: –817.66419714 eV

Table S58. Coordinates and energy for 48

1.C	-1.645354	1.595454	-5.914228
2.C	-1.850658	2.738576	-5.176498
3.C	-1.217789	0.457069	-5.251549
4.C	-1.008788	0.515221	-3.886652
5.C	-1.236254	1.719853	-3.219712
6.C	2.723478	0.178321	-2.278263
7.C	-0.999798	1.832225	-1.789269
8.C	4.214729	-1.613038	-1.327602
9.C	1.745733	-1.891372	-1.405096
10.C	2.871928	-0.875691	-1.170919
11.C	-1.283099	2.875920	-0.902748
12.C	-1.624055	-2.243422	-0.466984
13.C	5.088869	1.320553	-0.015981
14.C	-3.444792	-0.640371	0.031848
15.C	2.909308	2.594783	0.082706
16.C	-0.876808	2.396261	0.330579
17.C	3.724069	1.438169	0.681433
18.C	-2.643824	-1.824969	0.597842

19.C	-3.578863	-3.017730	0.832232
20.C	3.471231	-1.338281	1.682821
21.C	2.520361	-2.513275	1.868604
22.C	4.016916	1.757923	2.154530
23.C	0.296920	-3.250267	2.293268
24.C	-1.045933	-2.850547	2.883458
25.C	-3.038204	0.942908	3.035536
26.C	-4.125252	-1.251264	3.553262
27.C	-2.783668	-0.522552	3.408720
28.C	-2.093681	-0.559401	4.777311
29.H	-1.820608	1.594378	-6.991015
30.H	-2.193473	3.654020	-5.668047
31.H	-1.050178	-0.474688	-5.795882
32.H	-0.675144	-0.352176	-3.321371
33.H	2.721949	-0.344042	-3.249329
34.H	4.292970	-1.967672	-2.369212
35.H	3.543339	0.905070	-2.309346
36.H	1.924808	-2.390434	-2.372452
37.H	1.769613	0.714687	-2.190252
38.H	5.092785	-0.987129	-1.131780
39.H	0.782125	-1.375426	-1.466501
40.H	-2.170431	-2.639498	-1.339874
41.H	5.017007	1.188151	-1.100606
42.H	-1.723522	3.838177	-1.135394
43.H	-3.840632	-0.926317	-0.956195
44.H	4.275180	-2.502931	-0.684101
45.H	2.664489	2.444114	-0.974245
46.H	1.686490	-2.668630	-0.634047
47.H	-1.046020	-1.378392	-0.814319
48.H	5.646988	2.255564	0.158191
49.H	-4.076703	-3.254126	-0.123410
50.H	-0.943155	-3.033627	-0.124217
51.H	3.495333	3.525512	0.160821
52.H	-2.823818	0.253503	-0.110573
53.H	5.697498	0.503313	0.398143
54.H	-4.306919	-0.379545	0.660512
55.H	1.965160	2.737426	0.624624
56.H	-3.032853	-3.924376	1.132929
57.H	2.674779	-3.263774	1.066603
58.H	4.463785	-1.676836	1.351653
59.H	-0.904528	2.869305	1.305343
60.H	-4.367178	-2.832315	1.569380
61.H	0.157517	-3.916284	1.418257
62.H	4.472494	2.759035	2.210662
63.H	-3.528932	1.056531	2.062442
64.H	4.724200	1.048244	2.605442
65.H	3.589249	-0.820658	2.644059
66.H	-4.764191	-1.140735	2.668653
67.H	2.769291	-3.036128	2.813233
68.H	3.106195	1.780503	2.772456

69.H	-1.774155	-3.671728	2.823730
70.H	0.847411	-3.855092	3.039220
71.H	-2.099243	1.510645	3.005614
72.H	-4.003381	-2.322558	3.769413
73.H	-3.690749	1.399184	3.797931
74.H	-0.900314	-2.618194	3.945848
75.H	-4.672566	-0.809448	4.402584
76.H	-1.059444	-0.184333	4.740667
77.H	-2.084079	-1.566107	5.216859
78.H	-2.653070	0.089554	5.469507
79.N	-1.662928	2.832204	-3.852155
80.N	-0.432518	0.794513	-1.123574
81.N	-0.363258	1.148909	0.181669
82.N	1.113312	-2.079713	1.922141
83.P	2.676187	-0.127681	0.566660
84.P	-1.642316	-1.296701	2.112822
85.P	0.763637	0.994891	3.228153
86.Re	0.463402	-0.234697	1.560056

Energy: −555.17402249 eV

Table S59. Coordinates and energy for 49

1.Mo	2.204896	-1.726994	0.006265
2.P	-0.136540	-1.792474	-0.354342
3.C	-1.695463	1.270350	-3.753386
4.C	0.233860	-0.150469	-3.313806
5.C	-2.114662	-1.019871	-2.923983
6.C	-1.177341	0.208062	-2.785668
7.C	3.245880	-3.341899	-1.266549
8.C	-1.206762	0.683238	-1.304758
9.C	1.863789	-3.783285	-1.036871
10.C	-1.562157	2.029215	-1.059014
11.C	-3.330556	4.384243	-0.576491
12.C	3.405060	0.088790	-0.712626
13.C	2.120029	0.565476	-0.431776
14.C	3.857745	-3.304306	0.010170
15.C	-0.932090	4.839580	-0.004203
16.C	-1.025927	-0.171331	-0.177806
17.C	-1.836233	2.497242	0.193812
18.C	-2.167014	3.993762	0.361057
19.C	1.695210	-3.978175	0.358206
20.C	3.985389	-0.389581	0.480523
21.C	2.907351	-3.651030	1.014315
22.C	1.867951	0.368305	0.942070
23.C	-1.499939	0.266912	1.086207
24.C	-1.875630	1.583627	1.239201
25.C	3.010430	-0.199978	1.526948
26.C	-2.595347	4.326680	1.814171
27.C	-2.411112	-1.989238	1.887932
28.C	-1.728726	-0.675716	2.325413
29.C	-0.400516	-0.949347	3.108804

30.C	-2.679364	0.009260	3.350988
31.H	-1.731907	0.841030	-4.765099
32.H	0.152300	-0.513301	-4.350848
33.H	-2.137659	-1.359008	-3.972032
34.H	-1.040858	2.153085	-3.802427
35.H	-2.711248	1.610339	-3.502582
36.H	0.882562	0.738766	-3.324701
37.H	0.728599	-0.924685	-2.714732
38.H	-3.139595	-0.757767	-2.622030
39.H	-1.779247	-1.861245	-2.299635
40.H	3.713261	-3.185609	-2.232848
41.H	-1.637702	2.706078	-1.905914
42.H	1.116409	-3.951003	-1.802543
43.H	-3.078827	4.224312	-1.633098
44.H	3.895949	0.144455	-1.681126
45.H	-0.611373	4.651196	-1.038453
46.H	1.426801	1.004312	-1.139704
47.H	-3.578782	5.450370	-0.452297
48.H	-4.231872	3.794531	-0.353337
49.H	-1.158549	5.913726	0.089373
50.H	4.893135	-3.045416	0.199630
51.H	4.991275	-0.771257	0.603875
52.H	-0.082935	4.609106	0.656093
53.H	0.785606	-4.316898	0.840355
54.H	-1.795721	-2.563794	1.178143
55.H	-3.374396	-1.778334	1.400490
56.H	0.957290	0.649914	1.455992
57.H	-2.823794	5.399374	1.897212
58.H	3.104022	-3.763969	2.075935
59.H	-3.495717	3.768563	2.106842
60.H	-2.212650	1.933196	2.208243
61.H	-1.797167	4.099633	2.535212
62.H	3.154656	-0.405763	2.583870
63.H	-2.598974	-2.626433	2.766992
64.H	0.346170	-1.460639	2.487963
65.H	-3.622422	0.332252	2.888496
66.H	0.026383	-0.004824	3.475360
67.H	-2.215668	0.871196	3.848731
68.H	-0.617970	-1.582921	3.984045
69.H	-2.923311	-0.720434	4.137728

Energy: –456.68990743 eV

Table S60. Coordinates and energy for 50

1.Th	-0.245165	0.261813	0.013313
2.P	-1.616318	2.659220	-1.504861
3.P	0.836114	3.116077	0.817119
4.C	1.763632	-0.573173	-4.835411
5.C	2.568256	-1.724439	-4.641562
6.C	1.496343	0.314037	-3.824908
7.C	3.056574	-1.910096	-3.370681

8.C	-1.105341	2.842434	-3.251975
9.C	2.022917	0.095751	-2.541453
10.C	2.845199	-1.046143	-2.359646
11.C	-0.228582	-2.581291	-2.005069
12.C	-1.685831	-0.649896	-1.848713
13.C	-3.372720	2.805422	-1.541759
14.C	0.273939	-3.792680	-1.521417
15.C	-1.302957	-1.972870	-1.427184
16.C	1.694313	1.008242	-1.437963
17.C	-1.199178	4.266274	-0.737204
18.C	-0.328594	-4.398021	-0.428340
19.C	0.265890	4.342151	-0.419154
20.C	-1.814169	-2.630730	-0.332250
21.C	-1.418057	-3.845639	0.176304
22.C	2.636580	3.453845	0.786285
23.C	-3.890669	0.177603	1.275204
24.C	-4.783190	-0.869724	1.395270
25.C	1.058421	-1.420659	1.397293
26.C	3.276070	-0.337849	1.786124
27.C	-1.798822	1.275605	1.789215
28.C	-2.736024	0.242601	1.994609
29.C	-4.514384	-1.940582	2.299295
30.C	1.985972	-0.646166	2.151297
31.C	0.398644	3.857388	2.424665
32.C	4.100335	0.466749	2.584265
33.C	-2.501843	-0.806746	2.888661
34.C	-3.397796	-1.885393	3.016781
35.C	1.628968	0.038516	3.302561
36.C	3.668240	0.974068	3.765476
37.C	2.363655	0.840671	4.156163
38.H	1.354164	-0.382919	-5.831940
39.H	2.765701	-2.421768	-5.454067
40.H	0.880373	1.190691	-4.019106
41.H	-1.610591	3.684657	-3.752847
42.H	-1.339085	1.914773	-3.791151
43.H	3.684085	-2.787561	-3.178474
44.H	-0.020531	3.000107	-3.307083
45.H	0.241663	-2.138359	-2.887807
46.H	-1.367410	-0.414044	-2.871442
47.H	-3.719702	3.710819	-2.068096
48.H	-3.836383	1.933638	-2.026289
49.H	1.131096	-4.258870	-2.008589
50.H	1.598902	2.041823	-1.801556
51.H	-2.753423	-0.422933	-1.712305
52.H	3.280343	-1.228918	-1.375364
53.H	-1.515873	5.105288	-1.381714
54.H	0.870798	4.163257	-1.324591
55.H	2.489284	0.997938	-0.671228
56.H	-3.759179	2.846358	-0.513228
57.H	0.546786	5.353299	-0.073643

58.H	0.075408	-5.350573	-0.071025
59.H	3.059429	3.166031	-0.185187
60.H	-1.804263	4.339208	0.182929
61.H	-2.692900	-2.172050	0.142876
62.H	-4.131685	0.984209	0.577303
63.H	-5.698093	-0.880479	0.800549
64.H	-1.926541	-4.298787	1.025346
65.H	2.841980	4.520186	0.969847
66.H	1.518671	-2.166236	0.733898
67.H	3.707119	-0.765699	0.874730
68.H	3.129167	2.851239	1.563773
69.H	-2.256085	2.234236	1.507533
70.H	0.289049	-1.909682	2.012693
71.H	5.132623	0.629275	2.267908
72.H	-5.222409	-2.766377	2.384358
73.H	0.755195	4.895899	2.507842
74.H	-0.685630	3.830353	2.597953
75.H	-1.132595	1.444062	2.648109
76.H	0.875482	3.254843	3.211829
77.H	-1.607099	-0.787371	3.514442
78.H	-3.153036	-2.673665	3.733728
79.H	0.590158	-0.138156	3.626386
80.H	4.370511	1.552228	4.376165
81.H	1.943450	1.256273	5.069637

Energy: −540.08479851 eV

Table S61. Coordinates and energy for 51

1.C	2.524497	-5.748569	-1.240784
2.C	2.524497	-5.748569	1.240784
3.C	4.610415	-5.175142	0.000000
4.C	3.082446	-5.035419	0.000000
5.C	-1.299386	-2.653853	-1.555958
6.C	2.701643	-3.554566	0.000000
7.C	2.585913	-2.830525	-1.187774
8.C	3.114328	-1.552701	-3.623886
9.C	-1.299386	-2.653853	1.555958
10.C	-1.989579	-1.628304	-0.708193
11.C	2.585913	-2.830525	1.187774
12.C	-3.324064	-0.385742	-2.576124
13.C	-1.989579	-1.628304	0.708193
14.C	2.215923	-1.482713	-1.224228
15.C	-2.915545	-0.625196	-1.151174
16.C	0.939892	-0.460192	-3.221419
17.C	2.311906	-0.743627	-2.587159
18.C	2.215923	-1.482713	1.224228
19.C	-2.915545	-0.625196	1.151174
20.C	-3.498511	-0.017835	0.000000
21.C	1.856139	-0.832847	0.000000
22.C	-3.324064	-0.385742	2.576124
23.C	3.114328	-1.552701	3.623886

24.C	-4.546051	1.059565	0.000000
25.C	3.083103	0.572696	-2.400237
26.C	2.311906	-0.743627	2.587159
27.C	0.939892	-0.460192	3.221419
28.C	-2.502907	3.137099	-3.352282
29.C	-1.319383	2.739369	-2.469661
30.C	3.083103	0.572696	2.400237
31.C	0.020564	3.126233	-3.071244
32.C	-1.375991	2.399512	0.000000
33.C	-1.319383	2.739369	2.469661
34.C	-2.502907	3.137099	3.352282
35.C	-1.558994	4.562607	-0.673188
36.C	0.020564	3.126233	3.071244
37.C	-1.558994	4.562607	0.673188
38.C	-1.665915	5.743948	-1.586309
39.C	-1.665915	5.743948	1.586309
40.H	2.750483	-6.824567	-1.189236
41.H	2.750483	-6.824567	1.189236
42.H	1.433968	-5.631763	-1.314219
43.H	4.907080	-6.236768	0.000000
44.H	2.968703	-5.370887	-2.171203
45.H	1.433968	-5.631763	1.314219
46.H	5.050973	-4.697706	-0.887482
47.H	2.968703	-5.370887	2.171203
48.H	-1.847038	-3.609978	-1.507889
49.H	5.050973	-4.697706	0.887482
50.H	2.805071	-3.335118	-2.124435
51.H	2.599944	-2.473633	-3.933922
52.H	-1.847038	-3.609978	1.507889
53.H	-1.263553	-2.352365	-2.608627
54.H	-0.271575	-2.837909	-1.220400
55.H	-4.096884	-1.107955	-2.889434
56.H	-0.271575	-2.837909	1.220400
57.H	4.114098	-1.820896	-3.254130
58.H	0.457694	-1.400902	-3.522307
59.H	2.805071	-3.335118	2.124435
60.H	3.243796	-0.938361	-4.527321
61.H	-2.477285	-0.497139	-3.265980
62.H	-1.263553	-2.352365	2.608627
63.H	1.069406	0.152302	-4.128492
64.H	-3.744684	0.616565	-2.716312
65.H	-4.096884	-1.107955	2.889434
66.H	2.599944	-2.473633	3.933922
67.H	0.270628	0.061988	-2.527803
68.H	-5.560445	0.629144	0.000000
69.H	4.114098	-1.820896	3.254130
70.H	0.457694	-1.400902	3.522307
71.H	-2.477285	-0.497139	3.265980
72.H	4.093269	0.378214	-2.010272
73.H	3.182071	1.091906	-3.366917

74.H	-4.462981	1.703698	-0.884333
75.H	-2.479103	2.542780	-4.276265
76.H	3.243796	-0.938361	4.527321
77.H	-3.744684	0.616565	2.716312
78.H	-1.331175	1.649577	-2.324156
79.H	0.270628	0.061988	2.527803
80.H	2.579291	1.252235	-1.697198
81.H	-4.462981	1.703698	0.884333
82.H	0.139499	2.636994	-4.048320
83.H	-3.461131	2.945395	-2.851448
84.H	1.069406	0.152302	4.128492
85.H	4.093269	0.378214	2.010272
86.H	2.579291	1.252235	1.697198
87.H	0.839856	2.793698	-2.421312
88.H	-2.473472	4.195028	-3.644806
89.H	-1.331175	1.649577	2.324156
90.H	3.182071	1.091906	3.366917
91.H	0.113671	4.209026	-3.232041
92.H	-3.461131	2.945395	2.851448
93.H	-2.479103	2.542780	4.276265
94.H	0.839856	2.793698	2.421312
95.H	0.139499	2.636994	4.048320
96.H	-2.635088	5.789196	-2.104669
97.H	-0.880048	5.755702	-2.352383
98.H	-2.473472	4.195028	3.644806
99.H	0.113671	4.209026	3.232041
100.H	-1.566933	6.669297	-1.007955
101.H	-2.635088	5.789196	2.104669
102.H	-0.880048	5.755702	2.352383
103.H	-1.566933	6.669297	1.007955
104.Ir	-1.268287	0.374595	0.000000
105.N	-1.453628	3.234348	-1.082618
106.N	-1.453628	3.234348	1.082618
107.P	0.892108	0.767379	0.000000

Energy: –691.81054132 eV

Table S62. Coordinates and energy for 52

1.C	2.137173	1.057838	-6.696964
2.C	4.351530	0.156262	-5.987969
3.C	3.104476	0.916981	-5.502830
4.C	3.530040	2.304150	-5.029942
5.C	-1.039929	-1.880602	-4.915970
6.C	2.496706	0.171595	-4.326893
7.C	1.217867	-0.312606	-4.320265
8.C	-0.782986	-1.610935	-3.395765
9.C	3.220666	-0.065651	-3.155716
10.C	0.589879	-0.942673	-3.226443
11.C	-1.928028	-0.695288	-2.991490
12.C	-0.842844	-2.955096	-2.731104
13.C	2.712094	-0.694633	-2.012336

14.C	1.286060	-0.954110	-1.948450
15.C	5.097567	-1.273854	-1.441412
16.C	-2.802064	2.513149	-1.560584
17.C	-0.236113	2.121137	-1.444888
18.C	3.652241	-1.113725	-0.871739
19.C	-1.492662	2.448957	-0.795984
20.C	-5.207878	-1.548250	-0.536563
21.C	0.962984	2.250961	-0.737496
22.C	3.282552	-2.441654	-0.323836
23.C	-4.420915	-2.676162	0.130332
24.C	3.723424	-0.102462	0.225772
25.C	-5.334047	-0.353193	0.423917
26.C	-1.413296	2.882244	0.545375
27.C	-3.041611	-2.201642	0.598164
28.C	0.993991	2.689836	0.620538
29.C	2.693978	4.480594	1.007070
30.C	-3.951004	0.127878	0.881018
31.C	2.334390	3.005319	1.244846
32.C	-0.209410	2.993158	1.249713
33.C	-3.116262	-1.012404	1.551018
34.C	2.439429	2.634320	2.730386
35.C	-0.711456	-1.884375	2.779389
36.C	0.790217	-1.799613	3.131507
37.C	1.352914	-3.215823	3.313622
38.C	-1.907148	0.783408	3.497796
39.C	-1.462711	-2.591845	3.900714
40.C	-0.913352	-3.999007	4.099861
41.C	0.585985	-3.950628	4.388366
42.C	-3.117907	0.480605	4.369528
43.C	-0.697813	1.065800	4.408413
44.C	-3.381698	1.656444	5.308398
45.C	-0.968465	2.212090	5.343671
46.C	-2.186447	1.937472	6.169176
47.H	2.632513	1.615683	-7.504237
48.H	1.843007	0.077926	-7.097134
49.H	4.825384	0.687421	-6.828512
50.H	1.225583	1.605397	-6.419714
51.H	4.087720	-0.855869	-6.327007
52.H	4.011666	2.865763	-5.846411
53.H	5.099491	0.056752	-5.190488
54.H	-1.178443	-0.955444	-5.489896
55.H	-0.223170	-2.457660	-5.371975
56.H	0.647669	-0.256147	-5.244077
57.H	-1.965882	-2.465899	-5.015853
58.H	2.665515	2.887645	-4.680731
59.H	4.246129	2.240912	-4.199562
60.H	-1.909407	0.219523	-3.600621
61.H	4.278714	0.192991	-3.153433
62.H	-2.894888	-1.193840	-3.167694
63.H	-0.071532	-3.626185	-3.137071

64.H	-1.824937	-3.423223	-2.902914
65.H	5.113992	-1.939486	-2.314783
66.H	-2.929046	1.656731	-2.231263
67.H	-0.229132	1.757439	-2.471539
68.H	-2.820331	3.424069	-2.178560
69.H	-1.873745	-0.406769	-1.933324
70.H	5.547613	-0.312954	-1.724134
71.H	-0.686015	-2.900794	-1.642176
72.H	-4.684848	-1.225341	-1.451716
73.H	3.270790	-3.217194	-1.104834
74.H	1.899841	1.987966	-1.228120
75.H	-6.204652	-1.900256	-0.845476
76.H	-3.658413	2.557087	-0.878180
77.H	5.738293	-1.712690	-0.662233
78.H	-4.298727	-3.524875	-0.560531
79.H	-5.877246	0.474597	-0.059347
80.H	4.108322	0.857002	-0.150549
81.H	-2.433446	-1.923821	-0.276462
82.H	2.666626	4.731741	-0.062461
83.H	-3.376399	0.483575	0.014735
84.H	4.005365	-2.752538	0.447637
85.H	2.285489	-2.446374	0.146719
86.H	2.738772	0.076560	0.679882
87.H	-4.994320	-3.057069	0.994247
88.H	3.079181	2.402983	0.709553
89.H	4.408092	-0.439953	1.021600
90.H	-5.934790	-0.653210	1.300307
91.H	-2.347883	3.114516	1.060183
92.H	-2.522108	-3.047333	1.072476
93.H	3.706179	4.692588	1.382072
94.H	1.991631	5.148889	1.528332
95.H	-4.053996	0.982354	1.567280
96.H	-0.742898	-2.539459	1.888208
97.H	-0.224357	3.320905	2.288253
98.H	-3.637043	-1.326379	2.470613
99.H	1.279443	-3.755928	2.354341
100.H	1.327639	-1.266272	2.336193
101.H	2.219516	1.570386	2.885160
102.H	3.455573	2.832036	3.100144
103.H	-2.127942	1.729224	2.969618
104.H	-1.096403	-4.596236	3.189541
105.H	1.749221	3.225220	3.350014
106.H	2.423868	-3.168806	3.566547
107.H	-2.542908	-2.644657	3.694765
108.H	-4.016228	0.291574	3.763046
109.H	0.192732	1.260397	3.794611
110.H	0.933558	-1.229805	4.063644
111.H	0.978435	-4.973309	4.515209
112.H	-3.631464	2.549710	4.707717
113.H	-1.445570	-4.505199	4.921035

114.H	-1.350687	-2.027041	4.842227
115.H	-1.116388	3.140970	4.761789
116.H	-2.946011	-0.429014	4.967552
117.H	-0.477737	0.162275	5.001181
118.H	0.743263	-3.443556	5.357624
119.H	-4.268229	1.445636	5.928110
120.H	-0.091847	2.392467	5.987132
121.H	-2.392954	2.778772	6.851971
122.H	-1.995470	1.064285	6.819853
123.P	0.370999	-1.116090	-0.359701
124.P	-1.460694	-0.340118	2.056864
125.Ru	-0.305135	0.856035	0.345824

Energy: −798.38729995 eV

Table S63. Coordinates and energy for 53

1.C	-2.306198	-1.154543	-4.264186
2.C	5.441328	-0.889301	-3.439241
3.C	-0.564610	-2.636523	-3.210467
4.C	5.173212	0.389572	-2.940162
5.C	4.837074	-1.981849	-2.866575
6.C	-1.482096	-1.439801	-2.995775
7.C	0.531054	0.175841	-3.007776
8.C	1.106895	1.415541	-2.685093
9.C	-0.772768	-0.176655	-2.569773
10.C	-6.168685	0.032279	-1.805408
11.C	4.286561	0.537526	-1.883705
12.C	3.949994	-1.832384	-1.796868
13.C	0.410586	2.359416	-1.859532
14.C	-1.519497	0.807746	-1.860209
15.C	1.013566	3.684567	-1.510104
16.C	-5.896633	2.430264	-1.196670
17.C	-0.931033	2.033577	-1.495217
18.C	3.673196	-0.543761	-1.304079
19.C	-5.974976	0.999285	-0.634948
20.C	-7.187229	0.912795	0.291051
21.C	-4.708501	0.658161	0.153930
22.C	-1.786103	-3.122173	0.157742
23.C	-4.257852	-0.654909	0.295957
24.C	1.852200	-2.905990	0.554486
25.C	-3.996639	1.627185	0.866108
26.C	-3.044441	-0.992829	0.910089
27.C	-4.006803	-3.328727	1.193445
28.C	3.375379	0.980047	1.047487
29.C	2.538398	-1.781773	1.030513
30.C	1.873468	-4.103022	1.238545
31.C	-2.718964	-2.481390	1.194930
32.C	4.734569	0.782434	1.341433
33.C	-2.197878	0.086182	1.316104
34.C	-2.779970	1.366175	1.490888
35.C	2.745236	2.106237	1.525458

36.C	-1.121517	3.319263	1.798456
37.C	5.420338	1.700799	2.120465
38.C	3.437703	3.045871	2.293549
39.C	3.216288	-1.897318	2.233282
40.C	2.558787	-4.199197	2.433713
41.C	4.778556	2.823028	2.601098
42.C	-2.198203	2.445351	2.452164
43.C	-2.108457	-2.622952	2.597155
44.C	-3.304976	3.377323	2.956479
45.C	3.230107	-3.096600	2.931401
46.C	-1.609219	1.758943	3.684595
47.H	-1.650807	-0.891694	-5.108124
48.H	-2.888811	-2.042574	-4.550383
49.H	6.136332	-1.015149	-4.269160
50.H	-3.008129	-0.323901	-4.110131
51.H	0.090622	-2.494008	-4.082204
52.H	5.655925	1.263765	-3.375047
53.H	5.051797	-2.983395	-3.239789
54.H	1.122936	-0.543859	-3.569828
55.H	-1.157153	-3.543282	-3.395384
56.H	2.130961	1.623330	-2.984249
57.H	-7.051325	0.325453	-2.393502
58.H	-5.298852	0.033477	-2.477019
59.H	0.070579	-2.818176	-2.333908
60.H	0.782046	4.430910	-2.286502
61.H	-2.188951	-1.680566	-2.189599
62.H	-6.783451	2.638607	-1.813885
63.H	-5.004932	2.568763	-1.824061
64.H	-6.333953	-0.999148	-1.468526
65.H	4.098396	1.538067	-1.493247
66.H	-2.523822	0.574546	-1.511155
67.H	2.105313	3.620094	-1.427424
68.H	3.495133	-2.710077	-1.343577
69.H	-1.501270	2.727040	-0.881511
70.H	-8.115088	1.159662	-0.250250
71.H	-5.872912	3.184908	-0.400050
72.H	-2.286900	-3.204988	-0.816552
73.H	0.620673	4.065203	-0.559982
74.H	1.276298	-2.817730	-0.364795
75.H	-4.879937	-1.459924	-0.088129
76.H	-4.458510	-3.411641	0.195041
77.H	-0.867123	-2.539854	0.028735
78.H	-7.297126	-0.098629	0.708384
79.H	-1.514874	-4.139682	0.480764
80.H	-7.090498	1.610692	1.134966
81.H	1.332600	-4.961886	0.842816
82.H	-4.413095	2.628905	0.935290
83.H	5.255246	-0.091949	0.952919
84.H	-1.548709	3.928097	0.987448
85.H	-3.760958	-4.349758	1.520185

86.H	1.692192	2.250759	1.290945
87.H	-0.313347	2.706318	1.382457
88.H	-4.763561	-2.926412	1.881833
89.H	6.472074	1.530913	2.350519
90.H	-3.702258	4.034629	2.170461
91.H	-0.693003	4.011743	2.540356
92.H	2.926084	3.933799	2.661378
93.H	3.731977	-1.038125	2.657039
94.H	2.566045	-5.138320	2.987401
95.H	-1.136607	-2.115512	2.678247
96.H	-1.951976	-3.687474	2.830230
97.H	5.322283	3.544771	3.211177
98.H	-4.144199	2.815907	3.389967
99.H	-2.777961	-2.198579	3.359254
100.H	-2.894134	4.031081	3.740191
101.H	-0.764626	1.100842	3.429923
102.H	3.758982	-3.158198	3.881720
103.H	-2.366657	1.145652	4.192980
104.H	-1.241909	2.513227	4.398243
105.Os	0.360707	0.410853	-0.731099
106.P	2.435382	-0.242472	0.032793
107.P	-0.335830	-0.175801	1.293112

Energy: −723.24481393 eV

Table S64. Coordinates and energy for 54

1.C	-3.462078	-0.283530	-3.267803
2.C	-1.374147	-1.471785	-2.613862
3.C	-2.688404	-0.872846	-2.079822
4.C	-1.602466	1.824971	-1.788810
5.C	-0.289811	2.099273	-1.707814
6.C	2.030151	-1.866140	-1.661079
7.C	-3.476828	-2.008277	-1.424062
8.C	4.363380	-1.673486	-0.819495
9.C	-4.938125	1.116172	-0.655238
10.C	1.911213	1.726539	-0.909399
11.C	2.900596	-1.865399	-0.380888
12.C	2.832767	1.042829	-0.208753
13.C	-3.657410	0.997113	0.176090
14.C	2.711080	-3.197913	0.332409
15.C	-3.360167	2.405196	0.725720
16.C	-3.869962	0.027261	1.342578
17.C	4.421486	-0.874379	2.368234
18.C	2.958579	-0.438619	2.368101
19.C	2.907949	0.992986	2.924844
20.C	2.124387	-1.366481	3.275148
21.H	-3.641785	-1.089520	-3.997787
22.H	-2.881829	0.500098	-3.775506
23.H	-1.603636	-2.165061	-3.439237
24.H	-4.437280	0.131703	-2.990963
25.H	-0.689707	-0.701659	-2.998135

26.H	2.449174	-2.589311	-2.379631
27.H	-2.255111	2.424197	-2.419302
28.H	-3.599447	-2.820813	-2.158549
29.H	0.146741	2.916588	-2.291417
30.H	2.004540	-0.881795	-2.148919
31.H	-0.868910	-2.045896	-1.828385
32.H	4.624966	-2.494604	-1.506745
33.H	-4.817348	1.799111	-1.508325
34.H	4.497397	-0.728043	-1.363834
35.H	2.197295	2.589463	-1.519221
36.H	1.006418	-2.181014	-1.430919
37.H	-4.483076	-1.704346	-1.107996
38.H	-5.302223	0.152238	-1.027950
39.H	-2.943839	-2.418339	-0.553927
40.H	2.939424	-4.015205	-0.371653
41.H	-5.727581	1.535115	-0.011158
42.H	5.076644	-1.701076	0.011067
43.H	3.877680	1.342484	-0.220624
44.H	-3.263243	3.148284	-0.076851
45.H	1.673911	-3.329799	0.675612
46.H	-4.102959	-0.990086	1.007882
47.H	3.379564	-3.319387	1.194461
48.H	-4.193283	2.703552	1.381585
49.H	-2.442820	2.448133	1.328651
50.H	5.044121	-0.244196	1.717011
51.H	-4.717146	0.383195	1.951760
52.H	4.558425	-1.921536	2.075403
53.H	-2.985946	-0.034162	1.989651
54.H	3.526095	1.688710	2.342316
55.H	2.124347	-2.406054	2.925898
56.H	1.888895	1.401550	2.958069
57.H	4.814697	-0.769862	3.392322
58.H	1.078978	-1.039063	3.337094
59.H	3.282896	0.975381	3.960654
60.H	2.556386	-1.347893	4.288999
61.Cl	-0.826303	-2.180046	1.209848
62.Cl	0.234019	3.305053	1.676980
63.N	0.586271	1.388866	-0.924745
64.Os	-0.121073	-0.073143	0.336373
65.P	-2.163880	0.434991	-0.831483
66.P	2.229978	-0.418748	0.627471
67.P	-0.383968	1.233020	1.920256

Energy: –413.44378374 eV

Table S65. Coordinates and energy for 55

1.Ni	0.056225	-1.111156	-0.394425
2.P	1.542379	-2.440641	-1.323176
3.P	-1.503016	-2.638293	-0.546169
4.P	-0.208247	0.948037	-0.348638
5.C	0.850814	-1.986889	-3.943324

6.C	3.148454	-2.847009	-3.685059
7.C	2.092416	-1.933732	-3.057432
8.C	2.580322	-0.485080	-3.054544
9.C	5.678450	2.217551	-2.661407
10.C	-2.585471	-1.190612	-2.640204
11.C	-3.921351	-3.183190	-2.022679
12.C	3.409232	3.129829	-1.999901
13.C	4.526555	2.340578	-1.695605
14.C	-0.833137	-3.953397	-1.675890
15.C	0.699574	-4.080058	-1.562042
16.C	1.154802	4.127851	-1.476672
17.C	-3.049777	-2.057625	-1.451452
18.C	2.354665	3.264928	-1.129641
19.C	3.697643	-4.250212	-0.659330
20.C	4.561844	1.709406	-0.467877
21.C	-3.890999	-1.151921	-0.533951
22.C	4.132253	-1.778799	-0.405436
23.C	3.065405	-2.887643	-0.305338
24.C	-2.920644	3.551046	-0.086223
25.C	2.387103	2.600616	0.092776
26.C	3.505137	1.830244	0.431582
27.C	-3.228453	-4.453813	0.928491
28.C	-4.589788	1.984762	0.924183
29.C	-3.299010	2.480789	0.900189
30.C	-1.994004	-3.550109	1.037802
31.C	0.058247	2.152896	0.990737
32.C	1.305871	2.846131	1.072870
33.C	2.618840	-2.968575	1.169660
34.C	-0.817670	-4.431094	1.451093
35.C	-6.369895	0.425804	1.791213
36.C	-4.976354	0.998598	1.817419
37.C	3.571697	1.130657	1.760087
38.C	-2.349667	1.984812	1.802219
39.C	-0.970165	2.562305	1.862726
40.C	1.484122	3.818975	2.032909
41.C	-2.216319	-2.497812	2.137023
42.C	-4.038520	0.524460	2.728060
43.C	-0.740175	3.552249	2.798784
44.C	-2.731811	1.016526	2.748035
45.C	0.483579	4.186944	2.902366
46.C	-1.732756	0.489904	3.746863
47.H	1.106294	-1.594178	-4.940707
48.H	3.295451	-2.543779	-4.735054
49.H	0.470429	-3.008746	-4.084614
50.H	2.755102	-0.168535	-4.096132
51.H	2.845108	-3.904446	-3.693967
52.H	5.502568	2.804503	-3.571257
53.H	0.045063	-1.360917	-3.539223
54.H	4.125002	-2.770704	-3.190427
55.H	-2.046680	-1.776573	-3.396924

56.H	5.841131	1.172808	-2.964549
57.H	-3.474859	-0.764225	-3.130838
58.H	3.379454	3.651639	-2.959659
59.H	-3.363082	-3.843371	-2.702405
60.H	1.833046	0.188184	-2.611584
61.H	-4.731690	-2.727033	-2.614982
62.H	6.617016	2.570653	-2.210241
63.H	-1.106053	-3.643415	-2.693600
64.H	1.111246	-4.628212	-2.423924
65.H	1.295244	4.616310	-2.449765
66.H	3.516504	-0.345729	-2.504419
67.H	-1.944527	-0.361595	-2.309865
68.H	4.082786	-4.293330	-1.684721
69.H	-1.316496	-4.928013	-1.503732
70.H	-4.392631	-3.803240	-1.253610
71.H	0.242951	3.514871	-1.524626
72.H	4.633057	-1.767128	-1.381049
73.H	-4.679945	-0.675479	-1.137341
74.H	0.990189	4.906040	-0.719306
75.H	0.945469	-4.675550	-0.672850
76.H	-3.781608	3.843587	-0.700499
77.H	2.997784	-5.084848	-0.519586
78.H	-2.121431	3.199518	-0.756341
79.H	5.429961	1.107847	-0.188847
80.H	4.548558	-4.427207	0.019277
81.H	-3.279874	-0.356726	-0.086496
82.H	3.700736	-0.787556	-0.225885
83.H	4.904169	-1.961997	0.358833
84.H	-3.142822	-5.192534	0.118361
85.H	-5.318186	2.377741	0.210254
86.H	-4.384623	-1.715673	0.268115
87.H	-2.540401	4.448394	0.422266
88.H	-4.155699	-3.884626	0.787362
89.H	-6.583413	-0.064005	0.829981
90.H	-0.643630	-5.261665	0.752392
91.H	1.993301	-3.850138	1.356396
92.H	2.060531	-2.070933	1.474696
93.H	-7.130156	1.208880	1.926322
94.H	-3.334959	-5.015666	1.871834
95.H	0.104291	-3.846927	1.547472
96.H	3.508735	-3.060598	1.812577
97.H	4.471947	0.508747	1.837481
98.H	2.689935	0.490910	1.911855
99.H	-3.057356	-1.831741	1.920904
100.H	2.449711	4.329341	2.072900
101.H	-1.032344	-4.874449	2.437127
102.H	-6.512079	-0.317267	2.585424
103.H	-1.322186	-1.872902	2.260466
104.H	3.583487	1.846213	2.594641
105.H	-2.419474	-3.011251	3.092132

106.H	-4.329275	-0.242162	3.449473
107.H	-1.560890	3.842840	3.457866
108.H	0.646383	4.967175	3.644289
109.H	-0.788685	0.217351	3.256808
110.H	-2.125569	-0.388283	4.273478
111.H	-1.484779	1.250489	4.502004

Energy: −714.71482289 eV

Table S66. Coordinates and energy for 56

1.C	-4.357311	2.127345	-3.864833
2.C	-0.910551	2.327879	-3.545062
3.C	2.472680	0.144281	-3.084515
4.C	-3.841609	1.327260	-2.719805
5.C	-5.635942	-0.472786	-2.544988
6.C	-2.310625	4.051123	-2.325833
7.C	-1.698511	2.663448	-2.291994
8.C	-4.380574	0.231251	-2.163465
9.C	4.404361	1.230728	-2.041149
10.C	2.952973	0.795592	-1.778535
11.C	2.165574	2.078207	-1.508393
12.C	0.551580	-3.762327	-1.101498
13.C	-3.325967	-2.583947	-1.051785
14.C	-2.486031	0.706738	-1.010201
15.C	2.854248	-0.233802	-0.624729
16.C	1.705225	-1.047811	-0.406322
17.C	-3.662680	-1.330749	-0.264506
18.C	3.981038	-0.429201	0.171929
19.C	-4.998793	-1.415359	0.449001
20.C	0.884580	-3.410554	0.356543
21.C	1.877022	-2.221023	0.384008
22.C	6.499757	-1.950908	1.169252
23.C	1.494998	-4.685557	0.962007
24.C	-0.700591	3.989389	0.980403
25.C	4.066326	-1.434455	1.119349
26.C	3.033775	-2.355489	1.149726
27.C	-0.400165	-3.139329	1.140063
28.C	-3.457060	2.534813	1.650455
29.C	-0.865456	2.609512	1.537488
30.C	5.301076	-1.582373	2.024116
31.C	-2.077809	1.973055	1.879958
32.C	1.660613	2.082002	1.973121
33.C	0.208580	1.750914	1.951768
34.C	5.585244	-0.235248	2.682845
35.C	-1.787966	0.733566	2.489732
36.C	-0.359268	0.596182	2.537928
37.C	5.106901	-2.603192	3.119491
38.C	-2.766805	-0.212316	3.113223
39.C	0.414384	-0.452254	3.259529
40.H	-3.584602	2.335350	-4.615671
41.H	-5.157603	1.575483	-4.372045

42.H	-1.536112	2.337147	-4.448386
43.H	2.565149	0.858381	-3.918284
44.H	-4.781496	3.092180	-3.549512
45.H	-5.985933	-0.105722	-3.517177
46.H	-0.108735	3.065147	-3.691620
47.H	-0.449582	1.336466	-3.447686
48.H	-2.884320	4.241206	-3.242322
49.H	3.080815	-0.741328	-3.322056
50.H	4.423758	1.877208	-2.930802
51.H	1.422425	-0.178783	-3.025502
52.H	-5.498817	-1.557319	-2.640900
53.H	-1.508195	4.801097	-2.285958
54.H	5.065126	0.373911	-2.235766
55.H	2.186387	2.724256	-2.401168
56.H	-6.450694	-0.304192	-1.825313
57.H	-4.057270	-2.789045	-1.845776
58.H	-2.972323	4.222716	-1.466259
59.H	1.465198	-4.028709	-1.653563
60.H	0.073674	-2.925275	-1.632478
61.H	-1.018723	2.591440	-1.431143
62.H	-2.331496	-2.488899	-1.506535
63.H	4.828618	1.814467	-1.211700
64.H	-0.133370	-4.624571	-1.134774
65.H	1.121419	1.870253	-1.244258
66.H	2.621923	2.640348	-0.681853
67.H	-3.313892	-3.454864	-0.381463
68.H	-5.817821	-1.720076	-0.215148
69.H	4.838191	0.225375	0.032132
70.H	6.683187	-1.198736	0.389789
71.H	6.350153	-2.917551	0.664548
72.H	2.439846	-4.964740	0.474299
73.H	0.146226	4.049921	0.283285
74.H	-1.598832	4.328277	0.451080
75.H	-2.868414	-1.169748	0.478018
76.H	-5.271883	-0.457597	0.911953
77.H	0.787776	-5.516409	0.821992
78.H	-3.478680	3.208157	0.784986
79.H	-0.883776	-2.206886	0.823935
80.H	-1.106679	-3.973632	0.999489
81.H	-4.934392	-2.167138	1.248097
82.H	7.413434	-2.028019	1.781881
83.H	-4.190618	1.740625	1.464691
84.H	1.889112	2.956164	1.352878
85.H	-0.507757	4.720065	1.784481
86.H	3.131465	-3.225113	1.791565
87.H	5.770223	0.552993	1.941224
88.H	2.281008	1.246569	1.624231
89.H	1.676135	-4.597477	2.042869
90.H	-0.184342	-3.065284	2.214982
91.H	-3.806213	3.108898	2.523521

92.H	4.983238	-3.622266	2.725301
93.H	-3.776249	-0.084506	2.704921
94.H	1.261454	-0.822544	2.667807
95.H	1.979351	2.322031	3.001906
96.H	6.479102	-0.299911	3.324216
97.H	4.741924	0.089235	3.309723
98.H	-2.472418	-1.260633	2.968093
99.H	5.988786	-2.618807	3.777381
100.H	4.229442	-2.372962	3.740450
101.H	-0.213032	-1.309985	3.527292
102.H	-2.838183	-0.046315	4.201854
103.H	0.822475	-0.042603	4.199357
104.N	-2.682894	1.612759	-2.001346
105.N	-3.543649	-0.137608	-1.112841
106.P	0.036739	-0.560241	-1.062178
107.Rh	-1.007657	0.690600	0.390225

Energy: –689.72211429 eV

Table S67. Coordinates and energy for 57

1.C	0.837197	1.773108	-5.079763
2.C	-0.210037	6.149871	-3.933551
3.C	0.830111	1.131523	-3.682803
4.C	-0.350200	0.149520	-3.653165
5.C	2.142846	0.335570	-3.574670
6.C	0.541708	3.533721	-2.954596
7.C	1.655985	6.857411	-2.444475
8.C	0.342899	6.052433	-2.507681
9.C	0.776305	2.211523	-2.563297
10.C	0.619340	4.605236	-2.083697
11.C	-3.545458	-0.787592	-1.966935
12.C	-1.602568	-4.837347	-1.754859
13.C	-0.677261	6.682339	-1.547790
14.C	-1.169012	-6.071960	-1.322588
15.C	-2.493752	2.194465	-1.437901
16.C	1.039203	1.945436	-1.191450
17.C	-1.301488	-3.691857	-1.020988
18.C	1.091217	4.338053	-0.796786
19.C	-3.100391	-0.229033	-0.644104
20.C	3.655016	-2.986675	-0.522983
21.C	-2.596028	1.091232	-0.427330
22.C	-0.423917	-6.178163	-0.157988
23.C	2.294850	-2.832167	-0.344851
24.C	1.357278	3.056048	-0.336525
25.C	-0.570044	-3.781962	0.157264
26.C	4.527864	-2.765904	0.527257
27.C	-0.112116	-5.037471	0.567823
28.C	-3.244003	-0.859685	0.628622
29.C	3.389473	2.076139	0.782027
30.C	-3.876830	-2.196577	0.883109
31.C	1.768180	-2.472873	0.901363

32.C	2.125532	2.912972	1.009599
33.C	-2.447343	1.263908	0.989248
34.C	2.616391	4.276676	1.534952
35.C	-2.144250	2.574895	1.650257
36.C	-2.825291	0.059249	1.632804
37.C	4.025205	-2.395567	1.758276
38.C	2.654477	-2.255252	1.948544
39.C	1.293496	2.315571	2.146915
40.C	-0.388365	-2.509791	2.788971
41.C	-2.933185	-0.170645	3.119078
42.C	-1.076713	-3.608729	3.294207
43.C	0.003904	-1.502018	3.673910
44.C	-1.326900	-3.709699	4.656575
45.C	-0.206954	-1.636250	5.037175
46.C	-0.863701	-2.737206	5.529614
47.H	0.971118	0.979854	-5.830190
48.H	-0.107337	2.282808	-5.317453
49.H	1.661130	2.489578	-5.202092
50.H	0.508941	5.779330	-4.677566
51.H	-0.424797	7.200637	-4.177486
52.H	2.201079	-0.403430	-4.389426
53.H	-0.197749	-0.636229	-4.410518
54.H	-1.144120	5.581323	-4.047098
55.H	0.289287	3.732035	-3.990619
56.H	-1.288076	0.668447	-3.901282
57.H	3.012998	1.004088	-3.651200
58.H	2.410664	6.428255	-3.119259
59.H	1.482048	7.903775	-2.742658
60.H	2.219425	-0.205869	-2.618998
61.H	-2.845901	-0.536497	-2.771990
62.H	-0.464818	-0.330664	-2.673020
63.H	-2.171792	-4.750046	-2.681071
64.H	-2.328777	1.805967	-2.447898
65.H	-4.529592	-0.374840	-2.243450
66.H	-1.404198	-6.966327	-1.901441
67.H	-0.867367	7.732000	-1.822284
68.H	-3.652324	-1.877918	-1.930383
69.H	-1.634376	6.141796	-1.578544
70.H	2.077874	6.861846	-1.430252
71.H	4.037750	-3.272927	-1.502924
72.H	-3.432805	2.772868	-1.453984
73.H	-1.606905	-2.703498	-1.363659
74.H	-1.675209	2.885655	-1.207637
75.H	1.624298	-2.998937	-1.187074
76.H	-0.318857	6.668387	-0.509511
77.H	1.289747	5.181532	-0.140374
78.H	-0.062817	-7.149811	0.178866
79.H	4.022527	2.528093	0.004277
80.H	-3.744728	-2.885381	0.040905
81.H	5.602749	-2.883501	0.384209

82.H	3.154031	1.047093	0.470061
83.H	3.256670	4.796948	0.808807
84.H	-4.960036	-2.080250	1.049180
85.H	-1.442105	3.173025	1.058377
86.H	0.511024	-5.123417	1.458437
87.H	-3.462105	-2.680465	1.776905
88.H	3.975185	2.019558	1.713178
89.H	-3.074147	3.158021	1.757672
90.H	1.789587	4.945328	1.814374
91.H	0.819110	1.367136	1.865503
92.H	3.213650	4.105450	2.442435
93.H	4.699986	-2.222756	2.597139
94.H	0.503690	3.013570	2.455704
95.H	-1.724483	2.437120	2.653295
96.H	-1.432898	-4.391179	2.626237
97.H	2.288119	-1.981308	2.936196
98.H	1.940682	2.136253	3.021153
99.H	-2.831024	-1.230206	3.377122
100.H	-3.917785	0.166648	3.484399
101.H	0.470835	-0.599952	3.280326
102.H	-2.169916	0.391008	3.670436
103.H	-1.876536	-4.569942	5.039296
104.H	0.133563	-0.850489	5.712627
105.H	-1.045078	-2.837156	6.600371
106.Ir	-1.025709	-0.343257	0.275564
107.P	0.962287	0.215621	-0.517281
108.P	-0.052356	-2.232855	1.008163

Energy: –726.69666914 eV

Table S68. Coordinates and energy for 58

1.C	-1.532659	-2.138973	-3.663571
2.C	0.125431	-0.382032	-3.152312
3.C	-2.341071	-0.051586	-2.676418
4.C	-1.162223	-1.033670	-2.640168
5.C	1.738387	3.933624	-2.581213
6.C	-1.300367	4.281633	-1.578438
7.C	1.268633	3.886154	-1.157082
8.C	-0.910449	-1.631059	-1.229445
9.C	-0.496231	-2.968662	-1.185675
10.C	1.112118	-5.401701	-1.239468
11.C	-0.098991	4.051565	-0.716267
12.C	-0.943047	-6.065575	0.000000
13.C	-1.015210	-0.908215	0.000000
14.C	2.105283	3.817555	0.000000
15.C	0.241735	-5.113660	0.000000
16.C	-0.235739	-3.646086	0.000000
17.C	3.608513	3.728036	0.000000
18.C	1.691618	0.530028	0.000000
19.C	-0.098991	4.051565	0.716267
20.C	1.268633	3.886154	1.157082

21.C	-1.300367	4.281633	1.578438
22.C	-0.910449	-1.631059	1.229445
23.C	-0.496231	-2.968662	1.185675
24.C	1.112118	-5.401701	1.239468
25.C	-2.341071	-0.051586	2.676418
26.C	1.738387	3.933624	2.581213
27.C	-1.162223	-1.033670	2.640168
28.C	0.125431	-0.382032	3.152312
29.C	-1.532659	-2.138973	3.663571
30.H	-1.829068	-1.659588	-4.607478
31.H	-0.015853	-0.020429	-4.184684
32.H	-2.504577	0.295493	-3.708389
33.H	-0.689053	-2.801490	-3.896683
34.H	-2.373392	-2.755325	-3.315177
35.H	0.998198	3.500441	-3.264677
36.H	1.919017	4.973254	-2.900023
37.H	0.948854	-1.110319	-3.157174
38.H	-1.163782	3.860255	-2.581503
39.H	-3.264645	-0.527496	-2.317214
40.H	2.675396	3.379741	-2.713941
41.H	0.427384	0.469742	-2.528985
42.H	-2.173828	0.846457	-2.058663
43.H	-1.494543	5.359904	-1.692236
44.H	0.538207	-5.336381	-2.173532
45.H	-0.373381	-3.501140	-2.122945
46.H	-2.198686	3.825059	-1.144791
47.H	1.955955	-4.701362	-1.307073
48.H	1.515940	-6.423550	-1.180119
49.H	-1.576418	-5.917038	-0.887238
50.H	3.981561	3.200178	-0.885853
51.H	-0.600397	-7.113010	0.000000
52.H	4.055436	4.734479	0.000000
53.H	-1.576418	-5.917038	0.887238
54.H	-2.198686	3.825059	1.144791
55.H	3.981561	3.200178	0.885853
56.H	-1.494543	5.359904	1.692236
57.H	1.515940	-6.423550	1.180119
58.H	1.955955	-4.701362	1.307073
59.H	-3.264645	-0.527496	2.317214
60.H	-2.173828	0.846457	2.058663
61.H	-0.373381	-3.501140	2.122945
62.H	-1.163782	3.860255	2.581503
63.H	0.538207	-5.336381	2.173532
64.H	1.919017	4.973254	2.900023
65.H	0.427384	0.469742	2.528985
66.H	2.675396	3.379741	2.713941
67.H	-2.373392	-2.755325	3.315177
68.H	0.998198	3.500441	3.264677
69.H	-2.504577	0.295493	3.708389
70.H	0.948854	-1.110319	3.157174

71.H	-0.689053	-2.801490	3.896683
72.H	-0.015853	-0.020429	4.184684
73.H	-1.829068	-1.659588	4.607478
74.Ir	0.628178	2.043101	0.000000
75.O	2.361525	-0.395052	0.000000
76.P	-1.242541	0.927164	0.000000

Energy: –497.87193212 eV

Table S69. Coordinates and energy for 59

1.C	1.930551	-2.288985	-2.771437
2.C	-1.467960	-1.696475	-2.795043
3.C	2.916756	-1.282270	-2.617142
4.C	-0.467789	-3.954991	-2.495146
5.C	4.020905	1.419171	-2.448172
6.C	0.857249	-1.912284	-1.981429
7.C	-0.518191	-2.540992	-1.910613
8.C	-1.748337	3.079696	-1.825819
9.C	2.405398	-0.331273	-1.748037
10.C	3.111998	0.938687	-1.293320
11.C	2.486197	3.440432	-0.952323
12.C	2.171648	2.087169	-0.961487
13.C	1.296294	4.146064	-0.630538
14.C	-1.122997	-2.593482	-0.518839
15.C	0.298642	3.199565	-0.458536
16.C	-1.199448	3.414531	-0.416030
17.C	-1.481837	4.897794	-0.162392
18.C	4.039650	0.643826	-0.090780
19.C	-1.792358	-3.630683	0.118893
20.C	-1.942686	2.570556	0.599788
21.C	2.259403	-2.688494	0.798110
22.C	-3.123590	2.897646	1.248264
23.C	-2.415595	-3.072006	1.269273
24.C	-2.111937	-1.715724	1.281877
25.C	-2.624189	0.738378	1.677012
26.C	-3.551929	1.737086	1.936434
27.C	1.920612	-1.877741	2.046141
28.C	-2.611814	-0.662631	2.264277
29.C	-4.058429	-1.033905	2.655246
30.C	3.204853	-1.627230	2.855370
31.C	0.941140	-2.680173	2.912886
32.C	-1.795178	-0.667291	3.576417
33.H	-1.086795	-1.647863	-3.828414
34.H	-0.105506	-3.926945	-3.532812
35.H	2.000491	-3.167193	-3.408287
36.H	3.425654	1.642114	-3.342752
37.H	3.883707	-1.259169	-3.113897
38.H	4.765484	0.650085	-2.703601
39.H	-2.483462	-2.126769	-2.796383
40.H	-1.473212	-4.400511	-2.497507
41.H	-1.242367	3.689888	-2.592016

42.H	-1.534475	-0.673537	-2.405003
43.H	4.566379	2.326803	-2.153981
44.H	0.200533	-4.604570	-1.913093
45.H	-2.835310	3.261006	-1.867396
46.H	-1.568794	2.023427	-2.062818
47.H	3.453063	3.889556	-1.168709
48.H	-1.006624	5.513848	-0.939209
49.H	1.185342	5.227369	-0.596773
50.H	4.718344	-0.193017	-0.320591
51.H	-2.562555	5.094896	-0.191092
52.H	-1.879688	-4.659967	-0.221397
53.H	4.629578	1.539155	0.165246
54.H	2.905455	-2.145250	0.095814
55.H	1.348622	-2.980493	0.265075
56.H	-1.098626	5.212566	0.817753
57.H	3.442506	0.384340	0.794498
58.H	2.786108	-3.620398	1.097395
59.H	-3.641859	3.853108	1.220837
60.H	-4.715983	-1.033034	1.776365
61.H	-3.051282	-3.606693	1.971472
62.H	3.985585	-1.148515	2.247048
63.H	-4.444888	1.655632	2.551385
64.H	0.003056	-2.889180	2.380553
65.H	-4.084578	-2.034401	3.110386
66.H	1.407184	-3.649324	3.186238
67.H	3.607427	-2.591543	3.221035
68.H	-4.452919	-0.321196	3.394474
69.H	-0.762284	-0.338473	3.395080
70.H	3.017978	-0.982551	3.730158
71.H	0.698699	-2.156214	3.850929
72.H	-1.770574	-1.677089	4.015646
73.H	-2.247627	0.034485	4.296096
74.N	1.144593	-0.727080	-1.346678
75.N	0.839707	1.938597	-0.624243
76.N	-1.289465	-1.438110	0.207712
77.N	-1.629089	1.250682	0.862143
78.P	1.166723	-0.166817	1.718214
79.Ru	-0.054729	0.200118	0.030205

Energy: −545.51817191 eV

Table S70. Coordinates and energy for 60

1.C	2.105061	2.673618	-3.556257
2.C	-0.796444	2.853326	-3.163512
3.C	-2.124796	2.411571	-2.943493
4.C	-2.958396	-0.848220	-2.384507
5.C	0.029880	2.102437	-2.333006
6.C	3.510015	0.484880	-2.167195
7.C	1.550649	2.203668	-2.198589
8.C	-4.510930	1.047186	-1.962835
9.C	-2.066608	1.418449	-1.976553

10.C	2.226268	0.883628	-1.826275
11.C	3.707635	-0.800307	-1.603457
12.C	-3.159849	0.446078	-1.559686
13.C	1.942621	3.316723	-1.197454
14.C	1.114798	-3.142448	-1.219169
15.C	2.542297	-1.123752	-0.947078
16.C	3.382958	-3.342575	-0.180985
17.C	2.145560	-2.436894	-0.294795
18.C	-3.197264	0.066244	-0.092728
19.C	-4.304898	-0.246697	0.686813
20.C	1.503092	-2.272750	1.065818
21.C	-3.819243	-0.735644	1.925547
22.C	-2.434863	-0.713792	1.856568
23.C	2.875796	0.711175	1.948591
24.C	1.798711	-2.870143	2.277845
25.C	-0.125781	-1.687074	2.463004
26.C	1.986755	2.708337	3.034224
27.C	-1.452231	-1.115425	2.958464
28.C	0.757853	-2.491898	3.179108
29.C	-2.125341	-2.223529	3.801343
30.C	-1.210648	0.065977	3.927992
31.H	1.881102	1.946600	-4.347915
32.H	1.664922	3.641642	-3.834028
33.H	-0.491498	3.617127	-3.875022
34.H	3.195663	2.806466	-3.501724
35.H	-3.008290	2.766794	-3.467843
36.H	-2.956746	-0.623215	-3.463885
37.H	-4.530746	1.253088	-3.042289
38.H	4.224608	1.030478	-2.778625
39.H	-3.754064	-1.578251	-2.159423
40.H	1.542269	-3.298350	-2.223428
41.H	-1.994495	-1.309850	-2.134405
42.H	-5.327209	0.345034	-1.739716
43.H	4.592328	-1.422534	-1.714245
44.H	-4.700681	1.984533	-1.423139
45.H	1.521458	4.282117	-1.522821
46.H	0.216243	-2.522378	-1.320761
47.H	3.040229	3.396998	-1.133134
48.H	3.814962	-3.523568	-1.175507
49.H	0.812788	-4.112551	-0.791011
50.H	1.552282	3.107276	-0.193739
51.H	3.102452	-4.311786	0.256608
52.H	4.153074	-2.883427	0.454010
53.H	-5.350884	-0.154526	0.403842
54.H	2.657984	-0.011297	1.161765
55.H	3.787721	1.274110	1.674008
56.H	2.623247	-3.545528	2.494721
57.H	-4.430510	-1.067785	2.760839
58.H	2.867997	3.310435	2.735688
59.H	3.067170	0.165653	2.890692

60.H	1.106446	3.366475	3.063728
61.H	-2.329282	-3.109111	3.185880
62.H	-0.784137	0.929179	3.398515
63.H	2.172721	2.325092	4.056725
64.H	0.654348	-2.829193	4.208023
65.H	-3.074557	-1.865350	4.227879
66.H	-2.165098	0.387590	4.376542
67.H	-1.473268	-2.520374	4.635036
68.H	-0.511708	-0.232565	4.726144
69.N	-0.752969	1.246287	-1.588947
70.N	1.639450	-0.093007	-1.060879
71.N	-2.055377	-0.225782	0.627819
72.N	0.353104	-1.526711	1.190272
73.N	1.747030	1.629508	2.103097
74.P	0.308601	1.597092	1.263994
75.Ru	-0.135757	0.040396	-0.046776

Energy: –523.08654911 eV

Table S71. Coordinates and energy for 61

1.C	0.443847	2.906776	-5.068048
2.C	-0.727184	3.474939	-4.645656
3.C	1.076778	1.978931	-4.270134
4.C	-1.265484	3.131396	-3.422019
5.C	0.535918	1.630588	-3.043607
6.C	-4.233339	-0.550558	-2.772539
7.C	-3.184073	0.346080	-2.603037
8.C	-0.639453	2.205100	-2.599635
9.C	2.558748	-1.894894	-2.277333
10.C	-4.856976	-1.114296	-1.682067
11.C	4.745833	-2.039289	-1.177713
12.C	-2.739165	0.663009	-1.324924
13.C	3.328070	-1.460674	-1.019519
14.C	3.468542	0.070002	-0.986056
15.C	-3.527011	3.497050	-0.453894
16.C	-2.152265	3.348575	-0.523228
17.C	-4.426787	-0.794296	-0.406973
18.C	-4.089165	4.728499	-0.136351
19.C	-1.357473	4.465028	-0.286619
20.C	-3.379932	0.079011	-0.231522
21.C	-3.286063	5.818121	0.109247
22.C	-1.920047	5.687096	0.031033
23.C	-1.256366	-3.824533	0.064773
24.C	2.628334	-2.018923	0.250524
25.C	1.211725	-2.061299	0.429054
26.C	3.431218	-2.553468	1.250235
27.C	0.691638	-2.946127	1.420328
28.C	-0.792314	-3.397857	1.464219
29.C	1.006368	2.483500	1.599528
30.C	1.627046	1.254509	1.796706
31.C	-1.721800	-2.319975	2.015001

32.C	-0.298611	2.363834	2.130116
33.C	-0.973731	-4.642763	2.363783
34.C	2.934138	-3.217009	2.361934
35.C	1.566842	-3.461182	2.371810
36.C	0.745487	0.400269	2.404353
37.C	5.051808	-4.462969	2.910060
38.C	-0.448373	1.083887	2.623498
39.C	3.876214	-3.676610	3.484857
40.C	4.409444	-2.422511	4.191491
41.C	3.174848	-4.554664	4.505122
42.H	0.876510	3.186730	-6.030289
43.H	-1.239432	4.210348	-5.267499
44.H	2.008840	1.516953	-4.597288
45.H	-4.559288	-0.805276	-3.781759
46.H	-2.695371	0.777497	-3.477057
47.H	-2.193453	3.606262	-3.099628
48.H	3.073149	-1.526041	-3.178233
49.H	1.043693	0.903201	-2.411883
50.H	2.498598	-2.991178	-2.336042
51.H	1.527694	-1.505633	-2.294039
52.H	5.150844	-1.718776	-2.148502
53.H	-5.677018	-1.819682	-1.823561
54.H	3.939890	0.424611	-1.916254
55.H	4.740196	-3.138271	-1.154908
56.H	2.498909	0.568643	-0.870719
57.H	-4.181104	2.647670	-0.651592
58.H	5.439983	-1.679948	-0.406417
59.H	-1.212171	-2.993859	-0.656426
60.H	-5.174397	4.822650	-0.086004
61.H	-0.272123	4.376405	-0.354954
62.H	-0.629413	-4.642043	-0.319633
63.H	4.111095	0.377195	-0.147565
64.H	-3.731679	6.781803	0.360911
65.H	-1.272089	6.545382	0.214489
66.H	-2.298928	-4.176008	0.106834
67.H	-4.911783	-1.239942	0.462488
68.H	-3.021717	0.306718	0.772744
69.H	4.510608	-2.463192	1.147870
70.H	1.443300	3.366461	1.146705
71.H	-1.688240	-1.408883	1.405783
72.H	2.643519	1.008376	1.511021
73.H	-0.313456	-5.465726	2.057150
74.H	5.638349	-3.869166	2.197299
75.H	-2.759089	-2.690228	2.026894
76.H	4.704378	-5.366497	2.388004
77.H	-1.056435	3.140137	2.131790
78.H	-2.013202	-4.988990	2.273684
79.H	0.950714	-0.627729	2.683274
80.H	-1.442866	-2.053690	3.046429
81.H	1.163336	-4.094799	3.155568

82.H	-1.339342	0.682951	3.095207
83.H	-0.793792	-4.426930	3.426198
84.H	5.730708	-4.775125	3.718843
85.H	4.948138	-1.770959	3.489538
86.H	2.777719	-5.471669	4.046551
87.H	3.587866	-1.838876	4.630942
88.H	5.104120	-2.701764	4.999938
89.H	2.342770	-4.030699	4.996329
90.H	3.885149	-4.853732	5.289654
91.Co	-0.011363	0.930961	0.590541
92.P	-1.327502	1.761642	-0.949280
93.P	0.095370	-0.883258	-0.481971

Energy: −634.22359191 eV
